# *Pheidole* Westwood, 1839 (Hymenoptera, Formicidae) of Madagascar – an introduction and a taxonomic revision of eleven species groups

**DOI:** 10.3897/zookeys.905.39592

**Published:** 2020-01-20

**Authors:** Sebastian Salata, Brian L. Fisher

**Affiliations:** 1 Department of Entomology, California Academy of Sciences, San Francisco, CA 94118, USA Department of Entomology, California Academy of Sciences San Francisco United States of America

**Keywords:** endemic species, Malagasy region, Myrmicinae, taxonomy

## Abstract

The present study represents an introduction to the revision of *Pheidole* Westwood, 1839 from Madagascar. Sixteen species groups are established, of which eleven are revised below, and illustrated identification keys to species groups and species of groups revised in this monograph are presented. Two species are raised to species level: *Pheidole petax* Forel, 1895 **stat. nov.**, and *P. scabrata* Forel, 1895 **stat. nov.** We also redescribe worker castes and designate lectotypes for *P. annemariae* Forel, 1918, *P. nemoralis* Forel, 1892, *P. petax* Forel, 1895, *P. ensifera* Forel, 1897, *P. longispinosa* Forel, 1891, and *P. scabrata* Forel, 1895. The following 46 new species are described: *Pheidole aelloea***sp. nov.**, *P. ala***sp. nov.**, *P. andapa***sp. nov.**, *P. ankerana***sp. nov.**, *P. avaratra***sp. nov.**, *P. bemarahaensis***sp. nov.**, *P. bemarivoensis***sp. nov.**, *P. binara***sp. nov.**, *P. boribora***sp. nov.**, *P. brevipilosa***sp. nov.**, *P. curvistriata***sp. nov.**, *P. diakritos***sp. nov.**, *P. ehazoara***sp. nov.**, *P. ferruginea***sp. nov.**, *P. fisaka***sp. nov.**, *P. fitarata***sp. nov.**, *P. glabra***sp. nov.**, *P. goavana***sp. nov.**, *P. lamperos***sp. nov.**, *P. longipilosa***sp. nov.**, *P. lutea***sp. nov.**, *P. madinika***sp. nov.**, *P. mahaboensis***sp. nov.**, *P. maizina***sp. nov.**, *P. makaensis***sp. nov.**, *P. makirovana***sp. nov.**, *P. manantenensis***sp. nov.**, *P. mantadia***sp. nov.**, *P. marieannae***sp. nov.**, *P. masoala***sp. nov.**, *P. mavesatra***sp. nov.**, *P. miramila***sp. nov.**, *P. moramanaensis***sp. nov.**, *P. navoatrensis***sp. nov.**, *P. ocypodea***sp. nov.**, *P. parviocula***sp. nov.**, *P. podargea***sp. nov.**, *P. praegrandis***sp. nov.**, *P. ranohirensis***sp. nov.**, *P. rugocephala***sp. nov.**, *P. rugofitarata***sp. nov.**, *P. typhlos***sp. nov.**, *P. vatovavensis***sp. nov.**, *P. voasara***sp. nov.**, *P. vohemarensis***sp. nov.**, and *P. zavamanira***sp. nov.** At present, there are 69 valid species and subspecies of *Pheidole* known from Madagascar, but this number is expected to increase significantly with upcoming taxonomic revisions of the species groups not revised in this study.

## Introduction

The genus *Pheidole* Westwood, 1839 is one of the most diverse ant genera and contains 1047 valid species and 134 valid subspecies (Bolton 2019). While distribution of the genus is worldwide, its species richness varies with zoogeographic region. The Neotropical region is the most species-rich; the current number of described taxa constitutes nearly 50 % of all known *Pheidole* species, with far fewer species numbers in other regions. The fauna of this genus was exhaustively studied by Wilson (2003), and later amended and updated by Longino (2009, 2019). The taxonomy of the genus has been revised for the Nearctic (Wilson 2003), Afrotropic (Fischer et al. 2012), Indomalayan (Eguchi 2001, 2006, 2008; Eguchi and Bui 2005; Eguchi et al. 2016), Australasian (Sarnat et al. 2016), and Oceanian (Sarnat 2008; Fischer et al. 2016) regions. The taxonomy of the Malagasy region has been greatly improved in recent years by Fischer and Fisher (2013). Covering Comoros, Juan de Nova Island, Mauritius, Mayotte, Réunion, and Seychelles, their survey confirmed the presence of thirteen species on these islands, of which seven were newly described.

Very little is known about the *Pheidole* fauna of Madagascar. Most of the data describing the diversity of this genus on the island can be found in a number of short faunistic notes, with the majority of species descriptions originating in the late 1800s. There are only 18 valid *Pheidole* taxa described from Madagascar, of which 17 were described before the twentieth century (Forel 1891, 1892, 1895a, b, 1897; Emery 1899). The last Madagascar *Pheidole* species was described a century ago (Forel 1918). Since the last publication of Forel (1918), almost 80 years passed without additional work on the *Pheidole* of Madagascar. In late 1990s Fisher (1997) provided a complete overview of the species described from the Malagasy region and discussed their known distributions. He also listed *Pheidole* within the four most species-rich and abundant ant genera of Madagascar. Later, Fisher and Peeters (2019) estimated that the number of undescribed endemic species exceeded 100.

Thanks to comprehensive inventories conducted across the island by Fisher and members of the Malagasy Arthropod team at the Madagascar Biodiversity Centre in Madagascar, the *Pheidole* collection deposited at the California Academy of Sciences (CAS) now contains more than 50,000 specimens from almost 4,500 localities. Recent inventories have yielded a large number of undescribed *Pheidole* species, confirming assumptions presented by Fisher and Peeters (2019).

Here we present an introduction to *Pheidole* in Madagascar, define species groups, and provide an illustrated identification key to species groups. This monograph is a first attempt to define species groups for the Malagasy region. Our divisions are based mostly on morphological similarities within taxa. We recognize 16 species groups containing approximately 140 species. In this work we define and revise eleven groups containing 52 species, 46 of which are described as new. This publication is meant to be the first in a series aiming to revise the taxonomy of all *Pheidole* in Madagascar.

Based on data gathered thus far, *Pheidole* of Madagascar are distinct from species known from other islands in the Malagasy region. Only three species are confirmed from Madagascar as well as surrounding islands. Two are invasive worldwide: *P. megacephala* (Fabricius) and *P. indica* Mayr. The third, *P. megatron* Fischer & Fisher, is a member of the *megacephala* group described from Comoros. Here we confirm its presence in urban areas of Antsiranana prefecture. Three other species absent from Madagascar but found on nearby islands can be assigned to Malagasy species groups. *Pheidole ragnax* Fischer & Fisher is a member of the *P. bessonii* group and should be assigned to the *P. grallatrix* complex. *Pheidole jonas* Forel is similar to members of the *P. bemarivoensis* complex of the *P. nemoralis* group. Finally, *Pheidole vulcan* Fischer & Fisher represents a distinct member of the *P. petax* group and most likely is closely related to species grouped within *P. ankerana* complex. *Pheidole braueri* Forel, *P. dodo* Fischer & Fisher, and *P. komori* Fischer & Fisher bear a distinct set of characters unknown from Madagascar. *Pheidole loki* Fischer & Fisher cannot be assigned to any known species group until its major workers desctiption. However, based on the morphology of minor workers, it appears most similar to members of the *P. makaensis* group.

Among the 16 species groups known from Madagascar defined here, members of only three are reported from outside the Malagasy region. The *P. fervens* group is native to Indoaustralia (Sarnat et al 2015), while the *P. megacephala* group is considered native to the Afrotropics and Malagasy region (Sarnat et al 2015). Members of the *P. bessonii* group are morphologically reminiscent of the *P. longipes* group from Indo-Australia. In conclusion, the vast majority of groups and species are endemic to Madagascar, one group is also native to the Afrotropical Region, and two groups are of Indo-Australian origin.

## Materials and methods

The majority of the material was collected by Brian L. Fisher and members of the Madagascar Biodiversity Centre from across Madagascar between 1991 and 2018. The study was supported with material deposited in the Museum d’Historie Naturelle, Geneva, Switzerland.

Repositories. Collections are referred to by the following acronyms:

**CASC**California Academy of Sciences, San Francisco, California, USA;

**MHNG**Museum d’Historie Naturelle, Geneva, Switzerland.

All observations and measurements were taken using a pin-holding stage, permitting rotations around the X, Y, and Z axes at magnifications from 32× to 100× with a Leica MZ12.5 microscope and an orthogonal crosshair micrometre, at an accuracy of 0.01 mm to approximately 0.005 mm. All measurements are presented in mm units as minimum and maximum values, with the arithmetic mean in parentheses. We attach the measurement data in the Supplementary file. Photographs were taken using a JVC KY-75 or Leica DFC450 digital camera with a Leica Z16 APO microscope and Leica Application Suite software (v3.8). Unless stated otherwise, photographs were taken by Michele Esposito. Images of specimens and data of all pinned specimens examined in the present contribution are available online on AntWeb (https://www.AntWeb.org) and accessible using the unique CASENT identifying specimen code. Most measurements and indices are the same as in Longino (2009, 2019) and based on several other revisions (Eguchi 2008; Fischer et al. 2012; Fischer and Fisher 2013; Wang et al. 2018). The general morphological terminology follows Wilson (2003) and Longino (2009, 2019). As older taxa are often insufficiently characterized by their original describers, diagnoses are provided in the redescriptions for *P. annemariae* Forel, 1918, *P. nemoralis* Forel, 1892, *P. petax* Forel, 1895, *P. ensifera* Forel, 1897, *P. longispinosa* Forel, 1891, and *P. scabrata* Forel, 1895 to make identifications easier.

Pilosity inclination degree follows that used in Wilson (1955). Appressed (0–5°) hairs run parallel or nearly parallel to the body surface. Decumbent hairs stand 10–40°, subdecumbent hair stand ~45° from the surface°, suberect hairs bend about 10°–20° from vertical, and erect hairs stand vertical or nearly vertical.

Maps were generated using tmap v2.2 package on R v3.5. R Core Team (2018). The concepts of ecotones follow those used by Yoder and Nowak (2006).

### Measurements and indices

Measurements:

**EL** eye length; measured along the maximum vertical diameter of eye;

**HL** maximum distance from the midpoint of the anterior clypeal margin to the midpoint of the posterior margin of the head, measured in full-face view; in majors from midpoint of tangent between anteriormost position of clypeus to midpoint of tangent between posteriormost projection of the vertex;

**HW** head width; measured in full-face view, at widest point of the head, directly above the eyes;

**MTL** metatibia length; measured from the junction with femur to the junction with first tarsal segment;

**PNW** pronotum width; maximum width of promesonotum measured in dorsal view;

**PPW** postpetiole width; maximum width of postpetiole in dorsal view;

**PSL** propodeal spine length; measured from the centre of the propodeal spiracle to the tip of the propodeal spine in lateral view;

**PTW** petiole width; maximum width of petiole in dorsal view;

**SL** scape length; maximum straight-line length of scape excluding the basal condylar bulb;

**WL** mesosoma length (Weber’s length); diagonal length of mesosoma in lateral view from the anterior point of the pronotal slope and excluding the neck, to the posteroventral margin of the propodeum.

Indices:

**CI** cephalic index: HW / HL * 100;

**MTI** tibia index: MTL / HW * 100;

**SI** scape index: SL / HW * 100;

**PNI** pronotum index: PNW / HW * 100;

**PPI** postpetiole width index: PPW / PTW * 100;

**PSLI** propodeal spine index: PSL / HW * 100.

Abbreviations:

**m**. male;

**q**. gyne;

**s** major worker;

**w**. minor worker.

### Synopsis of species of Madagascar examined in this study

***Pheidole annemariae* group**

*Pheidole annemariae* Forel, 1918

*Pheidole marieannae* sp. nov.

***Pheidole curvistriata* group**

*Pheidole curvistriata* sp. nov.

*Pheidole makirovana* sp. nov.

*Pheidole mantadia* sp. nov.

*Pheidole moramanaensis* sp. nov.

***Pheidole diakritos* group**

*Pheidole diakritos* sp. nov.

***Pheidole ensifera* group**

*Pheidole ensifera* Forel, 1897

*Pheidole ocypodea* sp. nov.

*Pheidole aelloea* sp. nov.

*Pheidole podargea* sp. nov.

***Pheidole ferruginea* group**

*Pheidole longipilosa* complex

*Pheidole longipilosa* sp. nov.

*Pheidole ferruginea* complex

*Pheidole ferruginea* sp. nov.

*Pheidole rugocephala* sp. nov.

*Pheidole vohemarensis* sp. nov.

*Pheidole manantenensis* sp. nov.

***Pheidole longispinosa* group**

*Pheidole scabrata* complex

*Pheidole scabrata* Forel, 1895 stat. nov.

*Pheidole maizina* sp. nov.

*Pheidole longispinosa* complex

*Pheidole longispinosa* Forel, 1891

*Pheidole praegrandis* sp. nov.

*Pheidole mahaboensis* sp. nov.

***Pheidole lutea* group**

*Pheidole lutea* complex

*Pheidole lutea* sp. nov.

*Pheidole ranohirensis* sp. nov.

*Pheidole voasara* sp. nov.

*Pheidole navoatrensis* complex

*Pheidole navoatrensis* sp. nov.

*Pheidole parviocula* sp. nov.

*Pheidole typhlos* sp. nov.

***Pheidole makaensis* group**

*Pheidole makaensis* sp. nov.

*Pheidole fitarata* sp. nov.

*Pheidole rugofitarata* sp. nov.

*Pheidole ehazoara* sp. nov.

*Pheidole avaratra* sp. nov.

***Pheidole masoala* group**

*Pheidole masoala* complex

*Pheidole masoala* sp. nov.

*Pheidole madinika* sp. nov.

*Pheidole fisaka* sp. nov.

*Pheidole binara* sp. nov.

*Pheidole andapa* sp. nov.

*Pheidole lamperos* complex

*Pheidole lamperos* sp. nov.

*Pheidole zavamanira* complex

*Pheidole zavamanira* sp. nov.

***Pheidole nemoralis* group**

*Pheidole bemarivoensis* complex

*Pheidole bemarahaensis* sp. nov.

*Pheidole bemarivoensis* sp. nov.

*Pheidole nemoralis* complex

*Pheidole nemoralis* Forel, 1892

*Pheidole ala* sp. nov.

***Pheidole petax* group**

*Pheidole petax* complex

*Pheidole petax* Forel, 1895 stat. nov.

*Pheidole mavesatra* complex

*Pheidole mavesatra* sp. nov.

*Pheidole goavana* sp. nov.

*Pheidole ankerana* complex

*Pheidole ankerana* sp. nov.

*Pheidole vatovavensis* sp. nov.

*Pheidole boribora* complex

*Pheidole boribora* sp. nov.

*Pheidole miramila* sp. nov.

*Pheidole brevipilosa* complex

*Pheidole brevipilosa* sp. nov.

*Pheidole glabra* sp. nov.

**Figure 1.**
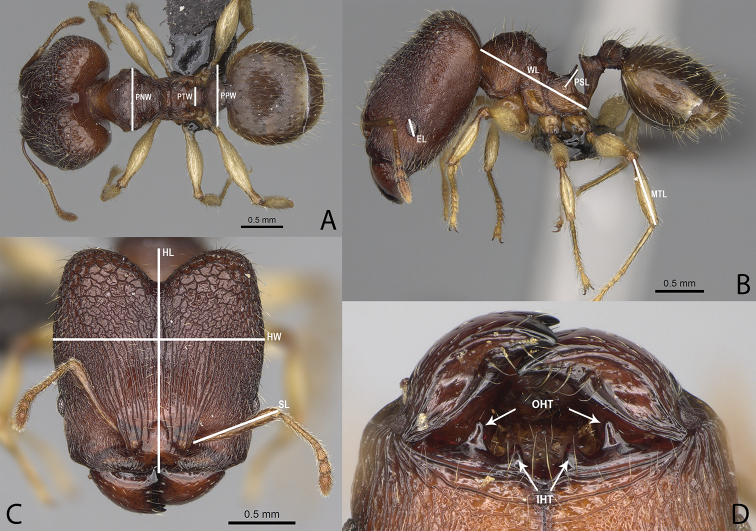
*Pheidole moramanaensis* sp. nov., illustrations of measurements (**A–C**) **A** dorsal view **B** profile **C** full-face view **D** inner hypostomal teeth (IHT) and outer hypostomal teeth (OHT).

## Taxonomy

### Preliminary key to *Pheidole* species groups of Madagascar (minor and major workers)

Note: This key is preliminary and ongoing revisions of all species groups may lead to further changes. Additionally, because of high infraspecific variability of minor workers we strongly encourage readers to use nest samples consisting of both major and minor workers while using the key. An asterisk (*) denotes groups revised in this monograph.

**Table:** 

1	Social parasite of *Pheidole* sp. Major workers. Absent. Minor workers. Yellow, with smooth body sculpture, promesonotal and metanotal grooves absent, promesonotum evenly arched and big eyes (Fig. 2A)	***Pheidole lucida* group**
–	Nonparasitic species. Both major and minor workers present. If body colouration of minor workers is yellow and body sculpture smooth them eyes never big (Fig. 2B, C)	**2**
2	Minor & major workers. Postpetiole in profile with conspicuous ventral convexity (sometimes subtriangular in major workers) (Fig. 3A, B). Minor workers. Head smooth, scape relatively short, surpassing the posterior head margin by two-fifths of its length, promesonotum in lateral view convex (Fig. 3A). Major workers. Head oval to cordate; occipital lobes shiny and smooth or with indistinct rugulae; frons with sparse and thick longitudinal rugulae and smooth to finely rugulose interspaces; inner hypostomal teeth indistinct or absent (Figs 3B, 4D)	***Pheidole megacephala* group**
–	Minor & major workers. Postpetiole in profile without conspicuous convexity, ventral process indistinct or, if present, acute and present only in major workers (Fig. 2A–C). Minor workers. Head at least partially foveolate or rugulose. If head entirely smooth then scape short and not surpassing the posterior head margin and promesonotum not evenly convex or scape long, surpassing the posterior head margin by at least one-third of its length and promesonotum of different shape (Fig. 2A–C). Major workers. Head shape different; if head oval or cordate then at least one of the following characters present: occipital lobes never smooth, inner hypostomal teeth well developed, antennal scrobes present, frons with sculpture other than sparse thick longitudinal rugae (Figs 3E, F, 4C, E, F)	**3**
3	Minor & major workers. Antennal sockets deep; frontal lobes distinct and lobe-like (Fig. 4A, B). Major workers. Outer hypostomal teeth absent (Fig. 4C)	*Pheidole diakritos* group*
–	Minor & major workers. Antennal sockets shallow; frontal lobes absent or indistinct (Fig. 3D–F). Major workers. Outer hypostomal teeth present (Fig. 4D–F)	**4**
4	Major & minor workers. Relatively large species, propodeal spines moderately to very long, thin and acute. Major workers. Inner and outer hypostomal teeth closely spaced and often connected by concavity (Fig. 4E). Minor workers. At least genae and frons with smooth notches (except *P. maizina*) (Fig. 5C, D, G, H)	**5**
–	Major & minor workers. Smaller species, propodeal spines short to moderately long, with wide or narrow base (Figs 7H, J, 9A–F, 10A–F). Major workers. Inner and outer teeth not closely spaced and if closely spaced, then propodeal spines shorter. Minor workers. Head foveolate, if genae and frons with smooth notches then propodeal spines short (Fig. 9A–F)	**6**
5	Major workers. Head, in full-face view, trapezoid, widening posteriorly; head sculpture fine, occipital lobes smooth or with indistinct microsculpture; promesonotum short, low, and evenly convex (Fig. 5A, B). Minor workers. Scape, when arranged along the anteroposterior axis of the head, surpassing posterior head margin by one third or more than half of its length; promesonotum low, long, and slightly convex; petiole with long and thin peduncle (Fig. 5C, D)	*Pheidole longispinosa* group*
–	Major workers. Head, in full-face view, rectangular; head sculpture strong rugoreticulate, sometimes weakens posteriorly; promesonotum short, angular, and relatively low to high (Fig. 5E, F). Minor workers. Scape, when arranged along the anteroposterior axis of the head, surpassing posterior head margin by one- to two-fifths of its length; promesonotum high, short and convex; petiole with shorter and thicker peduncle (Fig. 5G, H)	*Pheidole ensifera* group*
6	Major workers. Head in full-face view rectangular and longer than wide; in lateral view sub-oval to sub-rectangular; occipital lobes always with arcuate and/or transverse rugae; genae at least with a smooth notch (Fig. 6A, B). Minor workers. Head foveolate with at least smooth notches on genae; scape short, reaching the posterior margin of head or surpassing it by one- to two-fifths of its length; promesonotum low, convex, short; propodeal spines small and triangular; mesosoma predominately foveolate but always with smooth notches (Fig. 6E)	*Pheidole makaensis* group*
–	Major workers. Head in full-face view not elongated; if elongated then occipital lobes lacking arcuate or transverse rugae (Figs 6C, D, 7A–D). Minor workers. Head smooth or entirely foveolate; if head foveolate with smooth notches on genae then at least one of the following characters present: scape longer, surpassing the posterior margin of head by at least one-third of its length, promesonotum flat and long, posterior mesonotum steep, propodeal spines minute or relatively long, mesosoma predominately smooth (Fig. 8G–I)	**7**
7	Major workers. Head, in full-face view, elongate without arcuate or transverse rugae on occipital lobes or head oval; frons and lateral sides of head with thick longitudinal rugae; occipital lobes with irregular rugulae and sculpture weakening posteriorly; promesonotum high and arched; propodeal spines short with wide base (Fig. 6C, D, G). Minor workers. Head never foveolate, at least frons, genae, and malar area smooth and shiny smooth, sometimes lateral sides of frons with short, indistinct, longitudinal rugulae; scape short, when laid back, reaching posterior head margin or surpassing the posterior head margin by one-fifth of its length; mesosoma almost entirely smooth; body yellow (Fig. 6F)	*Pheidole lutea* group*
–	Major workers. Head not oval or elongate, if oval at least one of the following characters present: frons or lateral sides of head with thick irregular rugae, occipital lobes smooth, promesonotum low, propodeal spines relatively long (Fig. 7A–D). Minor workers. Head at least partly foveolate, if foveolae absent then at least one of the following characters present: scape longer, when laid back, surpassing the posterior head margin by at least two-fifths of its length, mesosoma at least partially foveolate, promesonotal groove present and deep, body not yellow (Fig. 8A, B, D–F)	**8**
8	Major workers. Head, in full-face view, sub-oval to cordate; frons and lateral sides of head predominately with irregular, thick rugae, if frons with longitudinal rugae then antennal scrobes present and well delimited *or* head in full face view square and in lateral view oval to sub-oval, with thick and sparse longitudinal rugae present on frons and lateral sides of head, promesonotum short, steep and high without promesonotal groove (Fig. 7A–F). Minor workers. Promesonotal groove absent or indistinct. Head and mesosoma predominately foveolate; promesonotum, in lateral view, low, long and convex with indistinct to moderately long propodeal spines (Fig. 8C), if posterior mesonotum steep then propodeal spines long (Fig. 8G); if head and mesosoma predominately smooth, promesonotum short and arched and propodeal spines minute to small (Fig. 8D)	**9**
–	Major workers. Head, in full-face view square to sub-rectangular, sometimes with lateral sides slightly convex; frons and lateral sides of had predominately with longitudinal rugae, promesonotum never steep or high, predominately low and arched (Fig. 7G–L). Minor workers. Promesonotal groove present and distinct (Fig. 8A, B, E, F), if absent or indistinct then head and mesosoma predominately foveolate; promesonotum, in lateral view, low, short to long with posterior mesonotum steep and propodeal spines minute to small (Fig. 8H, I)	**10**
9	Major workers. Head, in full-face view, sub-oval to cordate; frons with longitudinal rugae and antennal scrobes present and well delimited (Fig. 7A, B). Minor workers. Promesonotal groove absent; head and mesosoma predominately foveolate; promesonotum, in lateral view, box-like or convex with posterior mesonotum steep; propodeal spines short to long (Fig. 8G, J)	*Pheidole ferruginea* group*
–	Major workers. Head, in full-face view, sub-oval; frons and lateral sides of had predominately with irregular, thick rugae; antennal scrobes absent or poorly delimited *or* head in full face view square and in lateral view oval to sub-oval, with thick and sparse longitudinal rugae present on frons and lateral sides of head (Fig. 7C–F). Minor workers. Promesonotal groove absent or indistinct. Head and mesosoma predominately foveolate; promesonotum, in lateral view, low, long and convex with indistinct to short propodeal spines *or* head and mesosoma predominately smooth, promesonotum short and arched and propodeal spines minute to small (Fig. 8C, D)	***Pheidole sikorae* group**
10	Major workers. Head predominately with fine, sparse longitudinal rugae, sometimes fading posteriorly or head in lateral view oval to sub-oval with thick rugae, distinctly irregular on lateral sides of head with smooth to indistinctly foveolate interspaces; antennal scrobes absent or indistinct; promesonotum low; promesonotal groove present deep or indistinct (Fig. 7G–J). Minor workers. Head elongate, the posterior region of the head elongated into short to long neck, if neck absent then promesonotum, in lateral view, relatively long to long, low and convex and promesonotal groove present (Fig. 8A, E, F, K, L)	***Pheidole bessonii* group**
–	Major workers. Head predominately with fine, sparse longitudinal rugae limited to frons, occipital lobes with irregular rugulae and promesonotal groove absent; if occipital lobes with fine longitudinal rugae then antennal scrobes present and distinct, promesonotum low and short with deep promesonotal groove (Fig. 7K, L). Minor workers. Head not elongated; promesonotal groove absent, if present then promesonotum, in lateral view, short, relatively high and with steep posterior mesonotum (Fig. 8B, H, I)	**11**
11	Major workers. Promesonotal process well developed; promesonotum low and short; antennal scrobes distinct (Fig. 7K, L). Minor workers. Head and mesosoma predominately smooth and shiny; promesonotal groove present (Fig. 8B)	***Pheidole fervens* group**
–	Major workers. Promesonotal process absent or weakly developed; promesonotum moderately high to high; antennal scrobes absent or present but not delimited by carinulae (Figs 10A–F, 11A–D). Minor workers. Head and mesosoma predominately foveolae, sometimes with smooth notches; promesonotal groove absent (Fig. 8H, I)	**12**
12	Major & minor workers. Mesonotal spines present (sometimes major workers with teeth-like spines), propodeal spines long (Figs 9A, 10A)	*Pheidole annemariae* group*
–	Major & minor workers. Mesonotal spines absent (sometimes major workers with bulge-like process), propodeal spines short to moderately long (Figs 9B–F, 10B–F)	**13**
13	Major workers. Small body size (WL < 0.9 mm); head, in lateral view, sub-rectangular; ventral and dorsal faces relatively flat; antennal scrobes, when present, indistinctly to distinctly delimited (Figs 10B,C, 11A, B). Minor workers. Small body size (WL < 0.6mm); body yellow and foveolate with no additional sculpture (except *P. lamperos* and *P. zavamanira*); genae always smooth; propodeal spines minute to small. Minor workers are indistinguishable from those of the *nemoralis* group (Fig. 9B, C)	*Pheidole masoala* group*
–	Major workers. Body bigger (WL > 1.0 mm), if WL < 0.9 then head in lateral view, sub-rectangular to sub-oval, with ventral and dorsal faces convex and antennal scrobes not delimited (Figs 10D–F, 11C–E). Minor workers. Body bigger (WL > 0.65mm), if WL < 0.6 then body yellow or dark brown; head and mesosoma entirely foveolate or with distinct smooth notches; sometimes head and/or mesosoma with additional rugae. Note: Minor workers of the *masoala* group are indistinguishable from those of the *nemoralis* group (Fig. 9D–F)	**14**
14	Major workers. Head in full face view rectangular, slightly widening posteriorly, in lateral view sub-rectangular with ventral and dorsal faces finely convex (Figs 10F, 11E). Minor workers. Head foveolate, with thick, sparse, and longitudinal rugae on frons and sparse, irregular to arcuate, thick rugae on vertex; mesosoma foveolate or foveolate with additional thick and irregular rugae; body yellow to brown (Fig. 9F)	*Pheidole curvistriata* group*
–	Major workers. Head in full face view square to sub-rectangular, never widening posteriorly, in lateral view sub-oval or sob-rectangular with ventral and dorsal faces finely convex (Figs 10D, E, 11C, D). Minor workers. Head and mesosoma foveolate with no additional sculpture, if additional rugae present then body never yellow (except *Pheidole ankerana*) (Fig. 9D, E)	**15**
15	Major workers. Body size small (WL < 0.9 mm); head in full-face view square or subrectangular with anterior and posterior sides of eyes slightly convex, in lateral view sub-oval or sub-rectangular; body bright brown to dark brown (Figs 10E, 11D). Minor workers. Small body size (WL < 0.6mm); head foveolate with no additional rugae; mesosoma entirely foveolate or foveolate with smooth notches on its lateral sides; body yellow (Fig. 9E)	*Pheidole nemoralis* group*
–	Major workers. Body size bigger (WL > 1.1 mm); if WL < 0.9 mm then head, in lateral view not sub-oval, if sub-rectangular then in full-face view, square with anterior and posterior sides of eyes relatively straight and body dark brown to black (Figs 10D, 11C). Minor workers. Body larger (WL > 0.6 mm); if WL < 0.6 mm then head foveolate with additional rugae; mesosoma foveolate with smooth notches and additional indistinct rugae; body dark brown (Fig. 9D)	*Pheidole petax* group*

**Figure 2.**
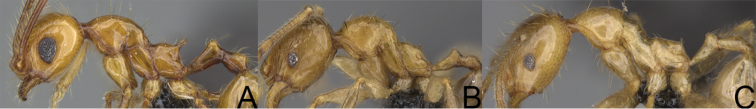
Minor worker, profile. *Pheidole oculata* Forel (**A**), *Pheidole lutea* sp. nov. (**B**), *Pheidole madecassa* (**C**).

**Figure 3.**
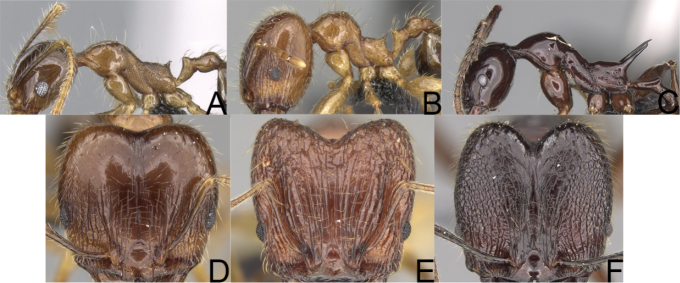
*Pheidole megacephala* (Fabricius), minor worker, profile (**A**) (Shannon Hartman). Major worker, profile (**B**), head (**D**). *Pheidole longispinosa* Forel minor worker, profile (**C**). *Pheidole ferruginea* sp. nov. major worker, head (**E**). Pheidole cf. sikorae major worker, head (**F**).

**Figure 4.**
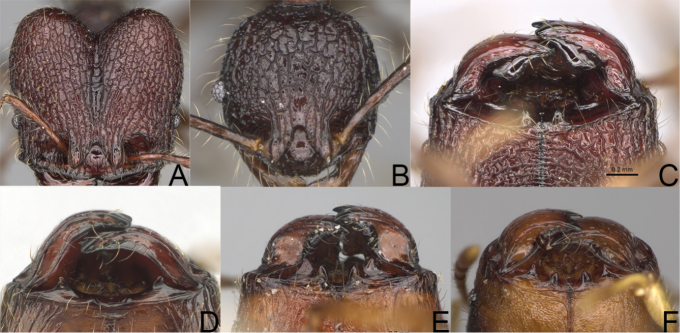
*Pheidole diakritos* sp. nov. minor worker, head (**B**). Major worker, head (**A**), hypostomal teeth (**C**). *Pheidole megacephala* (Fabricius) major worker, hypostomal teeth (**D**). *Pheidole ensifera* Forel major worker, hypostomal teeth (**E**). *Pheidole nemoralis* Forel major worker, hypostomal teeth (**F**).

**Figure 5.**
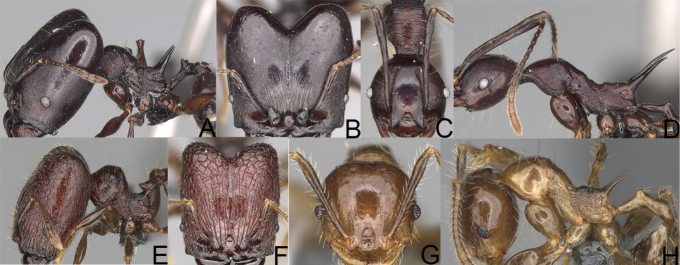
*Pheidole praegrandis* sp. nov. major worker, head (**B**), profile (**A**). Minor worker, head (**C**), profile (**D**). *Pheidole ocypodea* sp. nov. major worker, head (**F**), profile (**E**). Minor worker, head (**G**), profile (**H**).

**Figure 6.**
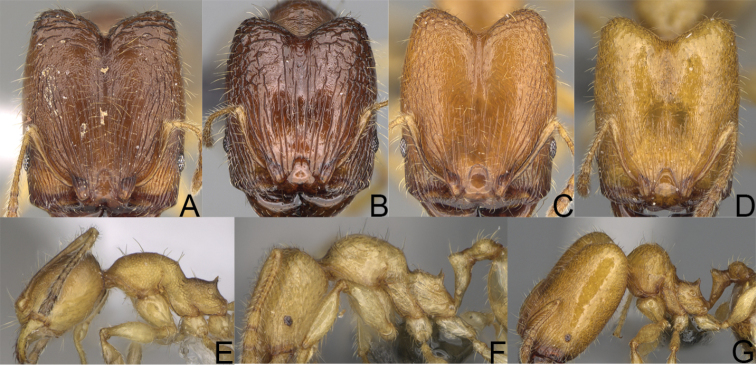
*Pheidole fitarata* sp. nov. major worker, head (**A**). Minor worker, profile (**E**). *Pheidole makaensis* sp. nov. major worker, head (**B**). *Pheidole navoatrensis* sp. nov. major worker, head (**C**). *Pheidole typhlos* sp. nov. major worker, head (**D**), profile (**G**). Minor worker, profile (**F**).

**Figure 7.**
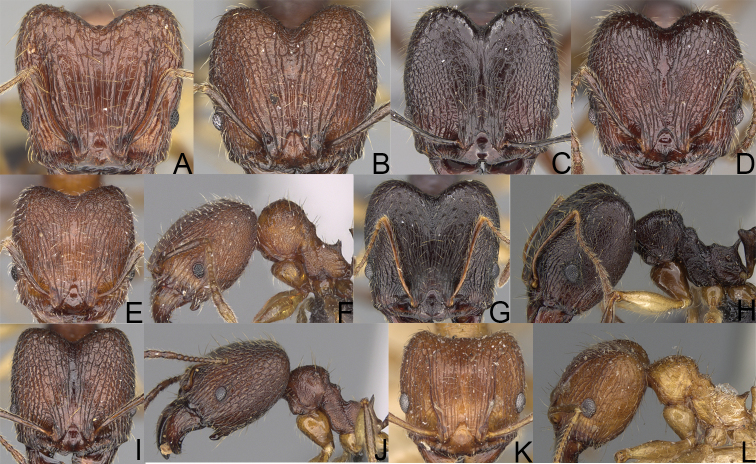
*Pheidole ferruginea* sp. nov. major worker, head (**A**). *Pheidole manantenensis* sp. nov. major worker, head (**B**). Pheidole cf. sikorae major worker, head (**C**). *Pheidole veteratrix* Forel major worker, head (**D**). Pheidole cf. veteratrix major worker, head (**E**), profile (**F**). *Pheidole bessonii* Forel major worker, head (**G**), profile (**H**). Pheidole cf. bessonii major worker, head (**I**), profile (**J**). *Pheidole indica* Mayr major worker, head (**K**), profile (**L**).

**Figure 8.**
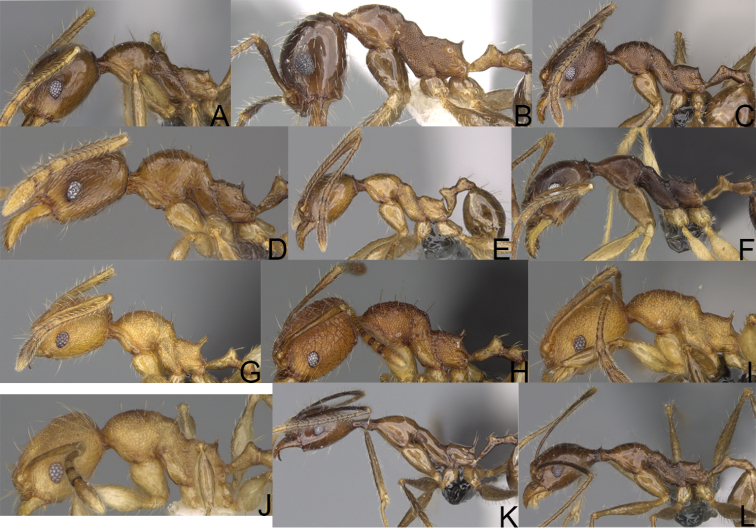
Pheidole cf. madecassa minor worker, profile (**A**). *Pheidole indica* Mayr minor worker, profile (**B**). *Pheidole veteratrix* Forel minor worker, profile (**C**). Pheidole cf. veteratrix minor worker, profile (**D**). Pheidole cf. bessonii minor worker, profile (**E**). *Pheidole bessonii* Forel minor worker, profile (**F**). *Pheidole vahamarensis* sp. nov. minor worker, profile (**G**). *Pheidole curvistriata* sp. nov. minor worker, profile (**H**). *Pheidole mavesatra* sp. nov. minor worker, profile (**I**). *Pheidole rugocephala* sp. nov. minor worker, profile (**J**). *Pheidole grallatrix* Forel minor worker, profile (**K**). Pheidole cf. grallatrix minor worker, profile (**L**).

**Figure 9.**
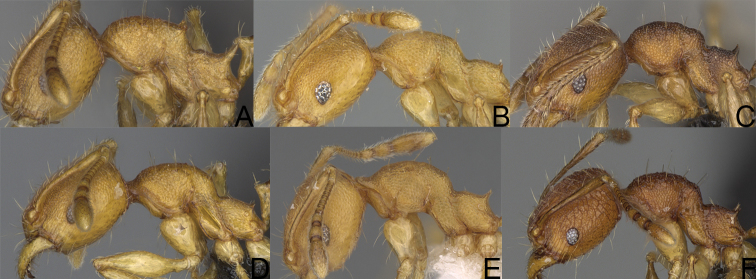
Minor worker, profile. *Pheidole annemariae* Forel (**A**). *Pheidole masoala* sp. nov. (**B**). Pheidole*zavamanira* sp. nov. (**C**). *Pheidole petax* Forel (**D**). *Pheidole bemarahaensis* sp. nov. (**E**). *Pheidole curvistriata* sp. nov. (**F**).

**Figure 10.**
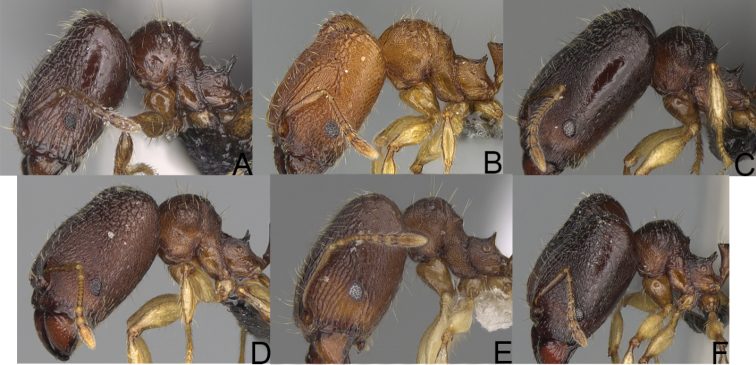
Major worker, profile. *Pheidole annemariae* Forel (**A**). *Pheidole masoala* sp. nov. (**B**). Pheidole*zavamanira* sp. nov. (**C**). *Pheidole petax* Forel (**D**). *Pheidole bemarahaensis* sp. nov. (**E**). *Pheidole curvistriata* sp. nov. (**F**).

**Figure 11.**
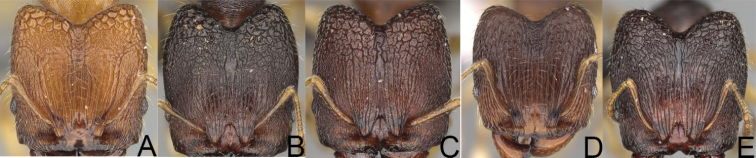
Major worker, head. *Pheidole masoala* sp. nov. (**A**). Pheidole*zavamanira* sp. nov. (**B**). *Pheidole petax* Forel (**C**). *Pheidole bemarahaensis* sp. nov. (**D**). *Pheidole curvistriata* sp. nov. (**E**).

### Species accounts

Repetitive characters occurring in the majority of species have been omitted. Unless stated otherwise, the following descriptions apply to all species treated here:

**Major workers.** Antennal sockets shallow; frontal lobes absent; head in full-face view with distinct median concavity; antenna 12-segmented, with 3-segmented club; masticatory margin of mandible with large, stout apical and preapical teeth, followed by a long diastema and then a short and crenulate tooth just before the rounded basal angle; outer surface of mandible mostly smooth and shining, sometimes with weak and sparse foveolae; antennal scrobes present; promesonotum strongly convex, well above the level of propodeum; postpetiole short with slightly convex dorsum; ventral process absent.

**Minor workers.** Antennal sockets shallow; frontal lobes absent; head in full-face view oval, posterior and anterior of eyes convex; antenna 12-segmented, with 3-segmented club; humeral area not developed; clypeus smooth and shiny; its anterior margin regularly convex; promesonotum well above the level of propodeum; petiole smooth; petiole with node moderately low, triangular and small postpetiole smooth; gaster smooth and shiny.

#### Revision of the *Pheidole diakritos* group

**Diagnosis. *Major worker*.** Head, in full-face view rectangular; in lateral view sub-rectangular; ventral and dorsal faces relatively flat; sides of the head with dense, relatively long, erect pilosity; antennal sockets deep, smooth or with a few rugae; frontal lobes distinct and lobe-like; head shiny, with sparse, thick, irregular rugae, interspaces smooth; inner hypostomal teeth distinct, closely spaced, triangular, with rounded apex and base slightly wider than apex; outer hypostomal teeth absent. Promesonotum short, angular and low; promesonotal groove absent; metanotal groove indistinct; propodeal spines long; mesosoma with thick, dense rugoreticulation; gaster shagreened on the whole surface. ***Minor worker*.** Head shiny, with sparse, thick, irregular rugae, interspaces smooth or with rugulae; scape, when laid back, surpassing posterior head margin by two-fifths of its length; promesonotum, in lateral view, convex; promesonotal groove present; metanotal groove present; humeral area with short triangular tubercles; pronotum and mesonotum with thick, dense rugoreticulation, sculpture slightly weakening on dorsum; katepisternum, anepisternum, and propodeum foveolate.

**Comments.** This species-group can be easily distinguished from others by presence of deep antennal sockets and distinct lobe-like frontal lobes in both, major and minor workers. Major workers also can be distinguished by absence of outer hypostomal teeth, and minor workers by presence of short triangular tubercles on the humeral area.

This group contains only one species: *Pheidole diakritos* sp. nov. distributed across the evergreen rainforest biome.

##### Pheidole diakritos sp. nov.

Taxon classificationAnimaliaHymenopteraFormicidae

http://zoobank.org/C5CE9E88-6E38-4A9F-BFA3-75D539949159

**Figure 12.**
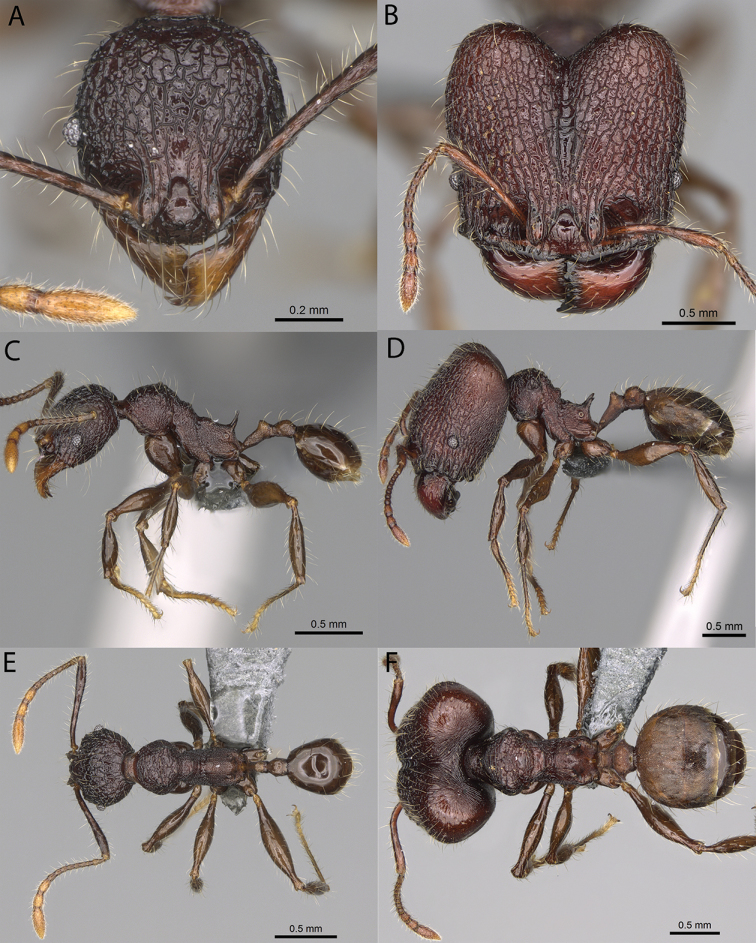
*Pheidole diakritos* sp. nov., full-face view (**A**), profile (**C**), and dorsal view (**E**) of paratype minor worker (CASENT0275480) and full-face view (**B**), profile (**D**), and dorsal view (**F**) of holotype major worker (CASENT0275436).

Figs 12A–F , 84M , 86M

###### Type material.

***Holotype*.** Madagascar. •1 major worker; Toamasina; Ankerana; -18.40829, 48.82107; alt. 750 m; 21 Jan 2012; B.L. Fisher et al. leg.; CASENT0275436 (CASC). ***Paratype*.** Madagascar. •1 w.; Toamasina; Ankerana; -18.4061, 48.82029; alt. 725 m; 16 Jan 2012; B.L. Fisher et al. leg.; BLF27931, CASENT0275480 (CASC).

**Other material.** Madagascar. –***Fianarantsoa***: •5w.; Forêt de Vevembe, 66.6 km 293° Farafangana; -22.791, 47.18183; alt. 600 m; 23 Apr 2006; B.L. Fisher et al. leg.; CASENT0108005, CASENT0108016, CASENT0108021, CASENT0108022, CASENT0108028 (CASC). –***Toamasina***: •2w.; Réserve Spéciale Ambatovaky, Sandrangato River; -16.77274, 49.26551; alt. 450 m; 20 Feb 2010; B.L. Fisher et al. leg.; CASENT0164424, CASENT0164471 (CASC). •3w., 2s.; Réserve Spéciale Ambatovaky, Sandrangato River; -16.7633, 49.26692; alt. 520 m; 22 Feb 2010; B.L. Fisher et al. leg.; CASENT0163714, CASENT0163724, CASENT0163831, CASENT0163832, CASENT0163976 (CASC). •1w.; Réserve Spéciale Ambatovaky, Sandrangato River; -16.81739, 49.29402; alt. 360 m; 25 Feb 2010; B.L. Fisher et al. leg.; CASENT0164250 (CASC). •2w.; Ankerana; -18.4061, 48.82029; alt. 725 m; 16 Jan 2012; B.L. Fisher et al. leg.; CASENT0275346, CASENT0275461 (CASC).

###### Diagnosis.

***Major workers*.** Large species: HL: 1.67–1.91 (1.75), HW: 1.66–1.86 (1.71), WL: 1.23–1.3 (1.27); head in full-face view rectangular; in lateral view sub-rectangular with visible inner hypostomal teeth; antennal scrobes absent; sides of the head with dense, relatively long, erect pilosity; antennal sockets deep; frontal lobes distinct and lobe-like; inner hypostomal teeth distinct, closely spaced, triangular, with rounded apex and base slightly wider than apex; outer hypostomal teeth absent. ***Minor workers*.** Head shiny, with sparse, thick, irregular rugae, interspaces smooth or rugulose; frontal lobes present; antennal sockets deep; propodeal spines long and thin (PSL: 0.17–0.21 (0.19)).

###### Description.

**Major workers.** Measurements (*N* = 5): HL: 1.67–1.91 (1.75); HW: 1.66–1.86 (1.71); SL: 0.8–0.87 (0.85); EL: 0.15–0.16 (0.155); WL: 1.23–1.3 (1.27); PSL: 0.27–0.3 (0.28); MTL: 0.78–0.87 (0.81); PNW: 0.65–0.74 (0.69); PTW: 0.16–0.2 (0.17); PPW: 0.42–0.54 (0.46); CI: 97.1–99.4 (98.3); SI: 46.7–51.6 (48.9); PSLI: 14.8–17.7 (16.3); PPI: 36.1–39.8 (37.7); PNI: 38.9–42.8 (40.3); MTI: 46.9–47.8 (47.2). ***Head*.** In full-face view rectangular, anterior of eyes relatively straight, posterior of eyes slightly convex (Fig. 12B). In lateral view sub-rectangular; ventral and dorsal faces relatively flat; inner hypostomal teeth visible. Sides of the head with dense, relatively long, erect pilosity; whole head with moderately dense, long, suberect to erect pilosity. Antennal scrobes absent. Antennal sockets deep, smooth, or with a few rugae. Frontal lobes distinct and lobe-like. Occipital lobes with thick, sparse rugae; sculpture slightly weakening posteriorly. Whole head shiny, with sparse, thick, irregular rugae, interspaces smooth. Clypeus shiny, with a few longitudinal rugae; median notch present, wide, and shallow. Scape, when laid back, just reaching the midlength of head; pilosity decumbent to erect (Fig. 12B, D). Inner hypostomal teeth distinct, closely spaced, triangular, with rounded apex and base slightly wider than apex; outer hypostomal teeth absent (Fig. 84M). ***Mesosoma*.** In lateral view, promesonotum short, angular, and low, posterior mesonotum convex, with low tubercle-like projection; promesonotal groove absent; metanotal groove indistinct; propodeal spines long, massive basally, with acute apex; humeral area with short, triangular tubercles (Fig. 12D). Surface shiny, with thick, dense rugoreticulation, sculpture slightly weakening on dorsum. Pilosity moderately dense, long and erect (Fig. 12D, F). ***Petiole*.** Shiny, foveolate; peduncle relatively long, without horizontal lobes on its basal part; node, low and narrow, with flat apex, in rear view node dorsoventrally depressed; pilosity moderately dense and erect (Fig. 12D, F). ***Postpetiole*.** Foveolate; in dorsal view sides with short, acute, and triangular projections; pilosity long, moderately sparse and erect (Fig. 12D, F). ***Gaster*.** Shagreened on the whole surface; pilosity moderately dense, very long and erect (Fig. 12D, F). ***Colour*.** Unicolourous, dark brown (Fig. 12D, F).

**Minor workers.** Measurements (*N* = 10): HL: 0.68–0.78 (0.73); HW: 0.59–0.68 (0.64); SL: 0.73–0.83 (0.81); EL: 0.07–0.12 (0.1); WL: 0.89–1.04 (0.98); PSL: 0.17–0.21 (0.19); MTL: 0.56–0.66 (0.62); PNW: 0.44–0.52 (0.49); PTW: 0.08–0.13 (0.1); PPW: 0.12–0.19 (0.16); CI: 85.3–90.2 (87.8); SI: 121.9–129.7 (125.3); PSLI: 25.1–28.7 (26.4); PPI: 59.1–69.3 (64.6); PNI: 73.9–78.4 (76.0); MTI: 92.6–103.0 (97.1). ***Head*.** Occipital margin indistinctly concave; occipital carina indistinct, weakly developed (Fig. 12A). Pilosity sparse, long, suberect to erect; antennal sockets deep, smooth or with a few rugae; frontal lobes distinct and lobe-like. Whole head shiny, with sparse, thick, irregular rugae, interspaces smooth or with rugulae. Clypeus rugulose and shiny; median longitudinal carina absent; two lateral longitudinal carinae absent. Scape, when laid back, surpassing posterior head margin by two-fifths of its length; pilosity suberect to erect (Fig. 12A, C). ***Mesosoma*.** In lateral view, promesonotum convex; promesonotal groove present; metanotal groove present; humeral area with short triangular tubercles; propodeal spines moderately long, with base almost as wide as apex, apex acute (Fig. 12C). Pronotum and mesonotum with thick, dense rugoreticulation, sculpture slightly weakening on dorsum; katepisternum, anepisternum, and propodeum foveolate. Pilosity sparse, long, and erect (Fig. 12C, E). ***Petiole*.** Shiny; peduncle foveolate, moderately long and thin; node smooth, low, bulge-like; with few long, erect setae (Fig. 12C, E). ***Postpetiole*.** Sometimes partially foveolate; moderately short, low and slightly convex; with few long, erect setae at the anterior edge (Fig. 12C, E). ***Gaster*.** Pilosity sparse and erect (Fig. 12C, E). ***Colour*.** Brown to dark brown, legs brighter (Fig. 12C, E).

**Etymology.** Greek for distinct [d?a???t??], in reference to its being the only known species with distinct frontal lobes and deep antennal sockets.

**Biology.** The species was collected at elevation between 360–865 m, in rainforest, and in montane rainforest. Nesting preferences are unknown.

#### Revision of the *Pheidole lutea* group

**Diagnosis. *Major workers*.** Head, in full-face view, oval or elongate, in lateral view sub-oval, ventral and dorsal faces convex or relatively flat; dorsal face not depressed posteriorly; antennal scrobes absent or very indistinct, occipital lobes smooth or with thick, sparse, irregular rugae, sculpture weakening posteriorly; frons with thick rugae, interspaces smooth to rugo-foveolate; genae smooth or with thick, sparse, irregular rugae; promesonotum relatively low to high, and arched; propodeal spines absent, weakly developed, and lobe-like or small and short, triangular; mesosoma surface with fine, sparse to moderately sparse rugoreticulation (dorsal surface with weaker sculpture) or smooth; body yellow to occasionally bright brown. ***Minor workers*.** At least frons, genae, and malar area smooth and shiny, sometimes lateral sides of frons with short, indistinct, longitudinal rugulae; scape short, when laid back, reaching posterior head margin or surpassing the posterior head margin by one-fifth of its length; promesonotum, in lateral view, convex or box-like; mesosoma almost entirely smooth; body yellow.

**Comments.** Major workers of this group can be easily distinguished from others based on elongate to oval head capsule in full-face view and sub-oval in lateral view, head sculpture weakening posteriorly and reduced sculpture of mesosoma, strongly reduced and sometimes absent to small propodeal spines, and bright body colouration. Minor workers can be separated from other species based on smooth and shiny head and mesosoma sculpture, short scape, and yellow body colouration.

The group is divided into two complexes. The *P. lutea* complex contains three species: *P. lutea* sp. nov., *P. ranohirensis* sp. nov., and *P. voasara* sp. nov., all distributed across dry deciduous forest biome and Sambirano rainforest biome. The *P. navoatrensis* complex also contains three species: *P. navoatrensis* sp. nov., *P. parviocula* sp. nov., and *P. typhlos* sp. nov. *Pheidole navoatrensis* sp. nov. and *P. parviocula* are known from central highlands, and *P. navoatrensis* additionally expands its range to the dry deciduous biome. *Pheidole typhlos* is known only from its type locality, the Galoko massif.

##### Key to the *Pheidole lutea* group

**Table:** 

1	Major workers. Head, in full-face view, elongate; occipital lobes smooth, sometimes with indistinct, very sparse, longitudinal to irregular rugoreticulate; genae smooth (Fig. 13). Minor workers. Head vertex smooth and never with few arcuate, interrupted rugae; scape, when laid back, reaching posterior head margin (Fig. 13)	**2**
–	Major workers. Head, in full-face view, oval; occipital lobes and genae with thick, sparse, irregular rugae, interspaces with indistinct foveolae (Fig. 14). Minor workers. Vertex smooth and with few arcuate, interrupted rugae; scape, when laid back, surpassing the posterior head margin by one-fifth of its length (Fig. 14)	**3**
2	Major workers. Genae and antennal scrobes never foveolate, outer hypostomal teeth approximately as high as inner hypostomal teeth, with very wide base and upper half thin, rectangular, and pointed outward, anepisternum, katepisternum, and mesosoma smooth (Fig. 13A, F, I). Minor workers. Promesonotum in later view evenly arched, high, and short (Fig. 13D)	***P. lutea* sp. nov.**
–	Major workers. Genae and antennal scrobes foveolate, outer hypostomal teeth dentate, smaller and thinner than inner hypostomal teeth and never pointed outward; anepisternum, katepisternum, and mesosoma never entirely smooth (Fig. 13B, G, J). Minor workers. Promesonotum in later view low, short, slightly convex, and with relatively steep posterior declivity (Fig. 13E)	***P. ranohirensis* sp. nov.^1^**
3	Major workers. Eyes well-developed (EL > 0.1), posterior mesonotum never concave, outer hypostomal teeth weakly developed (Fig. 14A, D). Minor workers. Eyes well developed (EL > 0.07), promesonotum convex, and propodeal spines small, triangular (Fig. 14G)	***P. navoatrensis* sp. nov.**
–	Major workers. Eyes small and reduced (EL < 0.1), posterior mesonotum concave, outer hypostomal teeth well developed (Fig. 14B, C, E, F). Minor workers. Eyes small and reduced (EL < 0.06), promesonotum box-like, if convex then propodeal spines indistinct (Fig. 14H, I)	**4**
4	Major workers. Sides of head with sparse and short pilosity, inner hypostomal teeth distinct, triangular, propodeal spines short and triangular (Fig. 14C, F, K). Minor workers. Promesonotum box-like, propodeal spines distinct, short and triangular (Fig. 14I)	***P. typhlos* sp. nov.**
–	Major workers. Sides of head with dense and relatively long pilosity, inner hypostomal teeth indistinct, lobe-like, propodeal spines absent or weakly developed (Fig. 14B, E, J). Minor workers. Promesonotum convex, propodeal spines weakly developed, indistinct (Fig. 14H)	***P. parviocula* sp. nov.**

**Figure 13.**
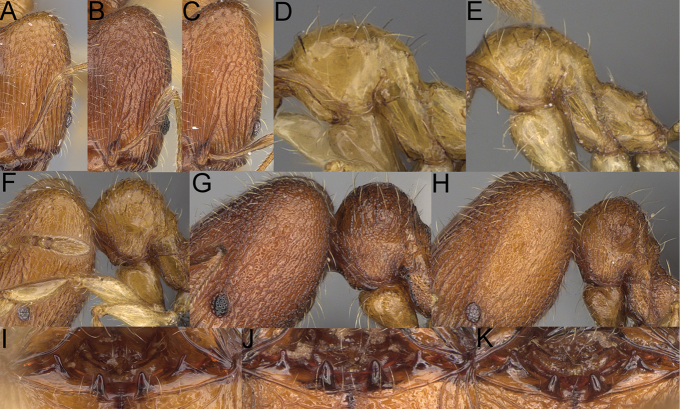
*Pheidole lutea* sp. nov., head of major worker (**A**), profile of major worker (**F**) and minor worker (**D**), and hypostomal teeth (**I**). *Pheidole ranohirensis* sp. nov., head of major worker (**B**), profile of major worker (**G**) and minor worker (**E**), and hypostomal teeth (**J**). *Pheidole voasara* sp. nov., head of major worker (**C**), profile of major worker (**H**), and hypostomal teeth (**K**).

**Figure 14.**
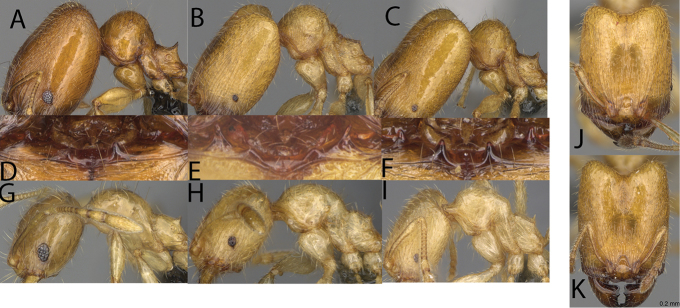
*Pheidole navoatrensis* sp. nov., profile of major worker (**A**), minor worker (**G**) and hypostomal teeth (**D**). *Pheidole typchlos*, head of major worker (**K**), profile of major worker (**C**), minor worker (**I**) and hypostomal teeth (**F**). *Pheidole parviocula* sp. nov., head of major worker (**J**), profile of major worker (**B**), minor worker (**H**) and hypostomal teeth (**E**).

#### Revision of the *Pheidole lutea* complex

**Diagnosis. *Major workers*.** Head, in full-face view, oval; sides of the head with moderately dense to dense, long, erect pilosity; antennal scrobes indistinct and not delimited by carinulae; scrobe surface shiny, with sparse, thick, longitudinal to irregular rugae, interspaces smooth, indistinctly rugulose to foveolate; occipital lobes and genae with thick, sparse, irregular rugae, interspaces with indistinct foveolae, sculpture weakening posteriorly; frons with dense, thick, and longitudinal rugae, interspaces smooth to foveolate; promesonotum, in lateral view, short, angular, and relatively high; promesonotal and metanotal grooves absent; propodeal spines minute and indistinct to small, triangular; mesosoma with fine, sparse rugoreticulation and indistinct, sparse foveolae or foveolate with additional sparse and thick rugae on promesonotal dorsum and propodeum, sculpture slightly weakening on dorsum; anepisternum and mesosoma with indistinct sculpture or smooth; gaster smooth to finely shagreened; body orange to yellowish brown. ***Minor workers*.** Frons, genae, and malar area smooth and shiny, vertex smooth and with few arcuate, interrupted rugae; scape, when laid back, surpassing the posterior head margin by one-fifth of its length; promesonotum moderately low to high, short, evenly arched; promesonotal and metanotal grooves absent; propodeal spines very small, indistinct, triangular; mesosoma smooth and shiny, only dorsum with few transverse, thick rugulae; body yellow.

**Comments.** Major workers of this complex can be easily distinguished based on a combination of the following characters: head, in full-face view, oval (but not elongate) and in lateral view sub-oval, lacking smooth patches and predominately covered with longitudinal rugae; minute to small propodeal spines; gaster never distinctly shagreened, and bright body colouration. Minor workers can be distinguished based on smooth sculpture of head and mesosoma, with few additional arcuate rugae on vertex and transverse rugulae on mesosoma, moderately short scape, and yellow body.

##### Pheidole lutea sp. nov.

Taxon classificationAnimaliaHymenopteraFormicidae

http://zoobank.org/F0BCD45A-F0B1-4275-B479-8910261BE825

**Figure 15.**
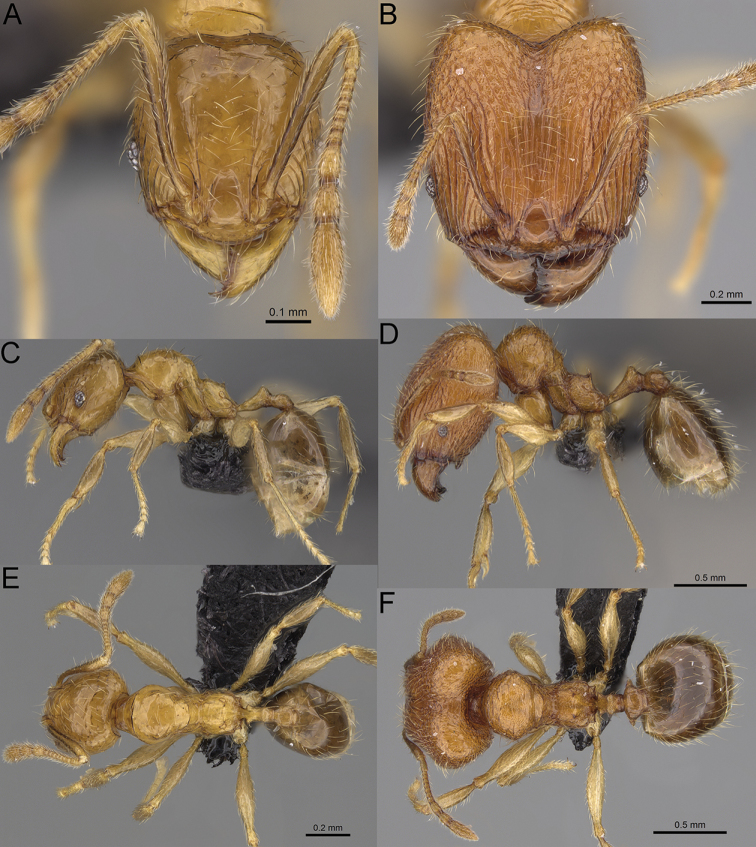
*Pheidole lutea* sp. nov., full-face view (**A**), profile (**C**), and dorsal view (**E**) of paratype minor worker (CASENT0485689) and full-face view (**B**), profile (**D**), and dorsal view (**F**) of holotype major worker (CASENT0485691).

Figs 15A–F , 84Z , 87D

###### Type material.

***Holotype*.** Madagascar. •1 major worker; Fianarantsoa; Parc National d’Isalo, 9.1 km 354°N Ranohira; -22.48167, 45.46167; alt. 725 m; 31 Jan 2003; B.L. Fisher et al. leg.; BLF07348, CASENT0485691 (CASC). ***Paratypes*.** Madagascar. •5w.; same data as for holotype; CASENT0485689, CASENT0485690, CASENT0872151–CASENT0872153 (CASC).

###### Diagnosis.

***Major workers*.** Head in full-face view oval, with anterior and posterior sides slightly convex; sides of the head with moderately dense, long, erect pilosity; occipital lobes and genae shiny, with thick, sparse, irregular rugae, interspaces with indistinct foveolae, sculpture weakening posteriorly; inner hypostomal teeth distinct, moderately high, closely spaced, triangular, with rounded apex and wide base; outer hypostomal approximately as high as inner hypostomal teeth, dentate, with very wide base, and upper half thin, rectangular with top pointed outward; mesosoma shiny, with fine, sparse rugoreticulation and indistinct, sparse foveolae, sculpture weakening on dorsum, anepisternum, katepisternum, and mesosoma smooth; gaster smooth; body orange to bright brown. ***Minor workers*.** Frons, genae, and malar area smooth and shiny; vertex smooth and shiny with few arcuate, interrupted rugae; promesonotum moderately high, short, evenly arched; propodeal spines very small, indistinct, triangular; mesosoma smooth and shiny, only dorsum with few transverse, thick rugulae.

###### Description.

**Major workers.** Measurements (*N* = 1): HL: 0.97; HW: 0.9; SL: 0.47; EL: 0.09; WL: 0.8; PSL: 0.1; MTL: 0.48; PNW: 0.48; PTW: 0.14; PPW: 0.34; CI: 92.5; SI: 52.6; PSLI: 10.7; PPI: 40.8; PNI: 53.1; MTI: 53.6. ***Head*.** In full-face view oval, with anterior and posterior sides slightly convex (Fig. 15B). In lateral view sub-oval; ventral and dorsal faces convex; dorsal face not depressed posteriorly; inner hypostomal teeth visible. Sides of the head with moderately dense, long, erect pilosity; whole head with dense, short, suberect to erect pilosity. Antennal scrobes indistinct and not delimited by carinulae, scrobe surface shiny, with sparse, thick, longitudinal rugae, interspaces smooth or indistinctly rugulose. Occipital lobes and genae shiny, with thick, sparse, irregular rugae, interspaces with indistinct foveolae, sculpture weakening posteriorly; frons and malar area with dense, thick and longitudinal rugae, interspaces smooth. Centre of clypeus smooth and shiny, lateral sides with longitudinal rugae; median notch present, wide, and indistinct; median longitudinal carina absent; lateral longitudinal carinae absent. Scape, when laid back, slightly exceeding the midlength of head; pilosity suberect to erect (Fig. 15B, D). Inner hypostomal teeth distinct, moderately high, closely spaced, triangular, with rounded apex and wide base; outer hypostomal approximately as high as inner hypostomal teeth, dentate, with very wide base, and upper half thin, rectangular with top pointed outward (Fig. 84Z). ***Mesosoma*.** In lateral view, promesonotum short, angular, and relatively high, posterior mesonotum relatively steep, tubercle-like projections absent or very indistinct; promesonotal groove absent; metanotal groove absent; propodeal spines minute, indistinct, triangular, with acute apex; humeral area laterally weakly produced (Fig. 15D). Surface shiny, with fine, sparse rugoreticulation and indistinct, sparse foveolae, sculpture weakening on dorsum, anepisternum, and katepisternum, mesosoma smooth. Pilosity moderately dense, long, and erect (Fig. 15D, F). ***Petiole*.** Shiny with fine, indistinct, and sparse foveolae; peduncle moderately short, with indistinct horizontal lobes on its basal part; node smooth, relatively low, triangular, with rounded apex, in rear view node straight; pilosity moderately sparse and erect (Fig. 15D, F). ***Postpetiole*.** Shiny, with fine, indistinct, and sparse foveolae; in dorsal view sides with short, acute, and triangular projections; pilosity long, moderately dense, and erect (Fig. 15D, F). ***Gaster*.** Smooth; pilosity dense, moderately long, and erect (Fig. 15D, F). ***Colour*.** Unicolourous, orange to bright brown; legs yellow; gaster brown (Fig. 15D, F).

**Minor workers.** Measurements (*N* = 4): HL: 0.46–0.48 (0.47); HW: 0.41–0.43 (0.42); SL: 0.39–0.43 (0.42); EL: 0.06–0.08 (0.07); WL: 0.51–0.55 (0.53); PSL: 0.04–0.06 (0.05); MTL: 0.31–0.32 (0.32); PNW: 0.28–0.29 (0.28); PTW: 0.06–0.07 (0.07); PPW: 0.1–0.11 (0.1); CI: 88.7–90.3 (89.7); SI: 95.2–104.3 (100.0); PSLI: 9.4–12.4 (10.7); PPI: 61.6–71.2 (66.8); PNI: 65.6–68.4 (67.2); MTI: 74.8–76.8 (75.5). ***Head*.** Occipital margin straight or indistinctly concave; occipital carina absent (Fig. 15A). Pilosity moderately dense, relatively short, erect. Frons, genae, and malar area smooth and shiny; vertex smooth and shiny with few arcuate, interrupted rugae; antennal sockets with few thick, curved outward rugae. Clypeus with median longitudinal carina absent; two lateral longitudinal carinae absent. Scape, when laid back, surpassing the posterior head margin by one-fifth of its length; pilosity suberect (Fig. 15A, C). ***Mesosoma*.** In lateral view, promesonotum moderately high, short, evenly arched; promesonotal groove absent; metanotal groove absent; propodeal spines very small, indistinct, triangular, apex acute (Fig. 15C). Sculpture smooth and shiny, only dorsum with few transverse, thick rugulae. Pilosity moderately sparse, moderately long, and erect (Fig. 15C, E). ***Petiole*.** Peduncle moderately short and thin with ventral face slightly convex; node low, triangular, and small; with few moderately long, erect setae (Fig. 15C, E). ***Postpetiole*.** Short, low and slightly convex; with few moderately long, erect setae (Fig. 15C, E). ***Gaster*.** With moderately sparse, erect pilosity (Fig. 15C, E). ***Colour*.** Unicoloured, yellow (Fig. 15C, E).

###### Etymology.

Latin for yellow, in reference to body colouration of major and minor workers.

###### Biology.

The species was collected at elevation 725 m, in gallery forest. Nest was located in rotten log.

###### Comments.

*Pheidole lutea* sp. nov. is most similar to *P. ranohirensis* sp. nov. and *P. voasara* sp. nov. ***Major workers*.***Pheidole lutea* sp. nov. differs from those both taxa by surface of genae and antennal scrobes never foveolate, outer hypostomal teeth approximately as high as inner hypostomal teeth, with very wide base, and upper half thin, rectangular with top pointed outward, and by smooth anepisternum, katepisternum, and mesosoma. ***Minor workers*.***Pheidole lutea* sp. nov. differs from *P. ranohirensis* sp. nov. in evenly arched, high and short promesonotum. Minor workers of *P. voasara* sp. nov. are unknown.

##### Pheidole ranohirensis sp. nov.

Taxon classificationAnimaliaHymenopteraFormicidae

http://zoobank.org/C2BF9E41-D942-455E-8C54-CF97CC35F5BA

**Figure 16.**
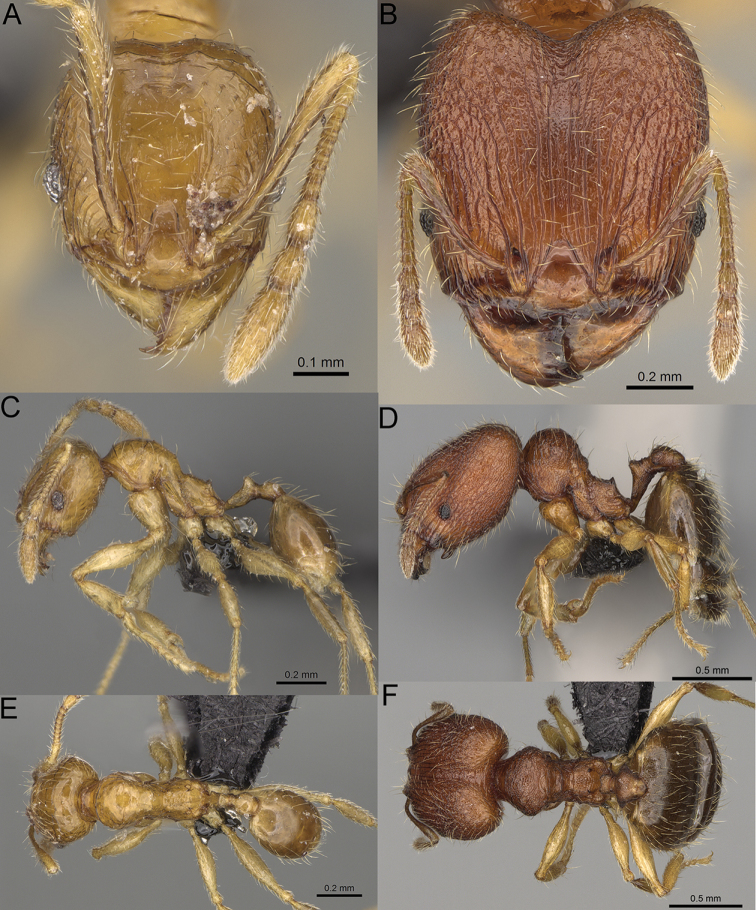
*Pheidole ranohirensis* sp. nov., full-face view (**A**), profile (**C**), and dorsal view (**E**) of paratype minor worker (CASENT0490856) and full-face view (**B**), profile (**D**), and dorsal view (**F**) of holotype major worker (CASENT0490851).

Figs 16A–F , 85T , 87D

###### Type material.

***Holotype*.** Madagascar. •1 major worker; Fianarantsoa; Parc National d’Isalo, Sahanafa River, 29.2 km 351°N Ranohira; -22.31333, 45.29167; alt. 500 m; 10 Feb 2003; B.L. Fisher et al. leg.; BLF07681, CASENT0490851, top specimen (CASC). ***Paratypes*.** Madagascar. •6w., 5s.; same data as for holotype; CASENT0490850, CASENT0490852–CASENT0490854, CASENT0490856, CASENT0872083, CASENT0872206–CASENT0872214 (CASC).

**Other material.** Madagascar. –***Antsiranana***: •1s.; Ampasindava, Forêt d’Ambilanivy, 3.9 km 181°S Ambaliha; -13.79861, 48.16167; alt. 600 m; 4 Mar 2001; Fisher et al. leg.; CASENT0464406 (CASC). –***Fianarantsoa***: •9w., 8s., 2q.; Parc National d’Isalo, Sahanafa River, 29.2 km 351°N Ranohira; -22.31333, 45.29167; alt. 500 m; 10 Feb 2003; Fisher et al. leg.; CASENT0031826, CASENT0490740, CASENT0490756, CASENT0490757, CASENT0490761, CASENT0490763, CASENT0490768, CASENT0490772, CASENT0490864, CASENT0490865 (CASC). –***Mahajanga***: •2s.; Forêt de Tsimembo, 11.0 km 346°NNW Soatana; -18.99528, 44.4435; alt. 50 m; 21 Nov 2001; Fisher et al. leg.; CASENT0483413, CASENT0483517 (CASC). •1s.; Parc National d’Ankarafantsika, Forêt de Tsimaloto, 18.3 km 46°NE de Tsaramandroso; -16.22806, 47.14361; alt. 135 m; 2 Apr 2001; Fisher et al. leg.; CASENT0432165 (CASC). •3w., 3s.; Parc National de Namoroka, 16.9 km 317°NW Vilanandro; -16.40667, 45.31; alt. 100 m; 12-Nov-2002; Fisher et al. leg.; CASENT0038806, CASENT0038833, CASENT0038875, CASENT0023575, CASENT0023577, CASENT0023583 (CASC). •1w., 1s.; Parc National Tsingy de Bemaraha, 10.6 km ESE 123° Antsalova; -18.70944, 44.71817; alt. 150 m; 16 Nov 2001; Fisher et al. leg.; CASENT0078424, CASENT0078429 (CASC). •1s.; Parc National Tsingy de Bemaraha, 3.4 km 93°E Bekopaka, Tombeau Vazimba; -19.14194, 44.828; alt. 50 m; 6 Nov 2001; Fisher et al. leg.; CASENT0477494 (CASC). •4w., 1s.; Réserve Spéciale de Bemarivo, 23.8 km 223°SW Besalampy; -16.925, 44.36833; alt. 30 m; 19 Nov 2002; Fisher et al. leg.; CASENT0022564, CASENT0022764, CASENT0022772, CASENT0022775, CASENT0022787 (CASC).

###### Diagnosis.

***Major workers*.** Head in full-face view oval, with anterior and posterior sides slightly convex; sides of the head with dense, long, erect pilosity; occipital lobes and genae shiny, with thick, sparse, irregular rugae, interspaces with fine but distinct foveolae, sculpture weakening posteriorly; inner hypostomal teeth distinct, moderately high, closely spaced, triangular, with rounded apex and narrow base; outer hypostomal approximately smaller and thinner than inner hypostomal teeth, with moderately wide base, dentate; mesosoma shiny, foveolate with additional sparse and thick rugae on promesonotal dorsum and propodeum, sculpture slightly weakening on dorsum; anepisternum and mesosoma with indistinct sculpture or smooth; body reddish brown. ***Minor workers*.** Frons, genae, and malar area smooth and shiny; vertex smooth and shiny with few arcuate, interrupted rugae; promesonotum low, short, slightly convex, with relatively steep posterior declivity; propodeal spines very small, indistinct, triangular; mesosoma smooth and shiny, only dorsum with few transverse, thick rugulae.

###### Description.

**Major workers.** Measurements (*N* = 10): HL: 0.92–1.08 (0.97); HW: 0.83–0.99 (0.87); SL: 0.46–0.57 (0.49); EL: 0.08–0.11 (0.1); WL: 0.76–0.93 (0.8); PSL: 0.09–0.12 (0.11); MTL: 0.42–0.53 (0.45); PNW: 0.46–0.51 (0.49); PTW: 0.12–0.15 (0.13); PPW: 0.33–0.37 (0.35); CI: 89.1–92.7 (90.9); SI: 51.9–59.0 (55.6); PSLI: 9.4–13.2 (11.3); PPI: 35.9–44.1 (39.8); PNI: 51.4–59.4 (55.9); MTI: 49.5–53.5 (51.6). ***Head*.** In full-face view oval, with anterior and posterior sides slightly convex (Fig. 16B). In lateral view sub-oval; ventral and dorsal faces convex; dorsal face not depressed posteriorly; inner hypostomal teeth visible. Sides of the head with dense, long, erect pilosity; whole head with dense, short, suberect to erect pilosity. Antennal scrobes indistinct and not delimited by carinulae; scrobe surface shiny, with sparse, thick, longitudinal rugae, interspaces with fine but distinct foveolae. Occipital lobes and genae shiny, with thick, sparse, irregular rugae, interspaces with fine but distinct foveolae, sculpture weakening posteriorly; frons with dense, thick, and longitudinal rugae, interspaces with fine but distinct foveolae; malar area with dense, thick, longitudinal rugulae, interspaces smooth. Centre of clypeus smooth and shiny, lateral sides with longitudinal rugae; median notch present, wide, and indistinct; median longitudinal carina absent; lateral longitudinal carinae absent. Scape, when laid back, slightly exceeding the midlength of head; pilosity suberect to erect (Fig. 16B, D). Inner hypostomal teeth distinct, moderately high, closely spaced, triangular, with rounded apex and narrow base; outer hypostomal teeth approximately smaller and thinner than inner hypostomal teeth, with moderately wide base, dentate (Fig. 85T). ***Mesosoma*.** In lateral view, promesonotum short, angular, and relatively high, posterior mesonotum relatively steep, with small tubercle-like projections; promesonotal groove absent; metanotal groove absent; propodeal spines minute, triangular, with acute apex; humeral area laterally weakly produced (Fig. 16D). Surface shiny, foveolate with additional sparse and thick rugae on promesonotal dorsum and propodeum, sculpture slightly weakening on dorsum; anepisternum and mesosoma with indistinct sculpture or smooth. Pilosity sparse, long, and erect (Fig. 16D, F). ***Petiole*.** Shiny with fine and sparse foveolae; peduncle moderately long, with indistinct horizontal lobes on its basal part; node smooth, relatively low, triangular, with rounded apex, in rear view node dorsoventrally concave; pilosity moderately sparse and erect (Fig. 16D, F). ***Postpetiole*.** Shiny, with fine and sparse foveolae; in dorsal view postpetiole very wide, almost semi-oval; pilosity long, moderately sparse and erect (Fig. 16D, F). ***Gaster*.** Shiny and finely shagreened; pilosity dense, moderately long and erect (Fig. 16D, F). ***Colour*.** Unicolourous, reddish brown; legs dark yellow; gaster brown (Fig. 16D, F).

**Minor workers.** Measurements (*N* = 10): HL: 0.43–0.47 (0.45); HW: 0.39–0.42 (0.41); SL: 0.41–0.44 (0.42); EL: 0.07–0.09 (0.08); WL: 0.5–0.56 (0.53); PSL: 0.05–0.07 (0.06); MTL: 0.3–0.35 (0.32); PNW: 0.26–0.28 (0.27); PTW: 0.05–0.07 (0.06); PPW: 0.1–0.14 (0.12); CI: 86.1–95.8 (89.9); SI: 101.7–112.2 (104.8); PSLI: 11.9–15.2 (13.4); PPI: 47.3–59.8 (52.2); PNI: 65.4–72.7 (67.5); MTI: 74.3 –85.7 (79.0). ***Head*.** Occipital margin straight or indistinctly concave; occipital carina absent (Fig. 16A). Pilosity moderately dense, long, erect. Frons, genae, and malar area smooth and shiny; vertex smooth and shiny with few arcuate, interrupted rugae; antennal sockets with few thick, curved outward rugae. Clypeus with median longitudinal carina absent; two lateral longitudinal carinae absent. Scape, when laid back, surpassing the posterior head margin by one-fifth of its length; pilosity suberect (Fig. 16A, C). ***Mesosoma*.** In lateral view, promesonotum low, short, slightly convex, with relatively steep posterior declivity; promesonotal groove absent; metanotal groove absent; propodeal spines very small, indistinct, triangular, apex acute (Fig. 16C). Sculpture smooth and shiny, only dorsum with few transverse, thick rugulae. Pilosity sparse, moderately short, and erect (Fig. 16C, E). ***Petiole*.** Peduncle short and thin with ventral face slightly convex; node low, triangular, and small; with few short, erect setae (Fig. 16C, E). ***Postpetiole*.** Short, low, and slightly convex; with few short, erect setae (Fig. 16C, E). ***Gaster*.** With sparse, erect pilosity (Fig. 16C, E). ***Colour*.** Unicolourous, yellow (Fig. 16C, E).

###### Etymology.

From the type locality.

###### Biology.

The species was collected at elevation between 30–600 m, in gallery forest, in tropical dry forest, in rainforest. Nests were located in rotten logs and in dead twigs above ground.

###### Comments.

*Pheidole ranohirensis* sp. nov. is most similar to *P. lutea* sp. nov. and *P. voasara* sp. nov. ***Major workers*.***Pheidole ranohirensis* sp. nov. differs from *P. lutea* sp. nov. by surface of genae and antennal scrobes foveolate, dentate outer hypostomal teeth, which are smaller and thinner than inner hypostomal teeth and is never pointed outward, and by anepisternum, katepisternum, and mesosoma never entirely smooth; from *P. voasara* sp. nov. in indistinct or partially smooth sculpture on anepisternum and mesosoma, foveolate frons, and dentate outer hypostomal teeth, which are smaller and thinner than inner hypostomal teeth. ***Minor workers*.***Pheidole ranohirensis* sp. nov. differs from *P. lutea* sp. nov. in promesonotum low, short, slightly convex, and with relatively steep posterior declivity. Minor workers of *P. voasara* sp. nov. are unknown.

##### Pheidole voasara sp. nov.

Taxon classificationAnimaliaHymenopteraFormicidae

http://zoobank.org/67AF4A17-3387-43A2-A15E-25CB06CB3776

**Figure 17.**
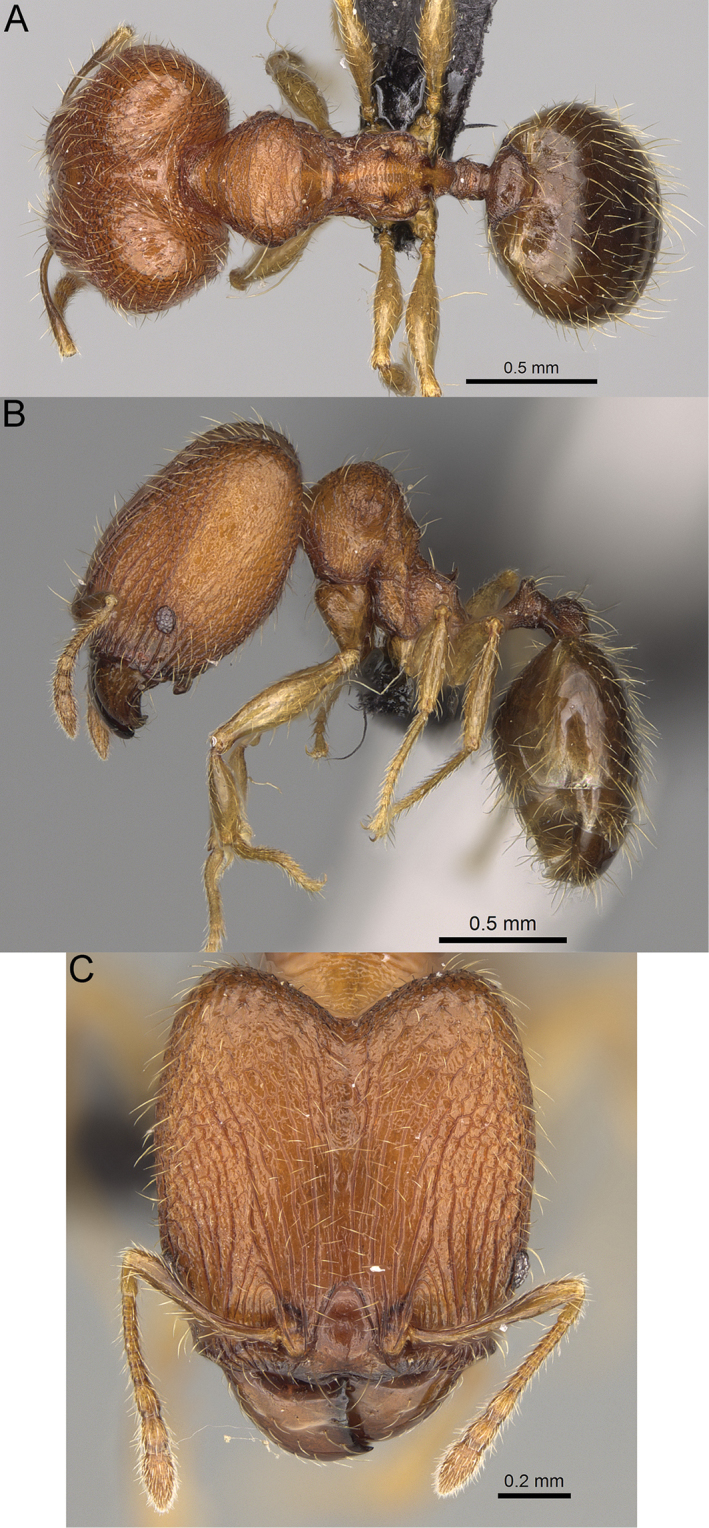
*Pheidole voasara* sp. nov., dorsal view (**A**), profile (**B**), and full-face view (**C**) of holotype major worker (CASENT0050060).

Figs 17A–C , 85Z , 88J

###### Type material.

***Holotype*.** Madagascar. •1 major worker; Toliara; Réserve Spéciale d’Ambohijanahary, Forêt d’Ankazotsihitafototra, 35.2 km 312°NW Ambaravaranala; -18.26667, 45.40667; alt. 1050 m; 13 Jan 2003; B.L. Fisher et al. leg.; BLF07018, CASENT0050060 (CASC).

###### Other material.

Madagascar. –***Fianarantsoa***: •1s.; Parc National d’Isalo, 9.1 km 354°N Ranohira; -22.48167, 45.46167; alt. 725 m; 27 Jan 2003; Fisher et al. leg.; CASENT0036511 (CASC). –***Toliara***: •2s.; Réserve Spéciale d’Ambohijanahary, Forêt d’Ankazotsihitafototra, 34.6 km 314°NW Ambaravaranala; -18.26, 45.41833; alt. 1100 m; 16 Jan 2003; Fisher et al. leg.; CASENT0029300, CASENT0029748 (CASC). •1s.; Réserve Spéciale d’Ambohijanahary, Forêt d’Ankazotsihitafototra, 35.2 km 312°NW Ambaravaranala; -18.26667, 45.40667; alt. 1050 m; 13 Jan 2003; Fisher et al. leg.; CASENT0028086 (CASC).

###### Diagnosis.

***Major workers*.** Head, in full-face view, oval, with anterior and posterior sides slightly convex; sides of the head with sparse, long, erect pilosity; occipital lobes and genae shiny, with sparse, indistinct and irregular rugae, interspaces with fine but distinct foveolae, sculpture weakening posteriorly; inner hypostomal teeth distinct, moderately high, closely spaced, triangular, with rounded apex and wide base, tops directed inward; outer hypostomal bigger and wider than inner hypostomal teeth, with wide base, lobe-like; mesosoma shiny, shiny, foveolate with additional sparse to moderately dense, and moderately thick, rugae, sculpture slightly weakening on dorsum; upper part of mesosoma with indistinct sculpture; body yellowish brown.

###### Description.

**Major workers.** Measurements (*N* = 10): HL: 1.05–1.2 (1.16); HW: 0.96–1.11 (1.06); SL: 0.46–0.52 (0.5); EL: 0.09–0.13 (0.11); WL: 0.79–0.89 (0.85); PSL: 0.12–0.15 (0.13); MTL: 0.46–0.52 (0.49); PNW: 0.46–0.56 (0.53); PTW: 0.13–0.17 (0.15); PPW: 0.3–0.42 (0.38); CI: 90.9–94.2 (92.0); SI: 45.3–49.6 (46.9); PSLI: 10.2–13.0 (11.5); PPI: 32.7–43.3 (39.2); PNI: 47.3–52.3 (49.6); MTI: 44.4–48.5 (46.3). ***Head*.** In full-face view oval, with anterior and posterior sides slightly convex (Fig. 17C). In lateral view sub-oval; ventral and dorsal faces convex; dorsal face not depressed posteriorly; inner hypostomal teeth visible. Sides of the head with moderately dense, long, erect pilosity; whole head with moderately dense, short, suberect to erect pilosity. Antennal scrobes indistinct and not delimited by carinulae; scrobe surface shiny, with sparse, thick, longitudinal to irregular rugae, interspaces with fine but distinct foveolae. Occipital lobes and genae shiny, with sparse, indistinct and irregular rugae, interspaces with fine but distinct foveolae, sculpture weakening posteriorly; frons with dense, thick, and longitudinal rugae, interspaces smooth; malar area with dense, thick, longitudinal rugulae, interspaces smooth. Centre of clypeus smooth and shiny, lateral sides with longitudinal rugae; median notch present, wide and indistinct; median longitudinal carina absent; lateral longitudinal carinae absent. Scape, when laid back, reaching the midlength of head; pilosity suberect to erect (Fig. 17B–C). Inner hypostomal teeth distinct, moderately high, closely spaced, triangular, with rounded apex and wide base, tops directed inward; outer hypostomal teeth larger and wider than inner, with wide base, lobe-like (Fig. 85Z). ***Mesosoma*.** In lateral view, promesonotum short, angular, and high, posterior mesonotum relatively steep, with small tubercle-like projections; promesonotal groove absent; metanotal groove absent; propodeal spines small, triangular, with acute apex; humeral area laterally weakly produced (Fig. 17B). Surface shiny, foveolate with additional sparse to moderately dense, and moderately thick rugae, sculpture slightly weakening on dorsum; upper part of mesosoma with indistinct sculpture. Pilosity moderately sparse, long, and erect (Fig. 17A, B). ***Petiole*.** Shiny and with foveolae; peduncle moderately long, with distinct horizontal lobes on its basal part; node relatively low, triangular, with rounded apex, in rear view node dorsoventrally concave; pilosity moderately dense and erect (Fig. 17A, B). ***Postpetiole*.** Shiny and with foveolae, dorsum partially smooth; short and flat; in dorsal view very wide, almost semi-oval; pilosity long, moderately dense, and erect (Fig. 17A, B). ***Gaster*.** Shiny and finely shagreened on the basal part of first tergite; pilosity dense, moderately long and erect (Fig. 17A, B). ***Colour*.** Unicolourous, orange to yellowish brown; gaster brown; legs dark yellow (Fig. 17A, B).

**Minor workers.** Unknown.

###### Etymology.

Malagasy for orange, in reference to bright body colouration.

###### Biology.

The species was collected at elevation between 725–1100 m, in gallery forest, and in montane rainforest. Nesting preferences unknown.

###### Comments.

Pheidole voasara sp. nov. is most similar to P. lutea sp. nov. and P. ranohirensis sp. nov. ***Major workers*.***Pheidole voasara* sp. nov. differs from *P. lutea* sp. nov. by surface of genae and antennal scrobes foveolate, lobe-like outer hypostomal teeth, inner hypostomal teeth pointed inward, and by anepisternum, katepisternum, and mesosoma never smooth; from *P. ranohirensis* sp. nov. in distinct and never smooth sculpture on anepisternum and mesosoma, frons never foveolate, and lobe-like outer hypostomal teeth, which are bigger and wider than inner hypostomal teeth. ***Minor workers*.** Unknown.

#### Revision of the *Pheidole navoatrensis* complex

**Diagnosis. *Major workers*.** Head, in full-face view, elongate; antennal scrobes absent or very indistinct, occipital lobes smooth, sometimes with indistinct, very sparse, longitudinal to irregular rugoreticulations, sculpture, when present, fading anteriorly; frons with thick, sparse, short and interrupted rugae and smooth interspaces or smooth with few short, thick, longitudinal rugae on the anterior part; genae smooth; promesonotum relatively low to high, and arched; propodeal spines absent, weakly developed, and lobe-like or small and short, triangular; mesosoma surface with fine, sparse to moderately sparse rugoreticulation (dorsal surface with weaker sculpture) or smooth with indistinct and sparse sculpture on lateral sides; gaster smooth; body yellow, orange and occasionally bright brown. ***Minor workers*.** Head smooth, only lateral sides of frons with short, indistinct, longitudinal rugulae; scape, when laid back, reaching posterior head margin; promesonotum, in lateral view, convex or box-like; mesosoma sculpture smooth and shiny, and sometimes lateral sides with indistinct, irregular, and sparse rugae; body yellow.

**Comments.** Major workers of this complex can be easily distinguished from others based on elongate head capsule, strongly reduced sculpture of head and mesosoma, strongly reduced or small propodeal spines, and bright body colouration. Minor workers can be separated from other species based on smooth and shiny head and mesosoma sculpture, short scape, and yellow body colouration.

##### Pheidole navoatrensis sp. nov.

Taxon classificationAnimaliaHymenopteraFormicidae

http://zoobank.org/BCC3CD28-96B1-4DFC-B2D3-CF13452587F6

**Figure 18.**
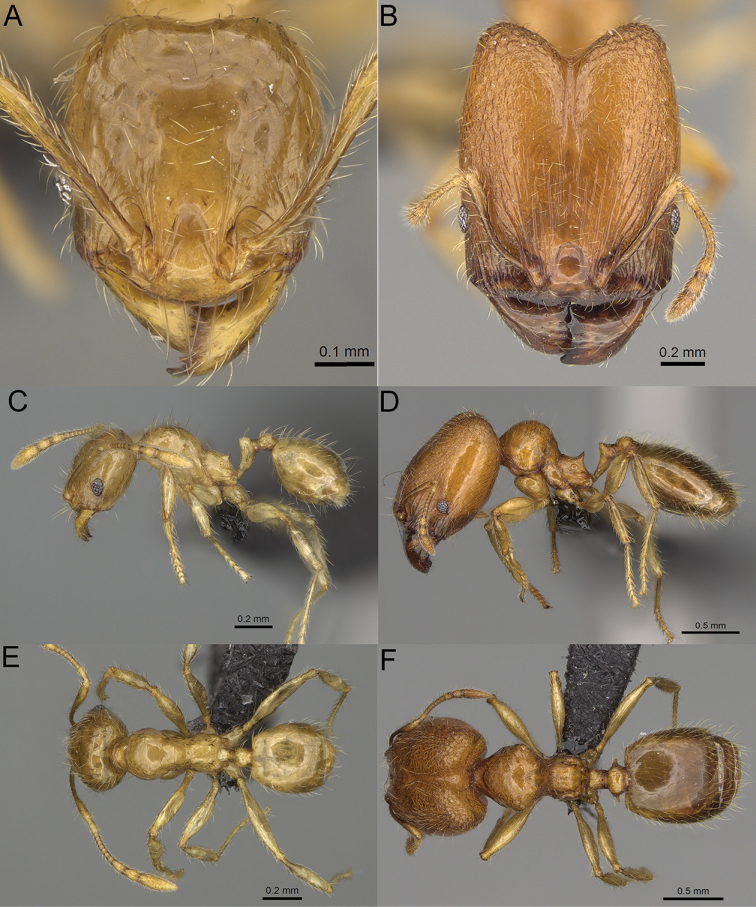
*Pheidole navoatrensis* sp. nov., full-face view (**A**), profile (**C**), and dorsal view (**E**) of paratype minor worker (CASENT0204254) and full-face view (**B**), profile (**D**), and dorsal view (**F**) of holotype major worker (CASENT0204235).

Figs 18A–F , 85M , 87R

###### Type material.

***Holotype*.** Madagascar. •1 major worker; Antananarivo; Navoatra I Non-Protected Area, 7.64 km NW Arivonimamo; -18.97806, 47.11929; alt. 1373 m; 6 May 2010; Andrianjaka & Ravelomanana leg.; CASENT0204235 (CASC). ***Paratype*.** Madagascar. •1w.; same data as for holotype; ARA0915, CASENT0204254 (CASC).

###### Other material.

Madagascar. –***Fianarantsoa***: •3w.; Ampangabe I Non-Protected Area, 21.4 km W Itremo; -20.61111, 46.60688; alt. 1414 m; 21 Mar 2010; Andrianjaka & Ravelomanana leg.; CASENT0211553, CASENT0213731, CASENT0213781 (CASC). •1w.; Ampangabe V Non-Protected Area, 21.37 km W Itremo; -20.61361, 46.60799; alt. 1449 m; 22 Mar 2010; Andrianjaka & Ravelomanana leg.; CASENT0207631 (CASC). •2w.; Ampangabe VI Non-Protected Area, 21.16 km W Itremo; -20.61444, 46.6104; alt. 1379 m; 21 Mar 2010; Andrianjaka & Ravelomanana leg.; CASENT0164859, CASENT0236320 (CASC). •1w.; Ampotoampoto I National Parc, 8.02 km NW Ilakaka; -22.62833, 45.18859; alt. 917 m; 26 Feb 2010; Andrianjaka & Ravelomanana leg.; CASENT0207300 (CASC). •3w.; Antohatsahomby I Non-Protected Area, 22.77 km NW Ambatofinandrahana; -20.55056, 46.58562; alt. 1550 m; 15 Mar 2010; Andrianjaka & Ravelomanana leg.; CASENT0207419, CASENT0207420, CASENT0207442 (CASC). •1w.; Antohatsahomby I Non-Protected Area, 22.77 km NW Ambatofinandrahana; -20.55056, 46.58562; alt. 1550 m; 15 Mar 2010; Andrianjaka & Ravelomanana leg.; CASENT0207433 (CASC). •1w.; Antohatsahomby II Non-Protected Area, 23.38 km NW Itremo; -20.55444, 46.58438; alt. 1640 m; 15 Mar 2010; Andrianjaka & Ravelomanana leg.; CASENT0211933 (CASC). •2s.; Parc National d’Isalo, 9.1 km 354°N Ranohira; -22.48167, 45.46167; alt. 725 m; 27 Jan 2003; Fisher et al. leg.; CASENT0036594, CASENT0036601 (CASC). –***Mahajanga***: •2s.; Parc National d’Ankarafantsika, Ampijoroa Station Forestière, 5.4 km 331°NW Andranofasika; -16.29889, 46.813; alt. 70 m; 26 Mar 2001; Fisher et al. leg.; CASENT0469065, CASENT0469084 (CASC). •1w.; Réserve forestière Beanka, 52.7 km E Maintirano; -18.0622, 44.52587; alt. 300 m; 24 Oct 2009; Fisher et al. leg.; CASENT0157649 (CASC). –***Toliara***: •10w.; Makay Mts.; -21.30997, 45.12946; alt. 590 m; 3 Dec 2010; Fisher et al. leg.; CASENT0205797, CASENT0205801, CASENT0205807, CASENT0205808, CASENT0205812, CASENT0205824, CASENT0205827, CASENT0205832, CASENT0205984 (CASC). •1s.; Réserve Spéciale Kalambatritra; -23.4185, 46.4583; alt. 1365 m; 8 Feb 2009; Fisher et al. leg.; CASENT0149710 (CASC).

###### Diagnosis.

***Major workers*.** Head elongate; sides of the head with very sparse, moderately short, erect pilosity; frons smooth, with few short, thick, longitudinal rugae on the anterior part; genae shiny and smooth; inner hypostomal teeth distinct, small, closely spaced, triangular, with rounded apex; outer hypostomal teeth weakly developed, dentate; body yellow to orange; mesosoma smooth, sometimes with indistinct and sparse foveolae or rugulae on propodeum and promesonotum. ***Minor workers*.** Body yellow; head shiny and smooth, only lateral sides of frons with short, indistinct, longitudinal rugulae; propodeal spines short and triangular; mesosoma smooth and shiny.

###### Description.

**Major workers.** Measurements (*N* = 10): HL: 1.09–1.45 (1.21); HW: 0.86–1.14 (0.94); SL: 0.46–0.58 (0.5); EL: 0.12–0.15 (0.13); WL: 0.84–1.02 (0.91); PSL: 0.14–0.19 (0.17); MTL: 0.49–0.6 (0.53); PNW: 0.5–0.65 (0.55); PTW: 0.15–0.19 (0.17); PPW: 0.3–0.41 (0.34); CI: 74.5–80.3 (77.8); SI: 50.2–56.7 (53.1); PSLI: 12.9–15.6 (14.0); PPI: 43.3–54.9 (49.3); PNI: 56.6–61.4 (58.0); MTI: 52.4 –59.9 (57.1). ***Head*.** In full-face view longer than wide, anterior of eyes straight, posterior of eyes straight and slightly convex (Fig. 18B). In lateral view sub-oval; ventral and dorsal faces convex; dorsal face not depressed posteriorly; inner hypostomal teeth invisible. Sides of the head with very sparse, moderately short, erect pilosity; whole head with moderately dense, very short, suberect to erect pilosity. Antennal scrobes very weakly impressed and not delimited, scrobe surface shiny, with thick, longitudinal, short, and interrupted rugae; interspaces distinctly rugo-foveolate. Occipital lobes shiny, with thick, irregular rugae, interspaces rugo-foveolate, rugae fading anteriorly; frons smooth, with few short, thick, longitudinal rugae on the anterior part; genae shiny and smooth; malar area with thin, longitudinal, moderately dense rugae, interspaces smooth or with indistinct rugulae. Centre of clypeus shiny and smooth, lateral sides with longitudinal rugulae; median notch present, moderately wide and shallow; median longitudinal carina absent; lateral longitudinal carinae indistinct. Scape, when laid back, not reaching the midlength of head; pilosity subdecumbent to erect (Fig. 18B, D). Inner hypostomal teeth distinct, small, closely spaced, triangular, with rounded apex; outer hypostomal teeth weakly developed, dentate (Fig. 85M). ***Mesosoma*.** In lateral view, promesonotum relatively low and arched, posterior mesonotum convex, without tubercle-like projections; promesonotal groove absent; metanotal groove absent; propodeal spines small, triangular, with sharp apex and wide base; humeral area laterally weakly produced (Fig. 18D). Surface shiny and smooth, sometimes with indistinct and sparse foveolae or rugulae on propodeum and promesonotum. Pilosity moderately dense, very long and suberect to erect (Fig. 18D, F). ***Petiole*.** Shiny; peduncle moderately long, finely foveolate, without horizontal lobes on its basal part; node smooth, relatively low, triangular, with rounded apex, in rear view node slightly concave; pilosity moderately dense and erect (Fig. 18D, F). ***Postpetiole*.** Shiny; in dorsal view sides with moderately short, acute, and triangular projections; pilosity long, moderately dense and erect (Fig. 18D, F). ***Gaster*.** Shiny and smooth; pilosity dense, short, and erect (Fig. 18D, F). ***Colour*.** Yellow to orange; malar area, lower frons and gaster dark yellow to yellowish brown (Fig. 18D, F).

**Minor workers.** Measurements (*N* = 5): HL: 0.45–0.49 (0.47); HW: 0.41–0.45 (0.43); SL: 0.39–0.43 (0.41); EL: 0.09–0.11 (0.1); WL: 0.53–0.59 (0.54); PSL: 0.06–0.09 (0.07); MTL: 0.33–0.36 (0.35); PNW: 0.27–0.3 (0.28); PTW: 0.06–0.08 (0.07); PPW: 0.1–0.12 (0.11); CI: 88.7–90.7 (89.7); SI: 90.7–99.1 (95.6); PSLI: 12.6–17.5 (15.1); PPI: 61.5–75.5 (66.2); PNI: 64.0–68.5 (66.7); MTI: 78.0–84.3 (80.7). ***Head*.** Occipital margin straight or indistinctly concave; occipital carina absent (Fig. 18A). Pilosity moderately dense, long, suberect. Whole head shiny and smooth, only lateral sides of frons with short, indistinct, longitudinal rugulae; antennal sockets with sparse carinae curved outward. Clypeus with median longitudinal carina absent; two lateral longitudinal carinae absent. Scape, when laid back, reaching posterior head margin; pilosity suberect to erect (Fig. 18A, C). ***Mesosoma*.** In lateral view, promesonotum convex; promesonotal groove absent; metanotal groove indistinct; propodeal spines short and triangular, apex acute (Fig. 18C, E). Sculpture smooth and shiny. Pilosity moderately dense, long, and erect (Fig. 18C, E). ***Petiole*.** Peduncle short and thin with ventral face slightly convex; node globular; with few moderately long, erect setae (Fig. 18C, E). ***Postpetiole*.** Short, low, and convex; with few moderately long, erect setae (Fig. 18C, E). ***Gaster*.** With moderately dense, erect pilosity (Fig. 18C, E). ***Colour*.** Unicolourous, yellow (Fig. 18C, E).

###### Etymology.

From the type locality.

###### Biology.

The species was collected at elevation between 70–1640 m, in dry forest on sandy soil, in Uapaca woodland, in savannah woodland, in tropical dry forest, in gallery forest, in shrubland. Nests were located in soil and under stones.

###### Comment.

*Pheidole navoatrensis* sp. nov. is most similar to *P. typhlos* sp. nov. ***Major workers*.***Pheidole navoatrensis* sp. nov. differs from *P. typhlos* sp. nov. in well-developed eyes, never smooth occipital lobes, small inner hypostomal teeth and weakly developed outer hypostomal teeth, and posterior mesonotum never convex. ***Minor workers*.***Pheidole navoatrensis* sp. nov. differs from *P. typhlos* sp. nov. in convex promesonotum, big eyes, and shallow metanotal groove.

##### Pheidole parviocula sp. nov.

Taxon classificationAnimaliaHymenopteraFormicidae

http://zoobank.org/9627A1BF-A948-432D-A0F7-334E6D92D970

**Figure 19.**
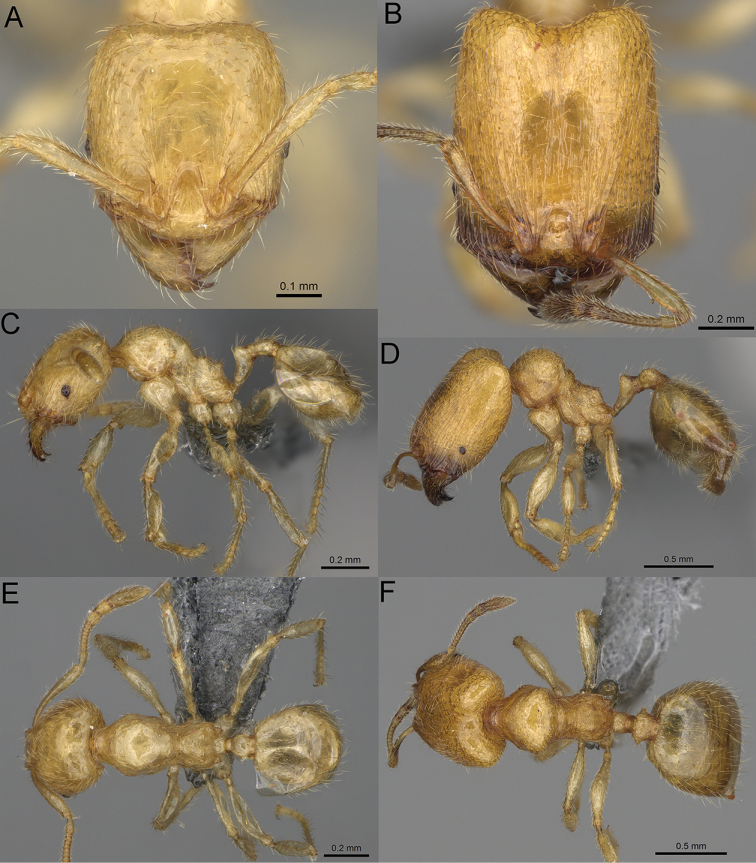
*Pheidole parviocula* sp. nov., full-face view (**A**), profile (**C**), and dorsal view (**E**) of paratype minor worker (CASENT0923187) and full-face view (**B**), profile (**D**), and dorsal view (**F**) of holotype major worker (CASENT0303345).

Figs 19A–F , 85P , 87U

###### Type material.

***Holotype*.** Madagascar. •1 major worker; Antananarivo; Ankalalahana; -19.00716, 47.1124; alt. 1370 m; 3 Jan 2013; B.L. Fisher et al. leg.; BLF31313, CASENT0303345 (CASC). ***Paratype*.** Madagascar. •1 w.; same data as for holotype; CASENT0923187 (CASC).

###### Other material.

Madagascar. – ***Antananarivo***: •2w., 2s.; Andohony I Non-Protected Area, 22.62 km SW Antsirabe; -20.06784, 46.99068; alt. 1451 m; 6 Mar 2012; Andrianjaka & Ravelomanana leg.; CASENT0302331, CASENT0302483 (CASC). •2w.; Antaponimanadala I Non-Protected Area, 6.59 km E Manalalondo; -19.25528, 47.1771; alt. 1984 m; 13 May 2010; Andrianjaka & Ravelomanana leg.; CASENT0211687, CASENT0211787 (CASC). •3s.; Antaponimanadala III Non-Protected Area, 6.55 km E Manalalondo; -19.25583, 47.17751; alt. 1987 m; 17 May 2010; Andrianjaka & Ravelomanana leg.; CASENT0236559, CASENT0284933, CASENT0284998 (CASC). •2w., 1s., 1m.; Antaponimanadala IV Non-Protected Area, 6.66 km E Manalalondo; -19.25361, 47.17634; alt. 1951 m; 15 May 2010; Andrianjaka & Ravelomanana leg.; CASENT0228718, CASENT0228735 (CASC). •1w.; Beapombo II Non-Protected Area, 22.65 km SW Antsirabe; -20.07022, 47.00555; alt. 1689 m; 28 Feb 2012; Andrianjaka & Ravelomanana leg.; CASENT0292480 (CASC). •1w.; Navoatra II Non-Protected Area, 7.54 km NW Arivonimamo; -18.97889, 47.11975; alt. 1357 m; 6 May 2010; Andrianjaka & Ravelomanana leg.; CASENT0203015 (CASC). –***Fianarantsoa***: •3w., 1s.; Ambinanindranomena Non-Protected Area, 39.16 km SE Ambalavao; -21.96077, 47.29125; alt. 1002 m; 3 Feb 2012; Andrianjaka & Ravelomanana leg.; CASENT0285095, CASENT0289395, CASENT0289462, CASENT0289567 (CASC). •3w., 1s.; Ampanenitra Non-Protected Area, 41.19 km SE Ambalavao; -21.9652, 47.31001; alt. 1010 m; 8 Feb 2010; Andrianjaka & Ravelomanana leg.; CASENT0289604, CASENT0289694, CASENT0289797, CASENT0292637 (CASC). •4w.; Ampangabe IV Non-Protected Area, 21.37 km W Itremo; -20.61278, 46.60774; alt. 1417 m; 21 Mar 2010; Andrianjaka & Ravelomanana leg.; CASENT0236453, CASENT0236497, CASENT0236524, CASENT0236525 (CASC). •1w., 1s.; Mampiarika III Non-Protected Area, 28.93 km SW Ambositra; -20.73583, 47.08399; alt. 1487 m; 1 Feb 2010; Andrianjaka & Ravelomanana leg.; CASENT0165724, CASENT0165857 (CASC). •1w.; Manandriana I Non-Protected Area, 27.11 km SW Ambositra; -20.73194, 47.09413; alt. 1590 m; 9 Feb 2010; Andrianjaka & Ravelomanana leg.; CASENT0167038 (CASC). •1s., 1m.; Manandriana III Non-Protected Area, 27.25 km SW Ambositra; -20.73333, 47.09391; alt. 1578 m; 8 Feb 2010; Andrianjaka & Ravelomanana leg.; CASENT0206047, CASENT0210577 (CASC). •1w.; Soanierenana I Non-Protected Area, 25.33 km SW Ambositra; -20.72139, 47.10994; alt. 1723 m; 6 Feb 2010; Andrianjaka & Ravelomanana leg.; CASENT0207108 (CASC). •3s.; Soanierenana III Non-Protected Area, 25.25 km SW Ambositra; -20.72194, 47.11019; alt. 1707 m; 5 Feb 2010; Andrianjaka & Ravelomanana leg.; CASENT0213472, CASENT0213840, CASENT0213844 (CASC). •1w., 1s., 1m.; Soanierenana IV Non-Protected Area, 25.22 km SW Ambositra; -20.72389, 47.10705; alt. 1736 m; 5 Feb 2010; Andrianjaka & Ravelomanana leg.; CASENT0168320, CASENT0168431 (CASC). –***Toliara***: •1w.; Réserve Spéciale Kalambatritra; -23.4185, 46.4583; alt. 1365 m; 8 Feb 2009; B.L. Fisher et al. leg.; CASENT0149620 (CASC).

###### Diagnosis.

***Major workers*.** Eyes small and reduced; body yellow; head rectangular, anterior of eyes relatively straight, posterior of eyes slightly convex; sides of the head with dense, relatively long, erect pilosity; occipital lobes and genae shiny and smooth, sometimes with indistinct, very sparse, longitudinal rugulae; frons and malar area shiny, with thick, sparse, short, and interrupted rugae, interspaces smooth; propodeal spines absent or weakly developed, lobe-like; mesosoma shiny, with fine, sparse to moderately sparse rugoreticulation, sculpture on dorsum weaker or with smooth patches; inner hypostomal teeth indistinct, very low, lobe-like, with rounded apex; outer hypostomal teeth distinct, dentate, and with rounded tops directed outward. ***Minor workers*.** Eyes small and reduced; body yellow; head and mesosoma shiny and smooth; propodeal spines indistinct, triangular.

###### Description.

**Major workers.** Measurements (*N* = 10): HL: 0.72–1.07 (0.93); HW: 0.6–0.83 (0.74); SL: 0.36–0.48 (0.43); EL: 0.05–0.07 (0.06); WL: 0.7–0.88 (0.8); PSL: 0.08–0.12 (0.1); MTL: 0.37–0.46 (0.43); PNW: 0.38–0.53 (0.47); PTW: 0.11–0.16 (0.14); PPW: 0.23–0.32 (0.28); CI: 76.1–82.9 (80.1); SI: 55.8–61.4 (58.4); PSLI: 9.7–12.2 (11.0); PPI: 45.2–54.6 (49.1); PNI: 60.0–65.5 (63.2); MTI: 54.7–62.9 (58.0). ***Head*.** In full-face view elongate, anterior of eyes relatively straight, posterior of eyes slightly convex (Fig. 19B). In lateral view sub-oval; ventral and dorsal faces moderately convex; inner hypostomal teeth invisible. Sides of the head with dense, relatively long, erect pilosity; whole head with moderately dense, short, suberect to erect pilosity. Antennal scrobes absent. Occipital lobes and genae shiny and smooth, sometimes with indistinct, very sparse, longitudinal rugulae; frons and malar area shiny, with thick, sparse, short and interrupted rugae, interspaces smooth. Clypeus shiny and smooth, with a few longitudinal rugae on the lateral sides; median notch present, wide, and shallow; median longitudinal carina indistinct; lateral longitudinal carinae absent. Scape, when laid back, reaching slightly over the midlength of head; pilosity decumbent to erect (Fig. 19B, D). Inner hypostomal teeth indistinct, very low, lobe-like, with rounded apex; outer hypostomal teeth distinct, dentate, and with rounded tops directed outward (Fig. 85P). ***Mesosoma*.** In lateral view, promesonotum short, angular, and low, posterior mesonotum convex, with low tubercle-like projection; promesonotal groove present; metanotal groove absent; propodeal spines absent or weakly developed, lobe-like; humeral area with short triangular tubercles (Fig. 19D). Surface shiny, with fine, sparse to moderately sparse rugoreticulation, sculpture on dorsum weaker or with smooth patches. Pilosity moderately dense, long, and erect (Fig. 19D, F). ***Petiole*.** Peduncle relatively long, without horizontal lobes on its basal part; node moderately high and globular, with convex apex, in rear view node concave; pilosity moderately dense and erect (Fig. 19D, F). ***Postpetiole*.** Smooth and shiny; in dorsal view sides with short, acute, and tubercle-like projections; pilosity long, moderately dense and erect (Fig. 19D, F). ***Gaster*.** Smooth and shiny; pilosity dense, short and erect (Fig. 19D, F). ***Colour*.** Yellow, malar area and lower part of frons dark yellow to bright brown (Fig. 19D, F).

**Minor workers.** Measurements (*N* = 10): HL: 0.45–0.51 (0.49); HW: 0.4–0.45 (0.44); SL: 0.34–0.37 (0.35); EL: 0.04–0.06 (0.05); WL: 0.5–0.58 (0.55); PSL: 0.05–0.07 (0.06); MTL: 0.25–0.31 (0.29); PNW: 0.26–0.3 (0.29); PTW: 0.07–0.08 (0.07); PPW: 0.11–0.12 (0.11); CI: 86.4–92.8 (89.6); SI: 76.9–85.4 (81.1); PSLI: 9.3–13.5 (11.8); PPI: 60.2–72.4 (65.1); PNI: 59.1–70.1 (65.9); MTI: 62.4–71.5 (66.9). ***Head*.** In full-face view rectangular, posterior and anterior of eyes slightly convex, occipital margin straight or indistinctly convex; occipital carina absent (Fig. 19A). Pilosity moderately dense, short, subdecumbent to suberect. Whole head shiny and smooth, only antennal sockets with sparse carinae curved outward. Clypeus with median longitudinal carina absent; two lateral longitudinal carinae absent. Scape, when laid back, reaching posterior head margin; pilosity suberect to erect (Fig. 19A, C). ***Mesosoma*.** In lateral view, promesonotum convex; promesonotal groove present; metanotal groove indistinct; propodeal spines indistinct, triangular, apex acute (Fig. 19C). Sculpture smooth and shiny. Pilosity moderately sparse, moderately long and erect (Fig. 19C, E). ***Petiole*.** Peduncle moderately short and thin; node globular and moderately high; with few long, erect setae (Fig. 19C, E). ***Postpetiole*.** Moderately short, low, and slightly convex; with few long, erect setae at the anterior edge (Fig. 19C, E). ***Gaster*.** Pilosity moderately dense, erect, and moderately long (Fig. 19C, E). ***Colour*.** Unicolourous, yellow (Fig. 19C, E).

###### Etymology.

Latin for small eyes.

###### Biology.

The species was collected at elevation between 1002–1987 m, in savannah grassland, in Uapaca woodland, in savannah woodland, and in grassland. Nests were located in soil, under stones, and in dead tree stumps.

###### Comments.

This species is most similar to *P. typhlos* sp. nov. ***Major workers*.***Pheidole parviocula* sp. nov. can be distinguished from *P. typhlos* sp. nov. by dense, relatively long pilosity on sides of the head, small, lobe-like inner hypostomal teeth and weakly developed or absent propodeal spines. ***Minor workers*.***Pheidole parviocula* sp. nov. can be distinguished from *P. typhlos* sp. nov. by convex shape of promesonotum convex, presence of promesonotal groove and weakly developed, small, triangular, apex acute.

##### Pheidole typhlos sp. nov.

Taxon classificationAnimaliaHymenopteraFormicidae

http://zoobank.org/99FD33D4-9BC1-4595-9231-1A85822A7B35

**Figure 20.**
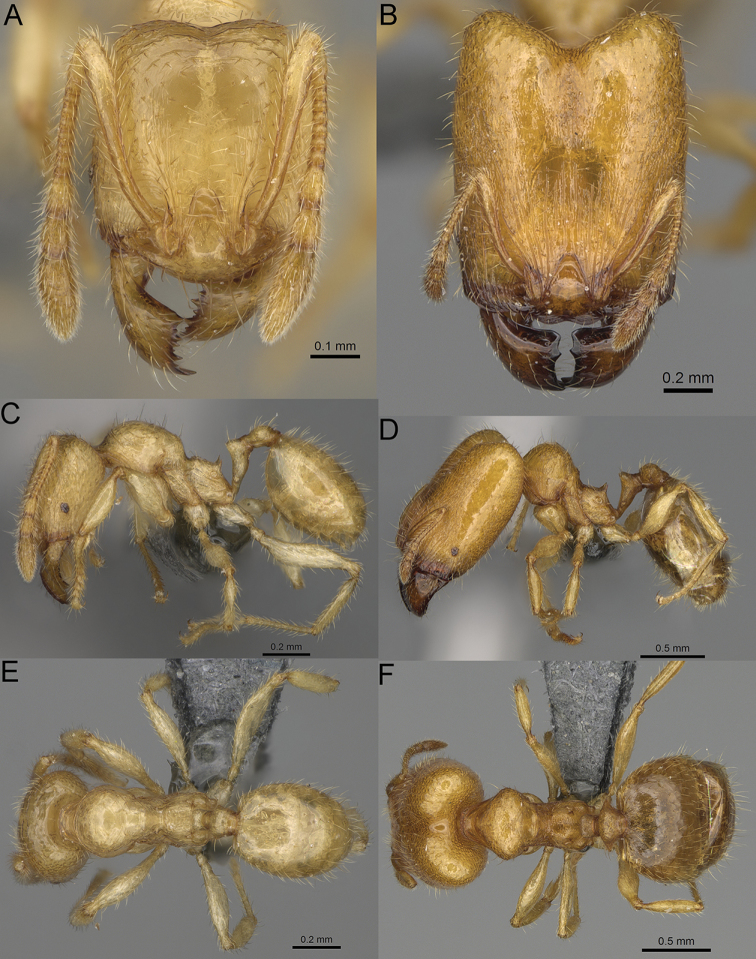
*Pheidole typhlos* sp. nov., full-face view (**A**), profile (**C**), and dorsal view (**E**) of paratype minor worker (CASENT0923217) and full-face view (**B**), profile (**D**), and dorsal view (**F**) of holotype major worker (CASENT0302953).

Figs 20A–F , 85X , 88H

###### Type material.

***Holotype*.** Madagascar. •1 major worker; Antsiranana; Galoko chain, Mont Galoko; -13.58487, 48.71818; alt. 520 m; 19 Feb 2013; B.L. Fisher et al. leg.; BLF30851, CASENT0302953 (CASC). ***Paratype*.** Madagascar. •1 w.; same data as for holotype; CASENT0923217 (CASC).

###### Other material.

Madagascar. – ***Antananarivo***: •6s.; Galoko chain, Mont Galoko; -13.58487, 48.71818; alt. 520 m; 16 Feb 2013; B.L. Fisher et al. leg.; CASENT0305030 (CASC). •1w.; Galoko chain, Mont Galoko; -13.5888, 48.72864; alt. 980 m; 22 Feb 2013; B.L. Fisher et al. leg.; CASENT0304748 (CASC).

###### Diagnosis.

***Major workers*.** Eyes small and reduced; body yellow; head rectangular, anterior of eyes relatively straight, posterior of eyes slightly convex; sides of the head with sparse, short, erect pilosity; occipital lobes, genae, and posterior part of frons shiny and smooth, sometimes with indistinct, very sparse, longitudinal rugulae; anterior part of frons and malar area shiny, with thick, very sparse, short, and interrupted rugae, interspaces smooth; propodeal spines short, triangular, with acute top; pronotum smooth and shiny, sometimes on lateral sides with indistinct and sparse rugulae; mesonotum and propodeum shiny, with fine, sparse to moderately sparse rugoreticulation; katepisternum indistinctly foveolate; anepisternum with thick rugoreticulation; inner hypostomal teeth distinct, moderately high, narrow, triangular, with rounded apex; outer hypostomal teeth distinct, triangular, and with rounded tops directed outward; outer hypostomal teeth are slightly lower than inner hypostomal teeth. ***Minor workers*.** Eyes small and reduced; body yellow; head and mesosoma shiny and smooth, lateral sides of frons with short, indistinct, longitudinal rugulae; propodeal spines short and triangular.

###### Description.

**Major workers.** Measurements (*N* = 8): HL: 1.2–1.34 (1.28); HW: 0.96–1.07 (1.01); SL: 0.49–0.52 (0.5); EL: 0.08–0.12 (0.1); WL: 0.79–0.91 (0.84); PSL: 0.15–0.19 (0.16); MTL: 0.48–0.52 (0.51); PNW: 0.48–0.55 (0.51); PTW: 0.14–0.16 (0.15); PPW: 0.37–0.43 (0.4); CI: 75.1–82.0 (79.5); SI: 48.0–52.3 (49.8); PSLI: 11.9–14.3 (12.7); PPI: 36.3–39.9 (37.9); PNI: 47.4–55.3 (50.6); MTI: 46.9–52.9 (49.8). ***Head*.** In full-face view elongate, anterior of eyes relatively straight, posterior of eyes slightly convex (Fig. 20B). In lateral view sub-oval; ventral and dorsal faces relatively flat; inner hypostomal teeth invisible. Antennal scrobes absent. Sides of the head with sparse, short, erect pilosity; whole head with dense, short, suberect to erect pilosity. Occipital lobes, genae, and posterior part of frons shiny and smooth, sometimes with indistinct, very sparse, longitudinal rugulae; anterior part of frons and malar area shiny, with thick, very sparse, short, and interrupted rugae, interspaces smooth. Clypeus shiny and smooth, with a few indistinct, longitudinal rugae on the lateral sides; median notch present, wide, and shallow; median longitudinal carina absent; lateral longitudinal carinae absent. Scape, when laid back, reaching the midlength of head; pilosity decumbent to erect (Fig. 20B, D). Inner hypostomal teeth distinct, moderately high, narrow, triangular, with rounded apex; outer hypostomal teeth distinct, triangular, and with rounded tops directed outward; outer hypostomal teeth are slightly lower than inner hypostomal teeth (Fig. 85X). ***Mesosoma*.** In lateral view, promesonotum short, angular, and moderately high, posterior mesonotum convex, with indistinct tubercle-like projection; promesonotal groove absent; metanotal groove absent or very indistinct; propodeal spines short, triangular, with acute top; humeral area with wide and flat tubercles (Fig. 20D). Pronotum smooth and shiny, sometimes on lateral sides with indistinct and sparse rugulae; mesonotum and propodeum shiny, with fine, sparse to moderately sparse rugoreticulation; katepisternum indistinctly foveolate; anepisternum with thick rugoreticulation. Pilosity moderately dense, long, and erect (Fig. 20D, F). ***Petiole*.** Shiny; peduncle finely foveolate, without horizontal lobes on its basal part; node smooth, moderately high, with convex apex, in rear view node dorsoventrally concave; pilosity moderately dense and erect (Fig. 20D, F). ***Postpetiole*.** Finely foveolate and shiny; in dorsal view sides with moderately long, acute, and triangular projections; pilosity moderately long, moderately dense, and erect (Fig. 20D, F). ***Gaster*.** Smooth and shiny; pilosity dense, short, and erect (Fig. 20D, F). ***Colour*.** Yellow, malar area and lower part of frons dark yellow to bright brown (Fig. 20D, F).

**Minor workers.** Measurements (*N* = 1): HL: 0.55; HW: 0.46; SL: 0.45; EL: 0.05; WL: 0.62; PSL: 0.09; MTL: 0.35; PNW: 0.32; PTW: 0.09; PPW: 0.14; CI: 83.9; SI: 97.4; PSLI: 16.4; PPI: 61.2; PNI: 69.8; MTI: 76.7. ***Head*.** In full-face view rectangular, posterior and anterior of eyes slightly convex, occipital margin straight or indistinctly concave; occipital carina absent (Fig. 20A). Pilosity moderately dense, short, subdecumbent to suberect. Whole head shiny and smooth, only lateral sides of frons with short, indistinct, longitudinal rugulae; antennal sockets with sparse carinae curved outward. Clypeus with median longitudinal carina absent; two lateral longitudinal carinae absent. Scape, when laid back, reaching posterior head margin; pilosity suberect to erect (Fig. 20A, C). ***Mesosoma*.** In lateral view, promesonotum box-like; promesonotal groove absent; metanotal groove distinct; propodeal spines short and triangular, apex acute (Fig. 20C). Sculpture smooth and shiny, sometimes lateral sides with indistinct, irregular, and sparse rugae. Pilosity moderately sparse, short, and erect (Fig. 20C, E). ***Petiole*.** Peduncle short with ventral face slightly convex; node globular; with few short, erect setae (Fig. 20C, 20E). ***Postpetiole*.** Short, low, and convex; with few short, erect setae (Fig. 20C, E). ***Gaster*.** Pilosity moderately dense, erect, and short (Fig. 20C, E). ***Colour*.** Unicolourous, yellow (Fig. 20C, E).

###### Etymology.

Greek for blind [t?f???], in reference to the reduced eyes.

###### Biology.

The species was collected at elevation between 520–980 m, in rainforest, in montane forest. Nesting preferences unknown.

###### Comments.

This species is most similar to *P. parviocula* sp. nov. ***Major workers*.***Pheidole typhlos* sp. nov. can be distinguished from *P. parviocula* sp. nov. by sparse and short pilosity on sides of the head, presence of distinct, triangular inner hypostomal teeth and presence of short, triangular propodeal spines. ***Minor workers*.***Pheidole typhlos* sp. nov. can be distinguished from *P. parviocula* sp. nov. by box-like promesonotum, absence of promesonotal groove and presence of short and triangular propodeal spines.

#### Revision of the *Pheidole longispinosa* group

**Diagnosis. *Major workers*.** Large species; head, in full-face view, trapezoid, widened posteriorly, in lateral view sub-oval, ventral and dorsal faces convex, dorsal face not depressed posteriorly; antennal scrobes absent or indistinct; occipital lobes smooth, at least on the posterior part; genae smooth to finely rugulose; head sculpture weakens posteriorly; promesonotum short, low, and evenly convex; propodeal spines very long; first gastral tergite smooth to shagreened; body brown to black. ***Minor workers*.** Head smooth to rugoreticulate, central part of frons usually with smooth notch; scape, when laid back, surpassing posterior head margin by one third or more than half of its length; promesonotum low, long, and slightly convex; promesonotal groove absent or very indistinct; metanotal groove very indistinct; propodeal spines very long; petiole with long and thin peduncle; body brown to black.

**Comments.** Members of this group are divided into two complexes. The *P. longispinosa* complex contains three species: *P. longispinosa* Forel, *P. praegrandis* sp. nov., and *P. mahaboensis* sp. nov. Majority of records of *Pheidole longispinosa* come from central highlands, but the species is known also from dry deciduous biome and evergreen rainforest. *Pheidole mahaboensis* is distributed cross the evergreen rainforest biome and central highlands, in southern part of its distribution range *P. mahaboensis* occurs sympatric with *P. longispinosa*. *Pheidole praegrandis* is known exclusively from the evergreen rainforest biome and a centre of its distribution is located in its northernmost part. However, the species is sympatric with *P. mahaboensis* in remaining parts of this biome. The *P. scabrata* complex contains two species: *P. scabrata* and *P. maizina* sp. nov. of sympatric distribution limited to the northern parts of evergreen forest and central highlands biomes.

##### Key to the *Pheidole longispinosa* group

**Table:** 

1	Major workers. Sides of the head with relatively long, erect pilosity; antennal lobes never predominately smooth; promesonotum with strong sculpture (Fig. 21). Minor workers. Scape, when laid back, surpassing posterior head margin by one third of its length; mesosoma with dense rugoreticulation, sometimes dorsum with weaker sculpture, but never smooth (Fig. 21)	**2**
–	Major workers. Sides of the head without or with short and decumbent pilosity; promesonotum predominately smooth (Fig. 22). Minor workers. Scape, when laid back, surpassing posterior head margin by more than half of its length; mesosoma smooth, indistinctly rugulose or the whole surface finely rugoreticulate (Fig. 22)	**3**
2	Major workers. Genae smooth and shiny on the entire surface or indistinctly rugulose, tips of outer hypostomal teeth never directed outward, inner hypostomal teeth slightly bigger than outer hypostomal teeth; katepisternum with smooth area; body brown to dark brown (Fig. 21A, B, G). Minor workers. Head sculpture smooth or indistinctly rugoreticulate on its central part, genae always smooth (Fig. 21D, E)	***P. scabrata* Forel**
–	Major workers. Genae with rugulae, sometimes posterior part with reduced sculpture, tips of outer hypostomal teeth directed outward; inner hypostomal teeth distinctly bigger than outer hypostomal teeth; katepisternum never with smooth area; body dark brown to black (Fig. 21C, F). Minor workers. Head sculpture rugoreticulate, genae never smooth (Fig. 21H, I)	***P. maizina* sp. nov.**
3	Major workers. Head and first gastral tergite at least partially shagreened, body dark brown to black (Fig. 22A, H). Minor workers. Mesosoma rugoreticulate, body dark brown to black (Fig. 15E)	***P. praegrandis* sp. nov.**
–	Major workers. Head and first gastral tergite never shagreened, body brown to dark brown (Fig. 22B–D). Minor workers. Mesosoma smooth or with reduced and indistinct rugulae, body brown to dark brown (Fig. 22F, G)	**4**
4	Major workers. Metanotal groove absent or shallow, frons with longitudinal rugae never reaching further than midlength of head (Fig. 22C). Minor workers. Promesonotal groove absent, metanotal groove shallow and wide, mesosoma with reduced sculpture (Fig. 22G)	***P. longispinosa* Forel**
–	Major workers. Metanotal groove present; frons with longitudinal rugae reaching further than midlength of head (Fig. 22B). Minor workers. Promesonotal groove present, metanotal groove narrow and relatively deep, mesosoma with fine superficial rugulae (Fig. 22F)	***P. mahaboensis* sp. nov.**

**Figure 21.**
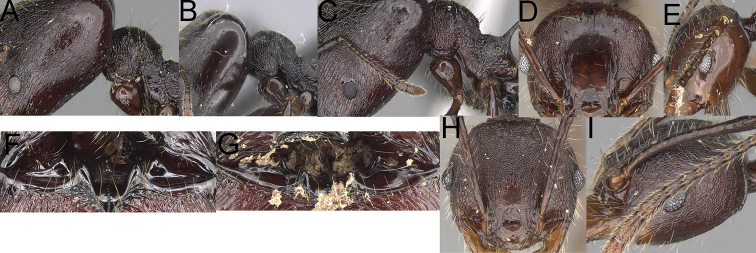
*Pheidole scabrata*, head and profile of major worker (**A, B**), head of minor worker (**D, E**), and hypostomal teeth (**G**). *Pheidole maizina* sp. nov., head and profile of major worker (**C**), head of minor worker (**H, I**), and hypostomal teeth (**F**).

**Figure 22.**
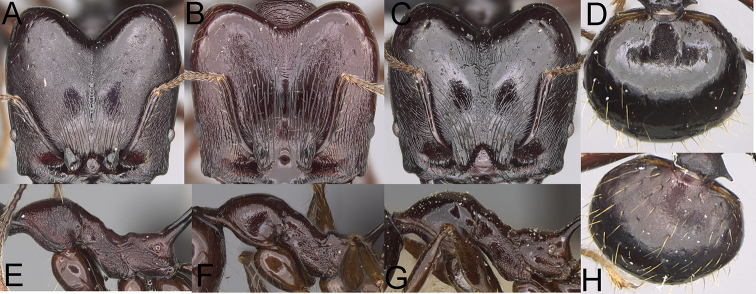
*Pheidole praegrandis* sp. nov., head of major worker (**A**), profile of minor worker (**E**), and gaster (**H**). *Pheidole mahaboensis* sp. nov., head of major worker (**B**), profile of minor worker (**F**), and gaster (**D**). *Pheidole longispinosa* Forel, head of major worker (**C**), profile of minor worker (**G**).

#### Revision of the *Pheidole longispinosa* complex

**Diagnosis. *Major workers*.** Sides of the head with pilosity absent or very sparse, short, and decumbent; antennal scrobes absent or very indistinct; occipital lobes smooth and shiny or partially shagreened; genae smooth or partially superficially rugulose or shagreened; promesonotum low, short, and relatively flat; promesonotal groove absent; metanotal groove absent or distinct; propodeal spines very long; mesosoma shiny and smooth with weak, sparse, and irregular rugae on pronotum and lateral sides of propodeum or rugoreticulate, with weaker sculpture on propodeal dorsum; gaster smooth or first gastral tergite shagreened. ***Minor workers*.** Scape, when laid back, surpassing posterior head margin by more than half of its length; promesonotal groove absent; metanotal groove indistinct to distinct; mesosoma smooth, indistinctly rugulose or the whole surface finely rugoreticulate.

**Comments.** Species of this complex are characterized by a large body size, both in minor and major workers. Major workers can be distinguished by a combination of the following characters: head, in full-face view, trapezoid and widened posteriorly, in lateral view sub-oval; sides of the head with pilosity absent or very sparse and short; predominantly smooth occipital lobes; very long and massive propodeal spines and brown to black body colouration. Minor workers can be distinguished by smooth or finely rugulose head with smooth patches; long scape (surpassing posterior head margin by more than half of its length); very long and massive propodeal spines; smooth or indistinctly rugulose mesosoma, and long peduncle of petiole.

##### Pheidole longispinosa

Taxon classificationAnimaliaHymenopteraFormicidae

Forel, 1891

**Figure 23.**
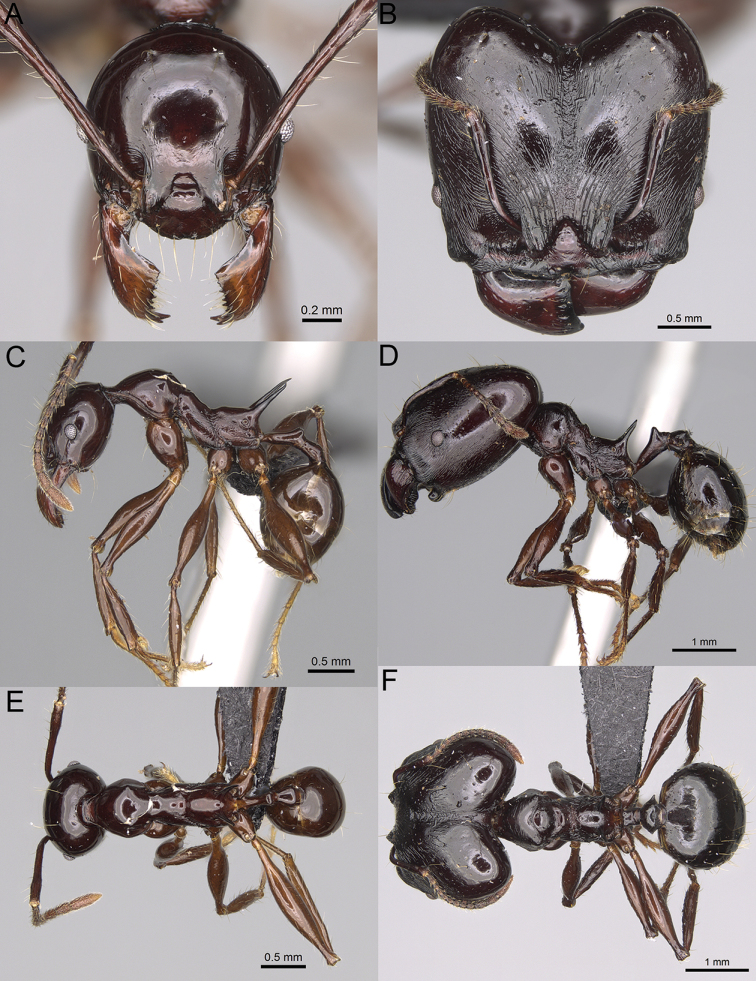
*Pheidole longispinosa* Forel, full-face view (**A**), profile (**C**), and dorsal view (**E**) of minor worker (CASENT0451282) and full-face view (**B**), profile (**D**), and dorsal view (**F**) of major worker (CASENT0451284).

Figs 23A–F , 84Y , 87C

###### Type material.

*Pheidole longispinosa* Forel, 1891: 170, pl. 5, fig. 4 (s.w.q.). Lectotype [designated here]: major worker (top specimen, CASENT0101682): Madagascar, Antananarivo, Ambatomanjaka, Miarinarivo (MHNG) [examined]. Paralectotypes: 1 major worker (CASENT0810542, bottom specimen, the same pin as lectotype) (MHNG) [examined], 3 minor workers (1 pin, CASENT0101598, CASENT0923202, CASENT0923203) (MHNG) [examined], 1 dealate queen (CASENT0101657) (MHNG) [examined]: the same data as lectotype.

###### Other material.

Madagascar. – ***Antananarivo***: •12w., 2s.; 3 km 41°NE Andranomay, 11.5 km 147°SSE Anjozorobe; -18.47333, 47.96; alt. 1300 m; 5 Dec 2000; B.L. Fisher et al. leg.; CASENT0406929, CASENT0406930, CASENT0413533, CASENT0413537, CASENT0427722, CASENT0427724, CASENT0427725, CASENT0427733. •1 w.; Ambatolaona; -18.928, 47.88283; alt. 1382 m; 19 Feb 2007; B.L. Fisher et al. leg.; CASENT0120674 (CASC). •4w., 1s, 1q.; Mandraka; -18.91813, 47.91717; alt. 1312 m; 20 Mar 2014; B.L. Fisher et al. leg.; CASENT0377078, CASENT0378205, CASENT0378163, CASENT0378568 (CASC). •4w, 1m.; Mandraka Park; -18.9019, 47.90786; 1360 m; 11 Mar 2012; B.L. Fisher et al. leg.; CASENT0275803, CASENT0275804, CASENT0275863 (CASC). •11w., 5s., 1m., 1q; Reg. Analamanga, St. Forestière Mandraka; -18.9183, 47.91687; alt. 1285 m; 25 Mar 2015; B.L. Fisher et al. leg.; CASENT0390404-CASENT0390406, CASENT0390415, CASENT0390429, CASENT0390496, CASENT0390497, CASENT0390506, CASENT0390533, CASENT0720697, CASENT0720833 (CASC); •3w., 1s.; Tsinjoarivo forest, Ankadivory; -19.71572, 47.82084; alt. 1385 m; 23 Aug 2014; B.L. Fisher et al. leg.; CASENT0389650, CASENT0389655, CASENT0389682 (CASC). – ***Antsiranana***: •1 w.; 9.2 km WSW Befingotra, Rés. Anjanaharibe-Sud; -14.75, 49.46667; alt. 1280 m; 5 Nov 1994; B.L. Fisher leg.; CASENT0198233 (CASC). •3w., 1s., 2q; Betaolana Forest, along Bekona River; -14.52996, 49.44039; alt. 880 m; 4 Mar 2009; B.L. Fisher et al. leg.; CASENT0152375, CASENT0152403, CASENT0152410 (CASC).•3w.; Parc National de Marojejy, Manantenina River, 27.6 km 35°NE Andapa, 9.6 km 327°NNW Manantenina; -14.435, 49.76; alt. 775 m; 17 Nov 2003; B.L. Fisher leg.; CASENT0487914 (CASC). – ***Fianarantsoa***: •5w., 2s.; 2 km W Andrambovato, along river Tatamaly; -21.51167, 47.41; alt. 1075 m; 3 Jun 2005; B.L. Fisher et al. leg.; CASENT0060852, CASENT0060853, CASENT0060932, CASENT0060933, CASENT0061669 (CASC). •1w.; 38 km S Ambalavao, Rés. Andringitra; -22.2, 46.96667; alt. 1680 m; 23 Oct 1993; B.L. Fisher leg.; CASENT0198397 (CASC). •3w., 1q.; 40 km S Ambalavao, Rés. Andringitra; -22.21667, 46.96667; alt. 1275 m; 19 Oct 1993; B.L. Fisher leg.; CASENT0198376, CASENT0198377 (CASC). •7w, 1s., 1q.; 8.0 km NE Ivohibe; -22.42167, 46.89833; alt. 1200 m; 3 Nov 1997; B.L. Fisher leg.; CASENT0198369, CASENT0198371, CASENT0198803, CASENT0198868 (CASC). •4w., 3s., 1q.; Parc National Befotaka-Midongy, Papango 28.5 km S Midongy-Sud, Mount Papango; -23.84083, 46.9575; alt. 1250 m; 17 Nov 2006; B.L. Fisher et al. leg.; CASENT0118416, CASENT0119618, CASENT0119592, CASENT0118442 (CASC). •7w., 1s., 1q.; R.S. Ivohibe 8.0 km E Ivohibe; -22.48333, 46.96833; alt. 1200 m; 15 Oct 1997; B.L. Fisher leg.; CASENT0196901, CASENT0198003, CASENT0198004, CASENT0198372, CASENT0198805, CASENT0198807, CASENT0198867 (CASC). – ***Toamasina***: •1w.; [Morano-Chrome forêt, 25 km W]; Morarano Chrome; Amparafaravola; -17.75, 47.98333; alt. 1276 m; 15 Jun 1991; A. Pauly leg.; CASENT0198378 (CASC). •1w., 1s., 1q.; 6.9 km NE Ambanizana, Ambohitsitondroina; -15.56667, 50; alt. 1000 m; 9 Dec 1993; B.L. Fisher leg.; CASENT0198379 (CASC). •1w.; Ambanizana, Parc National Masoala; -15.57167, 50.00611; alt. 800–897 m; 26 Feb 2003; Andriamalala D. et al. leg.; CASENT0047728 (CASC). •8w.; Ankerana; -18.40636, 48.80254; alt. 1108 m; 19 Jan 2012; B.L. Fisher et al. leg.; CASENT0273728, CASENT0274171, CASENT0274172, CASENT0274173, CASENT0274993, CASENT0274994, CASENT0274994 (CASC). •1w., 1s.; Corridor Forestier Analamay-Mantadia, Tsaravoniana; -18.76124, 48.42134; alt. 939 m; 2 Dec 2012; B.L. Fisher et al. leg.; CASENT0300341 (CASC). •3w., 2s.; F.C. Didy; -18.19833, 48.57833; alt. 960 m; 16 Dec 1998; Ratsirarson H.J. leg.; CASENT0198005, CASENT0198006, CASENT0198373 (CASC). •2w., 1s.; Montagne d’Anjanaharibe, 19.5 km 27°NNE Ambinanitelo; -15.17833, 49.635; alt. 1100 m; 12 Mar 2003; B.L. Fisher et al. leg.; CASENT0037455, CASENT0037462, CASENT0048698, CASENT0048727 (CASC). •1w.; P.N. Mantadia; -18.79167, 48.42667; alt. 895 m; 25 Nov 1998; Ratsirarson H.J. leg.; CASENT0198384 (CASC). •1w., 1s.; Parc National d´ Andasibe-Mantadia, Forêt de Mantadia, 25.7 km 248° Moramanga; -18.81402, 48.43028; alt. 1040 m; 13 Jul 2006; Raharimalala & Blaimer leg.; CASENT0117442 (CASC). •1w., 1s.; Parc National de Zahamena, Onibe River; -17.75908, 48.85468; alt. 780 m; 21 Feb 2009; B.L. Fisher et al. leg.; CASENT0152053 (CASC). – ***Toliara***: •7w., 1s., 1q.; 13 km NW Enkara, Rés Andohahela; -24.55, 46.8; alt. 1300 m; 2 Dec 1992; B.L. Fisher leg.; CASENT0198374, CASENT0198375, CASENT0198381, CASENT0198382 (CASC). •31w., 14s., 9q.; Anosy Region, Anosyenne Mts, 29.33 km NW Manantenina; -24.13993, 47.07418; alt. 540 m; 21 Feb 2015; B.L. Fisher et al. leg.; CASENT0704727, CASENT0704727, CASENT0704749, CASENT0704780, CASENT0723465, CASENT0704473, CASENT0704200, CASENT0704201, CASENT0704205, CASENT0704206, CASENT0704217, CASENT0704218, CASENT0704226, CASENT0704227, CASENT0704228, CASENT0704229, CASENT0704242, CASENT0704245, CASENT0704273, CASENT0704353, CASENT0704354, CASENT0704355, CASENT0704393, CASENT0704394, CASENT0704867, CASENT0704874, CASENT0704886, CASENT0704887, CASENT0705875, CASENT0705877, CASENT0721001, CASENT0721005, CASENT0721007, CASENT0721009, CASENT0721013 (CASC). •20w., 2s.; Parc National d’Andohahela, Col du Sedro, 3.8 km 113°ESE Mahamavo, 37.6 km 341°NNW Tolagnaro; -24.76389, 46.75167; alt. 900 m; 21 Jan 2002; B.L. Fisher et al. leg.; CASENT0078380, CASENT0430695, CASENT0430759, CASENT0451281, CASENT0451303, CASENT0451309, CASENT0456249, CASENT0460156, CASENT0460158, CASENT0483964, CASENT0484129, CASENT0484130 (CASC). •3w.; Parc National de Kirindy Mite, 16.3 km 127°SE Belo sur Mer; -20.79528, 44.147; alt. 80 m; 6 Dec 2001; B.L. Fisher et al. leg.; CASENT0477205 (CASC). •5w. 2s.,1q.; Réserve Spéciale Kalambatritra, Ambinanitelo; -23.4502, 46.45658; alt. 1325 m; 11 Feb 2009; B.L. Fisher et al. leg.; CASENT0148863, CASENT0148866, CASENT0149937–CASENT0149939 (CASC). •18w., 7s., 3m., 11q.; Réserve Spéciale Kalambatritra, Ampanihy; -23.463, 46.47057; alt. 1269 m; 10 Feb 2009; B.L. Fisher et al. leg.; CASENT0148871, CASENT0148872, CASENT0148875, CASENT0148945, CASENT0148953, CASENT0148964, CASENT0148967, CASENT0148972, CASENT0148983, CASENT0148994, CASENT0148995, CASENT0148996, CASENT0149001, CASENT0149008, CASENT0149801, CASENT0149992, CASENT0150511, CASENT0150535, CASENT0151012, CASENT0151013, CASENT0151019, CASENT0151020, CASENT0152223, CASENT0153227, CASENT0235023 (CASC). •6w., 3s., 2q.; Réserve Spéciale Kalambatritra, Befarara; -23.4178, 46.4478; alt. 1390 m; 7 Feb 2009; B.L. Fisher et al. leg.; CASENT0149598, CASENT0149599, CASENT0149869, CASENT0149871, CASENT0150723 (CASC). •1w., 1s.; Réserve Spéciale Kalambatritra, Betanana; -23.4144, 46.459; alt. 1360 m; 8 Feb 2009; B.L. Fisher et al. leg.; CASENT0148654 (CASC).

###### Diagnosis.

***Major workers*.** Large species: HL: 2.62–3.0 (2.77), HW: 2.64–2.92 (2.75), WL: 1.88–2.14 (2.04); propodeal spines very long (PSL: 0.67–0.87 (0.73)); head in full-face view trapezoid, widened posteriorly; sides of the head without pilosity; frons with longitudinal rugae never reaching further than midlength of head; gaster smooth and shiny; body brown to dark brown. ***Minor workers*.** Large species: HL: 0.99–1.15 (1.1), HW: 0.98–1.06 (1.0), WL: 1.62–1.76 (1.66); propodeal spines very long (PSL: 0.62–0.76 (0.69)); scape, when laid back, surpassing posterior head margin by more than half its length; mesosoma smooth and shiny, only anepisternum, katepisternum, and propodeum sometimes with indistinct and sparse superficial rugulae; promesonotal groove absent; metanotal groove shallow and wide.

###### Redescription.

**Major workers.** Measurements (*N* = 10): HL: 2.62–3.0 (2.77); HW: 2.64–2.92 (2.75); SL: 1.24–1.35 (1.3); EL: 0.24–0.28 (0.26); WL: 1.88–2.14 (2.04); PSL: 0.67–0.87 (0.73); MTL: 1.39–1.55 (1.46); PNW: 0.8–0.97 (0.89); PTW: 0.23–0.31 (0.27); PPW: 0.68–0.82 (0.75); CI: 94.3–106.4 (99.3); SI: 43.9–50.9 (47.4); PSLI: 24.1–29.0 (26.5); PPI: 30.1–42.3 (35.4); PNI: 30.45–33.38 (32.4); MTI: 49.6–57.6 (53.0). ***Head*.** In full-face view trapezoid, widened posteriorly (Fig. 23B). In lateral view sub-oval; ventral and dorsal faces convex; inner hypostomal teeth visible. Sides of the head without pilosity; frons and vertex with few, long, erect setae. Antennal scrobes absent or very indistinct. Occipital lobes, genae, posterior part of frons smooth and shiny; lateral sides of head and genae with sparse, partially fading superficial rugulae; centre of frons shiny with fine, thin, longitudinal rugae reaching at most to midlength of head; malar area with fine, thin, longitudinal rugae. Centre of clypeus smooth and shiny, lateral sides with fine and sparse rugae; median notch present, shallow, and wide; median longitudinal carina absent; lateral longitudinal carinae absent. Scape, when laid back, reaching midlength of head; pilosity suberect to erect (Fig. 23B, D). Inner hypostomal teeth distinct, low, triangular and thick, with rounded apex, closely spaced; outer hypostomal teeth distinct, slightly smaller and thinner than inner hypostomal teeth, lobe-like (Fig. 84Y). ***Mesosoma*.** In lateral view, promesonotum low, short, and relatively flat; pronotum slightly convex; dorsal mesonotum concave; posterior mesonotum relatively steep; promesonotal groove absent; metanotal groove absent; propodeal spines very long, massive basally, with acute apex; humeral area laterally either weakly or not produced (Fig. 23D). Surface shiny and smooth with weak, sparse, and irregular rugae on pronotum and lateral sides of propodeum. Pilosity very sparse, short, and decumbent; dorsum with few additional, long, erect setae (Fig. 23D, F). ***Petiole*.** Smooth and shiny; peduncle long and thin, superficially rugulose ventrally; node triangular, with rounded apex, in rear view node dorsoventrally depressed; pilosity sparse, short, and erect (Fig. 23D, F). ***Postpetiole*.** Smooth and shiny; in dorsal view sides with acute, narrow, moderately short triangular projections; pilosity short to long, sparse, and erect (Fig. 23D, F). ***Gaster*.** Smooth and shiny; pilosity sparse, erect, and long (Fig. 23D, F). ***Colour*.** Unicolourous, brown to dark brown (Fig. 23D, F).

**Minor workers.** Measurements (*N* = 10): HL: 0.99–1.15 (1.1); HW: 0.98–1.06 (1.0); SL: 1.35–1.47 (1.4); EL: 0.14–0.21 (0.18); WL: 1.62–1.76 (1.66); PSL: 0.62–0.76 (0.69); MTL: 1.21–1.29 (1.24); PNW: 0.65–0.7 (0.67); PTW: 0.13–0.17 (0.16); PPW: 0.22–0.27 (0.25); CI: 88.3–99.1 (91.3); SI: 136.7–144.4 (139.7); PSLI: 56.8–67.8 (62.5); PPI: 56.3–66.9 (62.9); PNI: 65.6–68.3 (66.9); MTI: 121.3–128.1 (124.4). ***Head*.** Occipital margin convex; occipital carina narrow, weakly developed (Fig. 23A). Pilosity sparse, long, and erect on frons and median part of occiput. Sculpture smooth and shiny on the whole surface, only antennal sockets with sparse carinae curved outward. Clypeus with median longitudinal carina absent; two lateral longitudinal carinae present. Scape, when laid back, surpassing posterior head margin by more than half of its length; pilosity suberect to erect (Fig. 23A, C). ***Mesosoma*.** In lateral view, promesonotum low, long, and slightly convex; promesonotal groove absent; metanotal groove indistinct and wide; propodeal spines very long, massive basally, with acute apex (Fig. 23C). Surface smooth and shiny, only anepisternum, katepisternum, and propodeum sometimes with indistinct and sparse superficial rugulae. Pilosity absent or short, very sparse, suberect (Fig. 23C, E). ***Petiole*.** Smooth and shiny; peduncle long and thin; node bulge-like with rounded apex; pilosity absent (Fig. 23C, E). ***Postpetiole*.** Moderately long, low, and with slightly convex dorsum; with two long, erect setae at the anterior edge (Fig. 23C, E). ***Gaster*.** With few long, erect setae (Fig. 23C, E). ***Colour*.** Unicolourous, brown to dark brown (Fig. 23C, E).

###### Biology.

The species was collected at between 80–1680 m in elevation, in montane rainforest, montane shrubland, thicket, tropical dry forest, and urban areas. Nests were located in rotten logs, tree stumps, soil, and rock crevasses.

###### Comments.

This species is most similar to *P. mahaboensis* sp. nov. and *P. praegrandis* sp. nov. ***Major workers*.***Pheidole longispinosa* can be distinguished from *P. mahaboensis* sp. nov. by absence of metanotal groove, lack of pilosity at the sides of head, and longitudinal rugae never reaching further than midlength of head; from *P. praegrandis* sp. nov. by brighter body colouration, reduced head sculpture, and not shagreened first gastral tergite and head. ***Minor workers*.***Pheidole longispinosa* can be distinguished from *P. mahaboensis* sp. nov. by absence of promesonotal groove, shallow and wide metanotal groove and reduced sculpture on mesosoma; from *P. praegrandis* sp. nov. by mostly smooth and shiny sculpture of head and mesosoma, which is never rugoreticulate.

##### Pheidole mahaboensis sp. nov.

Taxon classificationAnimaliaHymenopteraFormicidae

http://zoobank.org/85A50CF5-C746-4EA8-854B-6FE694B8F7F7

**Figure 24.**
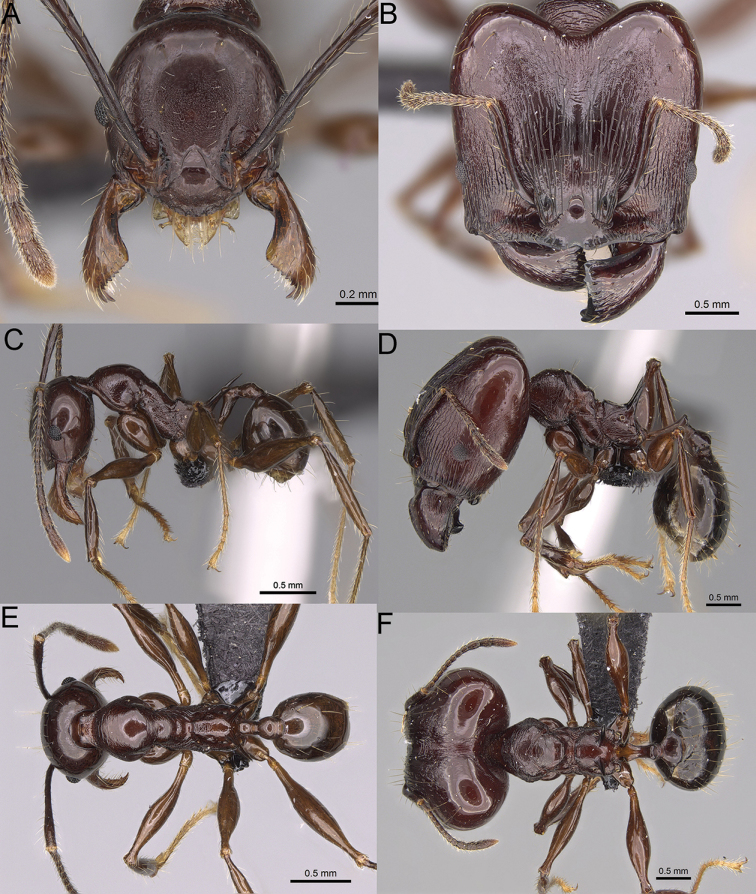
*Pheidole mahaboensis* sp. nov., full-face view (**A**), profile (**C**), and dorsal view (**E**) of paratype minor worker (CASENT0070926) and full-face view (**B**), profile (**D**), and dorsal view (**F**) of holotype major worker (CASENT0070925).

Figs 24A–F , 85B , 87F

###### Type material.

***Holotype*.** Madagascar. •1 major worker; Fianarantsoa; Réserve Forestière d’Agnalazaha, Mahabo, 42.9 km 215° Farafangana; -23.19383, 47.723; alt. 20 m; 19 Apr 2006; B.L. Fisher et al. leg.; BLF13900, CASENT0070925 (CASC). ***Paratypes*.** Madagascar. •2w. 1m.; same data as for holotype; CASENT0872077, CASENT0070926, CASENT0872197 (CASC).

###### Other material.

Madagascar. – ***Antsiranana***: •7w., 4s, 2q., 1m; Masoala National Park; -15.3014, 50.22776; alt. 280 m; 7 Mar 2014; B.L. Fisher et al. leg.; CASENT0376080, CASENT0376081, CASENT0377018, CASENT0377035, CASENT0377036, CASENT0377038, CASENT0377568 (CASC). – ***Fianarantsoa***: •1w., 1s.; 2 km W Andrambovato, along river Tatamaly; -21.51167, 47.41; alt. 1075 m; 3 Jun 2005; B.L. Fisher et al. leg.; CASENT0060947 (CASC). •4w., 2q; 40 km S Ambalavao, Rés. Andringitra; -22.21667, 46.96667; alt. 1275 m; 19 Oct 1993; B.L. Fisher leg.; CASENT0198822, CASENT0198823, CASENT0198815, CASENT0198831 (CASC). •5w., 3s., 2q.; 45 km S Ambalavao; -22.21667, 47.01667; alt. 720 m; 26 Sep 1993; B.L. Fisher leg.; CASENT0198830, CASENT0198832, CASENT0198824, CASENT0198829 (CASC). •14w., 7s., 1q.; 7.6 km 122° Kianjavato, Forêt Classée Vatovavy; -21.4, 47.94; alt. 175 m; 6 Jun 2005; B.L. Fisher et al. leg.; CASENT0059894, CASENT0059895, CASENT0059940, CASENT0059941, CASENT0059944, CASENT0060019, CASENT0060020, CASENT0060166, CASENT0060167, CASENT0060298, CASENT0060495, CASENT0061087, CASENT0061148, CASENT0061233 (CASC). •10w., 1s.; 8.0 km NE Ivohibe; -22.42167, 46.89833; alt. 1200 m; 3 Nov 1997; B.L. Fisher et al. leg.; CASENT0198821, CASENT0009788, CASENT0198231, CASENT0198806, CASENT0198825, CASENT0198871, CASENT0198874 (CASC). •2w., 1s.; Belle Vue trail, Ranomafana National Park, Fianarantsoa Prov.; -21.2665, 47.42017; alt. 1020 m; 23 May 2002; Harin’Hala leg.; CASENT0079551, CASENT0080544, CASENT0080545, CASENT0080546, CASENT0112227, CASENT0112304, CASENT0112699, CASENT0112768, CASENT0112778, CASENT0113028, CASENT0113154, CASENT0113191, CASENT0113192, CASENT0113227, CASENT0113325, CASENT0113341, CASENT0113369, CASENT0113399, CASENT0113408, CASENT0113411, CASENT0113816, CASENT0113817, CASENT0114099, CASENT0114211, CASENT0114327, CASENT0114751, CASENT0114767 (CASC). •4w.; Fitovavy Fitovinany Region, District of Ifanadiana Belle vue area1200 m S of Ranomafana National Park entrance; -21.2665, 47.42017; alt. 1018 m; 28 May 2003; Rin’ha leg.; CASENT0114066, CASENT0114710, CASENT0112925, CASENT0113635 (CASC). •8w., 6s.; Forêt de Vevembe, 66.6 km 293° Farafangana; -22.791, 47.18183; alt. 600 m; 24 Apr 2006; B.L. Fisher et al. leg.; CASENT0070669, CASENT0070706, CASENT0070718, CASENT0070740, CASENT0070753, CASENT0071145, CASENT0108002, CASENT0108034 (CASC). •1w.; Ifanadiana Pref: Ranomafana S.-Pref: Ranomafana, Talatakely site near R. Namorona; -21.26806, 47.4247; alt. 967 m; 5 Dec 2004; Lees et al. leg.; CASENT0056933 (CASC). •3w.; JIRAMA water works near river, Ranomafana National Park; -21.2485, 47.45217; alt. 690 m, 28 Jan 2002; Harin’Hala leg.; CASENT0114140, CASENT0114293, CASENT0114295 (CASC). •12w., 6s., 1q.; Parc National Befotaka-Midongy, Papango 27.7 km S Midongy-Sud, Mount Papango; -23.83517, 46.96367, alt. 940 m, 14 Nov 2006; B.L. Fisher et al. leg.; CASENT0119097, CASENT0119108, CASENT0119110, CASENT0119111, CASENT0119113, CASENT0119121, CASENT0119424, CASENT0119441, CASENT0125642, CASENT0128545, CASENT0128694, CASENT0128699 (CASC). •1w.; Parc National Befotaka-Midongy, Papango 28.5 km S Midongy-Sud, Mount Papango; -23.84083, 46.9575; alt. 1250 m; 17 Nov 2006; B.L. Fisher et al. leg.; CASENT0128569 (CASC). •129w., 19s., 1q.; Parc National de Ranomafana, Vatoharanana River, 4.1 km 231°SW Ranomafana; -21.29, 47.43333; alt. 1100 m; 27 Mar 2003; Fisher et al. leg.; CASENT0039679, CASENT0039915, CASENT0039916, CASENT0039920, CASENT0039923, CASENT0039930, CASENT0039941, CASENT0039951, CASENT0039954, CASENT0039959, CASENT0039963, CASENT0039967, CASENT0039967, CASENT0039970, CASENT0039978, CASENT0040051, CASENT0040056, CASENT0040058, CASENT0040075, CASENT0040079, CASENT0040084, CASENT0040181, CASENT0040200, CASENT0040215, CASENT0040232, CASENT0040235, CASENT0040238, CASENT0040240, CASENT0040244, CASENT0040251, CASENT0040253, CASENT0040264, CASENT0040270, CASENT0040326, CASENT0040331, CASENT0040336, CASENT0040337, CASENT0040344, CASENT0040350, CASENT0040360, CASENT0040415, CASENT0040421, CASENT0049613, CASENT0050290, CASENT0073552, CASENT0488608, CASENT0488609, CASENT0488627, CASENT0488640, CASENT0488641, CASENT0488642, CASENT0488643, CASENT0488659, CASENT0488662, CASENT0488677, CASENT0488678, CASENT0488679, CASENT0488680, CASENT0497159, CASENT0497161, CASENT0497211–CASENT0497216, CASENT0497327–CASENT0497333, CASENT0497372–CASENT0497377, CASENT0497412–CASENT0497416, CASENT0497428, CASENT0497429, CASENT0497595–CASENT0497598, CASENT0497643–CASENT0497646, CASENT0497685 (CASC). •11w., 1s.; Parc Nationale Ranomafana: Talatakely; -21.24833, 47.42667; 9 Apr 1998; Griswold et al. leg.; CASENT0096608, CASENT0096609, CASENT0096712, CASENT0096713, CASENT0096714, CASENT0096765, CASENT0096770, CASENT0096771, CASENT0096772, CASENT0096774, CASENT0096775, CASENT0097967 (CASC). •3w., 4s.; R.S. Ivohibe 8.0 km E Ivohibe; -22.48333, 46.96833; alt. 1200 m; 15 Oct 1997; B.L. Fisher et al. leg.; CASENT0198804, CASENT0198872 (CASC). •2w., 1s.; R.S. Ivohibe, 7.5 km ENE Ivohibe; -22.47,46.96; alt. 900 m; 7 Oct 1997; B.L. Fisher et al. leg.; CASENT0198232, CASENT0198802, CASENT0198869 (CASC). •21w., 1s.; radio tower, Ranomafana National Park; -21.25833, 47.40717; alt. 1130 m; 14 Jun 2002; Irwin & Harin’Hala leg.; CASENT0052853, CASENT0053113, CASENT0053232, CASENT0053238, CASENT0078772, CASENT0079894, CASENT0080504, CASENT0111901, CASENT0112824, CASENT0112862, CASENT0113134, CASENT0113293, CASENT0113643, CASENT0113751, CASENT0114008, CASENT0114062, CASENT0114517, CASENT0114536, CASENT0114537, CASENT0114642, CASENT0114661 (CASC). •3w.; Ranomafana; -21.25, 47.36667; 1 Mar 1994; A. Pauly leg.; CASENT0096206-CASENT0096208 (CASC). •2w.; Ranomafana National Park, Talatakely area, 0.4 km WSW of Park Entrance; -21.41667, 47.68333; alt. 900 m; 2 Jan 2001; Kavanaugh leg.; CASENT0007643, CASENT0007644 (CASC). •3w.; Ranomafana National Park, Talatakely; Sahambavy; Fianarantsoa Rural; -21.451179, 47.3023894; alt. 1139 m; 30 Oct 1998; Lee & Ribardo leg.; CASENT0198826 (CASC). •1w.; research cabin at Talatakely, Ranomafana National Park; -21.25041, 47.41945; alt. 900 m; 11 Apr 1998; Irwin & Schlinger leg.; CASENT0198817 (CASC). •9w., 4s., 2q.; Réserve Forestière d’Agnalazaha, Mahabo, 42.9 km 215° Farafangana; -23.19383, 47.723; alt. 20 m; 19 Apr 2006; B.L. Fisher et al. leg.; CASENT0070855–CASENT0070858, CASENT0070976, CASENT0070977, CASENT0071584, CASENT0072846, CASENT0072853 (CASC). •17w., 8s., 1q.; Réserve Spéciale Manombo 24.5 km 228° Farafangana; -23.01583, 47.719; alt. 30 m; 21 Apr 2006; B.L. Fisher et al. leg.; CASENT0071522–CASENT0071525, CASENT0071528, CASENT0071547, CASENT0071605, CASENT0071606, CASENT0072089, CASENT0072093, CASENT0072098, CASENT0073134, CASENT0073136, CASENT0073142, CASENT0073153, CASENT0073157, CASENT0108512, CASENT0108514, CASENT0108519, CASENT0108527 (CASC). •13w., 1s.; Vohiparara broken bridge; -21.22617, 47.36983; alt. 1110 m; 25 Jul 2002; Harin’Hala leg.; CASENT0052215, CASENT0078742, CASENT0079658, CASENT0079845, CASENT0111141, CASENT0111855, CASENT0111955, CASENT0111993, CASENT0112046, CASENT0112070, CASENT0112157, CASENT0112176, CASENT0112606, CASENT0113065 (CASC). – ***Toamasina***: •2w., 1s.; 6.2 km SSE Ambanizana, Be Dinta; -15.66667, 49.99806; alt. 600 m; 20 Nov 1993; B.L. Fisher leg.; CASENT0198816 (CASC). •2w., 1s., 1q.; Analalava, 7.0 km 255° Mahavelona; -17.7095, 49.454; alt. 50 m; 27 Nov 2005; B.L. Fisher et al. leg.; CASENT0067391, CASENT0067392 (CASC). •1w.; F.C. Andriantantely; -18.695, 48.81333; alt. 530 m; 4 Dec 1998; Ratsirarson leg.; CASENT0198230 (CASC). •2w.; F.C. Sandranantitra; -18.04833, 49.09167; alt. 450 m; 21 Jan 1999; Ratsirarson leg.; CASENT0198808 (CASC). •2w., 2s.; Mahavelona (Foulpointe); -17.66667, 49.5; 25 Dec 1993; A. Pauly leg.; CASENT0096124, CASENT0096181, CASENT0096365, CASENT0096366 (CASC). •1w., 1s., 1 q.; Manakambahiny, near Vavatenina Forest; -17.46667, 49.35; 9 Feb 1995; A. Pauly leg.; CASENT0094829, CASENT0095095, CASENT0095102 (CASC). •3q.; Montagne d’Anjanaharibe, 18.0 km 21°NNE Ambinanitelo; -15.18833, 49.615; alt. 470 m; 8 Mar 2003; Fisher et al. leg.; CASENT0495177 (CASC). •1w.; Parc National de Zahamena, Onibe River; -17.75908, 48.85468; alt. 780 m; 21 Feb 2009; B.L. Fisher et al. leg.; CASENT0152016 (CASC). •6w., 4s.; Parc National Mananara-Nord, 7.1 km 261° Antanambe; -16.455, 49.7875; alt. 225 m; 15 Nov 2005; B.L. Fisher et al. leg.; CASENT0067356, CASENT0067462, CASENT0067464, CASENT0067473, CASENT0067601, CASENT0071327 (CASC). •4w., 2s.; Reserve Betampona, Camp Rendrirendry 34.1 km 332° Toamasina; -17.924, 49.19967; alt. 390 m; 30 Nov 2005; B.L. Fisher et al. leg.; CASENT0067806, CASENT0068019, CASENT0071898, CASENT0072529 (CASC). •40w., 7s., 5q.; Réserve Spéciale Ambatovaky, Sandrangato River; -16.81745, 49.2925; alt. 400 m; 26 Feb 2010 B.L. Fisher et al. leg.; CASENT0160439, CASENT0160440, CASENT0161071, CASENT0161072, CASENT0161084, CASENT0161085, CASENT0161474, CASENT0161475, CASENT0161806-CASENT0161808; CASENT0161881, CASENT0161953, CASENT0161954, CASENT0162120, CASENT0162121, CASENT0162670, CASENT0163018, CASENT0163071, CASENT0163072, CASENT0163114, CASENT0163138, CASENT0163160, CASENT0163552, CASENT0163614, CASENT0163667, CASENT0163871, CASENT0163941, CASENT0163985, CASENT0164288, CASENT0164294, CASENT0164299, CASENT0164304, CASENT0164319, CASENT0164433, CASENT0164441, CASENT0164456, CASENT0164469, CASENT0164487, CASENT0164496 (CASC). – ***Toliara***: •1w., 1s.; 10 km NW Enakara, Rés. Andohahela; -24.56667, 46.81667; alt. 430 m; 22 Nov 1992; B.L. Fisher leg.; CASENT0198809 (CASC). •4w., 1s.; 10 km NW Enakara, Rés. Andohahela; -24.56667, 46.81667; alt. 425 m; 24 Nov 1992; B.L. Fisher leg.; CASENT0198811, CASENT0709102 (CASC). •4w., 1s.; 11 km NW Enakara, Rés. Andohahela; -24.56667, 46.83333; alt. 800 m; 20 Nov 1992; B.L. Fisher leg.; CASENT0198812, CASENT0198813 (CASC). •2w.; 13 km NW Enakara, Rés. Andohahela; -24.55, 46.8; alt. 1250 m; 30 Nov 1992; B.L. Fisher leg.; CASENT0198380, CASENT0198814 (CASC). •6w., 2s., 1q.; 2.7 km WNW 302° Ste. Luce; -24.77167, 47.17167; alt. 20 m; 9 Dec 1998; B.L. Fisher et al. leg.; CASENT0198818–CASENT0198820, CASENT0198870 (CASC). •12w., 4s., 2m.; Anosy Region, Anosyenne Mts, 29.33 km NW Manantenina; -24.13993, 47.07418; alt. 540 m; 21 Feb 2015; B.L. Fisher et al. leg.; CASENT0704710, CASENT0705814, CASENT0723419, CASENT0723782, CASENT0723788, CASENT0723798, CASENT0723801, CASENT0723811, CASENT0723833, CASENT0704455, CASENT0704456, CASENT0704499, CASENT0704520 (CASC). •2w., 1s., 1m.; Anosy Region, Parc National Andohahela, Col de Tanatana; -24.7585, 46.85367; alt. 275 m; 7 Mar 2015; B.L. Fisher et al. leg.; CASENT0724259, CASENT0724260 (CASC). •5w., 1s.; Cul du Marosohy; -24.55, 46.83333; alt. 600 m; 14 Nov 1992; B.L. Fisher leg.; CASENT0198810, CASENT0198827 (CASC). •8w., 6s., 1q.; Forêt Ivohibe 55.0 km N Tolagnaro; -24.569, 47.204; alt. 200 m; 3 Dec 2006; B.L. Fisher et al. leg.; CASENT0122618, CASENT0121993, CASENT0122577, CASENT0122635, CASENT0122790, CASENT0122792, CASENT0122793, CASENT0122800 (CASC). •4w., 4s.; Grand Lavasoa, 25.9 km W Tolagnaro; -25.08767, 46.749; alt. 450 m; 30 Nov 2006; B.L. Fisher et al. leg.; CASENT0122875, CASENT0122896, CASENT0122907, CASENT0122908 (CASC). •1w., 1q.; Manatantely, 8.9 km NW Tolagnaro; -24.9815, 46.92567; alt. 100 m; 27 Nov 2006; B.L. Fisher et al. leg.; CASENT0125791, CASENT0125793 (CASC). •3w., 2s.; Parc National Andohahela, Col de Tanatana, 33.3 km NW Tolagnaro; -24.7585, 46.85367; alt. 275 m; 22 Nov 2006; B.L. Fisher et al. leg. CASENT0121898, CASENT0121900, CASENT0121917 (CASC).

###### Diagnosis.

***Major workers*.** Large species: HL: 2.38–2.71 (2.56), HW: 2.34–2.61 (2.48), WL: 1.78–1.96 (1.83); propodeal spines very long (PSL: 0.64–0.75 (0.7)); head in full-face view trapezoid, widened posteriorly; sides of the head with very sparse, short, decumbent pilosity; frons with longitudinal rugae reaching further than midlength of head; gaster smooth and shiny; body reddish brown to dark brown. ***Minor workers*.** Large species: 0.93–1.08 (1.0), HW: 0.79–0.93 (0.88), WL: 1.2–1.5 (1.4); propodeal spines very long (PSL: 0.53–0.66 (0.61)); scape, when laid back, surpassing posterior head margin by more than half its length; pronotum and mesonotal dorsum shiny, smooth or with indistinct, sparse, superficial rugulae; anepisternum, katepisternum and propodeum shiny, with slightly denser superficial rugulae; promesonotal groove present; metanotal groove narrow and relatively deep.

###### Description.

**Major workers.** Measurements (*N* = 10): HL: 2.38–2.71 (2.56); HW: 2.34–2.61 (2.48); SL: 1.1–1.24 (1.16); EL: 0.22–0.28 (0.25); WL: 1.78–1.96 (1.83); PSL: 0.64–0.75 (0.7); MTL: 1.21–1.32 (1.27); PNW: 0.74–0.86 (0.8); PTW: 0.21–0.26 (0.24); PPW: 0.73–0.83 (0.77); CI: 93.0–101.0 (96.9); SI: 44.3–49.7 (46.6); PSLI: 25.5–29.6 (27.3); PPI: 27.8–33.7 (30.9); PNI: 30.1–35.6 (32.4); MTI: 49.8–53.5 (51.3). ***Head*.** In full-face view trapezoid, widened posteriorly (Fig. 24B). In lateral view sub-oval; ventral and dorsal faces convex; inner hypostomal teeth visible. Sides of the head with very sparse, short, decumbent pilosity; frons and vertex with few additional, long, erect setae. Antennal scrobes absent or very indistinct. Occipital lobes smooth and shiny; genae smooth and shiny or dull, superficially rugulose with median shiny patch widening posteriorly; centre of frons shiny with longitudinal rugae reaching at least midlength of head, sometimes surface between rugae with superficial rugulae; malar area and lateral sides of head superficially rugulose, sometimes with several short, longitudinal rugae; posterior part of frons smooth and shiny or superficially rugulose; head sculpture weakens posteriorly. Centre of clypeus smooth and shiny, lateral sides superficially rugulose; median notch present, shallow, and wide; median longitudinal carina absent; lateral longitudinal carinae absent. Scape, when laid back, reaching midlength of head; pilosity decumbent to subdecumbent (Fig. 24B, D). Inner hypostomal teeth distinct, low, triangular, and thick, with rounded apex, closely spaced; outer hypostomal teeth distinct, slightly smaller and thinner than inner hypostomal teeth, lobe-like (Fig. 85B). ***Mesosoma*.** In lateral view, promesonotum low, short, and relatively flat; pronotum slightly convex; mesonotum slightly concave; posterior mesonotum relatively steep; promesonotal groove absent; metanotal groove narrow and relatively deep; propodeal spines very long, massive basally, with acute apex; humeral area laterally weakly or not produced (Fig. 24D). Surface shiny and smooth with weak, sparse, and irregular rugae on pronotum, lateral sides of propodeum, katepisternum, and anepisternum. Pilosity very sparse, short, and decumbent; dorsum with few additional, long erect setae (Fig. 24D, F). ***Petiole*.** Smooth and shiny; peduncle long and thin, superficially rugulose ventrally; node triangular with rounded apex, in rear view node dorsoventrally depressed; pilosity sparse, short, and erect (Fig. 24D, F). ***Postpetiole*.** Smooth and shiny; in dorsal view sides with acute, narrow, triangular projections; pilosity short to long, sparse, and erect (Fig. 24D, F). ***Gaster*.** Pilosity sparse, long, and erect (Fig. 24D, F). ***Colour*.** Unicolourous, reddish brown to dark brown (Fig. 24D, F).

**Minor workers.** Measurements (*N* = 10): HL: 0.93–1.08 (1.0); HW: 0.79–0.93 (0.88); SL: 1.16–1.32 (1.26); EL: 0.13–0.19 (0.16); WL: 1.2–1.5 (1.4); PSL: 0.53–0.66 (0.61); MTL: 0.96–1.11 (1.06); PNW: 0.53–0.68 (0.6); PTW: 0.12–0.13 (0.13); PPW: 0.19–0.22 (0.21); CI: 85.0–90.1 (87.4); SI: 141.5–146.5 (144.1); PSLI: 57.3–63.2 (60.5); PPI: 55.2–66.8 (61.8); PNI: 62.7–73.1 (68.3); MTI: 116.8–126.5 (120.8). ***Head*.** Occipital margin convex; occipital carina narrow, weakly developed (Fig. 24A). Pilosity sparse, short, and appressed on the whole surface and additionally long and erect on frons and median part of occiput. Sculpture smooth and shiny on the whole surface, only antennal sockets with sparse carinae curved outward. Clypeus smooth and shiny; its anterior margin regularly convex, with weakly developed teeth; median longitudinal carina absent; two lateral longitudinal carinae present. Scape, when laid back, surpassing posterior head margin by more than half of its length; pilosity suberect to erect (Fig. 24A, C). ***Mesosoma*.** In lateral view, promesonotum low, long, and slightly convex; promesonotal groove indistinct; metanotal groove narrow and relatively deep; propodeal spines very long, massive basally, with acute apex (Fig. 24C). Pronotum and mesonotal dorsum shiny, smooth or with indistinct, sparse, superficial rugulae; anepisternum, katepisternum, and propodeum shiny, with slightly denser superficial rugulae. Pilosity short, very sparse, suberect (Fig. 24C, E). ***Petiole*.** Peduncle long and thin; node bulge-like with rounded apex; pilosity absent (Fig. 24C, E). ***Postpetiole*.** Moderately long, low, and slightly convex; with two long, erect setae at the anterior edge (Fig. 24C, E). ***Gaster*.** With few long, erect setae (Fig. 24C, E). ***Colour*.** Unicolourous, brown to dark brown (Fig. 24C, E).

###### Etymology.

From the type locality.

###### Biology.

The species was collected at between 20–1275 m in elevation, in rainforest and once in open area near stream. Nests were located in rotten logs and tree stumps.

###### Comments.

This species is most similar to *P. longispinosa* and *P. praegrandis* sp. nov. ***Major workers*.***Pheidole mahaboensis* sp. nov. can be distinguished from *P. longispinosa* by presence of metanotal groove, presence of very sparse, short, decumbent pilosity at the sides of head, and longitudinal rugae reaching at least midlength of head; from *P. praegrandis* sp. nov. by presence of metanotal groove, presence of very sparse, short, decumbent pilosity at the sides of head, and not shagreened first gastral tergite and head. ***Minor workers*.***Pheidole mahaboensis* sp. nov. can be distinguished from *P. longispinosa* by presence of promesonotal groove, narrow and relatively deep metanotal groove, and presence of fine superficial rugulae on most of mesosoma; from *P. praegrandis* sp. nov. by mostly smooth and shiny sculpture of head and mesosoma, which is never rugoreticulate.

##### Pheidole praegrandis sp. nov.

Taxon classificationAnimaliaHymenopteraFormicidae

http://zoobank.org/F27040F5-CB05-40AD-B4E5-A7532D3FCAF1

**Figure 25.**
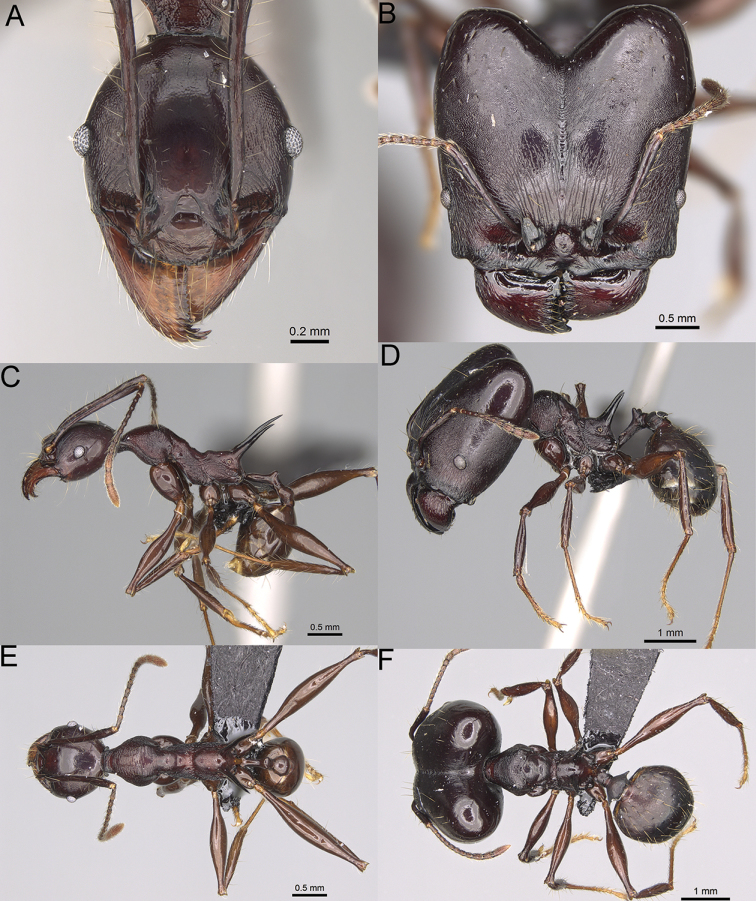
*Pheidole praegrandis* sp. nov., full-face view (**A**), profile (**C**), and dorsal view (**E**) of paratype minor worker (CASENT0494945) and full-face view (**B**), profile (**D**), and dorsal view (**F**) of holotype major worker (CASENT0494942).

Figs 25A–F , 85S , 88C

###### Type material.

***Holotype*.** Madagascar. •1 major worker; Antsiranana; Parc National de Marojejy, Manantenina River, 27.6 km 35°NE Andapa, 9.6 km 327°NNW Manantenina; -14.435, 49.76; alt. 775 m; 15 Nov 2003; B.L. Fisher et al. leg.; BLF08889, CASENT0494942, top specimen on the pin (CASC). ***Paratypes*.** Madagascar. •9w., 2s.; same data as for holotype; CASENT0494952, CASENT0872086, CASENT0494943-CASENT0494945, CASENT0872223–CASENT0872229 (CASC).

###### Other material.

Madagascar. –***Antsiranana***: •1w., 1s.; Makirovana Forest; -14.16044, 49.95216; alt. 550 m; 1 May 2011; B.L. Fisher et al. leg. CASENT0212471 (CASC). •7w.; Makirovana Forest; -14.17066, 49.95409; alt. 415 m; 29 Apr 2011; B.L. Fisher et al. leg. CASENT0212806, CASENT0231274, CASENT0236088, CASENT0236098 (CASC). •6w., 2s., 1q.; Makirovana Forest; -14.16506, 49.9477; alt. 900 m; 30 Apr 2011; B.L. Fisher et al. leg.; CASENT0231001, CASENT0231002, CASENT0231012, CASENT0231014, CASENT0231043 (CASC). •4w., 2s., 2q.; Makirovana Forest; -14.16666, 49.95, alt. 715 m; 2 May 2011; B.L. Fisher et al. leg.; CASENT0231097, CASENT0231098, CASENT0231113, CASENT0231167 (CASC). •2w., 2s.; Masoala National Park; -15.33058, 50.30279, alt. 250 m; 13 Mar 2014; B.L. Fisher et al. leg.; CASENT0374505, CASENT0377081 (CASC). •21w., 11s., 1q.; Parc National de Marojejy, Manantenina River, 27.6 km 35°NE Andapa, 9.6 km 327°NNW Manantenina; -14.435, 49.76; alt. 775 m; 15 Nov 2003; B.L. Fisher et al. leg.; CASENT0045333, CASENT0045341, CASENT0045357, CASENT0045368, CASENT0045372, CASENT0048887, CASENT0048895, CASENT0235122, CASENT0487704, CASENT0487706, CASENT0487825, CASENT0487903, CASENT0494780, CASENT0494781, CASENT0077076, CASENT0077079, CASENT0077119, CASENT0077122 (CASC). –***Fianarantsoa***: •4w.; Forêt d’Ambalagoavy Nord, Ikongo, Ambatombe; -21.857068, 47.37849; alt. 625 m; 1 Dec 2000; Harin’Hala & Irwin leg.; CASENT0009561, CASENT0009562, CASENT0009566, CASENT0009571 (CASC). –***Toamasina***: •10w., 8s., 3q.; Montagne d’Anjanaharibe, 18.0 km 21°NNE Ambinanitelo; -15.18833, 49.615; alt. 470 m; 8 Mar 2003; B.L. Fisher et al. leg.; CASENT0495134, CASENT0495141, CASENT0495145, CASENT0495193, CASENT0495220, CASENT0495221, CASENT0495222, CASENT0495393, CASENT0495395, CASENT0495396 (CASC). •4w., 3s.; Reserve Betampona, Camp Vohitsivalana, 37.1 km 338° Toamasina; -17.88667, 49.2025; alt. 520 m; 2 Dec 2005; B.L. Fisher et al. leg.; CASENT0067653, CASENT0067655, CASENT0067915, CASENT0069180 (CASC).

###### Diagnosis.

***Major workers*.** Large species: HL: 2.94–3.2 (3.0), HW: 2.68–3.1 (2.8), WL: 2.01–2.24 (2.11); propodeal spines very long (PSL: 0.81–0.96 (0.89)); head in full-face view trapezoid, widened posteriorly; sides of the head without pilosity; frons with fine, dense, longitudinal rugae reaching at most midlength of head; first gastral tergite shagreened, at least on its basal half; body dark brown to black. ***Minor workers*.** Large species: HL: 1.15–1.29 (1.2), HW: 1.04–1.14 (1.1), WL: 1.86–2.01 (1.92); propodeal spines very long (PSL: 0.91–1.0 (0.97)); scape, when laid back, surpassing posterior head margin by more than half its length; mesosoma finely rugoreticulate; promesonotal groove absent or very indistinct; metanotal groove shallow and wide.

###### Description.

**Major workers.** Measurements (*N* = 10): HL: 2.94–3.2 (3.0); HW: 2.68–3.1 (2.8); SL: 1.36–1.45 (1.39); EL: 0.24–0.3 (0.27); WL: 2.01–2.24 (2.11); PSL: 0.81–0.96 (0.89); MTL: 1.53–1.7 (1.6); PNW: 0.84–1.0 (0.92); PTW: 0.28–0.32 (0.3); PPW: 0.76–0.9 (0.82); CI: 90.1–96.6 (93.4); SI: 47.4–51.0 (49.3); PSLI: 27.4–31.7 (29.5); PPI: 33.1–38.4 (36.5); PNI: 30.1–35.9 (32.6); MTI: 54.9–59.9 (56.5). ***Head*.** In full-face view trapezoid, widened posteriorly (Fig. 25B). In lateral view sub-oval; ventral and dorsal faces convex; inner hypostomal teeth visible. Sides of the head without pilosity; frons and vertex with few, long, erect setae. Antennal scrobes absent. Occipital lobes shiny and smooth or partially shagreened; genae smooth and shiny, sometimes partially shagreened; centre of frons shiny with fine, dense, longitudinal rugae reaching at most midlength of head; malar area shagreened with several short, longitudinal rugae; lateral sides of head and posterior part of frons shagreened; head sculpture weakens posteriorly. Centre of clypeus smooth and shiny, lateral sides with longitudinal rugae; median notch present, shallow and wide; median longitudinal carina absent; lateral longitudinal carinae absent. Scape, when laid back, reaching midlength of head; pilosity suberect to erect (Fig. 25B, D). Inner hypostomal teeth distinct, triangular and thick, with rounded apex, closely spaced; outer hypostomal teeth distinct, slightly smaller and thinner than inner hypostomal teeth, lobe-like (Fig. 85S). ***Mesosoma*.** In lateral view, promesonotum low, short, and relatively flat; dorsal pronotum slightly convex; dorsal mesonotum slightly concave; posterior mesonotum relatively convex; promesonotal groove absent; metanotal groove absent; propodeal spines very long, massive basally, with acute apex; humeral area laterally weakly or not produced (Fig. 25D). Surface shiny, rugoreticulate, propodeal dorsum with weaker sculpture. Pilosity very sparse, short, and decumbent; dorsum with few additional, long, erect setae (Fig. 25D, F). ***Petiole*.** Weakly shagreened; peduncle relatively long and thin; node triangular with rounded apex, in rear view node dorsoventrally depressed; pilosity sparse, short, and erect (Fig. 25D, F). ***Postpetiole*.** Weakly shagreened; in dorsal view sides with acute, moderately wide, short, and triangular projections; pilosity short to long, sparse and erect (Fig. 25D, F). ***Gaster*.** First gastral tergite shagreened, at least on its basal half; pilosity sparse, long, and erect (Fig. 25D, F). ***Colour*.** Unicolourous, dark brown to black (Fig. 25D, F).

**Minor workers.** Measurements (*N* = 10): HL: 1.15–1.29 (1.2); HW: 1.04–1.14 (1.1); SL: 1.52–1.69 (1.62); EL: 0.17–0.2 (0.19); WL: 1.86–2.01 (1.92); PSL: 0.91–1.0 (0.97); MTL: 1.42–1.54 (1.49); PNW: 0.75–0.81 (0.77); PTW: 0.15–0.2 (0.17); PPW: 0.27–0.35 (0.32); CI: 87.5–92.7 (89.7); SI: 140.8–156.6 (147.6); PSLI: 75.8–84.0 (78.5); PPI: 45.6–60.8 (53.1); PNI: 67.6–72.1 (70.1); MTI: 126.2–143.4 (134.8). ***Head*.** Occipital margin convex; occipital carina narrow, weakly developed (Fig. 25A). Pilosity sparse, short, and appressed on the whole surface and additionally long and erect on frons and median part of occiput. Sculpture variable, shiny, smooth to finely rugulose on vertex, centre, and posterior part of frons and genae; lateral sides of head finely rugulose; antennal sockets with sparse carinae curved outward. Clypeus with median longitudinal carina absent; two lateral longitudinal carinae present. Scape, when laid back, surpassing posterior head margin by more than half of its length; pilosity suberect to erect (Fig. 25A, C). ***Mesosoma*.** In lateral view, promesonotum low, long, and slightly convex; promesonotal groove absent or very indistinct; metanotal groove shallow and wide; propodeal spines very long, massive basally, with acute apex (Fig. 25C). Surface finely rugoreticulate. Pilosity short, very sparse, suberect (Fig. 25C, E). ***Petiole*.** Peduncle long and thin; node triangular with rounded apex; pilosity absent (Fig. 25C, E). ***Postpetiole*.** Moderately short, low, and slightly convex; with two long, erect setae at the anterior edge (Fig. 25C, E). ***Gaster*.** With few long, erect setae (Fig. 25C, E). ***Colour*.** Unicolourous, dark brown to black (Fig. 25C, E).

###### Etymology.

Latin for huge, in reference to large body size and very long propodeal spines.

###### Biology.

The species was collected between 250–900 m in elevation, in rainforest and montane rainforest. Nests were located in rotten logs and tree stumps.

###### Comments.

This species is most similar to *P. longispinosa* and *P. mahaboensis* sp. nov. ***Major workers*.***Pheidole praegrandis* sp. nov. can be distinguished from *P. longispinosa* by shagreened surface of head and first gastral tergite, and darker body colouration; from *P. mahaboensis* sp. nov. by absence of metanotal groove, absence of very sparse, short, decumbent pilosity at the sides of head, and shagreened first gastral tergite and head. ***Minor workers*.***Pheidole praegrandis* sp. nov. can be distinguished from *P. longispinosa* by rugoreticulate mesosoma sculpture; from *P. mahaboensis* sp. nov. by rugoreticulate mesosoma sculpture, and shallow and wide metanotal groove.

#### Revision of the *Pheidole scabrata* complex

**Diagnosis.** Sides of the head with sparse, relatively long, erect pilosity; antennal scrobes absent; occipital lobes smooth, at least on the posterior part; genae smooth to finely rugulose; centre of frons with longitudinal rugae directed outward posteriorly; inner and outer teeth closely spaced and connected by indistinct concavity; promesonotal groove absent; mesosoma with sparse to relatively dense rugoreticulation, mesosomal dorsum and propodeum with weaker sculpture or smooth patches; first gastral tergite smooth to shagreened. ***Minor workers*.** Scape, when laid back, surpassing posterior head margin by one third of its length; promesonotal groove absent or very indistinct; metanotal groove very indistinct; mesosoma with dense rugoreticulation, sometimes dorsum with weaker sculpture, but never smooth.

**Comments.** Species of this complex are characterized by a large body, both in minor and major workers. Major workers can be distinguished by a combination of the following characters: head, in full-face view, trapezoid and widened posteriorly, in lateral view sub-oval; sides of the head with sparse, relatively long, erect pilosity, occipital lobes predominantly smooth; frons with rugae directed outward, and very long, massive propodeal spines. Minor workers can be distinguished based on long scape (surpassing posterior head margin by one-third of its length); very long propodeal spines; mesosoma with dense rugoreticulation, and long and thin peduncle of petiole.

##### Pheidole scabrata

Taxon classificationAnimaliaHymenopteraFormicidae

Forel, 1895 stat. nov.

**Figure 26.**
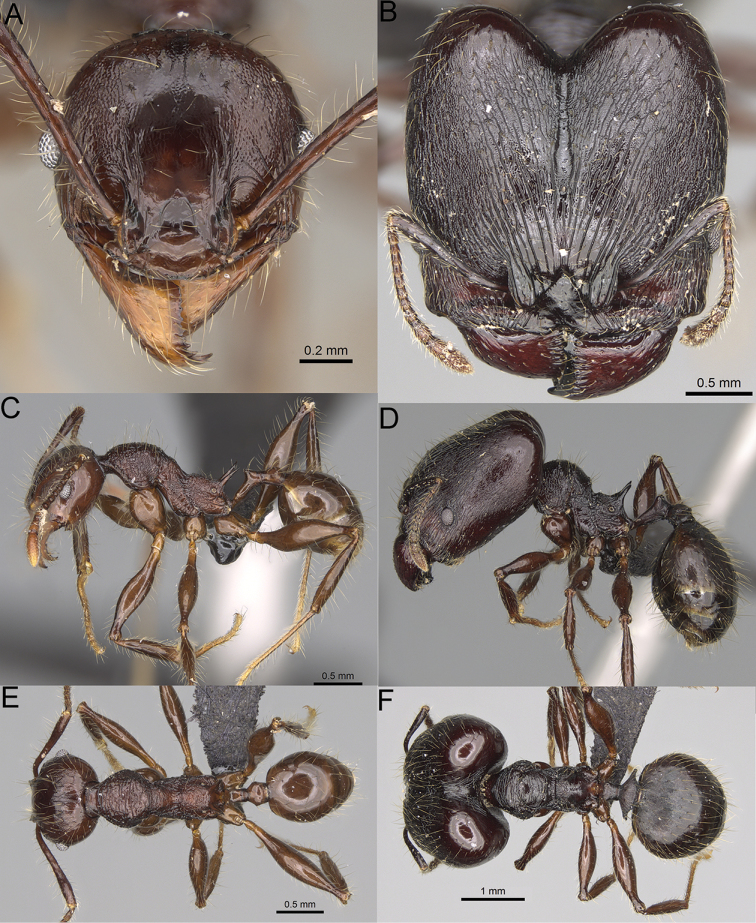
*Pheidole scabrata* Forel, full-face view (**A**), profile (**C**), and dorsal view (**E**) of minor worker (CASENT0496745) and full-face view (**B**), profile (**D**), and dorsal view (**F**) of major worker (CASENT0496747).

Figs 26A–F , 85W , 88G

###### Type material.

*Pheidole longispinosa scabrata* Forel, 1895a: 249 (s.w.). Lectotype [designated here]: major worker (CASENT0101695): Madagascar, Est Imerina, coll. Sikora (MHNG) [examined]. Paralectotypes: 3 minor workers (1 pin, CASENT0101869) (MHNG) [examined], 1 major worker (CASENT0923189) (MHNG) [examined]: the same data as lectotype.

###### Other material.

Madagascar. –***Antsiranana***: •4w., 4q.; 9.2 km WSW Befingotra, Rés. Anjanaharibe-Sud; -14.75, 49.46667; alt. 1200 m; 9 Nov 1994; B.L. Fisher leg.; CASENT0198388, CASENT0198392, CASENT0198395, CASENT0198396 (CASC). •32w., 7s.; Forêt de Binara, 9.4 km 235°SW Daraina; -13.26333, 49.6; alt. 1100 m; 5 Dec 2003; B.L. Fisher leg.; CASENT0043382, CASENT0043383, CASENT0494064–CASENT0494066, CASENT0494103–CASENT0494105, CASENT0494126–CASENT0494128, CASENT0494134–CASENT0494136, CASENT0494152 (CASC). •4w.; R.S. Manongarivo, 14.5 km 220°SW Antanambao; -13.99833, 48.42833; alt. 1175 m; 20 Oct 1998; B.L. Fisher leg.; CASENT0198007, CASENT0198386 (CASC). •3w.; R.S. Manongarivo, 14.5 km 220°SW Antanambao; -14, 48.43167; alt. 1220 m; 18 Oct 1998; B.L. Fisher leg.; CASENT0198387 (CASC). •1w.; R.S. Manongarivo, 14.5 km 220°SW Antanambao; -13.99833, 48.42833; alt. 1175 m; 20 Oct 1998; B.L. Fisher leg.; CASENT0198767 (CASC). –***Mahajanga***: •2w.; Réserve Spéciale Marotandrano, Marotandrano 48.3km S Mandritsara; -16.28322, 48.81443; alt. 865 m; 6 Dec 2007; B.L. Fisher et al. leg.; CASENT0140632, CASENT0140653 (CASC). –***Toamasina***: •1w., 1s.; 23 km E Moramanga; -18.98028, 48.45306; alt. 900 m; 10 Nov 1996; B.L. Fisher leg.; CASENT0198385 (CASC). •1w.; Ambatovy, 12.4 km NE Moramanga; -18.85813, 48.28488; alt. 1040 m; 5 Mar 2007; B.L. Fisher et al. leg.; CASENT0124242 (CASC). •2w., 2s.; Bevolota 17.1 km N Andasibe; -18.77071, 48.43164; alt. 995 m; 12 Dec 2007; B.L. Fisher et al. leg.; CASENT0135100, CASENT0235126 (CASC). •2w., 1s.; Forêt Ambatovy, 14.3 km 57° Moramanga; -18.85083, 48.32; alt. 1075 m; 21 Mar 2004; B.L. Fisher et al. leg.; CASENT0047076, CASENT0052866 (CASC). –***Toliara***: •4w., 3s.; Réserve Spéciale d’Ambohijanahary, Forêt d’Ankazotsihitafototra, 34.6 km 314°NW Ambaravaranala; -18.26, 45.41833; alt. 1100 m; 16 Jan 2003; B.L. Fisher et al. leg.; CASENT0029499, CASENT0029619, CASENT0029620, CASENT0029719–CASENT0029722 (CASC). •22w., 5s.; Réserve Spéciale d’Ambohijanahary, Forêt d’Ankazotsihitafototra, 35.2 km 312°NW Ambaravaranala; -18.26667, 45.40667; alt. 1050 m; 13 Jan 2003; B.L. Fisher et al. leg.; CASENT0028299, CASENT0028313, CASENT0028333, CASENT0485910, CASENT0485911, CASENT0485912, CASENT0485913, CASENT0496690, CASENT0496691, CASENT0496743, CASENT0496744, CASENT0496745, CASENT0496747 (CASC).

###### Diagnosis.

***Major workers*.** Large species: HL: 2.44–2.72 (2.56), HW: 2.3–2.58 (2.43), WL: 1.69–1.85 (1.77); propodeal spines very long (PSL: 0.5–0.63 (0.55)); head in full-face view trapezoid, widened posteriorly; sides of the head with sparse, relatively long, erect pilosity; frons with rugoreticulation and additional longitudinal rugae on the whole surface, sculpture weakening posteriorly; first gastral tergite smooth to slightly shagreened; body brown to dark brown; inner hypostomal teeth distinct, triangular and thick, with rounded apex, closely spaced; outer hypostomal teeth distinct, low, lobe-like, with wide base, smaller and thinner than inner hypostomal teeth. ***Minor workers*.** Large species: HL: 0.93–1.06 (0.99); HW: 0.9–1.06 (0.96), 1.41–1.65 (1.48); propodeal spines very long (PSL: 0.41–0.48 (0.43)); scape, when laid back, surpassing posterior head margin by one third of its length; head sculpture never rugoreticulate; mesosoma with dense rugoreticulation, sometimes dorsum with weaker sculpture, but never smooth.

###### Redescription.

**Major workers.** Measurements (*N* = 10): HL: 2.44–2.72 (2.56); HW: 2.3–2.58 (2.43); SL: 1.0–1.12 (1.06); EL: 0.23–0.3 (0.27); WL: 1.69–1.85 (1.77); PSL: 0.5–0.63 (0.55); MTL: 1.14–1.29 (1.22); PNW: 0.77–0.94 (0.88); PTW: 0.25–0.29 (0.27); PPW: 0.71–0.89 (0.8); CI: 93.1–96.5 (94.8); SI: 41.3–45.7 (43.5); PSLI: 19.2–24.9 (21.4); PPI: 31.0–38.3 (34.0); PNI: 33.5–38.4 (36.1); MTI: 48.2–52.5 (50.3). ***Head*.** In full-face view trapezoid, widened posteriorly (Fig. 26B). In lateral view sub-oval; ventral and dorsal faces convex; inner hypostomal teeth visible. Sides of the head with sparse, relatively long, erect pilosity; whole head with moderately sparse, long erect setae. Antennal scrobes absent. Occipital lobes shiny and smooth or partially rugulose; genae smooth and shiny or with indistinct rugulae; centre of frons shiny, rugoreticulate, longitudinal rugae directed outward posteriorly; malar area rugoreticulate; lateral sides of head shiny, with sparse rugoreticulation; head sculpture weakens posteriorly. Centre of clypeus smooth and shiny, lateral sides with longitudinal rugae; median notch present, shallow and wide; median longitudinal carina absent; lateral longitudinal carinae absent. Scape, when laid back, reaching midlength of head; pilosity suberect to erect (Fig. 26B, D). Inner hypostomal teeth distinct, triangular and thick, with rounded apex, closely spaced; outer hypostomal teeth distinct, low, lobe-like, with wide base, smaller and thinner than inner hypostomal teeth; inner and outer teeth closely spaced and connected by indistinct concavity (Fig. 85W). ***Mesosoma*.** In lateral view, promesonotum short, low, and evenly convex, with relatively steep posterior declivity; promesonotal groove absent; metanotal groove present; propodeal spines very long, massive basally, with acute apex; humeral area laterally weakly produced (Fig. 26D). Surface shiny, with sparse to relatively dense rugoreticulation, mesosomal dorsum and propodeum with weaker sculpture, sometimes with smooth patches; katepisternum with smooth area. Pilosity moderately sparse, long, and erect (Fig. 26D, F). ***Petiole*.** Smooth to weakly shagreened; peduncle relatively long and thin; node triangular with rounded apex, in rear view node dorsoventrally slightly depressed; pilosity moderately sparse and erect (Fig. 26D, F). ***Postpetiole*.** Smooth to weakly shagreened; in dorsal view sides with acute, and short to moderately short angular projections; pilosity long, moderately sparse, and erect (Fig. 26D, F). ***Gaster*.** First gastral tergite smooth to slightly shagreened; pilosity moderately sparse, long, and erect (Fig. 26D, F). ***Colour*.** Unicolourous, brown to dark brown, sometimes malar area and lower part of frons with brighter colouration than the rest of body (Fig. 26D, F).

**Minor workers.** Measurements (*N* = 10): HL: 0.93–1.06 (0.99); HW: 0.9–1.06 (0.96); SL: 1.14–1.35 (1.23); EL: 0.17–0.19 (0.18); WL: 1.41–1.65 (1.48); PSL: 0.41–0.48 (0.43); MTL: 1.05–1.21 (1.12); PNW: 0.64–0.72 (0.67); PTW: 0.11–0.16 (0.13); PPW: 0.24–0.37 (0.3); CI: 95.4–100.5 (97.4); SI: 123.8–132.7 (127.5); PSLI: 41.1–45.6 (43.7); PPI: 41.8–51.3 (45.7); PNI: 67.6–73.2 (69.8); MTI: 111.8–119.5 (116.3). ***Head*.** Occipital margin convex; occipital carina narrow, weakly developed (Fig. 26A). Pilosity moderately sparse, long, and erect. Sculpture variable, shiny, smooth to finely rugoreticulate on vertex, centre and posterior part of frons; genae smooth; lateral sides of head with few short, longitudinal rugulae, and sometimes finely shagreened; antennal sockets with sparse carinae curved outward. Clypeus with median longitudinal carina absent; two lateral longitudinal carinae present. Scape, when laid back, surpassing posterior head margin by one third of its length; pilosity suberect to erect (Fig. 26A, C). ***Mesosoma*.** In lateral view, promesonotum low, long, and slightly convex; promesonotal groove absent or very indistinct; metanotal groove very indistinct; propodeal spines very long, massive basally, with acute apex (Fig. 26C). Surface shiny, with dense rugoreticulation, sometimes dorsum with weaker sculpture, but never smooth. Pilosity sparse, long, and erect (Fig. 26C, E). ***Petiole*.** Peduncle long and thin; node triangular with rounded apex; with few long, erect setae (Fig. 26C, E). ***Postpetiole*.** Moderately short, low, and indistinctly convex; with few long, erect setae at the anterior edge (Fig. 26C, E). ***Petiole*.** With few long, erect setae (Fig. 26C, E). ***Colour*.** Unicolourous, brown to dark brown (Fig. 26C, E).

###### Biology.

The species was collected at between 825–1220 m in elevation, in montane rainforest, rainforest, transitional humid forest, and tropical dry forest. Nests were located in rotten logs, and in rotten sticks on the ground.

###### Comments.

This species is most similar to *P. maizina* sp. nov. ***Major workers*.***Pheidole scabrata* can be distinguished from *P. maizina* sp. nov. by smooth and shiny genae or genae with fine sculpture; smooth area on katepisternum; tips of outer hypostomal teeth not directed outward, never densely shagreened surface of first gastral tergite, and brighter body colouration. ***Minor workers*.***Pheidole scabrata* can be distinguished from *P. maizina* sp. nov. by weak head sculpture, and always smooth genae.

##### Pheidole maizina sp. nov.

Taxon classificationAnimaliaHymenopteraFormicidae

http://zoobank.org/0E6A56CB-6415-4402-A3C9-80FFD4F86BF7

**Figure 27.**
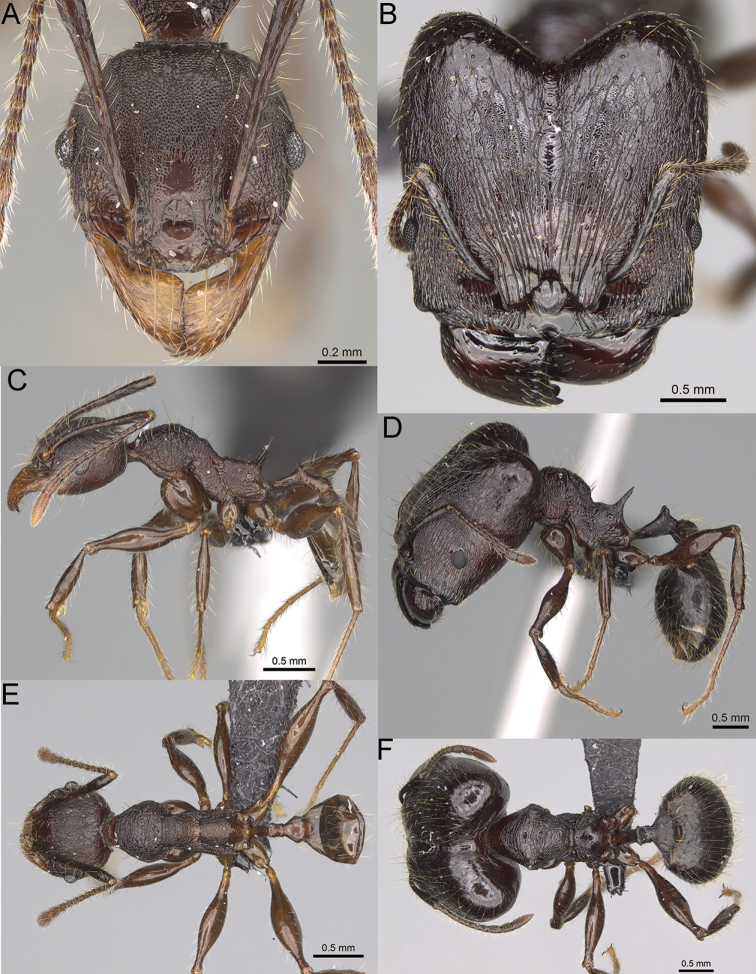
*Pheidole maizina* sp. nov., full-face view (**A**), profile (**C**), and dorsal view (**E**) of paratype minor worker (CASENT0923223) and full-face view (**B**), profile (**D**), and dorsal view (**F**) of holotype major worker (CASENT0162231).

Figs 27A–F , 85C , 87G

###### Type material.

***Holotype*.** Madagascar. •1 major worker; Toamasina; Réserve Spéciale Ambatovaky, Sandrangato River; -16.80561, 49.29507; alt. 480 m; 27 Feb 2010; B.L. Fisher et al. leg.; BLF25039, CASENT0162231 (CASC). ***Paratypes*.** Madagascar. •2w., 1q.; same data as for holotype; CASENT0162230, CASENT0923223, CASENT0872222 (CASC).

###### Other material.

Madagascar. –***Antsiranana***: •3w., 1s.; 6.5 km SSW Befingotra, Rés. Anjanaharibe-Sud; -14.75, 49.5; alt. 875 m; 19 Oct 1994; B.L. Fisher leg.; CASENT0198389, CASENT0198390 (CASC). •7w., 4s., 1q.; 9.2 km WSW Befingotra, Rés. Anjanaharibe-Sud; -14.75, 49.46667; alt. 1200 m; 8 Nov 1994; B.L. Fisher leg.; CASENT0198391, CASENT0198393, CASENT0198394, CASENT0235113, CASENT0235114 (CASC). –***Fianarantsoa***: •1w.; Parc National de Ranomafana, Vatoharanana River, 4.1 km 231°SW Ranomafana; -21.29, 47.43333; alt. 1100 m; 27 Mar 2003; B.L. Fisher et al. leg.; CASENT0497619 (CASC). –***Mahajanga***: •3w., 2s.; Réserve Spéciale Marotandrano, Marotandrano 48.3 km S Mandritsara; -16.28322, 48.81443; alt. 865 m; 7 Dec 2007; B.L. Fisher et al. leg.; CASENT0134242, CASENT0134279, CASENT0235121 (CASC). –***Toamasina***: •1w.; Ambanizana, Parc National Masoala; -15.57222, 50.00694; alt. 930–1110 m; 2 Mar 2003; Andriamalala et al. leg.; CASENT0073482 (CASC). •1w.; F.C. Andriantantely; -18.695, 48.81333; alt. 530 m; 4 Dec 1998; Ratsirarson leg.; CASENT0198237 (CASC). •19w., 1s.; Montagne d’Akirindro 7.6 km 341°NNW Ambinanitelo; -15.28833, 49.54833; alt. 600 m; 17 Mar 2003; B.L. Fisher et al. leg.; CASENT0039054, CASENT0039064, CASENT0039103, CASENT0039188, CASENT0039200, CASENT0039209, CASENT0039219, CASENT0235138, CASENT0496323, CASENT0496345, CASENT0496346, CASENT0496347 (CASC). •15w., 4s.; Montagne d’Anjanaharibe, 18.0 km 21°NNE Ambinanitelo; -15.18833, 49.615; alt. 470 m; 8 Mar 2003; B.L. Fisher et al. leg.; CASENT0037623, CASENT0037626, CASENT0048951, CASENT0495382, CASENT0495383, CASENT0495384, CASENT0495480, CASENT0495481, CASENT0495482 (CASC). •1w.; Réserve Spéciale Ambatovaky, Sandrangato River; -16.7633, 49.26692; alt. 520 m; 22 Feb 2010; B.L. Fisher et al. leg.; CASENT0160480 (CASC). •2w.; Réserve Spéciale Ambatovaky, Sandrangato River; -16.7702, 49.26638; alt. 470 m; 23 Feb 2010; B.L. Fisher et al. leg.; CASENT0161455 (CASC). •1w.; Réserve Spéciale Ambatovaky, Sandrangato River; -16.8162, 49.29202; alt. 425 m; 25 Feb 2010; B.L. Fisher et al. leg.; CASENT0161854 (CASC). •2w.; Réserve Spéciale Ambatovaky, Sandrangato River; -16.81745, 49.2925; alt. 400 m; 26 Feb 2010; B.L. Fisher et al. leg.; CASENT0162160 (CASC). •4w., 2s., 1q.; Réserve Spéciale Ambatovaky, Sandrangato River; -16.77274, 49.26551; alt. 450 m; 20 Feb 2010; B.L. Fisher et al. leg.; CASENT0162675, CASENT0162686, CASENT0162688, CASENT0162689 (CASC). •4w., 4s., 3q.; Réserve Spéciale Ambatovaky, Sandrangato River; -16.80561, 49.29507; alt. 480 m; 27 Feb 2010; B.L. Fisher et al. leg.; CASENT0163009, CASENT0163019, CASENT0163021, CASENT0163027, CASENT0163032, CASENT0163040, CASENT0163073 (CASC). •2w.; Réserve Spéciale Ambatovaky, Sandrangato River; -16.7633, 49.26692; alt. 520 m; 22 Feb 2010; B.L. Fisher et al. leg.; CASENT0163856, CASENT0163945 (CASC).

###### Diagnosis.

***Major workers*.** Large species: HL: 2.38–2.71 (2.55), HW: 2.17–2.54 (2.4), WL: 1.6–1.95 (1.76); propodeal spines very long (PSL: 0.48–0.59 (0.54)); head in full-face view trapezoid, widened posteriorly; sides of the head with sparse, relatively long, erect pilosity; frons with longitudinal, sparse, and directed outward rugae, interspaces with dense and fine rugulae, sculpture weakening posteriorly; first gastral tergite shagreened, at least on its basal part; body dark brown to black; inner hypostomal teeth distinct, triangular and thick, with rounded apex, closely spaced; outer hypostomal teeth distinct, low, lobe-like, with wide base and tops directed outward, smaller and thinner than inner hypostomal teeth. ***Minor workers*.** Large species: HL: 0.85–1.03 (0.93), HW: 0.8–1.0 (0.89), WL: 1.21–1.47 (1.36); propodeal spines very long (PSL: 0.34–0.44 (0.39)); scape, when laid back, surpassing posterior head margin by one-third of its length; head sculpture rugoreticulate; mesosoma with dense rugoreticulation, sometimes dorsum with weaker sculpture, but never smooth.

###### Description.

**Major workers.** Measurements (*N* = 10): HL: 2.38–2.71 (2.55); HW: 2.17–2.54 (2.4); SL: 0.95–1.17 (1.08); EL: 0.24–0.3 (0.27); WL: 1.6–1.95 (1.76); PSL: 0.48–0.59 (0.54); MTL: 1.16–1.28 (1.23); PNW: 0.8–0.95 (0.9); PTW: 0.24–0.31 (0.28); PPW: 0.7–0.92 (0.83); CI: 91.3–98.5 (94.0); SI: 43.6–46.3 (45.2); PSLI: 19.8–22.6 (21.2); PPI: 31.0–36.0 (34.1); PNI: 35.5–38.8 (37.4); MTI: 49.5–53.8 (51.2). ***Head*.** In full-face view trapezoid, widened posteriorly (Fig. 27B). In lateral view sub-oval; ventral and dorsal faces convex; inner hypostomal teeth visible. Sides of the head with sparse, relatively long, erect pilosity; whole head with moderately sparse, long, erect setae. Antennal scrobes absent. Occipital lobes shiny, and smooth at posterior part, anterior part with fine and dense rugulae; genae shiny, with fine and dense rugulae, sometimes posterior part smooth or with reduced sculpture; malar area and frons with longitudinal, sparse, and directed outward rugae, interspaces with dense and fine rugulae; lateral sides of head shiny, with dense and fine rugulae and thin, indistinct, longitudinal rugae. Centre of clypeus smooth and shiny, lateral sides with longitudinal rugae; median notch present, shallow, and wide; median longitudinal carina absent; lateral longitudinal carinae absent. Scape, when laid back, reaching mid-length of head; pilosity suberect to erect (Fig. 27B, D). Inner hypostomal teeth distinct, triangular, and thick, with rounded apex, closely spaced; outer hypostomal teeth distinct, low, lobe-like, with wide base and tops directed outward, smaller and thinner than inner hypostomal teeth (Fig. 85C). ***Mesosoma*.** In lateral view, promesonotum short, low, and evenly convex, with relatively steep posterior declivity; promesonotal groove absent; metanotal groove absent; propodeal spines very long, massive basally, with acute apex; humeral area laterally weakly produced (Fig. 27D). Surface shiny, with dense rugoreticulation, mesosomal dorsum and propodeum with weaker sculpture, but never smooth. Pilosity relatively dense, long, and erect (Fig. 27D, F). ***Petiole*.** Shagreened; peduncle relatively long and thin; node triangular with rounded apex, in rear view node dorsoventrally slightly depressed; pilosity moderately sparse and erect (Fig. 27D, F). ***Postpetiole*.** Shagreened; in dorsal view sides with acute, and short to moderately short angular projections; pilosity long, moderately sparse, and erect (Fig. 27D, F). ***Petiole*.** First gastral tergite shagreened, at least on its basal part; pilosity sparse, long and erect (Fig. 27D, F). ***Colour*.** Unicolourous, dark brown to black (Fig. 27D, F).

**Minor workers.** Measurements (*N* = 10): HL: 0.85–1.03 (0.93); HW: 0.8–1.0 (0.89); SL: 1.07–1.2 (1.16); EL: 0.16–0.21 (0.18); WL: 1.21–1.47 (1.36); PSL: 0.34–0.44 (0.39); MTL: 0.91–1.1 (1.01); PNW: 0.55–0.66 (0.61); PTW: 0.1–0.16 (0.13); PPW: 0.19–0.28 (0.23); CI: 93.3–101.0 (95.5); SI: 122.0–135.6 (130.7); PSLI: 40.0–44.9 (42.3); PPI: 50.5–60.2 (55.3); PNI: 66.3–70.4 (68.7); MTI: 111.2–117.0 (114.1). ***Head*.** Occipital margin convex; occipital carina narrow, weakly developed (Fig. 27A). Pilosity sparse, long, and erect. Sculpture variable, finely to strongly rugoreticulate on the whole surface; only centre of frons sometimes with sculpture reduced or smooth; antennal sockets with sparse carinae curved outward. Clypeus with median longitudinal carina absent; two lateral longitudinal carinae present. Scape, when laid back, surpassing posterior head margin by one-third of its length; pilosity suberect to erect (Fig. 27A, C). ***Mesosoma*.** In lateral view, promesonotum low, long, and slightly convex; promesonotal groove absent; metanotal groove very indistinct; propodeal spines very long, massive basally, with acute apex (Fig. 27C). Surface shiny, with dense rugoreticulation, sometimes dorsum with weaker sculpture, but never smooth. Pilosity sparse, long, and erect (Fig. 27C, E). ***Petiole*.** Finely shagreened; peduncle long and thin; node triangular with rounded apex; with few long, erect setae (Fig. 27C, E). ***Postpetiole*.** Moderately shagreened; moderately short, low, and convex; with few long, erect setae at the anterior edge (Fig. 27C, E). ***Petiole*.** With few long, erect setae (Fig. 27C, E). ***Colour*.** Unicolourous, dark brown to black (Fig. 27C, E).

###### Etymology.

Malagasy for dark, in reference to dark body colouration.

###### Biology.

The species was collected at between 400–1200 m in elevation, in rainforest, montane rainforest, and transitional humid forest. Nests were located in in rotten logs and stumps, and in rotten sticks on the ground.

###### Comments.

This species is most similar to *P. scabrata*. ***Major workers*.***Pheidole maizina* sp. nov. can be distinguished from *P. scabrata* by having distinct rugulae on at least half of the genae, tips of outer hypostomal teeth directed outward, never smooth katepisternum, densely shagreened surface of first gastral tergite, and darker body colouration. ***Minor workers*.***Pheidole maizina* sp. nov. can be distinguished from *P. scabrata* by rugoreticulate head sculpture with never smooth genae.

#### Revision of the *Pheidole ensifera* group

**Diagnosis. *Major workers*.** Head, in full face view, rectangular, in lateral view elongate and oval, ventral and dorsal faces slightly convex, dorsal face not depressed posteriorly; sides of the head with sparse, relatively long to long, suberect pilosity; antennal scrobes indistinct and not delimited by carinulae; scrobe surface shiny, with dense to sparse, fine, longitudinal to irregular rugulae; occipital lobes and genae smooth or with fine to thick, sparse rugoreticulation; frons with sparse, thick, and longitudinal rugae, sometimes rugae on posterior part of frons more irregular and directed outward; sculpture weakening posteriorly; inner and outer teeth closely spaced and connected by concavity; promesonotum short, angular, and relatively low to high; promesonotal and metanotal grooves absent; propodeal spines long to very long, thin; mesosoma with fine to thin, sparse to dense rugoreticulation, sculpture weakening on dorsum, sometimes propodeum with a smooth patch on its dorsal surface; gaster shagreened, at least at the basal part of first tergite; body reddish brown to dark brown. ***Minor workers*.** Head smooth or finely rugo-punctuate, lateral sides of head sometimes finely rugulose to rugo-punctate; scape, when laid back, surpassing posterior head margin by one- to two-fifths of its length; promesonotum high, short and convex; propodeal spines very to moderately long; whole mesosoma rugo-punctate, or smooth to moderately foveolate with sometimes thicker and denser sculpture on lateral sides; body bright brown to dark brown.

**Comments.** Species of this group are characterized by a large body size, both in minor and major workers. Major workers can be distinguished from others based on a combination of the following characters: head in full-face view rectangular, in lateral view elongate and oval; antennal scrobes with fine, longitudinal to irregular rugulae; occipital lobes and genae smooth or with sparse rugoreticulation; inner and outer teeth closely spaced and connected by concavity, and long to very long propodeal spines. Minor workers can be distinguished based on smooth to rugo-punctuate surface of frons; relatively short scape (surpassing posterior head margin by one- to two-fifths of its length); high, short, and convex promesonotum; very to moderately long propodeal spines, and bright brown to dark brown body colouration.

This group includes four species: *P. ensifera* Forel, *P. ocypodea* sp. nov., *P. aelloea* sp. nov., and *P. podargea* sp. nov. The distribution of the whole group is limited to the Northernmost part of the island (Antsiranana district) and all species are sympatric. Minor workers of members of this group manifest high infraspecific variability, therefore we encourage to use major workers in species determination.

##### Key to the *Pheidole ensifera* group

**Table:** 

1	Major workers. Occipital lobes and genae shiny, smooth or with very fine and sparse rugoreticulation; promesonotum, in lateral view, short, angular, and relatively low (Fig. 28A, E). Minor workers. Pronotum and mesonotum with fine to moderately dense foveolae, sometimes foveola weakening on the dorsal surface; head predominately smooth (Fig. 28I)	***P. ensifera* Forel**
–	Major workers. Occipital lobes shiny, with sparse and thick rugoreticulation; genae shiny, with moderately dense and fine rugoreticulation or smooth with anterior part with sparse and fine rugulae (Fig. 28B–D, F–H). Minor workers. Pronotum and at least dorsal surface of mesonotum smooth, with indistinct, sparse rugulae or with fine, dense rugoreticulation. If mesosoma entirely sculptured on the head finely rugo-punctate and smooth area, if present, limited to basal area of frons and genae (Fig. 28J–L)	**2**
2	Major workers. Posterior declivity of promesonotum steep to relatively steep, inner hypostomal teeth wide and pointed outward (Figs 28G, 29B). Minor workers. Petiolar peduncle long, postpetiole approximately 1.5 times longer that wide, head almost always smooth (Fig. 29E, I–J)	***P. ocypodea* sp. nov.**
–	Major workers. Posterior declivity of promesonotum convex, inner hypostomal teeth narrower and never pointed outward (Figs 28F, H, 29A, C). Minor workers. Petiolar peduncle short, postpetiole approximately as long as wide, at least lateral sides of head and malar area with fine rugae (Fig. 29D, F–H, K, L)	**3**
3	Major workers. Sides of head with sparse and long pilosity (distance between setae approximately as long as their length), genae and propodeum with reduced or partially absent sculpture (Fig. 28B, F). Minor workers. Vertex, genae, frons and mesosomal dorsum at least partially smooth, promesonotal and metanotal grooves absent (Figs 28J, 29D)	***P. aelloea* sp. nov.**
–	Major workers. Sides of head with denser and shorter pilosity (distance between shorter than their length), genae and propodeum never smooth (Fig. 28D, H). Minor workers. Head finely rugo-punctate and smooth area, if present, limited to basal area of frons and genae, promesonotal and metanotal grooves indistinct but always present (Figs 28L, 29F)	***P. podargea* sp. nov.**

**Figure 28.**
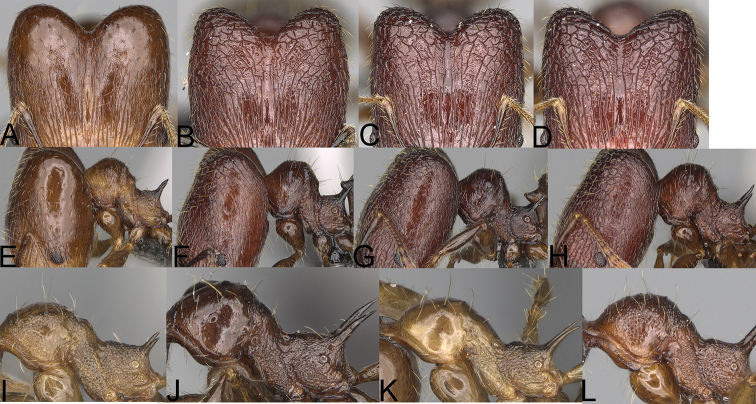
*Pheidole ensifera* Forel, head and profile of major worker (**A, E**), profile of minor worker (**I**). *Pheidole aelloea* sp. nov., head and profile of major worker (**B, F**), profile of minor worker (**J**). *Pheidole ocypodea* sp. nov., head and profile of major worker (**C, G)**, profile of minor worker (**K**). *Pheidole podargea* sp. nov., head and profile of major worker (**D, H**), profile of minor worker (**L**).

**Figure 29.**
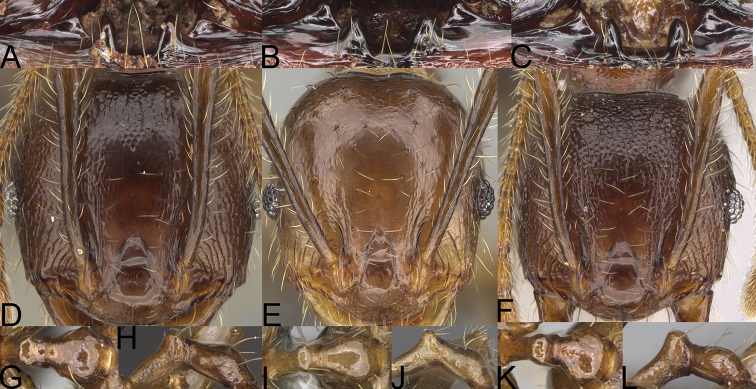
*Pheidole aelloea* sp. nov., hypostomal teeth (**A**), head of minor worker (**D**), petiole and postpetiole (**G, H**). *Pheidole ocypodea* sp. nov., hypostomal teeth (**B**), head of minor worker (**E**), petiole and postpetiole (**I, J**). *Pheidole podargea* sp. nov., hypostomal teeth (**C**), head of minor worker (**F**), petiole and postpetiole (**K, L**).

##### Pheidole ensifera

Taxon classificationAnimaliaHymenopteraFormicidae

Forel, 1897

**Figure 30.**
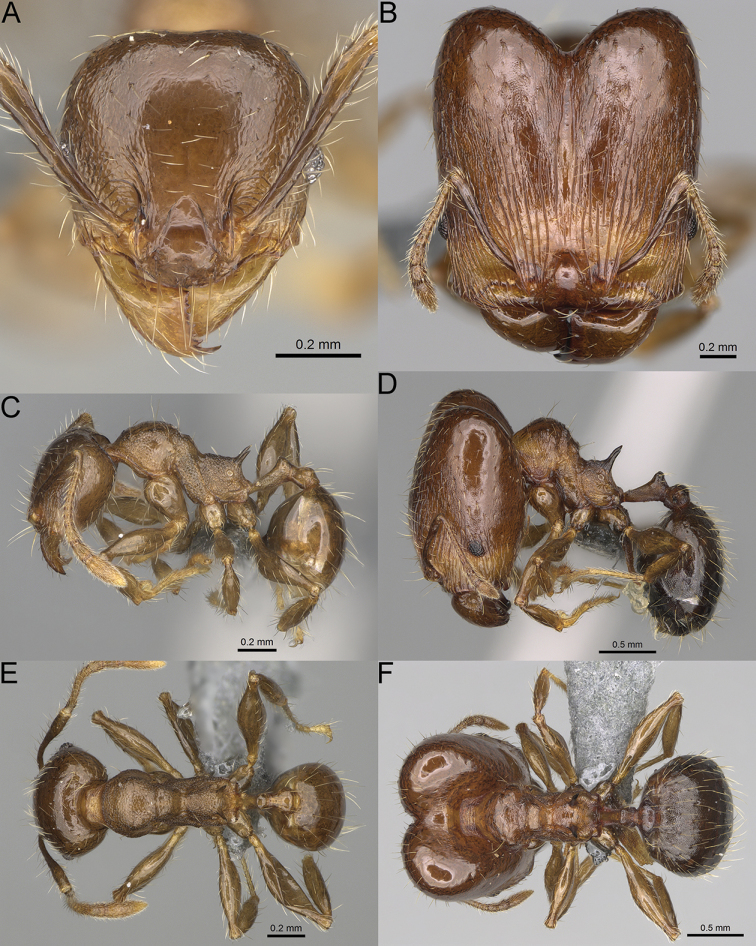
*Pheidole ensifera* Forel, full-face view (**A**), profile (**C**), and dorsal view (**E**) of minor worker (CASENT0298364) and full-face view (**B**), profile (**D**), and dorsal view (**F**) of major worker (CASENT0923226).

Figs 30A–F , 84O , 86O

###### Type material.

*Pheidole ensifera* Forel, 1897: 197 (s.w.). Lectotype [designated here]: major worker (top specimen, CASENT0101780): Madagascar, Antsiranana, Nosy Be, coll. Voeltzkow (MHNG) [examined]. Paralectotypes: 1 major worker (CASENT0810540, bottom specimen, the same pin as lectotype) (MHNG) [examined], 2 minor workers (1 pin, CASENT0101650) (MHNG) [examined], 2 minor workers (1 pin, CASENT0923207) (MHNG) [examined], 3 major workers (1 pin, CASENT0923208) (MHNG) [examined], 3 major workers (1 pin, CASENT0923209) (MHNG) [examined]: the same data as lectotype.

###### Other material.

Madagascar. –***Antsiranana***: •130w., 28s.; Ampasindava, Forêt d’Ambilanivy, 3.9 km 181°S Ambaliha; -13.79861, 48.16167; alt. 600 m; 4 Mar 2001; B.L. Fisher et al. leg.; CASENT0044202, CASENT0044202, CASENT0406703, CASENT0420010, CASENT0420016, CASENT0420048, CASENT0420052, CASENT0420054, CASENT0420057, CASENT0420059, CASENT0427703, CASENT0427705, CASENT046136, CASENT0464031, CASENT0464033, CASENT0464037, CASENT0464039, CASENT0464041, CASENT0464045, CASENT0464049, CASENT0464050, CASENT0464052, CASENT0464055, CASENT0464056, CASENT0464057, CASENT0464061, CASENT0464063, CASENT0464064, CASENT0464066, CASENT0464071, CASENT0464075, CASENT0464080, CASENT0464081, CASENT0464083, CASENT0464088, CASENT0464091, CASENT0464095, CASENT0464099, CASENT0464102, CASENT0464104–CASENT0464107, CASENT0464113, CASENT0464115, CASENT0464119, CASENT0464121, CASENT0464124, CASENT0464127, CASENT0464129, CASENT0464132, CASENT0464141, CASENT0464142, CASENT0464145, CASENT0464146, CASENT0464149, CASENT0464150, CASENT0464151, CASENT0464153, CASENT0464154, CASENT0464155, CASENT0464158, CASENT0464170, CASENT0464176, CASENT0464178, CASENT0464180, CASENT0464182, CASENT0464183, CASENT0464185, CASENT0464186, CASENT0464189, CASENT0464190, CASENT0464192, CASENT0464194, CASENT0464196, CASENT0464200–CASENT0464203, CASENT0464207–CASENT0464212, CASENT0464214, CASENT0464215, CASENT0464219, CASENT0464222, CASENT0464223, CASENT0464224, CASENT0464231, CASENT0464234,, CASENT0464237, CASENT0464240, CASENT0464240, CASENT0464243, CASENT0464244, CASENT0464250, CASENT0464253–CASENT0464256, CASENT0464258, CASENT0464259, CASENT0464261, CASENT0464262, CASENT0464264, CASENT0464266, CASENT0464273, CASENT0464279, CASENT0464289, CASENT0464292, CASENT0464296, CASENT0464298, CASENT0464307, CASENT0464315, CASENT0464317, CASENT0464326, CASENT0464332, CASENT0464342, CASENT0464357, CASENT0464364, CASENT0464367, CASENT0464368, CASENT0464371, CASENT0464381, CASENT0464382, CASENT0464383, CASENT0464391, CASENT0464393, CASENT0464396, CASENT046439, CASENT0464400, CASENT0464402, CASENT0464403, CASENT0464405, CASENT0464411, CASENT0464412, CASENT0464413, CASENT0464413, CASENT0464414, CASENT0464415 (CASC). •3w., 2s.; Forêt d’ Andavakoera, 21.4 km 75°ENE Ambilobe; 4.6 km 356°N Betsiaka; -13.11833, 49.23; alt. 425 m; 15 Dec 2003; B.L. Fisher leg.; CASENT0044099, CASENT0044127, CASENT0044204, CASENT0044205 (CASC). •1w., 1s.; Galoko chain, Mont Galoko; -13.58745, 48.71419; alt. 380 m; 23 Feb 2013; B.L. Fisher et al. leg.; CASENT0303020 (CASC). •1w., 3s.; Galoko chain, Mont Galoko; -13.58487, 48.71818; alt. 520 m; 17 Feb 2013; B.L. Fisher et al. leg.; CASENT0298364, CASENT0305044, CASENT0305049 (CASC). •1w., 1s.; Galoko chain, Mont Kalabenono; -13.63999, 48.67374; alt. 498 m; 15 Oct 2013; B.L. Fisher et al. leg.; CASENT0370653 (CASC). •41w., 20s., 3q.; Nosy Be, Réserve Naturelle Intégrale de Lokobe, 6.3 km 112°ESE Hellville; -13.41933, 48.33117; alt. 30 m; 19 Mar 2001; B.L. Fisher et al. leg.; CASENT0403272, CASENT0421458, CASENT0427835, CASENT0427836, CASENT0427876, CASENT0427879, CASENT0427887, CASENT0462766, CASENT0462792, CASENT0462797, CASENT0462798, CASENT0462803, CASENT0462805, CASENT0462806, CASENT0462807, CASENT0462810, CASENT0462880, CASENT0462881, CASENT0462884, CASENT0462886–CASENT0462891, CASENT0462896, CASENT0462913, CASENT0462914, CASENT0462916, CASENT0462924, CASENT0462930, CASENT0462933–CASENT0462935, CASENT0462938–CASENT0462942, CASENT0462963, CASENT0462988–CASENT0462991, CASENT0462993, CASENT0463018–CASENT0463021, CASENT0463044, CASENT0463060, CASENT0463063, CASENT0463067, CASENT0463075, CASENT0463101, CASENT0463118, CASENT0466246, CASENT0466297, CASENT0466299 (CASC). •5w., 2s.; R.S. Manongarivo, 10.8 km 229°SW Antanambao; -13.96167, 48.43333; alt. 400 m; 8 Nov 1998; B.L. Fisher leg.; CASENT0198013, CASENT0198014, CASENT0198522, CASENT0198524 (CASC). •4w., 1s; R.S. Manongarivo, 12.8 km 228°SW Antanambao; -13.97667, 48.42333; alt. 780 m; 11 Oct 1998; B.L. Fisher leg.; CASENT0198015, CASENT0198521, CASENT0198523 (CASC). •1w., 1s.; Sakaramy; -12.44114, 49.23197; alt. 260 m; 12 May 2011; B.L. Fisher et al. leg.; CASENT0261332 (CASC). –***Mahajanga***: •6w., 2s.; Réserve Spéciale Marotandrano, Marotandrano 48.3 km S Mandritsara; -16.28322, 48.81443; alt. 865 m; 6 Dec 2007; B.L. Fisher et al. leg.; CASENT0140640, CASENT0140643, CASENT0140667, CASENT0140672, CASENT0140682, CASENT0140685, CASENT0140687, CASENT0140692 (CASC).

###### Diagnosis.

***Major workers*.** Body size moderate: HL: 1.63–1.74 (1.69), HW: 1.43–1.51 (1.46), WL: 1.05–1.2 (1.11); propodeal spines very long (PSL: 0.31–0.37 (0.34)); head in full-face view rectangular, with lateral sides relatively straight, only their posteriormost part slightly convex; sides of the head with sparse, long, suberect pilosity; occipital lobes shiny, smooth or with very fine and sparse rugoreticulation; inner hypostomal teeth distinct, closely spaced, lobe-like, with rounded apex and wide base; outer hypostomal teeth bigger and wider than inner hypostomal teeth, lobe-like, with tops directed outward; inner and outer teeth closely spaced and connected by concavity. ***Minor workers*.** Body size moderate: HL: 0.58–0.69 (0.63), HW: 0.56–0.67 (0.6), WL: 0.76–0.87 (0.8); propodeal spines long (PSL: 0.18–0.22 (0.2)); scape, when laid back, surpassing posterior head margin by one-fifth of its length; lateral sides of head and malar area shiny, smooth or with indistinct, sparse rugulae, sculpture weakening posteriorly; vertex, genae and frons smooth; mesosoma foveolate.

###### Redescription.

**Major workers.** Measurements (*N* = 10): HL: 1.63–1.74 (1.69); HW: 1.43–1.51 (1.46); SL: 0.67–0.73 (0.7); EL: 0.17–0.19 (0.18); WL: 1.05–1.2 (1.11); PSL: 0.31–0.37 (0.34); MTL: 0.66–0.71 (0.69); PNW: 0.58–0.67 (0.61); PTW: 0.15–0.19 (0.17); PPW: 0.46–0.54 (0.5); CI: 85.6–88.8 (87.0); SI: 46.5–50.1 (47.9); PSLI: 18.8–22.5 (20.4); PPI: 30.0–36.9 (34.1); PNI: 40.2–44.6 (41.7); MTI: 44.3–48.7 (47.2). ***Head*.** In full-face view rectangular, with lateral sides relatively straight, only their posteriormost part slightly convex (Fig. 30B). In lateral view elongate and oval; ventral and dorsal faces slightly convex; inner hypostomal teeth visible. Sides of the head with sparse, long, suberect pilosity; whole head with moderately sparse, long, suberect to erect pilosity. Antennal scrobes indistinct and not delimited; scrobe surface shiny, with dense to sparse, fine, longitudinal to irregular rugulae. Occipital lobes and genae shiny, smooth or with very fine and sparse rugoreticulation; frons with sparse, thick, and longitudinal rugae, interspaces with very fine and sparse rugulae, sculpture weakening posteriorly; malar area with sparse to moderately sparse, thick, and longitudinal rugae, interspaces with fine and dense rugulae. Centre of clypeus smooth and shiny, lateral sides with longitudinal rugae; median notch present, narrow and shallow to moderate; median longitudinal carina absent; lateral longitudinal carinae absent. Scape, when laid back, slightly surpass the midlength of head; pilosity decumbent to erect (Fig. 30B, D). Inner hypostomal teeth distinct, closely spaced, lobe-like, with rounded apex and wide base; outer hypostomal teeth bigger and wider than inner hypostomal teeth, lobe-like, with tops directed outward; inner and outer teeth closely spaced and connected by concavity (Fig. 84O). ***Mesosoma*.** In lateral view, promesonotum short, angular and relatively low, posterior mesonotum convex, with low tubercle-like projection; promesonotal groove absent; metanotal groove absent; propodeal spines long, thin, massive basally, with acute apex; humeral area laterally weakly produced (Fig. 30D). Surface shiny, with fine to thin, dense rugoreticulation, sculpture weakening on dorsum, sometimes propodeum with smooth patch on its dorsal surface. Pilosity sparse, long, and erect (Fig. 30D, F). ***Petiole*.** Shiny; peduncle shagreened, long, without horizontal lobes on its basal part; node smooth, low, and thick, triangular, with rounded apex, in rear view node slightly convex; pilosity sparse and erect (Fig. 30D, F). ***Postpetiole*.** Shagreened; in dorsal view sides with short, acute, and triangular projections; pilosity long, sparse and erect (Fig. 30D, F). ***Petiole*.** Shagreened, most often on the whole surface; pilosity moderately sparse, long, and erect (Fig. 30D, F). ***Colour*.** Unicolourous, reddish brown to dark brown, malar area and lower parts of frons with colouration brighter that the rest of the body (Fig. 30D, F).

**Minor workers.** Measurements (*N* = 10): HL: 0.58–0.69 (0.63); HW: 0.56–0.67 (0.6); SL: 0.6–0.67 (0.63); EL: 0.1–0.12 (0.11); WL: 0.76–0.87 (0.8); PSL: 0.18–0.22 (0.2); MTL: 0.46–0.52 (0.49); PNW: 0.37–0.44 (0.39); PTW: 0.08–0.12 (0.09); PPW: 0.13–0.18 (0.14); CI: 92.5–100.9 (95.4); SI: 101.4–107.1 (104.5); PSLI: 29.0–34.1 (31.4); PPI: 56.3–67.0 (61.2); PNI: 63.0–67.6 (65.2); MTI: 79.2–83.2 (81.7). ***Head*.** Occipital margin straight or indistinctly concave; occipital carina indistinct, weakly developed (Fig. 30A). Pilosity moderately dense, long, suberect to erect. Lateral sides of head and malar area shiny, smooth or with indistinct, sparse rugulae, sculpture weakening posteriorly; vertex, genae, and frons smooth; antennal sockets with sparse, sometimes interrupted carinae curved outward. Clypeus with median longitudinal carina absent; two lateral longitudinal carinae present. Scape, when laid back, surpassing posterior head margin by one-fifth of its length; pilosity suberect to erect (Fig. 30A, C). ***Mesosoma*.** In lateral view, promesonotum convex; promesonotal groove absent; metanotal groove indistinct; propodeal spines moderately long, massive basally, with acute apex (Fig. 30C). Pronotum and mesonotum with fine to moderately dense foveolate, sometimes foveolae weakening on the dorsal surface; katepisternum, anepisternum, and propodeum with thicker and denser foveolae. Pilosity moderately sparse, long and erect (Fig. 30C, E). ***Petiole*.** Shiny; peduncle rugulae relatively long and thin; node smooth, relatively high, triangular; with few long, erect setae (Fig. 30C, E). ***Postpetiole*.** With indistinct rugulae; short and convex; with few long, erect setae at the anterior edge (Fig. 30C, E). ***Petiole*.** With sparse and erect setae (Fig. 30C, E). ***Colour*.** Unicolourous, brown to dark brown (Fig. 30C, E).

###### Biology.

The species was collected between 30–1343 m in elevation, in littoral and tropical dry rainforest and in-transition humid forest. Nests were located in litter (leaf mould, rotten wood), rotten logs and branches on the ground, and rotting tree stumps.

###### Comments.

***Major workers*.***Pheidole ensifera* differs from other members of the group in shiny and smooth to finely rugoreticulate occipital lobes and genae and relatively low and short promesonotum. ***Minor workers*.***Pheidole ensifera* differs from other members of the group in surface of pronotum and mesonotum never smooth and with fine to moderately dense foveolae.

##### Pheidole aelloea sp. nov.

Taxon classificationAnimaliaHymenopteraFormicidae

http://zoobank.org/9F68C01E-127B-41CA-9BB8-081867D948DB

**Figure 31.**
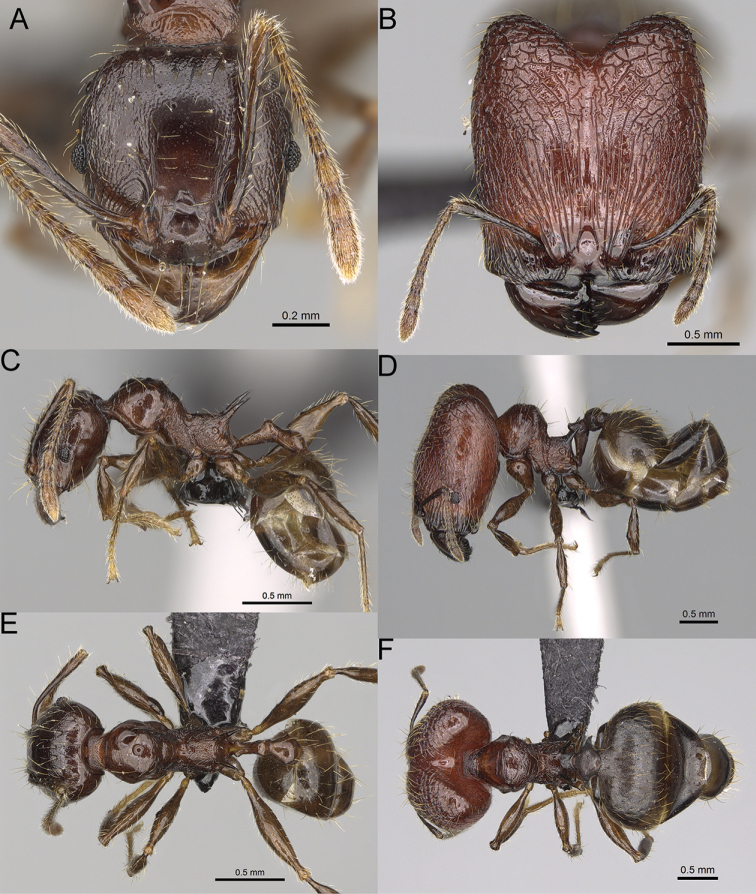
*Pheidole aelloea* sp. nov., full-face view (**A**), profile (**C**), and dorsal view (**E**) of paratype minor worker (CASENT0923221) and full-face view (**B**), profile (**D**), and dorsal view (**F**) of holotype major worker (CASENT0236213).

Figs 31A–F , 84A , 86A

###### Type material.

***Holotype*.** Madagascar. •1 major worker; Antsiranana; Makirovana Forest; -14.17066, 49.95409; alt. 415 m; 29 Apr 2011; B.L. Fisher et al. leg.; BLF26671, CASENT0236213 (CASC). ***Paratypes*.** Madagascar. •2w., 1q.; same data as for holotype; CASENT0923221, CASENT0236212, CASENT0872221 (CASC).

###### Other material.

Madagascar. –***Antsiranana***: •10w., 11s., 1m., 1q.; Ambondrobe, 41.1 km 175° Vohemar; -13.71533, 50.10167; alt. 10 m; 29 Nov 2004; B.L. Fisher leg.; CASENT0056070, CASENT0056092, CASENT0056307, CASENT0056312, CASENT0056322, CASENT0056517, CASENT0056522, CASENT0056529, CASENT0056534, CASENT0056536, CASENT0056662, CASENT0056663, CASENT0056666, CASENT0056666, CASENT0056679, CASENT0107922, CASENT0110497, CASENT0110498, CASENT0110545, CASENT0235068 (CASC). •1w., 1s.; Binara Forest; -13.26207, 49.60505; alt. 692 m; 18 Oct 2013; B.L. Fisher et al. leg.; CASENT0369418, CASENT0369423 (CASC). •16w., 5s., 1q.; Forêt Ambanitaza, 26.1 km 347° Antalaha; -14.67933, 50.18367; alt. 240 m; 26 Nov 2004; B.L. Fisher leg.; CASENT0054846, CASENT0054850, CASENT0054851, CASENT0054853, CASENT0054893, CASENT0054894, CASENT0054951, CASENT0054955, CASENT0054968, CASENT0054972, CASENT0055042, CASENT0055061, CASENT0055550, CASENT0055558, CASENT0055562, CASENT0055562, CASENT0055592, CASENT0058967, CASENT0058977, CASENT0109535 (CASC). •10w., 9s., 3q.; Forêt d’ Antsahabe, 11.4 km 275°W Daraina; -13.21167, 49.55667; alt. 550 m; 12 Dec 2003; B.L. Fisher leg.; CASENT0042268, CASENT0042329, CASENT0042334, CASENT0042335, CASENT0053689, CASENT0053690, CASENT0053936, CASENT0053937, CASENT0053941, CASENT0053942, CASENT0076806, CASENT0076807 (CASC). •21w., 1s., 2q.; Forêt de Binara, 7.5 km 230°SW Daraina; -13.255, 49.61667; alt. 375 m; 1 Dec 2003; B.L. Fisher leg.; CASENT0041716, CASENT0041724, CASENT0041734, CASENT0041742, CASENT0041795, CASENT0041797, CASENT0041800, CASENT0041805, CASENT0041808, CASENT0041811, CASENT0041815, CASENT0041823, CASENT0041828, CASENT0041885, CASENT0041887, CASENT0041889, CASENT0041897, CASENT0041902, CASENT0041906, CASENT0041907, CASENT0041909, CASENT0041909, CASENT0073337, CASENT0076448 (CASC). •1s.; Forêt de Binara, 9.1 km 233°SW Daraina; -13.26333, 49.60333; alt. 650–800 m; 19 Nov 2004; B.L. Fisher leg.; CASENT0053842 (CASC). •6w., 5s., 2q.; Galoko chain, Mont Galoko; -13.59358, 48.73157; alt. 1100 m; 22 Feb 2013; B.L. Fisher et al. leg.; CASENT0300979, CASENT0300983, CASENT0301038, CASENT0301040, CASENT0301059, CASENT0301069, CASENT0301073 (CASC). •1w., 1s.; Galoko chain, Mont Galoko; -13.58745, 48.71419; alt. 380 m; 23 Feb 2013; B.L. Fisher et al. leg.; CASENT0303019 (CASC). •5w., 3s.; Galoko chain, Mont Galoko; -13.58487, 48.71818; alt. 520 m; 19 Feb 2013; B.L. Fisher et al. leg.; CASENT0302940, CASENT0304461, CASENT0304986, CASENT0304992, CASENT0305000, CASENT0305011 (CASC). •1w., 1s.; Galoko chain, Mont Galoko; -13.5888, 48.72864; alt. 980 m; 20 Feb 2013; B.L. Fisher et al. leg.; CASENT0300190 (CASC). •2w., 1s.; Galoko chain, Mont Galoko; -13.5888, 48.72864; alt. 980 m; 20 Feb 2013; B.L. Fisher et al. leg.; CASENT0304408, CASENT0304755 (CASC). •4w., 3s.; Galoko chain, Mont Kalabenono; -13.64179, 48.67282; alt. 643 m; 10 Oct 2013; B.L. Fisher et al. leg.; CASENT0366980, CASENT0369681, CASENT0369743, CASENT0369780 (CASC). •2w.; Makirovana Forest; -14.104, 50.03574; alt. 225 m; 4 May 2011; B.L. Fisher et al. leg.; CASENT0231485, CASENT0243618 (CASC). •14w., 1s., 2q.; Makirovana Forest; -14.17066, 49.95409; alt. 415 m; 29 Apr 2011; B.L. Fisher et al. leg.; CASENT0231284, CASENT0231285, CASENT0231304, CASENT0231305, CASENT0236148, CASENT0236154, CASENT0243267, CASENT0243271, CASENT0243278, CASENT0243280, CASENT0243284, CASENT0243301, CASENT0243305, CASENT0243325 (CASC). •1w.; Makirovana Forest; -14.16044, 49.95216; alt. 550 m; 1 May 2011; B.L. Fisher et al. leg.; CASENT0230611 (CASC). •2w.; Masoala National Park; -15.3014, 50.22776; alt. 280 m; 7 Mar 2014; B.L. Fisher et al. leg.; CASENT0377714, CASENT0377725 (CASC). •2w.; Masoala National Park; -15.32331, 50.30751; alt. 60 m; 10 Mar 2014; B.L. Fisher et al. leg.; CASENT0376961, CASENT0378012 (CASC). •11w., 18s.; Parc National de Marojejy, Manantenina River, 28.0 km 38°NE Andapa, 8.2 km 333°NNW Manantenina; -14.43667, 49.775; alt. 450 m; 12 Nov 2003; B.L. Fisher et al. leg.; CASENT0045743, CASENT0045810, CASENT0045818, CASENT0045826, CASENT0045846, CASENT0045866, CASENT0045869, CASENT0045895, CASENT0045901, CASENT0045909, CASENT0045979, CASENT0046010, CASENT0046075, CASENT0077158, CASENT0077159, CASENT0077160, CASENT0077161, CASENT0077162, CASENT0077163 (CASC). •1s.; R.S. Manongarivo, 14.5 km 220°SW Antanambao; -13.99833, 48.42833; alt. 1175 m; 20 Oct 1998; B.L. Fisher leg.; CASENT0198835 (CASC). •2w., 1s.; Réserve Spéciale d’Ambre, 3.5 km 235°SW Sakaramy; -12.46889, 49.24217; alt. 325 m; 26 Jan 2001; B.L. Fisher et al. leg.; CASENT0423920, CASENT0423924, CASENT0484626 (CASC). •1w., 1s.; Sava Region: Parc National de Marojejy, Manantenina River, 28.1 km 25.7NE Andapa; -14.43553, 49.76463; alt. 680 m; 11 Feb 2018; B.L. Fisher et al. leg.; CASENT0809603 (CASC). •1w.; Sava Region: Parc National de Marojejy, near Manantenina River; -14.43677, 49.77541; alt. 475 m; 7 Feb 2018; B.L. Fisher et al. leg.; CASENT0825163 (CASC). •3w., 1s., 1m.; Sava Region: Parc National de Marojejy, near Manantenina tributary, 28.3 km 28.5NE Andapa, forest along trail below Camp 1; -14.43934, 49.77689; alt. 450 m; 8 Feb 2018; B.L. Fisher et al. leg.; CASENT0825240, CASENT0825241, CASENT0825254 (CASC). •1w., 1q.; Sava Region: Parc National de Marojejy, near summit, 25.4 km 20.1NE Andapa; -14.44918, 49.73243; alt. 2100 m; 10 Feb 2018; B.L. Fisher et al. leg.; CASENT0809554 (CASC). –***Toamasina***: •1q.; 6.3 km S Ambanizana, Andranobe; -15.6813, 49.958; alt. 25 m; 14 Nov 1993; B.L. Fisher leg.; CASENT0198118 (CASC).

###### Diagnosis.

***Major workers*.** Body size moderate: HL: 1.85–2.45 (2.0), HW: 1.56–2.02 (1.64), WL: 1.17–1.35 (1.25); propodeal spines very long (PSL: 0.38–0.48 (0.42)); head in full-face view rectangular, with lateral sides relatively straight, only their posteriormost part slightly convex; sides of the head with sparse, relatively long, erect pilosity; occipital lobes shiny, with sparse and thick rugoreticulation; inner hypostomal teeth distinct, triangular, and moderately thin, with rounded apex, closely spaced; outer hypostomal teeth distinct, approximately as high as inner hypostomal teeth, lobe-like, with base wider than inner hypostomal teeth, inner and outer teeth closely spaced and connected by indistinct concavity. ***Minor workers*.** Body size moderate: HL: 0.63–0.78 (0.68), HW: 0.61–0.79 (0.67), WL: 0.83–0.98 (0.9); propodeal spines very long (PSL: 0.3–0.37 (0.32)); scape, when laid back, surpassing posterior head margin by one-fifth of its length; lateral sides of head and malar area finely rugulose, sculpture weakening posteriorly; vertex, genae, and frons smooth; pronotum, dorsal surface of mesonotum, and dorsal and posterior surface of propodeum smooth, sometimes propodeum with indistinct, sparse rugulae; katepisternum, anepisternum, and lateral sides of propodeum with thick and dense rugo-punctae.

###### Description.

**Major workers.** Measurements (*N* = 10): HL: 1.85–2.45 (2.0); HW: 1.56–2.02 (1.64); SL: 0.73–0.89 (0.77); EL: 0.14–0.18 (0.16); WL: 1.17–1.35 (1.25); PSL: 0.38–0.48 (0.42); MTL: 0.67–0.89 (0.75); PNW: 0.7–0.8 (0.73); PTW: 0.2–0.22 (0.2); PPW: 0.59–0.7 (0.66); CI: 79.6–85.9 (83.2); SI: 44.1–49.3 (46.9); PSLI: 19.8–25.0 (21.5); PPI: 28.3–34.5 (31.0); PNI: 39.5–46.8 (44.8); MTI: 41.8–50.6 (45.8). ***Head*.** In full-face view rectangular, lateral sides relatively straight, only their posteriormost part slightly convex (Fig. 31B). In lateral view oval; ventral and dorsal faces finely convex; inner hypostomal teeth visible. Sides of the head with sparse, relatively long, erect pilosity; whole head with sparse, relatively long, erect pilosity. Antennal scrobes indistinct and not delimited by carinulae. Occipital lobes shiny, with sparse and thick rugoreticulation; genae shiny, with fine and sparse rugoreticulation, sometimes posterior part with reduced sculpture; malar area and frons with longitudinal and sparse rugae, on posterior part of frons rugae directed outward and more irregular, interspaces smooth; lateral sides of head shiny, with irregular, moderately dense and thick rugoreticulation. Centre of clypeus smooth and shiny, lateral sides with longitudinal rugae; median notch present, moderately wide, and narrow; median longitudinal carina present; lateral longitudinal carinae absent. Scape, when laid back, reaching mid-length of head; pilosity suberect to erect (Fig. 31B, D). Inner hypostomal teeth distinct, triangular, and moderately thin, with rounded apex, closely spaced; outer hypostomal teeth distinct, approximately as high as inner hypostomal teeth, lobe-like, with base wider than inner hypostomal teeth, inner and outer teeth closely spaced and connected by indistinct concavity (Fig. 84A). ***Mesosoma*.** In lateral view, promesonotum short, angular, and high, with slightly convex posterior declivity; promesonotal groove absent; metanotal groove absent; propodeal spines very long, narrow, massive basally, with acute apex; humeral area laterally absent to weakly produced (Fig. 31D). Surface shiny, pronotum, dorsal surface of mesonotum, and dorsal and posterior surface of propodeum with fine, sparse to moderately sparse rugoreticulation, sometimes sculpture on propodeum strongly reduced and absent on its dorsal surface; katepisternum, anepisternum, and lateral sides of propodeum with thick and sparse rugoreticulation. Pilosity moderately sparse, long, and erect (Fig. 31D, F). ***Petiole*.** Shagreened; peduncle relatively long, with triangular apex, and thick horizontal lobes on its basal part; node triangular with rounded apex, in rear view node dorsoventrally slightly depressed; pilosity moderately sparse and erect (Fig. 31D, F). ***Postpetiole*.** Shagreened; in dorsal view sides with acute, moderately long, relatively narrow, and angular projections; pilosity long, moderately sparse, and erect (Fig. 31D, F). ***Petiole*.** First gastral tergite shagreened, at least on its basal part; pilosity moderately sparse, long, and erect (Fig. 31D, F). ***Colour*.** Unicolourous, ocherous to reddish-brown, sometimes head brighter than the rest of the body (Fig. 31D, F).

**Minor workers.** Measurements (*N* = 10): HL: 0.63–0.78 (0.68); HW: 0.61–0.79 (0.67); SL: 0.66–0.8 (0.7); EL: 0.09–0.13 (0.11); WL: 0.83–0.98 (0.9); PSL: 0.3–0.37 (0.32); MTL: 0.52–0.66 (0.57); PNW: 0.39–0.49 (0.42); PTW: 0.09–0.14 (0.1); PPW: 0.15–0.25 (0.19); CI: 95.9–102.7 (98.3); SI: 100.5–119.0 (105.9); PSLI: 43.0–49.5 (46.7); PPI: 48.5–64.9 (54.3); PNI: 60.3–65.6 (63.0); MTI: 83.3–88.1 (85.5). ***Head*.** In full-face view square, posterior of eyes slightly convex, anterior of eyes relatively straight, occipital margin straight or indistinctly convex; occipital carina indistinct, weakly developed (Fig. 31A). Pilosity sparse, long, and erect. Lateral sides of head and malar area finely rugulose, sculpture weakening posteriorly; vertex, genae, and frons smooth; antennal sockets with sparse carinae curved outward. Clypeus with median longitudinal carina present; two lateral longitudinal carinae present. Scape, when laid back, surpassing posterior head margin by one-fifth of its length; pilosity suberect to erect (Fig. 31A, C). ***Mesosoma*.** In lateral view, promesonotum high, short, and convex; promesonotal groove absent; metanotal groove absent; propodeal spines very long, massive basally, with acute apex (Fig. 31C). Surface shiny, pronotum, dorsal surface of mesonotum, and dorsal and posterior surface of propodeum smooth, sometimes propodeum with indistinct, sparse rugulae; katepisternum, anepisternum, and lateral sides of propodeum with thick and dense rugose punctae. Pilosity sparse, long, and erect (Fig. 31C, E). ***Petiole*.** Vertex of peduncle finely shagreened; peduncle long and moderately thin; node low, bulge-like; with few long, erect setae (Fig. 31C, E). ***Postpetiole*.** Moderately long, low, and slightly convex; with few long, erect setae at the anterior edge (Fig. 31C, E). ***Petiole*.** With few long, erect setae (Fig. 31C, E). ***Colour*.** Unicolourous, brown to dark brown (Fig. 31C, E).

###### Etymology.

Named after Aello, a harpy from Greek mythology, in reference to the long, sharp propodeal spines of minor workers reminiscent of claws.

###### Biology.

The species was collected between 10–2100 m in elevation, in rainforest, tropical dry forest, littoral rainforest, montane rainforest, and montane shrubland. Nests were located in rotten logs, rotten sticks on ground, dead twigs above ground, and soil.

###### Comments.

This species is most similar to *P. ocypodea* sp. nov. and *P. podargea* sp. nov. ***Major workers*.***Pheidole aelloea* sp. nov. can be distinguished from *P. ocypodea* sp. nov. by slightly convex posterior declivity of promesonotum and inner and outer hypostomal teeth not pointed inward or outward; it differs from *P. podargea* sp. nov. by longer and sparser pilosity on sides of head and reduced to absent sculpture on genae and propodeum. ***Minor workers*.***Pheidole aelloea* sp. nov. can be distinguished from *P. ocypodea* sp. nov. by finely rugulose and never smooth lateral sides of head and malar area, absence of metanotal groove, long petiolar peduncle, and short postpetiole which is approximately as long as high; it differs from *P. podargea* sp. nov. in smooth sculpture of vertex, genae, and area between frontal carinae, absence of promesonotal and metanotal groove, and at least partially smooth surface of pronotum, dorsal surface of mesonotum, and dorsal and posterior surface of propodeum.

##### Pheidole ocypodea sp. nov.

Taxon classificationAnimaliaHymenopteraFormicidae

http://zoobank.org/C9F6D256-6F79-4517-90A2-FFA380105D9D

**Figure 32.**
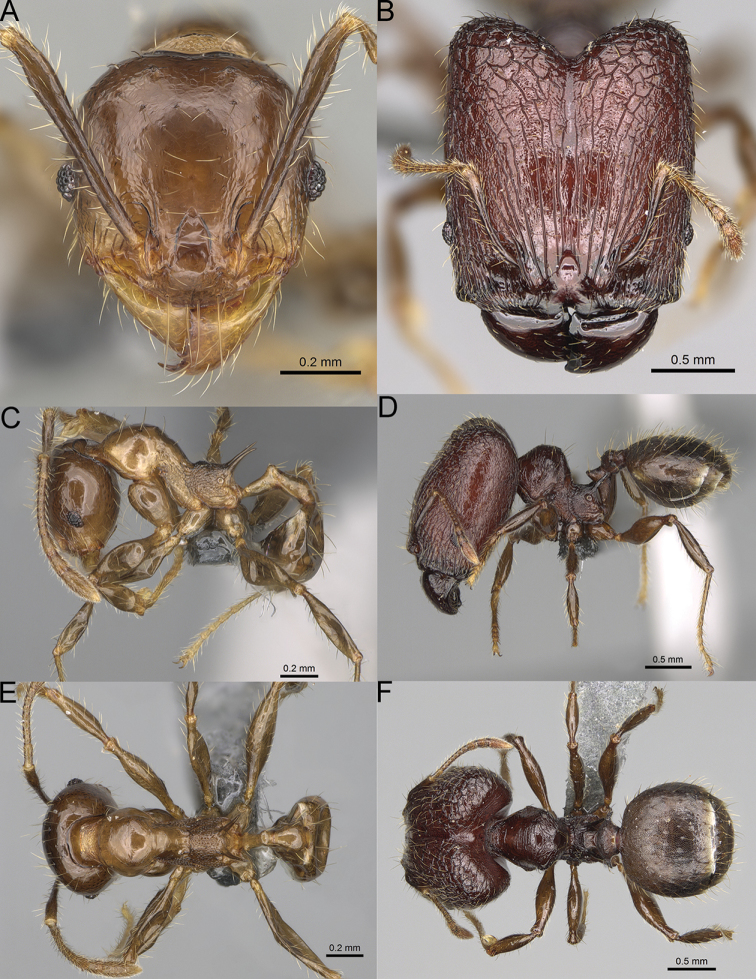
*Pheidole ocypodea* sp. nov., full-face view (**A**), profile (**C**), and dorsal view (**E**) of paratype minor worker (CASENT0923220) and full-face view (**B**), profile (**D**), and dorsal view (**F**) of holotype major worker (CASENT0304390).

Figs 32A–F , 85O , 87T

###### Type material.

***Holotype*.** Madagascar. •1 major worker; Antsiranana; Galoko chain, Mont Galoko; -13.5888, 48.72864; alt. 980 m; 20 Feb 2013; B.L. Fisher et al. leg.; BLF30960, CASENT0304390 (CASC). ***Paratype*.** Madagascar. •1 minor worker; same data as for holotype; CASENT0923220 (CASC).

###### Other material.

Madagascar. –***Antsiranana***: •8w., 11s.; R.S. Manongarivo, 10.8 km 229°SW Antanambao; -13.96167, 48.43333; alt. 400 m; 8 Nov 1998; B.L. Fisher leg.; CASENT0198625, CASENT0198626, CASENT0198834, CASENT0198836, CASENT0846551–CASENT0846562 (CASC). •32w., 24s.; R.S. Manongarivo, 12.8 km 228°SW Antanambao; -13.97667, 48.42333; alt. 780 m; 11 Sep 1998; B.L. Fisher leg.; CASENT0196883, CASENT0198627, CASENT0198837, CASENT0846501–CASENT0846550 (CASC).

###### Diagnosis.

***Major workers*.** Body size moderate: HL: 1.82–2.01 (1.95); HW: 1.52–1.66 (1.6), WL: 1.16–1.31 (1.26); propodeal spines very long (PSL: 0.38–0.45 (0.41)); head in full-face view rectangular, with lateral sides relatively straight, only their posteriormost part slightly convex; sides of the head with sparse, relatively long, erect pilosity; occipital lobes shiny, with sparse and thick rugoreticulation; inner hypostomal teeth distinct, low, closely spaced, triangular, with rounded apex directed outward; outer hypostomal teeth distinct, low, lobe-like, with base wide and tops directed inward; inner and outer teeth closely spaced and connected by concavity. ***Minor workers*.** Body size moderate: HL: 0.63–0.72 (0.66); HW: 0.6–0.7 (0.64), WL: 0.8–0.93 (0.85); propodeal spines very long (PSL: 0.26–0.32 (0.28)); scape, when laid back, surpassing posterior head margin by two-fifths of its length; lateral sides of head and malar area smooth and shiny or with indistinct, sparse rugulae, sculpture weakening posteriorly; vertex, genae, and frons smooth; pronotum, dorsal surface of mesonotum, and dorsal and posterior surface of propodeum smooth; katepisternum, anepisternum, and lateral sides of propodeum with thick and sparse rugae.

###### Description.

**Major workers.** Measurements (*N* = 10): HL: 1.82–2.01 (1.95); HW: 1.52–1.66 (1.6); SL: 0.71–0.8 (0.75); EL: 0.15–0.19 (0.17); WL: 1.16–1.31 (1.26); PSL: 0.38–0.45 (0.41); MTL: 0.69–0.79 (0.75); PNW: 0.68–0.78 (0.75); PTW: 0.18–0.25 (0.21); PPW: 0.62–0.73 (0.65); CI: 79.8–84.5 (82.3); SI: 44.5–50.1 (46.8); PSLI: 20.3–22.7 (21.3); PPI: 28.0–37.7 (32.4); PNI: 44.7–48.9 (46.6); MTI: 44.5–48.3 (46.5). ***Head*.** In full-face view rectangular, with lateral sides relatively straight, only their posteriormost part slightly convex (Fig. 32B). In lateral view oval; ventral and dorsal faces convex; inner hypostomal teeth visible. Sides of the head with sparse, relatively long, erect pilosity; whole head with moderately dense, relatively long, erect pilosity. Antennal scrobes indistinct and not delimited by carinulae. Occipital lobes shiny, with sparse and thick rugoreticulation; genae smooth and shiny, only anterior part sometimes with sparse and fine rugulae; frons with longitudinal and sparse rugae, on posterior part of frons rugae directed outward and more irregular, interspaces with sparse to moderately sparse rugose foveolae; malar area and lateral sides of head shiny, with longitudinal rugae, surface between rugae with dense rugose foveolae. Centre of clypeus smooth and shiny, lateral sides with longitudinal rugae; median notch present, moderately wide, and narrow; median longitudinal carina present; lateral longitudinal carinae absent. Scape, when laid back, reaching midlength of head; pilosity suberect to erect (Fig. 32B, D). Inner hypostomal teeth distinct, low, closely spaced, triangular, with rounded apex directed outward; outer hypostomal teeth distinct, low, lobe-like, with base wide and tops directed inward; inner and outer teeth closely spaced and connected by concavity (Fig. 85O). ***Mesosoma*.** In lateral view, promesonotum short, angular, and high, with relatively steep posterior declivity; promesonotal groove absent; metanotal groove absent; propodeal spines very long, narrow, with acute apex; humeral area laterally absent to weakly produced (Fig. 32D). Surface shiny, pronotum and dorsal surface of mesonotum smooth or sometimes with indistinct, sparse rugulae; propodeum, katepisternum, and anepisternum with thick and dense rugoreticulation. Pilosity sparse, long, and erect (Fig. 32D, F). ***Petiole*.** Shagreened; peduncle relatively long, without horizontal lobes on its basal part; node triangular and thin, with rounded apex, in rear view node dorsoventrally slightly depressed; pilosity moderately sparse and erect (Fig. 32D, F). ***Postpetiole*.** Shagreened; in dorsal view sides with acute, horn-like, moderately long projections; pilosity long, moderately sparse, and erect (Fig. 32D, F). ***Petiole*.** First gastral tergite shagreened, at least on its basal part; pilosity moderately dense, long, and erect (Fig. 32D, F). ***Colour*.** Unicolourous, ocherous to reddish-brown (Fig. 32D, F).

**Minor workers.** Measurements (*N* = 10): HL: 0.63–0.72 (0.66); HW: 0.6–0.7 (0.64); SL: 0.63–0.69 (0.66); EL: 0.09–0.13 (0.11); WL: 0.8–0.93 (0.85); PSL: 0.26–0.32 (0.28); MTL: 0.49–0.57 (0.53); PNW: 0.38–0.45 (0.42); PTW: 0.09–0.12 (0.1); PPW: 0.18–0.24 (0.19); CI: 93.6–98.2 (96.3); SI: 98.3–106.9 (102.9); PSLI: 40.4–45.5 (42.6); PPI: 45.1–57.6 (52.4); PNI: 62.5–68.1 (65.2); MTI: 78.9–86.9 (83.3). ***Head*.** In full-face view square, posterior of eyes slightly convex, anterior of eyes relatively straight, occipital margin straight or indistinctly convex; occipital carina indistinct, weakly developed (Fig. 32A). Pilosity sparse, long, and erect. Lateral sides of head and malar area smooth and shiny or with indistinct, sparse rugulae, sculpture weakening posteriorly; vertex, genae, and frons smooth; antennal sockets with sparse, interrupted carinae curved outward. Clypeus with median longitudinal carina present; two lateral longitudinal carinae present. Scape, when laid back, surpassing posterior head margin by two-fifths of its length; pilosity suberect to erect (Fig. 32A, C). ***Mesosoma*.** In lateral view, promesonotum high, short, and convex; promesonotal groove absent; metanotal groove shallow and indistinct; propodeal spines very long, massive basally, with acute apex (Fig. 32C). Surface shiny, pronotum, dorsal surface of mesonotum, and dorsal and posterior surface of propodeum smooth; katepisternum, anepisternum, and lateral sides of propodeum with thick and sparse rugae. Pilosity sparse, long, and erect (Fig. 32C, E). ***Petiole*.** Peduncle moderately long and thin; node low, bulge-like; with few long, erect setae (Fig. 32C, E). ***Postpetiole*.** Long, low, and slightly convex; with few long, erect setae at the anterior edge (Fig. 32C, E). ***Petiole*.** Pilosity sparse and erect (Fig. 32C, E). ***Colour*.** Unicolourous, bright brown to brown (Fig. 32C, E).

###### Etymology.

Named after Ocypode, a harpy from Greek mythology, in reference to the long and sharp propodeal spines of minor workers reminiscent of claws.

###### Biology.

The species was collected between 400–980 m in elevation, in rainforest and montane rainforest. Nests were located in in rotten logs and rotten sticks on ground.

###### Comments.

This species is most similar to *P. aelloea* sp. nov. and *P. podargea* sp. nov. ***Major workers*.***Pheidole ocypodea* sp. nov. can be distinguished from *P. aelloea* sp. nov. by steep to relatively steep posterior declivity of promesonotum, inner hypostomal teeth pointed outward and outer hypostomal teeth pointed inward; from *P. podargea* sp. nov. it differs in longer and sparser pilosity on sides of head, reduced to absent sculpture on genae and propodeum, and inner hypostomal teeth pointed outward. ***Minor workers*.***Pheidole ocypodea* sp. nov. can be distinguished from *P. aelloea* sp. nov. by smooth lateral sides of head and malar area, presence of metanotal groove, short petiolar peduncle, and long postpetiole which is approximately 1.5 times longer than high; from *P. podargea* sp. nov. it differs in smooth sculpture of vertex, genae, and area between frontal carinae, absence of promesonotal groove and at least partially smooth surface of pronotum, dorsal surface of mesonotum, and dorsal and posterior surface of propodeum.

##### Pheidole podargea sp. nov.

Taxon classificationAnimaliaHymenopteraFormicidae

http://zoobank.org/9A9D09F6-79B6-40AA-BB1E-BE65E6DC3FB3

**Figure 33.**
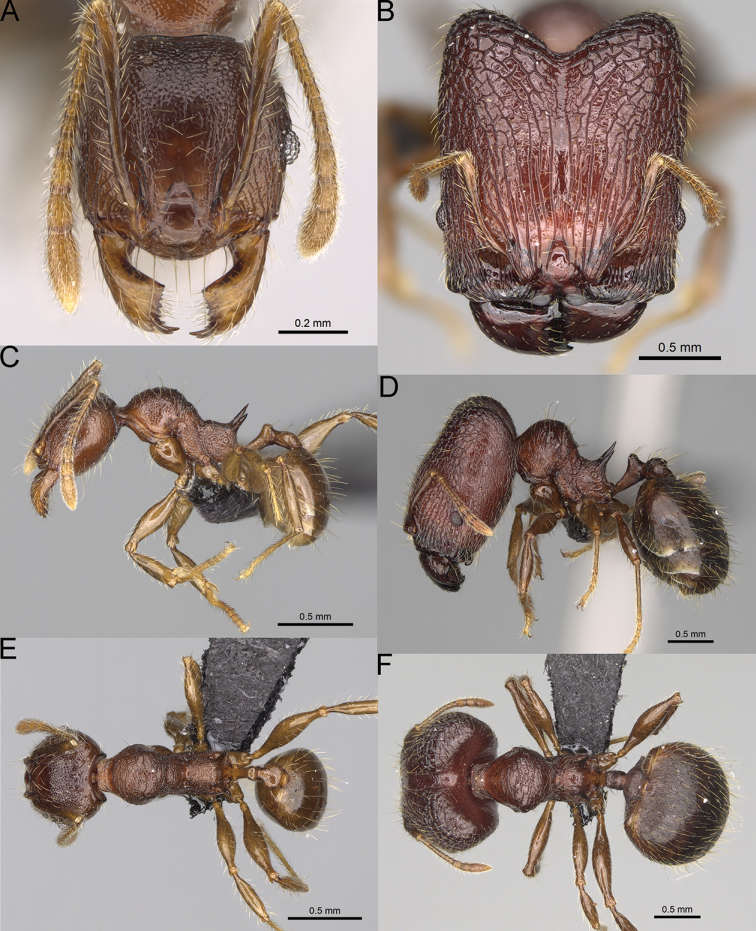
*Pheidole podargea* sp. nov., full-face view (**A**), profile (**C**), and dorsal view (**E**) of paratype minor worker (CASENT0487567) and full-face view (**B**), profile (**D**), and dorsal view (**F**) of holotype major worker (CASENT0487570).

Figs 33A–F , 85R , 88B

###### Type material.

***Holotype*.** Madagascar. •1 major worker; Antsiranana; Forêt d’ Andavakoera, 21.4 km 75°ENE Ambilobe; 4.6 km 356°N Betsiaka; -13.11833, 49.23; alt. 425 m; 16 Dec 2003; B.L. Fisher leg.; BLF10317, CASENT0487570, middle specimen on the pin (CASC). ***Paratypes*.** Madagascar. •9w., 8s.; same data as for holotype; CASENT0487566, CASENT0872085, CASENT0487569, CASENT0487568, CASENT0487567 (CASC).

###### Other material.

Madagascar. –***Antsiranana***: •9w., 4s.; Forêt de Binara, 7.5 km 230°SW Daraina; -13.255, 49.61667; alt. 375 m; 1 Dec 2003; B.L. Fisher leg.; CASENT0041888, CASENT0043218, CASENT0043224, CASENT0043226, CASENT0043235, CASENT0043241, CASENT0043253, CASENT0043256, CASENT0043259, CASENT0043317, CASENT0043319, CASENT0043331, CASENT0043402 (CASC). •5w., 1s., 2q.; Réserve Spéciale d’Ambre, 3.5 km 235°SW Sakaramy; -12.46889, 49.24217; alt. 325 m; 26 Jan 2001; Fisher et al. leg.; CASENT0406663, CASENT0406673, CASENT0423867, CASENT0427613, CASENT0427696, CASENT0427699 (CASC). •32w., 9s., 3q.; Réserve Spéciale de l’Ankarana, 13.6 km 192°SSW Anivorano Nord; -12.86361, 49.22583; alt. 210 m; 16 Feb 2001; Fisher et al. leg.; CASENT0440586, CASENT0440686, CASENT0440729, CASENT0440730, CASENT0440731, CASENT0440735, CASENT0440736, CASENT0440739, CASENT0440740, CASENT0440743, CASENT0440745, CASENT0440746, CASENT0440748, CASENT0440750, CASENT0440752, CASENT0440754, CASENT0440755, CASENT0440756, CASENT0440758, CASENT0440762–CASENT0440765, CASENT0440769, CASENT0440771, CASENT0440773, CASENT0440774, CASENT0440776, CASENT0440780, CASENT0440782, CASENT0440786–CASENT0440788, CASENT0440790, CASENT0440792, CASENT0440803, CASENT0440808, CASENT0440813, CASENT0441204–CASENT0441206, CASENT0441208, CASENT0441211, CASENT0441212, CASENT0441214, CASENT0441216 (CASC).

###### Diagnosis.

***Major workers*.** Body size moderate: HL: 1.85–2.0 (1.9), HW: 1.55–1.65 (1.59), WL: 1.19–1.35 (1.26); propodeal spines very long (PSL: 0.39–0.46 (0.42)); head in full-face view rectangular, with lateral sides relatively straight, only their posteriormost part slightly convex; sides of the head with moderately dense, moderately long, erect pilosity; occipital lobes shiny, with sparse and thick rugoreticulation; inner hypostomal teeth distinct, closely spaced, triangular, with rounded apex; outer hypostomal teeth distinct, as high as inner hypostomal teeth, lobe-like, with base wide and tops directed slightly inward; inner and outer teeth closely spaced and connected by concavity. ***Minor workers*.** Body size moderate: HL: 0.61–0.7 (0.66), HW: 0.62–0.69 (0.65), WL: 0.81–0.9 (0.86); propodeal spines very long (PSL: 0.25–0.31 (0.28)); scape, when laid back, surpassing posterior head margin by two-fifths of its length; whole head with fine and dense rugo-punctuation, only basal area of frons and genae with sculpture reduced or sometimes absent; mesosoma rugo-punctate, sometimes sculpture weakening on dorsal surface and pronotum.

###### Description.

**Major workers.** Measurements (*N* = 10): HL: 1.85–2.0 (1.9); HW: 1.55–1.65 (1.59); SL: 0.67–0.76 (0.72); EL: 0.16–0.2 (0.17); WL: 1.19–1.35 (1.26); PSL: 0.39–0.46 (0.42); MTL: 0.7–0.76 (0.73); PNW: 0.71–0.81 (0.76); PTW: 0.15–0.22 (0.19); PPW: 0.59–0.71 (0.66); CI: 80.1–87.6 (83.4); SI: 42.6–47.7 (45.6); PSLI: 20.9–24.4 (21.8); PPI: 25.4–32.7 (29.1); PNI: 45.5–51.0 (48.2); MTI: 42.6–48.8 (46.4). ***Head*.** In full-face view rectangular, with lateral sides relatively straight, only their posteriormost part slightly convex (Fig. 33B). In lateral view oval; ventral and dorsal faces convex; inner hypostomal teeth visible. Sides of the head with moderately dense, moderately long, erect pilosity. Antennal scrobes indistinct and not delimited by carinulae. Occipital lobes shiny, with sparse and thick rugoreticulation; genae shiny, with moderately dense to dense, fine rugoreticulation; frons with longitudinal, sparse to moderately sparse rugae, on posterior part of frons rugae directed outward and more irregular, interspaces with sparse rugulae, sometimes indistinct; malar area and lateral sides of head shiny, with longitudinal rugoreticulation, surface between rugae with sparse and indistinct rugulae. Centre of clypeus smooth and shiny or with fine longitudinal rugulae, lateral sides with longitudinal rugae; median notch present, wide, and shallow; median longitudinal carina present but sometimes indistinct; lateral longitudinal carinae absent. Scape, when laid back, reaching midlength of head; pilosity suberect to erect (Fig. 33B, D). Inner hypostomal teeth distinct, closely spaced, triangular, with rounded apex; outer hypostomal teeth distinct, as high as inner hypostomal teeth, lobe-like, with base wide and tops directed slightly inward; inner and outer teeth closely spaced and connected by concavity (Fig. 85R). ***Mesosoma*.** In lateral view, promesonotum short, angular, and high, with slightly convex posterior declivity; promesonotal groove absent; metanotal groove absent; propodeal spines very long, massive basally, with acute apex; humeral area laterally weakly produced (Fig. 33D). Surface shiny, with fine, dense rugoreticulation, sometimes sculpture weakening on dorsum. Pilosity sparse, long, and erect (Fig. 33D, F). ***Petiole*.** Shagreened; peduncle relatively long, with small tooth-like horizontal lobes on its basal part; node triangular, with rounded apex, in rear view node convex; pilosity moderately sparse and erect (Fig. 33D, F). ***Postpetiole*.** Shagreened; in dorsal view sides with acute, horn-like, moderately long projections; pilosity long, moderately sparse, and erect (Fig. 33D, F). ***Petiole*.** First gastral tergite shagreened, at least on its basal part; pilosity moderately dense, long, and erect (Fig. 33D, F). ***Colour*.** Unicolourous, ocherous to reddish-brown (Fig. 33D, F).

**Minor workers.** Measurements (*N* = 10): HL: 0.61–0.7 (0.66); HW: 0.62–0.69 (0.65); SL: 0.63–0.7 (0.67); EL: 0.11–0.12 (0.11); WL: 0.81–0.9 (0.86); PSL: 0.25–0.31 (0.28); MTL: 0.49–0.58 (0.53); PNW: 0.39–0.45 (0.43); PTW: 0.09–0.11 (0.1); PPW: 0.17–0.21 (0.19); CI: 94.3–100.7 (97.2); SI: 97.5–107.9 (103.1); PSLI: 38.8–44.3 (41.7); PPI: 46.9–56.9 (51.6); PNI: 63.4–69.7 (66.2); MTI: 78.4–85.0 (82.3). ***Head*.** In full-face view square, posterior of eyes slightly convex, anterior of eyes relatively straight, occipital margin straight or indistinctly convex; occipital carina indistinct, weakly developed (Fig. 33A). Pilosity moderately dense, long, and erect. Whole head with fine and dense rugo-punctuation, only basal area of frons and genae with sculpture reduced or sometimes absent; antennal sockets with dense carinae curved outward. Clypeus with median longitudinal carina present; two lateral longitudinal carinae absent. Scape, when laid back, surpassing posterior head margin by two-fifths of its length; pilosity suberect to erect (Fig. 33A, C). ***Mesosoma*.** In lateral view, promesonotum high, short, and convex; promesonotal groove present, indistinct; metanotal groove shallow and indistinct; propodeal spines very long, massive basally, with acute apex (Fig. 33C). Whole surface rugo-punctate, sometimes sculpture weakening on dorsal surface and pronotum. Pilosity sparse, long, and erect (Fig. 33C, E). ***Petiole*.** Peduncle relatively long and thin; node low, triangular; with few long, erect setae (Fig. 33C, E). ***Postpetiole*.** Short, low, and slightly convex; with few long, erect setae at the anterior edge (Fig. 33C, E). ***Petiole*.** With few long, erect setae (Fig. 33C, E). ***Colour*.** Unicolourous, bright brown to brown (Fig. 33C, E).

###### Etymology.

Named after Podarge, a harpy from Greek mythology, in reference to long and sharp propodeal spines of minor workers reminiscent of claws.

###### Biology.

The species was collected between 210–800 m in elevation, in rainforest and tropical dry forest. Nests were located in rotten logs.

###### Comments.

This species is most similar to *P. aelloea* sp. nov. and *P. ocypodea* sp. nov. ***Major workers*.***Pheidole podargea* sp. nov. can be distinguished from *P. aelloea* sp. nov. and *P. ocypodea* sp. nov. by outer hypostomal teeth approximately the same size as the inner ones and lack of smooth sculpture on genae and propodeum. ***Minor workers*.***Pheidole podargea* sp. nov. can be distinguished from *P. aelloea* sp. nov. and *P. ocypodea* sp. nov. by smooth area limited to basal area of frons and genae or the whole head finely rugo-punctate, presence of promesonotal and metanotal groove, and absence of smooth surfaces on mesosoma.

#### Revision of the *Pheidole ferruginea* group

**Diagnosis. *Major workers*.** Head in full-face view cordate or sub-oval, widened posteriorly, in lateral view oval to sub-oval, ventral and dorsal faces strongly convex, dorsal face not depressed posteriorly; antennal scrobes developed, well delimited by carinulae (except *P. longipilosa*); occipital lobes with sparse and irregular rugoreticulation; promesonotum short, angular, and low to high; promesonotal and metanotal grooves absent; propodeal spines moderately long to long, with base wide or narrow; mesosoma predominately sculptured sometimes with smooth notches; first gastral tergite smooth or shagreened, at least on its basal part; body bright brown to brown. ***Minor workers*.** Whole head foveolate, sometimes with additional longitudinal rugae on frons; scape, when laid back, surpassing posterior head margin by one- to two-fifths of its length; promesonotum box-like or convex; propodeal spines short, moderately long or very long; promesonotal and metanotal grooves absent or present; mesosoma predominately foveolate, sometimes with additional rugae on mesosomal dorsum; body yellow to brown.

**Comments.** Major workers of this group can be distinguished based on the combination of following characters: cordate to sub-oval head in lateral view, oval to sub-oval in dorsal view; well-developed antennal scrobes delimited by carinulae (except *P. longipilosa*); never smooth occipital lobes; lack of promesonotal and metanotal grooves; moderately long to long propodeal spines; strong sculpture of mesosoma, occasionally with smooth notches and bright brown to brown body. Minor workers can be separated entirely foveolate head, sometimes with additional longitudinal rugae on frons; short scape surpassing posterior head margin by one- to two-fifths of its length; and moderately long or very long propodeal spines (except *P. longipilosa*).

The group is divided into two complexes. The *P. ferruginea* complex includes four species: *P. ferruginea* sp. nov., *P. rugocephala* sp. nov., *P. vohemarensis* sp. nov., and *P. manantenensis* sp. nov. *Pheidole ferruginea* is relatively common across the evergreen forest biome and is sympatric with the remaining three members of the complex. The distribution range of *P. rugocephala* sp. nov., *P. vohemarensis* sp. nov., and *P. manantenensis* sp. nov. is predominately limited to the Antsiranana prefecture. *Pheidole rugocephala* is known from the evergreen forest biome located between Toamasina and Andapa, while *P. vohemarensis*, known from the same biome, inhabits area between Ambinaelo and Antsirabe. *Pheidole vohemarensis* sp. nov. is known from area spread between Andapa and Antisianana and its distribution predominately covers the dry deciduous forest biome. *Pheidole longipilosa* creates a single-species complex and is known from Forêt Classée d’Analavelona and Parc National d’Isalo in the Toliara prefecture.

##### Key to the *Pheidole ferruginea* group.

**Table:** 

1	Major workers. Head in full-face view sub-oval; sides of head with dense, very long, erect pilosity; antennal scrobes indistinct and not delimited by carinulae; propodeal spines triangular, with wide base (Fig. 34O). Minor workers. Propodeal spines short (Fig. 34P)	***P. longipilosa* sp. nov.**
–	Major workers. Head in full-face view cordate, widening posteriorly; sides of head never with dense, very long, erect pilosity; antennal scrobes developed, well delimited by carinulae; propodeal spines thin, with narrow base (Fig. 34A–D). Minor workers. Propodeal spines needle-like, moderately long to very long (Fig. 34I–L)	**2**
2	Major workers. Antennal scrobes never with foveolate surface; outer hypostomal teeth pointed inward (Fig. 34A, E). Minor workers. Propodeal spines long, promesonotum flat, long, and box-like (Fig. 34I)	***P. ferruginea* sp. nov.**
–	Major workers. Antennal scrobes with foveolate surface; outer hypostomal teeth pointed outward (Fig. 34B–D, F–H). Minor workers. Propodeal spines shorter, if long then promesonotum short, higher, and not box-like (Fig. 34J–L)	**3**
3	Major workers. Antennal scrobes deep, predominantly foveolate; frons with thick, sparse, longitudinal to irregular rugae, interspaces predominantly smooth (Fig. 34B). Minor workers. Propodeal spines moderately long (Fig. 34J)	***P. rugocephala* sp. nov.**
–	Major workers. Antennal scrobes shallow, foveolate with additional sculpture; frons with longitudinal rugae and never smooth interspaces (Fig. 34C, D). Minor workers. Propodeal spines long (Fig. 34K, L)	**4**
4	Major workers. Antennal scrobes foveolate with additional longitudinal rugae, frons with area between rugae with fine and dense rugulae, sometimes rugulae fading on the central part of frons, petiolar peduncle with wide and distinct horizontal lobes (Fig. 34C, M). Minor workers. Promesonotum with posterior declivity relatively steep, katepisternum and mesonotum with smooth notches (Fig. 34K)	***P. vohemarensis* sp. nov.**
–	Major workers. Antennal scrobes foveolate with additional irregular and indistinct rugae, frons with interspaces superficially foveolate, petiolar peduncle with shorter and less distinct horizontal lobes (Fig. 34D, N). Minor workers. Promesonotum with posterior declivity smoothly declining towards propodeum, katepisternum and mesonotum foveolate (Fig. 34L)	***P. manantenensis* sp. nov.**

**Figure 34.**
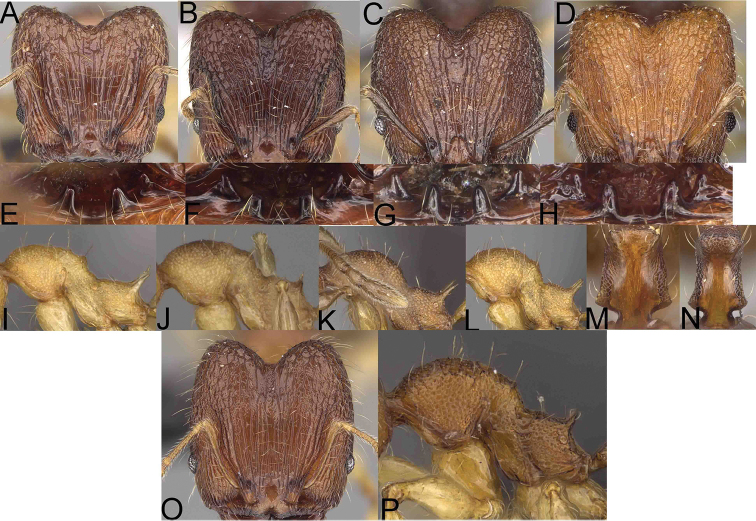
*Pheidole ferruginea* sp. nov., head of major worker (**A**), hypostomal teeth (**E**), profile of minor worker (**I**). *Pheidole rugocephala* sp. nov., head of major worker (**B**), hypostomal teeth (**F**), profile of minor worker (**J**). *Pheidole vohemarensis* sp. nov., head of major worker (**C**), hypostomal teeth (**G**), profile of minor worker (**K**), dorsal view of petiole of major worker (**M**). *Pheidole manantenensis* sp. nov., head of major worker (**D**), hypostomal teeth (**H**), profile of minor worker (**L**), dorsal view of petiole of major worker (**N**).

#### Revision of the *Pheidole ferruginea* complex

**Diagnosis. *Major workers*.** Head in full-face view cordate, widened posteriorly; antennal scrobes developed, well delimited by carinulae; occipital lobes with sparse and thick rugoreticulation or foveolae, with additional thick, sparse, irregular rugae; frons with sparse, thick, and longitudinal to irregular rugae, interspaces smooth, or with fine rugulae or superficially foveolate; promesonotum short, angular, and low to moderately high; propodeal spines long, with base wide or narrow; mesosoma with thick and sparse to dense rugoreticulation, sometimes sculpture weakening on dorsum, or foveolate, with additional sparse to dense rugoreticulation; first gastral tergite shagreened, at least on its basal part; body reddish brown to brown. ***Minor workers*.** Scape, when laid back, surpassing posterior head margin by one- to two-fifths of its length; promesonotum box-like or convex; propodeal spines moderately long to very long; promesonotal groove absent or present; metanotal groove absent or present; mesosoma foveolate, sometimes with additional rugae on mesosomal dorsum; sometimes katepisternum smooth; body yellow to brown.

**Comments.** Major workers of this complex can be distinguished based on a combination of the following characters: head in full-face view cordate, in lateral view oval to sub-oval; antennal scrobes developed and well delimited; never smooth occipital lobes and frons; long propodeal spines; and first gastral tergite shagreened, at least on its basal part. Minor workers can be distinguished based on foveolate head and mesosoma, sometimes with additional longitudinal rugae on frons or promesonotum; moderately long to long propodeal spines; and body yellow to brown.

##### Pheidole ferruginea sp. nov.

Taxon classificationAnimaliaHymenopteraFormicidae

http://zoobank.org/8B2D9618-D416-4469-96C4-713A4DB61FAC

**Figure 35.**
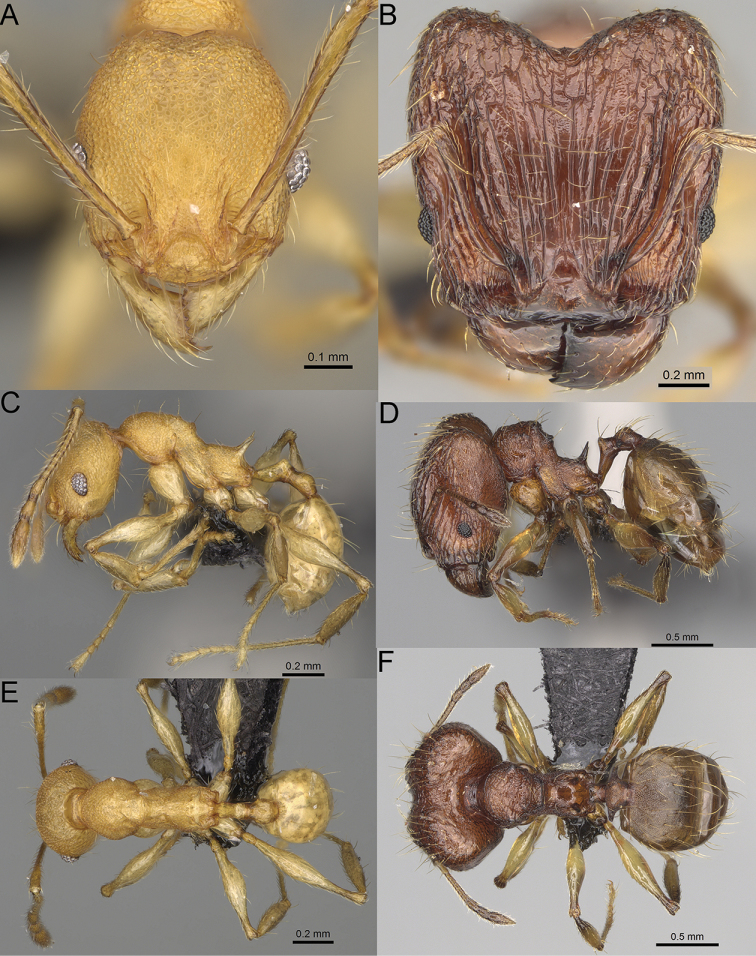
*Pheidole ferruginea* sp. nov., full-face view (**A**), profile (**C**), and dorsal view (**E**) of paratypeminor worker (CASENT0494994) and full-face view (**B**), profile (**D**), and dorsal view (**F**) of holotype major worker (CASENT0494997).

Figs 35A–F , 84P , 86P

###### Type material.

***Holotype*.** Madagascar. •1 major worker; Antsiranana; Parc National de Marojejy, Manantenina River, 27.6 km 35°NE Andapa, 9.6 km 327°NNW Manantenina; -14.435, 49.76; alt. 775 m; 16 Nov 2003; B.L. Fisher leg.; BLF08998, CASENT0494997, middle specimen on the pin (CASC). ***Paratypes*.** Madagascar. •9w., 5s.; same data as for holotype; CASENT0494993–CASENT0494996, CASENT0872087, CASENT0872230–CASENT0872238 (CASC).

###### Other material.

Madagascar. –***Antsiranana***: •15w., 6s., 1m., 1q.; Mandraka; -18.91813, 47.91717; alt. 1312 m; 20 Mar 2014; B.L. Fisher et al. leg.; CASENT0375496, CASENT0377119, CASENT0377122, CASENT0377125, CASENT0377128, CASENT0377134, CASENT0377137, CASENT0377144, CASENT0377148, CASENT0377153, CASENT0377156, CASENT0377159, CASENT0378177, CASENT0378178, CASENT0378179, CASENT0378299, CASENT0378301, CASENT0378305, CASENT0378566, CASENT0378569 (CASC). •16w., 5s.; Reg. Analamanga, St. Forestière Mandraka; -18.9183, 47.91687; alt. 1285 m; 25 Mar 2015; B.L. Fisher et al. leg.; CASENT0390402, CASENT0390414, CASENT0390440, CASENT0390443, CASENT0390444, CASENT0390507, CASENT0390537, CASENT0703931, CASENT0720608, CASENT0720646, CASENT0720649, CASENT0720831, CASENT0720836, CASENT0720837, CASENT0728120, CASENT0728121 (CASC). •6w., 3s.; 6.5 km SSW Befingotra, Rés. Anjanaharibe-Sud; -14.75, 49.5; alt. 875 m; 19 Oct 1994; B.L. Fisher leg.; CASENT0198459–CASENT0198461 (CASC). •2s.; 9.2 km WSW Befingotra, Rés. Anjanaharibe-Sud; -14.75, 49.46667; alt. 1200 m; 9 Nov 1994; B.L. Fisher leg.; CASENT0198916 (CASC). •3w., 1s.; Binara Forest; -13.26392, 49.59919; alt. 1065 m; 18 Oct 2013; B.L. Fisher et al. leg.; CASENT0369870, CASENT0369873, CASENT0369876, CASENT0369878 (CASC). •3w., 1s.; Forêt Ambanitaza, 26.1 km 347° Antalaha; -14.67933, 50.18367; alt. 240 m; 26 Nov 2004; B.L. Fisher leg.; CASENT0055590, CASENT0055598 (CASC). •3w., 1s.; Forêt de Binara, 9.1 km 233°SW Daraina; -13.26333, 49.60333; alt. 800 m; 3 Dec 2003; B.L. Fisher leg.; CASENT0043315, CASENT0043364, CASENT0043367, CASENT0043447 (CASC). •12w., 3s., 1m.; Makirovana Forest; -14.17066, 49.95409; alt. 415 m; 28 Apr 2011; B.L. Fisher et al. leg.; CASENT0231230, CASENT0231233, CASENT0231258, CASENT0231270, CASENT0231278, CASENT0236129, CASENT0236143, CASENT0243310, CASENT0243314, CASENT0243315, CASENT0243348 (CASC). •2w.; Masoala National Park; -15.32331, 50.30751; alt. 60 m; 10 Mar 2014; B.L. Fisher et al. leg.; CASENT0376974, CASENT0376995 (CASC). •1s.; Masoala, Cap Est, Forêt d’Andranoanala; -15.26158, 50.4758; alt. 15 m; 15 Mar 2014; B.L. Fisher et al. leg.; CASENT0377900 (CASC). •39w., 7s., 1q.; Parc National de Marojejy, Manantenina River, 27.6 km 35°NE Andapa, 9.6 km 327°NNW Manantenina; -14.435, 49.76; alt. 775 m; 15 Nov 2003; B.L. Fisher et al. leg.; CASENT0045227, CASENT0045231, CASENT0045238, CASENT0045240, CASENT0045243, CASENT0045248, CASENT0045250, CASENT0045252, CASENT0045254, CASENT0045260, CASENT0045264, CASENT0045267, CASENT0045272, CASENT0045334, CASENT0045351, CASENT0045363, CASENT0045366, CASENT0045744, CASENT0045904, CASENT0045987, CASENT0068463, CASENT0068464, CASENT0494859, CASENT0494860, CASENT0494917, CASENT0846566–CASENT0846569, CASENT0846571CASENT0846572, CASENT0846574, CASENT0846577–CASENT0846587 (CASC). •20w., 4s.; Parc National de Marojejy, Manantenina River, 28.0 km 38°NE Andapa, 8.2 km 333°NNW Manantenina; -14.43667, 49.775; alt. 450 m; 12 Nov 2003; B.L. Fisher et al. leg.; CASENT0045803, CASENT0045805, CASENT0045811, CASENT0045832, CASENT0045845, CASENT0045867, CASENT0045876, CASENT0045886, CASENT0045889, CASENT0045899, CASENT0045902, CASENT0045908, CASENT0046015, CASENT0046021, CASENT0047669, CASENT0077149, CASENT0077150, CASENT0077152 (CASC). –**Fianarantsoa**: •6w., 4s., 1q.; 2 km W Andrambovato, along river Tatamaly; -21.51167, 47.41; alt. 1075 m; 3 Jun 2005; B.L. Fisher et al. leg.; CASENT0060943, CASENT0060944, CASENT0061397, CASENT0061581, CASENT0061621, CASENT0061677, CASENT0109000 (CASC). •1w.; 43 km S Ambalavao, Rés. Andringitra; -22.23333, 47; alt. 825 m; 5 Oct 1993; B.L. Fisher leg.; CASENT0198537 (CASC). •1w.; 45 km S. Ambalavao; -22.21667, 47.01667; alt. 785 m; 25 Sep 1993; B.L. Fisher leg.; CASENT0198538 (CASC). •1w.; 7.6 km 122° Kianjavato, Forêt Classée Vatovavy; -21.4, 47.94; alt. 175 m; 6 Jun 2005; B.L. Fisher et al. leg.; CASENT0061235 (CASC). •3w., 1s.; 9.0 km NE Ivohibe; -22.42667, 46.93833; alt. 900 m; 12 Nov 1997; B.L. Fisher et al. leg.; CASENT0198539, CASENT0198541, CASENT0198918 (CASC). •5w., 2s., 1q.; Ambinanindranomena Non-Protected Area, 39.45 km SE Ambalavao; -21.95386, 47.29427; alt. 1069 m; 1 Feb 2012; Andrianjaka & Ravelomanana leg.; CASENT0293842, CASENT0293876, CASENT0293878, CASENT0293916, CASENT0293917, CASENT0293935 (CASC). •1w., 1s.; Forêt d’Ambalagoavy Nord, Ikongo, Ambatombe; -21.857068, 47.37849; alt. 625 m; 1 Dec 2000; Harin’Hala & Irwin leg.; CASENT0009589, CASENT0009636 (CASC). •2w.; P.N. Ranomafana, Sahavondrona-Ampitamarivo; -21.2575, 47.36015; alt. 1100 m; 18 Mar 2003; Clark leg.; CASENT0052899, CASENT0052904 (CASC). •1w.; P.N. Ranomafana, Tolongoina-Ampasimpotsy 3; -21.47412, 47.55742; alt. 520 m; 11 Apr 2003; Clark leg.; CASENT0052880 (CASC). •1w., 1s., 1q.; Parc National Befotaka-Midongy, Papango 27.7 km S Midongy-Sud, Mount Papango; -23.83517, 46.96367; alt. 940 m; 13 Nov 2006; B.L. Fisher et al. leg.; CASENT0128696, CASENT0128700, CASENT0128704 (CASC). •1w., 1s.; Parc National Befotaka-Midongy, Papango 28.5 km S Midongy-Sud, Mount Papango; -23.84083, 46.9575; alt. 1250 m; 17 Nov 2006; B.L. Fisher et al. leg.; CASENT0118391 (CASC). •1w., 1s.; Parc National de Ranomafana, Vatoharanana River, 4.1 km 231°SW Ranomafana; -21.29, 47.43333; alt. 1100 m; 27 Mar 2003; B.L. Fisher et al. leg.; CASENT0039949, CASENT0040207 (CASC). •2w., 1s.; R.S. Ivohibe 8.0 km E Ivohibe; -22.48333, 46.96833; alt. 1200 m; 15 Oct 1997; B.L. Fisher et al. leg.; CASENT0198540, CASENT0198542, CASENT0198919 (CASC). •1w.; Vohiparara Kidonavo 1; -21.22632, 47.37007; alt. 1100 m; 13 Mar 2003; Clark leg.; CASENT0052978 (CASC). –**Toamasina**: •3w.; Ambanizana, Parc National Masoala; -15.57167, 50.00611; alt. 900–950 m; 26 Feb 2003; Andriamalala et al. leg.; CASENT0008765, CASENT0073478, CASENT0073478 (CASC). •3w., 1s.; Ambatovy, 12.4 km NE Moramanga; -18.83937, 48.30842; alt. 1080 m; 4 Mar 2007; B.L. Fisher et al. leg.; CASENT0123826, CASENT0123867, CASENT0123870, CASENT0123997 (CASC). •1w.; Analamay; -18.80623, 48.33707; alt. 1068 m; 21 Mar 2004; B.L. Fisher et al. leg.; CASENT0046556 (CASC). •2w.; Ankerana; -18.4017, 48.80605; alt. 1035 m; 24 Jan 2012; B.L. Fisher et al. leg.; CASENT0274446, CASENT0274448 (CASC). •6w.; Ankerana; -18.4061, 48.82029; alt. 725 m; 16 Jan 2012; B.L. Fisher et al. leg.; CASENT0274767, CASENT0275345, CASENT0275347, CASENT0275348, CASENT0275462 (CASC). •2w., 2q.; Ankerana; -18.40829, 48.82107; alt. 750 m; 21 Jan 2012; B.L. Fisher et al. leg.; CASENT0274977, CASENT0274978 (CASC). •1w.; Ankerana; -18.4104, 48.8189; alt. 855 m; 22 Jan 2012; B.L. Fisher et al. leg.; CASENT0274417 (CASC). •1w.; Corridor Forestier Analamay-Mantadia, Tsaravoniana; -18.75641, 48.42195; alt. 1036 m; 2 Dec 2012; B.L. Fisher et al. leg.; CASENT0301883 (CASC). •3w., 2s.; F.C. Sandranantitra; -18.04833, 49.09167; alt. 450 m; 18 Jan 1999; Ratsirarson leg.; CASENT0198543, CASENT0198920 (CASC). •2w., 1s.; Ile Sainte Marie, Forêt Kalalao, 9.9 km 34° Ambodifotatra; -16.9225, 49.88733; alt. 100 m; 24 Nov 2005; B.L. Fisher et al. leg.; CASENT0069837, CASENT0069839, CASENT0069844 (CASC). •1w., 1s.; Mahavelona (Foulpointe); -17.66667, 49.5; 1 Nov 1985; A. Pauly leg.; CASENT0095644, CASENT0095650 (CASC). •11w., 11s.; Montagne d’Akirindro 7.6 km 341°NNW Ambinanitelo; -15.28833, 49.54833; alt. 600 m; 17 Mar 2003; B.L. Fisher et al. leg.; CASENT0038892, CASENT0038907, CASENT0039031, CASENT0039045, CASENT0039049, CASENT0039079, CASENT0039080, CASENT0039090, CASENT0039094, CASENT0039109, CASENT0039113, CASENT0039121, CASENT0039194, CASENT0039205, CASENT0039213, CASENT0039217, CASENT0039224, CASENT0039226, CASENT0039233, CASENT0496453 (CASC). •2w.; Montagne d’Anjanaharibe, 18.0 km 21°NNE Ambinanitelo; -15.18833, 49.615; alt. 470 m; 8 Mar 2003; B.L. Fisher et al. leg.; CASENT0037612, CASENT0037639 (CASC). •1w., 3s.; Parc National de Zahamena, Onibe River; -17.75908, 48.85468; alt. 780 m; 21 Feb 2009; B.L. Fisher et al. leg.; CASENT0150453, CASENT0151642, CASENT0153420, CASENT0153422 (CASC). •4w.; Parc National de Zahamena, Tetezambatana forest, near junction of Nosivola and Manakambahiny Rivers; -17.74298, 48.72936; alt. 860 m; 18 Feb 2009; B.L. Fisher et al. leg.; CASENT0151078, CASENT0153357, CASENT0153371, CASENT0153374 (CASC). •4w., 4s.; Parc National Mananara-Nord, 7.1 km 261° Antanambe; -16.455, 49.7875; alt. 225 m; 16 Nov 2005; B.L. Fisher et al. leg.; CASENT0067223, CASENT0068923, CASENT0069441, CASENT0069445CASENT0069451, CASENT0069605, CASENT0071324 (CASC). •1w., 2s.; Reserve Betampona, Camp Rendrirendry 34.1 km 332° Toamasina; -17.924, 49.19967; alt. 390 m; 28 Nov 2005; B.L. Fisher et al. leg.; CASENT0071885, CASENT0071912, CASENT0069185 (CASC). •1w.; Réserve Spéciale Ambatovaky, Sandrangato River; -16.81739, 49.29402; alt. 360 m; 25 Feb 2010; B.L. Fisher et al. leg.; CASENT0163669 (CASC). •8w., 1q.; Réserve Spéciale Ambatovaky, Sandrangato River; -16.77274, 49.26551; alt. 450 m; 20 Feb 2010; B.L. Fisher et al. leg.; CASENT0164042, CASENT0164417, CASENT0164422, CASENT0164440, CASENT0164468, CASENT0164483, CASENT0164485, CASENT0164492, CASENT0164498 (CASC). •5w., 1q.; Réserve Spéciale Ambatovaky, Sandrangato River; -16.7633, 49.26692; alt. 520 m; 22 Feb 2010; B.L. Fisher et al. leg.; CASENT0163718, CASENT0163722, CASENT0163723, CASENT0163833, CASENT0163873, CASENT0163877 (CASC). –***Toliara***: •1w., 3s.; 10 km NW Enakara, Rés Andohahela; -24.56667, 46.81667; alt. 420 m; 15 Nov 1992; B.L. Fisher leg.; CASENT0198037, CASENT0196935 (CASC). •6w., 1s., 1q.; 11 km NW Enakara, Rés. Andohahela; -24.56667, 46.83333; alt. 800 m; 17 Nov 1992; B.L. Fisher leg.; CASENT0196936, CASENT0198050, CASENT0217984, CASENT0217992, CASENT0217993 (CASC). •5w.; 2.7 km WNW 302° Ste. Luce; -24.77167, 47.17167; alt. 20 m; 9 Dec 1998; B.L. Fisher et al. leg.; CASENT0196937–CASENT0196939, CASENT0198049, CASENT0198051 (CASC). •8w., 2s.; Anosy Region, Anosyenne Mts, 31.2 km NW Manantenina; -24.13894, 47.06804; alt. 1125 m; 26 Feb 2015; B.L. Fisher et al. leg.; CASENT0704213, CASENT0704214, CASENT0704872, CASENT0704873, CASENT0704876, CASENT0704877, CASENT0721011 (CASC). •2w., 1s.; Parc National Andohahela, Col de Tanatana, 33.3 km NW Tolagnaro; -24.7585, 46.85367; alt. 275 m; 24 Nov 2006; B.L. Fisher et al. leg.; CASENT0122564, CASENT0129922, CASENT0129927 (CASC). •1w., 1s.; Parc National Andohahela, Manangotry, 33.8 km NW Tolagnaro; -24.75117, 46.85783; alt. 575 m; 24 Nov 2006; B.L. Fisher et al. leg.; CASENT0121878 (CASC). •21w., 6s.; Parc National d’Andohahela, Col du Sedro, 3.8 km 113°ESE Mahamavo, 37.6 km 341°NNW Tolagnaro; -24.76389, 46.75167; alt. 900 m; 21 Jan 2002; B.L. Fisher et al. leg.; CASENT0078392, CASENT0078409, CASENT0078410, CASENT0078412, CASENT0479172, CASENT0479174, CASENT0479187, CASENT0479188, CASENT0479239, CASENT0479260, CASENT0479261, CASENT0479262, CASENT0479299, CASENT0483039, CASENT0483924, CASENT0483951, CASENT0483967, CASENT0483968, CASENT0483980, CASENT0484029, CASENT0484043, CASENT0484060, CASENT0484062, CASENT0484063, CASENT0484111, CASENT0484183 (CASC). •6w., 2s., 1q.; Réserve Spéciale Kalambatritra, Ampanihy; -23.4635, 46.4631; alt. 1270 m; 9 Feb 2009; B.L. Fisher et al. leg.; CASENT0150522, CASENT0150523, CASENT0153214, CASENT0153217, CASENT0217989, CASENT0846595, CASENT0846596, CASENT0846597 (CASC).

###### Diagnosis.

Head in full-face view cordate, widened posteriorly; sides of the head with sparse, long, suberect pilosity; antennal scrobes strongly developed, well delimited and forming distinct dorsal concavity beneath frontal carina; scrobe surface shiny, with sparse, thick, longitudinal and sometimes additional irregular rugae; delimited ventrally and posteriorly by carinulae; propodeal spines long (PSL: 0.23–0.28 (0.26)); first gastral tergite shagreened, at least on its basal part. ***Minor workers*.** Head foveolate, sometimes with additional longitudinal rugae on frons; propodeal spines very long (PSL: 0.15–0.18 (0.16)); promesonotum box-like; mesosoma foveolate, sometimes with additional rugae on mesosomal dorsum, and katepisternum with smooth notch.

###### Description.

**Major workers.** Measurements (*N* = 10): HL: 1.18–1.34 (1.25); HW: 1.15–1.31 (1.23); SL: 0.61–0.69 (0.64); EL: 0.13–0.17 (0.15); WL: 0.93–1.05 (0.99); PSL: 0.23–0.28 (0.26); MTL: 0.58–0.66 (0.61); PNW: 0.5–0.6 (0.53); PTW: 0.13–0.17 (0.16); PPW: 0.45–0.58 (0.51); CI: 97.4–101.3 (99.0); SI: 48.7–55.8 (52.1); PSLI: 18.9–21.9 (20.8); PPI: 27.5–33.7 (30.7); PNI: 40.9–45.0 (43.0); MTI: 47.6–52.2 (49.7). ***Head*.** In full-face view cordate, widened posteriorly (Fig. 35B). In lateral view oval; ventral and dorsal faces strongly convex; inner hypostomal teeth visible. Sides of the head with sparse, long, suberect pilosity; whole head with moderately dense, long, suberect to erect pilosity. Antennal scrobes strongly developed, well delimited and forming distinct dorsal concavity beneath frontal carina, scrobe surface shiny, with sparse, thick, longitudinal, and sometimes additional irregular rugae, delimited ventrally and posteriorly by carinulae. Occipital lobes shiny, with sparse and thick rugoreticulation; genae shiny, with moderately dense to dense, fine rugoreticulation; frons and malar area with sparse, thick, and longitudinal rugae, interspaces smooth or with fine rugulae. Centre of clypeus smooth and shiny, lateral sides with longitudinal rugae; median notch present, narrow, and shallow; median longitudinal carina present but sometimes indistinct; lateral longitudinal carinae absent. Scape, when laid back, reaching slightly beyond the midlength of head; pilosity decumbent to erect (Fig. 35B, D). Inner hypostomal teeth distinct, closely spaced, triangular, with rounded apex and moderately narrow base; outer hypostomal teeth distinct, slightly lower than inner hypostomal teeth, lobe-like, with base wide and tops directed slightly inward (Fig. 84P). ***Mesosoma*.** In lateral view, promesonotum short, angular, and moderately high, posterior mesonotum with tubercle-like projection, dropping steeply to propodeum; promesonotal groove absent; metanotal groove absent; propodeal spines long, massive basally, with acute apex; humeral area laterally weakly produced (Fig. 35D). Surface shiny, with thick and dense rugoreticulation, sometimes sculpture weakening on dorsum. Pilosity moderately sparse, long, and erect (Fig. 35D, F). ***Petiole*.** Shagreened; peduncle relatively long, without horizontal lobes on its basal part; node low, triangular, with rounded apex, in rear view node slightly convex; pilosity moderately sparse and erect (Fig. 35D, F). ***Postpetiole*.** Shagreened; in dorsal view sides with acute, triangular, moderately long projections; pilosity long, moderately sparse, and erect (Fig. 35D, F). ***Petiole*.** First gastral tergite shagreened, at least on its basal part; pilosity moderately sparse, long, and erect (Fig. 35D, F). ***Colour*.** Head and mesosoma reddish brown to brown; dorsum of mesosoma dark brown; gaster brown; legs yellow to brown (Fig. 35D, F).

**Minor workers.** Measurements (*N* = 10): HL: 0.52–0.57 (0.55); HW: 0.44–0.48 (0.46); SL: 0.57–0.63 (0.6); EL: 0.11–0.13 (0.12); WL: 0.67–0.72 (0.7); PSL: 0.15–0.18 (0.16); MTL: 0.44–0.49 (0.46); PNW: 0.31–0.35 (0.33); PTW: 0.07–0.09 (0.08); PPW: 0.13–0.14 (0.13); CI: 80.9–86.2 (83.9); SI: 124.6–134.8 (130.0); PSLI: 27.5–31.4 (29.3); PPI: 57.4–67.9 (63.1); PNI: 69.6–74.8 (71.7); MTI: 96.9–102.3 (100.9). ***Head*.** Occipital margin straight or indistinctly concave; occipital carina absent (Fig. 35A). Pilosity sparse, long, suberect to erect. Whole head foveolate, sometimes with additional longitudinal rugae on frons. Clypeus foveolate; median longitudinal carina absent; two lateral longitudinal carinae absent. Scape, when laid back, surpassing posterior head margin by two-fifths of its length; pilosity suberect to erect (Fig. 35A, C). ***Mesosoma*.** In lateral view, promesonotum box-like; promesonotal groove absent; metanotal groove absent; propodeal spines very long, massive basally, with acute apex (Fig. 35C). Whole surface foveolate, sometimes with additional rugae on mesosomal dorsum; sometimes katepisternum with smooth notch. Pilosity sparse, long, and erect (Fig. 35C, E). ***Petiole*.** Peduncle relatively long and thin; node low, globular; with few long, erect setae (Fig. 35C, E). ***Postpetiole*.** Short, low, and convex; with few long, erect setae at the anterior edge (Fig. 35C, E). ***Petiole*.** Pilosity sparse and erect (Fig. 35C, E). ***Colour*.** Unicolourous, yellow to brown (Fig. 35C, E).

###### Etymology.

Latin for rusty, in reference to body colouration of major workers.

###### Biology.

The species was collected between 20–1312 m in elevation, in rainforest, montane rainforest, and littoral rainforest. Nests were located in rotten logs and tree stumps, rotten sticks on ground, and the petioles of Melastomataceae.

###### Comments.

This species is most similar to *P. rugocephala* sp. nov. ***Major workers*.***Pheidole ferruginea* sp. nov. can be distinguished from *P. rugocephala* sp. nov. by the surface of antennal scrobes never foveolate; presence of longitudinal rugae on frons; inner hypostomal teeth newer pointed inward; never smooth surface of promesonotum; and lack of horizontal lobes on the basal part of petiolar peduncle. ***Minor workers*.***Pheidole ferruginea* sp. nov. can be distinguished from *P. rugocephala* sp. nov. by long propodeal spines and foveolate clypeus, and lack of promesonotal and metanotal grooves.

##### Pheidole rugocephala sp. nov.

Taxon classificationAnimaliaHymenopteraFormicidae

http://zoobank.org/5BBD39BF-7F33-4566-BB02-8B77832D3971

**Figure 36.**
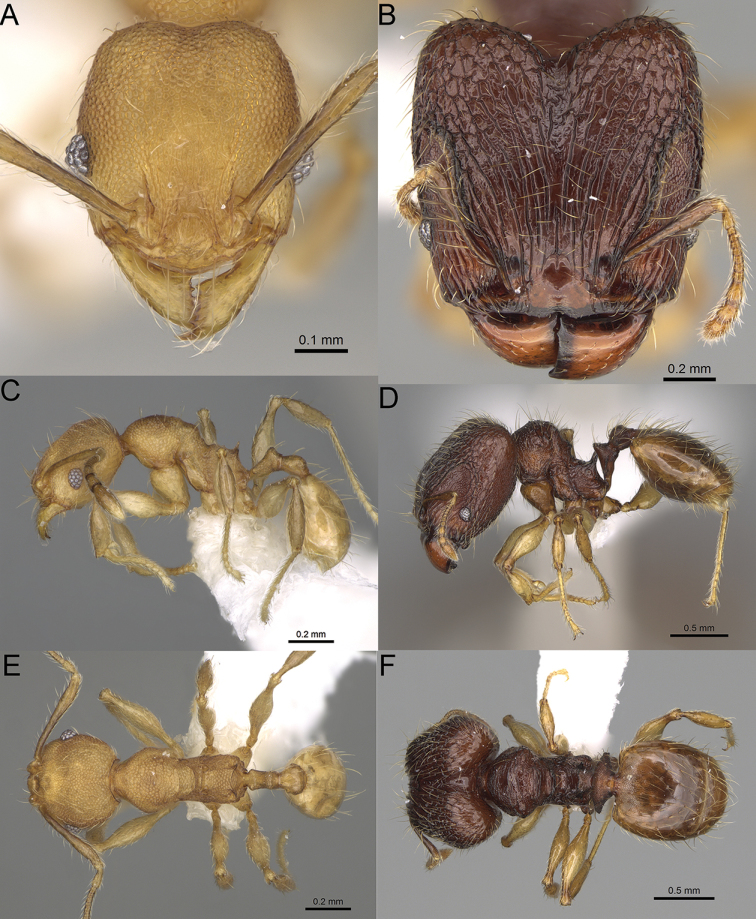
*Pheidole rugocephala* sp. nov., full-face view (**A**), profile (**C**), and dorsal view (**E**) of paratype minor worker (CASENT0198470) and full-face view (**B**), profile (**D**), and dorsal view (**F**) of holotype major worker (CASENT0923224).

Figs 36A–F , 85U , 88E

###### Type material.

***Holotype*.** Madagascar. •1 major worker; Toamasina; 6.9 km NE Ambanizana, Ambohitsitondroina; -15.58506, 50.00952; alt. 825 m; 2 Dec 1993; B.L. Fisher leg.; BLF00976, CASENT0923224 (CASC). ***Paratypes*.** Madagascar. •1w., 1q.; same data as for holotype; CASENT0198470, CASENT0872239 (CASC).

###### Other material.

Madagascar. –***Antsiranana***: •1q.; 9.2 km WSW Befingotra, Rés. Anjanaharibe-Sud; -14.75, 49.46667; alt. 1200 m; 9 Nov 1994; B.L. Fisher leg. CASENT0196913 (CASC). •1s.; Masoala National Park; -15.3014, 50.22776; alt. 280 m; 7 Mar 2014; B.L. Fisher et al. leg.; CASENT0377718 (CASC). •1s.; Masoala National Park; -15.32331, 50.30751; alt. 60 m; 10 Mar 2014; B.L. Fisher et al. leg.; CASENT0376820 (CASC). –***Toamasina***: •1w., 1s.; Montagne d’Anjanaharibe, 18.0 km 21°NNE Ambinanitelo; -15.18833, 49.615; alt. 470 m; 8 Mar 2003; B.L. Fisher et al. leg.; CASENT0037624, CASENT0037758 (CASC). •1s.; Montagne d’Anjanaharibe, 19.5 km 27°NNE Ambinanitelo; -15.17833, 49.635; alt. 1100 m; 12 Mar 2003; B.L. Fisher et al. leg.; CASENT0038365 (CASC). •4s.; Parc National Mananara-Nord, 7.1 km 261° Antanambe; -16.455, 49.7875; alt. 225 m; 14 Nov 2005; B.L. Fisher et al. leg.; CASENT0068924, CASENT0069459, CASENT0069581, CASENT0069598 (CASC). •1s.; Reserve Betampona, Camp Vohitsivalana, 37.1 km 338° Toamasina; -17.88667, 49.2025; alt. 520 m; 1 Dec 2005; B.L. Fisher et al. leg.; CASENT0069316 (CASC).

###### Diagnosis.

Head in full-face view cordate, widened posteriorly; sides of the head with moderately dense, long, suberect to erect pilosity; antennal scrobes present, strongly developed, well delimited and forming distinct dorsal concavity beneath frontal carina; scrobe surface foveolate; delimited ventrally and posteriorly by carinulae; propodeal spines long (PSL: 0.13–0.2 (0.15)); first gastral tergite shagreened on its basal part. ***Minor workers*.** Head foveolate; propodeal spines long (PSL: 0.09); promesonotum box-like; mesosoma foveolate; katepisternum with smooth notch.

###### Description.

**Major workers.** Measurements (*N* = 10): HL: 0.86–1.18 (0.97); HW: 0.87–1.12 (0.93); SL: 0.42–0.52 (0.45); EL: 0.12–0.14 (0.13); WL: 0.72–0.99 (0.82); PSL: 0.13–0.2 (0.15); MTL: 0.43–0.51 (0.45); PNW: 0.47–0.62 (0.53); PTW: 0.13–0.19 (0.14); PPW: 0.35–0.53 (0.41); CI: 95.3–101.3 (97.5); SI: 46.0–50.9 (48.2); PSLI: 14.3–17.0 (15.7); PPI: 30.9–36.9 (33.6); PNI: 53.7–59.9 (56.4); MTI: 45.5–52.3 (48.5). ***Head*.** In full-face view cordate, widened posteriorly (Fig. 36B). In lateral view oval; ventral face convex; dorsal face relatively flat; inner hypostomal teeth visible. Sides of the head with moderately dense, long, suberect to erect pilosity; whole head with moderately dense, long, suberect to erect pilosity. Antennal scrobes strongly developed, well delimited and forming distinct dorsal concavity beneath frontal carina, scrobe surface foveolate, delimited ventrally and posteriorly by carinulae. Occipital lobes shiny, with sparse and thick rugae, sculpture weakening posteriorly; frons and malar area with thick, sparse, longitudinal or irregular rugae, interspaces smooth; genae with dense and fine rugulae. Centre of clypeus smooth and shiny, lateral sides with longitudinal rugae; median notch present, narrow, and shallow; median longitudinal carina absent; lateral longitudinal carinae present. Scape, when laid back, reaching slightly beyond the midlength of head; pilosity decumbent to erect (Fig. 36B, D). Inner hypostomal teeth distinct, closely spaced, triangular, with rounded apex pointed inward; outer hypostomal teeth distinct, slightly higher than inner hypostomal teeth, lobe-like, with base wide and acute tops directed outward; inner and outer teeth closely spaced and connected by concavity (Fig. 85U). ***Mesosoma*.** In lateral view, promesonotum short, angular, and moderately high, posterior mesonotum with tubercle-like projection, dropping steeply to propodeum; promesonotal groove absent; metanotal groove absent; propodeal spines long, with narrow base and acute apex; humeral area laterally weakly produced (Fig. 36D). Surface shiny, with sparse rugoreticulation, dorsum with weaker sculpture and sometimes smooth patches. Pilosity moderately sparse, long, and erect (Fig. 36D, F). ***Petiole*.** Finely foveolate; peduncle relatively long, with horizontal, triangular lobes on its basal part; node high, triangular, with rounded apex, in rear view node dorsoventrally depressed; pilosity moderately sparse and erect (Fig. 36D, F). ***Postpetiole*.** Finely foveolate; short; in dorsal view sides with acute, very wide, and long triangular projections; pilosity long, moderately dense and erect (Fig. 36D, F). ***Petiole*.** First gastral tergite shagreened on its basal part; pilosity moderately dense, very long and erect (Fig. 36D, F). ***Colour*.** Head and mesosoma reddish brown to brown; gaster brown; legs yellow to brown (Fig. 36D, F).

**Minor workers.** Measurements (*N* = 1): HL: 0.48; HW: 0.43; SL: 0.42; EL: 0.09; WL: 0.6; PSL: 0.09; MTL: 0.35; PNW: 0.3; PTW: 0.08; PPW: 0.14; CI: 89.4; SI: 99.1; PSLI: 18.6; PPI: 58.6; PNI: 69.2; MTI: 82.2. ***Head*.** Occipital margin straight or indistinctly concave; occipital carina absent (Fig. 36A). Pilosity sparse, long, suberect to erect. Whole head foveolate. Clypeus with median longitudinal carina absent; two lateral longitudinal carinae absent. Scape, when laid back, surpassing posterior head margin by one-fifth of its length; pilosity suberect to erect (Fig. 36A, C). ***Mesosoma*.** In lateral view, promesonotum box-like; promesonotal groove present; metanotal groove present; propodeal spines moderately long, triangular, with acute apex (Fig. 36C). Whole surface foveolate, only katepisternum with smooth notch. Pilosity sparse, long and erect (Fig. 36C, E). ***Petiole*.** Shiny; peduncle foveolate, short and thin; node finely foveolate, globular; with few long, erect setae (Fig. 36C, E). ***Postpetiole*.** Finely foveolate with smooth centre; short, low and convex; with few long, erect setae at the anterior edge (Fig. 36C, E). ***Petiole*.** With few long, erect setae (Fig. 36C, E). ***Colour*.** Unicolourous, yellow (Fig. 36C, E).

###### Etymology.

Latin for strong and distinct head sculpture on major workers.

###### Biology.

The species was collected between 15–1200 m in elevation, in rainforest, montane rainforest, and littoral rainforest. Nesting preferences unknown.

###### Comments.

This species is most similar to *P. ferruginea* sp. nov. ***Major workers*.***Pheidole rugocephala* sp. nov. can be distinguished from *P. ferruginea* sp. nov. by foveolate surface of antennal scrobes; lack of longitudinal rugae on frons, inner hypostomal teeth pointed inward, presence of smooth surface on promesonotum, and presence of horizontal lobes on the basal part of petiolar peduncle. ***Minor workers*.***Pheidole rugocephala* sp. nov. can be distinguished from *P. ferruginea* sp. nov. by short and triangular propodeal spines, never foveolate clypeus, and presence of promesonotal and metanotal grooves.

##### Pheidole manantenensis sp. nov.

Taxon classificationAnimaliaHymenopteraFormicidae

http://zoobank.org/2A00A2C0-FA00-48C1-AEFB-847A94ABE490

**Figure 37.**
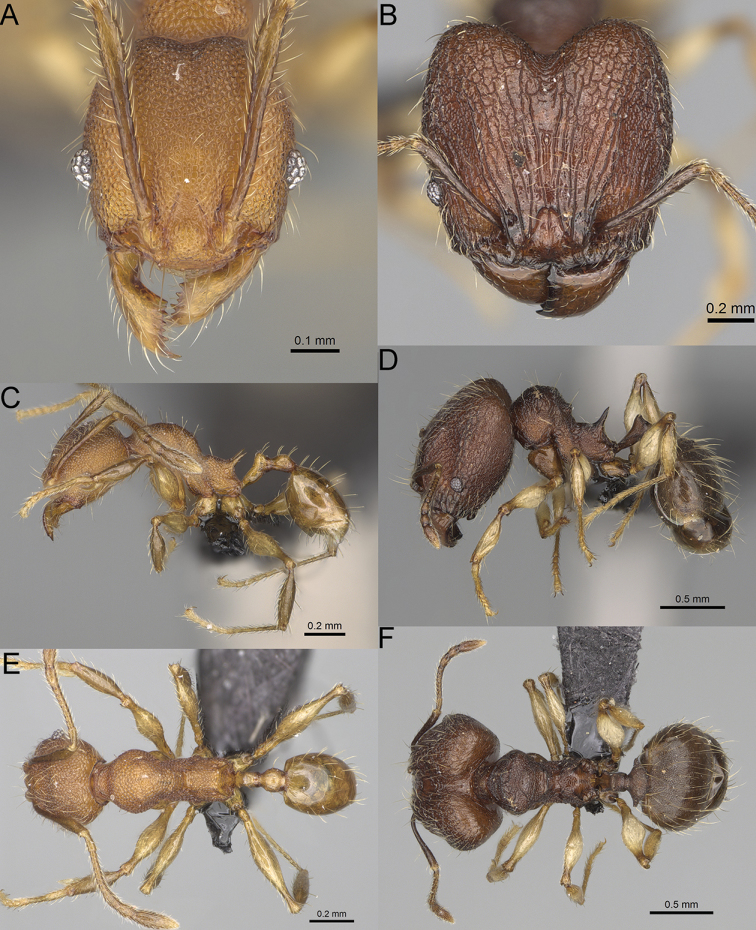
*Pheidole manantenensis* sp. nov., full-face view (**A**), profile (**C**), and dorsal view (**E**) of paratype minor worker (CASENT0923218) and full-face view (**B**), profile (**D**), and dorsal view (**F**) of holotype major worker (CASENT0068456).

Figs 37A–F , 85F , 87J

###### Type material.

***Holotype*.** Madagascar. •1 major worker; Antsiranana; Parc National de Marojejy, Manantenina River, 27.6 km 35°NE Andapa, 9.6 km 327°NNW Manantenina; -14.435, 49.76; alt. 775 m; 12 Dec 2005; Fisher et al. leg.; BLF13478, CASENT0068456 (CASC). ***Paratypes*.** Madagascar. •1w., 1q.; same data as for holotype; CASENT0068457, CASENT0923218 (CASC).

###### Other material.

Madagascar. –***Antsiranana***: •2w.; Makirovana Forest; -14.104, 50.03574; alt. 225 m; 4 May 2011; B.L. Fisher et al. leg.; CASENT0230806, CASENT0230809 (CASC). •1w.; Makirovana Forest; -14.16044, 49.95216; alt. 550 m; 1 May 2011; B.L. Fisher et al. leg.; CASENT0231430 (CASC). •4w.; Makirovana Forest; -14.16666, 49.95; alt. 715 m; 1 May 2011; B.L. Fisher et al. leg.; CASENT0243103, CASENT0243118, CASENT0243120, CASENT0243129 (CASC). –***Toamasina***: •1w., 1s.; Montagne d’Akirindro 7.6 km 341°NNW Ambinanitelo; -15.28833, 49.54833; alt. 600 m; 17 Mar 2003; B.L. Fisher et al. leg.; CASENT0038921, CASENT0039038 (CASC).

###### Diagnosis.

Head in full-face view cordate, widened posteriorly; sides of the head with moderately dense, short, suberect to erect pilosity; antennal scrobes present, weakly impressed, and indistinctly delimited ventrally and posteriorly by carinulae; scrobe surface foveolate, with indistinct, thick, moderately sparse, irregular rugae; propodeal spines long (PSL: 0.2–0.26 (0.24)); gaster shagreened; inner hypostomal teeth distinct, high, narrow, and triangular, closely spaced, with rounded apex; outer hypostomal teeth approximately as high as inner hypostomal teeth, but thinner and with wider base, lobe-like. ***Minor workers*.** Head and mesosoma foveolate; propodeal spines long (PSL: 0.14–0.17 (0.15)); promesonotum low, convex, short, with posterior declivity smoothly declining towards propodeum.

###### Description.

**Major workers.** Measurements (*N* = 10): HL: 0.96–1.13 (1.07); HW: 0.95–1.15 (1.07); SL: 0.52–0.58 (0.55); EL: 0.1–0.12 (0.12); WL: 0.79–0.94 (0.88); PSL: 0.2–0.26 (0.24); MTL: 0.49–0.57 (0.52); PNW: 0.45–0.54 (0.51); PTW: 0.12–0.15 (0.14); PPW: 0.33–0.44 (0.4); CI: 98.2–102.5 (100.5); SI: 48.3–54.7 (51.1); PSLI: 20.0–24.5 (22.1); PPI: 31.9–38.2 (34.8); PNI: 45.6–48.9 (47.1); MTI: 45.6–51.3 (48.2). ***Head*.** In full-face view cordate, slightly longer than wide, anterior of eyes slightly convex, posterior of eyes convex, occipital margins of lobes convex (Fig. 37B). In lateral view sub-oval; ventral and dorsal faces convex; dorsal face not depressed posteriorly; inner hypostomal teeth visible. Sides of the head with moderately dense, short, suberect to erect pilosity; whole head with moderately dense, long, suberect to erect pilosity. Antennal scrobes impressed and delimited ventrally and posteriorly by carinulae; scrobe surface foveolate, with indistinct, thick, moderately sparse, irregular rugae. Occipital lobes shiny, foveolate, with additional thick, sparse, irregular rugae, rugae weakening posteriorly; frons with moderately sparse, thick, longitudinal rugae, on the posterior part rugae longitudinal to irregular, interspaces superficially foveolate; genae foveolate, with additional, indistinct, and irregular rugulae; malar area with thick, sparse, longitudinal rugae, interspaces smooth to indistinctly rugulose. Centre of clypeus shiny and with indistinct, short rugulae, lateral sides with longitudinal rugae; median notch present, moderately wide, and shallow; median longitudinal carina present; lateral longitudinal carinae present. Scape, when laid back, slightly exceeding the midlength of head; pilosity suberect to erect (Fig. 37B, D). Inner hypostomal teeth distinct, high, narrow, and triangular, closely spaced, with rounded apex; outer hypostomal teeth approximately as high as inner hypostomal teeth, but thinner and with wider base, lobe-like (Fig. 85F). ***Mesosoma*.** In lateral view, promesonotum low and arched, dorsal mesonotum slightly concave, posterior mesonotum relatively steep, with small tubercle-like projections; promesonotal groove absent; metanotal groove absent; propodeal spines long, triangular, thin, with sharp apex and relatively narrow base; humeral area laterally weakly produced (Fig. 37D). Surface foveolate, with additional sparse and indistinct rugoreticulation; dorsoventral propodeum with fading sculpture. Pilosity sparse, long, erect (Fig. 37D, F). ***Petiole*.** Shiny; peduncle moderately long, foveolate, with distinct, short horizontal lobes on its basal part; node relatively high, triangular, with rounded apex, in rear view node slightly convex; pilosity moderately sparse and erect (Fig. 37D, F). ***Postpetiole*.** Shiny and foveolate; in dorsal view sides with moderately long, wide, acute, and triangular projections; pilosity moderately long, and erect (Fig. 37D, F). ***Petiole*.** Shiny and shagreened; pilosity moderately sparse, long, and erect (Fig. 37D, F). ***Colour*.** Brown to dark brown; legs yellow; malar area brighter that the rest of the head (Fig. 37D, F).

**Minor workers.** Measurements (*N* = 10): HL: 0.47–0.51 (0.49); HW: 0.41–0.46 (0.44); SL: 0.45–0.54 (0.49); EL: 0.08–0.11 (0.09); WL: 0.58–0.64 (0.62); PSL: 0.14–0.17 (0.15); MTL: 0.35–0.38 (0.37); PNW: 0.28–0.32 (0.31); PTW: 0.06–0.08 (0.07); PPW: 0.1–0.13 (0.11); CI: 85.2–93.0 (88.9); SI: 105.1–119.7 (111.8); PSLI: 29.2–34.3 (31.5); PPI: 60.0–73.2 (65.8); PNI: 67.5–74.1 (70.7); MTI: 79.8–88.6 (84.0). ***Head*.** Occipital margin straight or indistinctly concave; occipital carina indistinct (Fig. 37A). Pilosity moderately dense, long, suberect. Head foveolate; genae with fading sculpture. Clypeus foveolate; median longitudinal carina absent; two lateral longitudinal carinae absent. Scape, when laid back, surpassing the posterior head margin by two-fifths of its length; pilosity erect (Fig. 37A, C). ***Mesosoma*.** In lateral view, promesonotum low, convex, short, with posterior declivity smoothly declining towards propodeum; promesonotal groove absent; metanotal groove present; propodeal spines long, triangular, with acute apex and narrow base (Fig. 37C). Sculpture foveolate. Pilosity moderately sparse, short, and erect (Fig. 37C, E). ***Petiole*.** Peduncle moderately short and thin; with few short, erect setae (Fig. 37C, E). ***Postpetiole*.** Short, low, and convex; with few short, erect setae (Fig. 37C, E). ***Petiole*.** With sparse, erect pilosity (Fig. 37C, E). ***Colour*.** Dark yellow (Fig. 37C, E).

###### Etymology.

From the type locality.

###### Biology.

The species was collected between 225–775 m in elevation, in rainforest. Nest was located in the petiole of Melastomataceae.

###### Comments.

*Pheidole manantenensis* sp. nov. is most similar to *P. vohemarensis* sp. nov. **Majors workers.** It differs from *P. vohemarensis* sp. nov. in presence of irregular rugae on antennal scrobes, frons with foveolae, lower promesonotum, and petiolar peduncle with short horizontal lobes on its basal part. ***Minor workers*.** It differs from *P. vohemarensis* sp. nov. in promesonotum with posterior declivity smoothly declining towards propodeum, and foveolate katepisternum and mesonotum.

##### Pheidole vohemarensis sp. nov.

Taxon classificationAnimaliaHymenopteraFormicidae

http://zoobank.org/55119B1A-806B-491E-B551-8711DE650ACE

**Figure 38.**
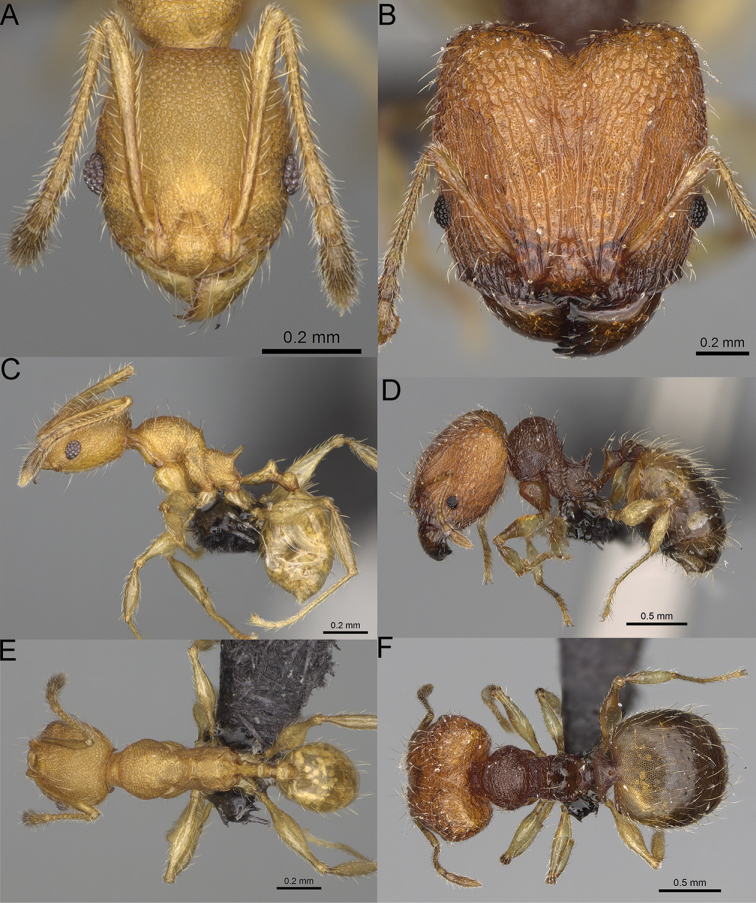
*Pheidole vohamarensis* sp. nov., full-face view (**A**), profile (**C**), and dorsal view (**E**) of paratype minor worker (CASENT0107947) and full-face view (**B**), profile (**D**), and dorsal view (**F**) of holotype major worker (CASENT0107946).

Figs 38A–F, 85AA, 88K

###### Type material.

***Holotype*.** Madagascar. •1 major worker; Antsiranana; Ambondrobe, 41.1 km 175° Vohemar; -13.71533, 50.10167; alt. 10 m; 1 Dec 2004; Fisher et al. leg.; BLF11264, CASENT0107946 (CASC). ***Paratypes*.** Madagascar. •2w., 1q.; same data as for holotype; CASENT0107947, CASENT0217986, CASENT0872084 (CASC).

###### Other material.

Madagascar. –***Antsiranana***: •1s.; Binara Forest; -13.26207, 49.60505; alt. 692 m; 20 Oct 2013; B.L. Fisher et al. leg.; CASENT0353324 (CASC). •2w., 1s.; Forêt d’Analabe, 30.0 km 72°ENE Daraina; -13.08333, 49.90833; alt. 30 m; 27 Nov 2003; B.L. Fisher et al. leg.; CASENT0041430, CASENT0041449, CASENT0048846 (CASC). •1w., 2s.; Forêt de Bekaraoka, 6.8 km 60°ENE Daraina; -13.16667, 49.71; alt. 150 m; 7 Dec 2003; B.L. Fisher et al. leg.; CASENT0044312, CASENT0044422, CASENT0044438 (CASC). •2w., 2s.; Forêt de Binara, 7.5 km 230°SW Daraina; -13.255, 49.61667; alt. 375 m; 1 Dec 2003; B.L. Fisher et al. leg.; CASENT0041832, CASENT0041833, CASENT0041890, CASENT0041895 (CASC). •1s.; Parc National de Marojejy, Manantenina River, 27.6 km 35°NE Andapa, 9.6 km 327°NNW Manantenina; -14.435, 49.76; alt. 775 m; 15 Nov 2003; B.L. Fisher et al. leg.; CASENT0045241 (CASC). •1w.; Rés. Analamerana, 28.4 km 99° Anivorano-Nord; -12.74667, 49.49483; alt. 60 m; 5 Dec 2004; B.L. Fisher et al. leg.; CASENT0054163 (CASC).

###### Diagnosis.

Head, in full-face view, cordate, widened posteriorly; sides of the head with dense, long, suberect to erect pilosity; antennal scrobes present, impressed, and indistinctly delimited ventrally and posteriorly by carinulae; scrobe surface foveolate, with distinct, thick, moderately sparse, longitudinal rugae; propodeal spines long (PSL: 0.19–0.23 (0.21)); gaster shagreened; inner hypostomal teeth distinct, high, narrow, and triangular, closely spaced, with rounded apex; outer hypostomal teeth slightly lower and thinner than inner hypostomal teeth, lobe-like. ***Minor workers*.** Head foveolate, genae with smooth notch; propodeal spines long (PSL: 0.12–0.14 (0.13)); promesonotum low, slightly convex, short, with posterior declivity relatively steep; mesosoma foveolate, katepisternum and mesonotum with smooth notches.

###### Description.

**Major workers.** Measurements (*N* = 10): HL: 1.03–1.11 (1.08); HW: 0.99–1.1 (1.05); SL: 0.49–0.57 (0.52); EL: 0.12–0.14 (0.13); WL: 0.83–0.91 (0.87); PSL: 0.19–0.23 (0.21); MTL: 0.48–0.54 (0.51); PNW: 0.48–0.57 (0.53); PTW: 0.13–0.17 (0.15); PPW: 0.4–0.51 (0.47); CI: 95.1–99.3 (98.0); SI: 47.1–53.1 (49.7); PSLI: 18.2–20.8 (19.1); PPI: 29.1–36.1 (33.1); PNI: 45.9–52.9 (49.9); MTI: 44.8–50.8 (48.8). ***Head*.** In full-face view cordate, slightly longer than wide, anterior of eyes slightly convex, posterior of eyes convex, occipital margins of lobes convex (Fig. 38B). In lateral view sub-oval; ventral and dorsal faces convex; dorsal face not depressed posteriorly; inner hypostomal teeth visible. Sides of the head with dense, long, suberect to erect pilosity; whole head with dense, short, suberect to erect pilosity. Antennal scrobes impressed and indistinctly delimited ventrally and posteriorly by carinulae; scrobe surface foveolate, with distinct, thick, moderately sparse, longitudinal rugae. Occipital lobes shiny, with thick, sparse, irregular rugae, rugae weakening posteriorly, interspaces with fine and dense rugulae; frons with moderately sparse, thick, longitudinal rugae, on the posterior part rugae longitudinal to irregular, interspaces with fine and dense rugulae, sometimes rugulae fading on the central part of frons; genae with dense, moderately thick, and irregular rugulae, interspaces with dense and fine rugulae; malar area with thick, sparse, longitudinal rugae, interspaces smooth to indistinctly rugulose. Centre of clypeus shiny and with indistinct, short rugulae, lateral sides with longitudinal rugae; median notch present, moderately wide and shallow; median longitudinal carina present; lateral longitudinal carinae present. Scape, when laid back, slightly exceeding the midlength of head; pilosity suberect to erect (Fig. 38B, D). Inner hypostomal teeth distinct, high, narrow, and triangular, closely spaced, with rounded apex; outer hypostomal teeth slightly lower and thinner than inner hypostomal teeth, lobe-like (Fig. 85AA). ***Mesosoma*.** In lateral view, promesonotum relatively low and arched, posterior mesonotum relatively steep, without tubercle-like projections; promesonotal groove absent; metanotal groove absent; propodeal spines long, triangular, thin, with sharp apex and moderately narrow base; humeral area laterally weakly produced (Fig. 38D). Surface foveolate, with additional sparse to dense, moderately thick rugoreticulation; dorsoventral propodeum with fading sculpture. Pilosity moderately sparse, long, erect (Fig. 38D, F). ***Petiole*.** Shiny; peduncle moderately long, foveolate, with very distinct, wide, horizontal lobes on its basal part; node relatively high, thin, with rounded apex, in rear view node with deep dorsoventral concavity; pilosity long and erect (Fig. 38D, F). ***Postpetiole*.** Shiny and foveolate; in dorsal view sides with long, wide, acute, and triangular projections; pilosity long and erect (Fig. 38D, F). ***Petiole*.** Shiny and shagreened; pilosity dense, long, and erect (Fig. 38D, F). ***Colour*.** Bright brown to dark brown, sometimes head with brighter colouration; legs yellow (Fig. 38D, F).

**Minor workers.** Measurements (*N* = 10): HL: 0.44–0.53 (0.49); HW: 0.4–0.45 (0.43); SL: 0.47–0.51 (0.5); EL: 0.08–0.11 (0.1); WL: 0.58–0.63 (0.6); PSL: 0.12–0.14 (0.13); MTL: 0.35–0.4 (0.37); PNW: 0.28–0.33 (0.3); PTW: 0.07–0.09 (0.08); PPW: 0.1–0.13 (0.11); CI: 82.6–94.3 (86.7); SI: 110.2–122.0 (115.8); PSLI: 23.4–30.9 (26.5); PPI: 60.4–82.9 (71.7); PNI: 67.2–75.3 (71.0); MTI: 81.0–90.9 (86.7). ***Head*.** Occipital margin straight or indistinctly concave; occipital carina indistinct (Fig. 38A). Pilosity moderately dense, long, suberect. Head foveolate; genae with smooth notch. Clypeus foveolate; median longitudinal carina absent; two lateral longitudinal carinae absent. Scape, when laid back, surpassing the posterior head margin by two-fifths of its length; pilosity erect (Fig. 38A, C). ***Mesosoma*.** In lateral view, promesonotum low, slightly convex, short, with posterior declivity relatively steep; promesonotal groove absent; metanotal groove present; propodeal spines long, triangular, with acute apex and narrow base (Fig. 38C). Sculpture foveolate; katepisternum and mesonotum with smooth notches. Pilosity moderately sparse, short, and erect (Fig. 38C, E). ***Petiole*.** Peduncle moderately short and thin; with few short, erect setae (Fig. 38C, E). ***Postpetiole*.** Short, low, and convex; with few short, erect setae (Fig. 38C, E). ***Petiole*.** With moderately sparse, erect pilosity (Fig. 38C, E). ***Colour*.** Yellow (Fig. 38C, E).

###### Etymology.

From type locality.

###### Biology.

The species was collected between 10–775 m in elevation, in tropical dry forest, littoral rainforest, and rainforest. Nests were located in rotten logs.

###### Comments.

*Pheidole vohemarensis* sp. nov. is most similar to *P. manantenensis* sp. nov. ***Major workers*.** It differs from *P. manantenensis* sp. nov. in presence of longitudinal rugae on antennal scrobes, frons never with foveolae, higher promesonotum and petiolar peduncle with wide and distinct horizontal lobes on its basal part. ***Minor workers*.** It differs from *P. vohemarensis* sp. nov. in promesonotum with posterior declivity relatively steep and katepisternum and mesonotum with smooth notches.

#### Revision of the *Pheidole longipilosa* complex

**Diagnosis. *Major workers*.** Head in full-face view sub-oval; sides of head with dense, very long, erect pilosity; antennal scrobes indistinct and not delimited by carinulae; occipital lobes with thick, irregular rugae, interspaces with distinct, irregular rugulae; genae shiny, with dense and thin, irregular rugulae, central part with smooth notch; promesonotum relatively high and arched; propodeal spines small to moderately long, triangular; promesonotum foveolate with additional indistinct, sparsely rugoreticulate, sometimes sculpture fading on dorsal surface and lower parts of lateral sides; gaster smooth; body reddish brown to bright brown. ***Minor workers*.** Scape, when laid back, surpassing the posterior head margin by one-fifth of its length; promesonotum low, convex, short; promesonotal and metanotal grooves absent; propodeal spines short, triangular; mesosoma foveolate; katepisternum smooth; anepisternum and mesonotum with fading sculpture and sometimes smooth; body yellow.

**Comments.** Major workers of this complex can be distinguished based on a combination of the following characters: head in full-face view elongated, in lateral view sub-oval; sides of the head with dense, long, erect pilosity; head sculptured but never with arcuate or transverse rugulae, central part of frons smooth; propodeal spines small to moderately long, triangular, and smooth gaster. Minor workers can be separated based on foveolate head and mesosoma with additional rugae on frons and smooth katepisternum, short and triangular propodeal spines, and yellow body colouration.

##### Pheidole longipilosa sp. nov.

Taxon classificationAnimaliaHymenopteraFormicidae

http://zoobank.org/88804AF2-4F5F-4A67-B2BD-9012E4F43AE9

**Figure 39.**
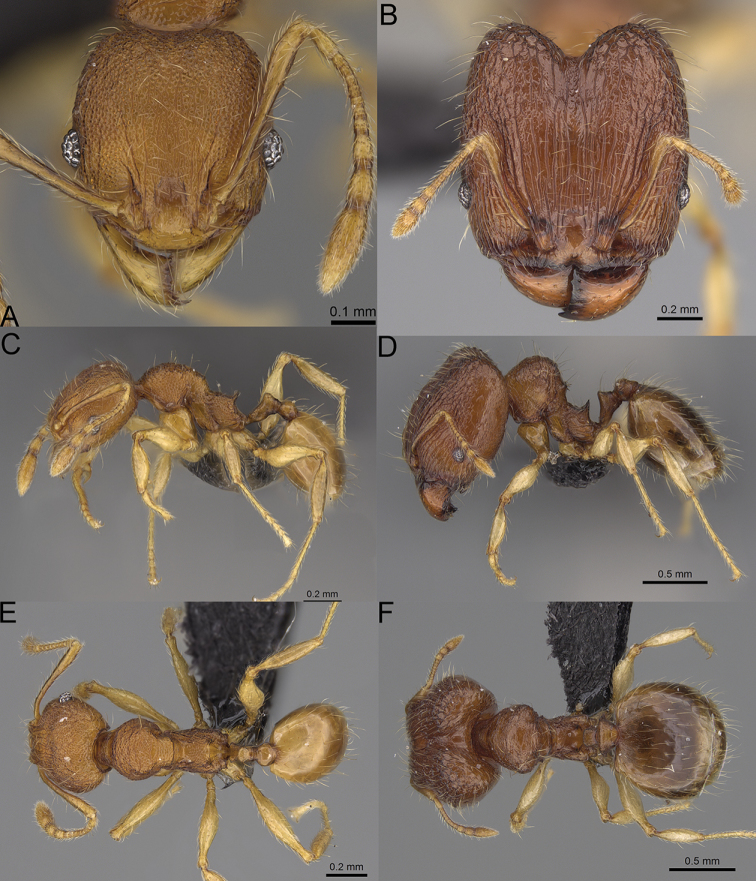
*Pheidole longipilosa* sp. nov., full-face view (**A**), profile (**C**), and dorsal view (**E**) of paratype minor worker (CASENT0235043) and full-face view (**B**), profile (**D**), and dorsal view (**F**) of holotype major worker (CASENT0498298).

Figs 39A–F , 84X , 87B

###### Type material.

***Holotype*.** Madagascar. •1 major worker; Toliara; Forêt Classée d’Analavelona, 29.2 km 343°NNW Mahaboboka; -22.675, 44.19; alt. 1100 m; 18 Feb 2003; B.L. Fisher et al. leg.; CASENT0498298 (CASC). ***Paratypes*.** Madagascar. •9w.; same data as for holotype; CASENT0235043, CASENT0498295–CASENT0498297, CASENT0872154–CASENT0872158 (CASC).

###### Other material.

Madagascar. –***Fianarantsoa***: •1w.; Parc National d’Isalo, Sahanafa River, 29.2 km 351°N Ranohira; -22.31333, 45.29167; alt. 500 m; 10 Feb 2003; Fisher et al. leg.; CASENT0031767 (CASC). –***Toliara***: •7w., 4s., 3q.; Forêt Classée d’Analavelona, 29.2 km 343°NNW Mahaboboka; -22.675, 44.19; alt. 1100 m; 18 Feb 2003; Fisher et al. leg.; CASENT0031011, CASENT0031099, CASENT0049501, CASENT0073507, CASENT0235043, CASENT0496936, CASENT0496937, CASENT0496938 (CASC). •1w., 5s.; Forêt Classée d’Analavelona, 29.4 km 343°NNW Mahaboboka; -22.675, 44.18667; alt. 1050 m; 21 Feb 2003; Fisher et al. leg.; CASENT0023935, CASENT0023938, CASENT0023943, CASENT0023946, CASENT0023947, CASENT0024032 (CASC).

###### Diagnosis.

***Major workers*.** Head in full-face view sub-oval; sides of the head with dense, long, erect pilosity; occipital lobes shiny, with thick, irregular rugae, interspaces with distinct, irregular rugulae not fading posteriorly; inner hypostomal teeth distinct, high, closely spaced, triangular, with rounded apex directed inward, and wide base; outer hypostomal teeth taller and wider than inner hypostomal teeth, lobe-like, directed outward; gaster smooth. ***Minor workers*.** Head shiny, foveolate, with additional short, longitudinal, thick rugae on frons, genae with fading sculpture and smooth notch; scape, when laid back, surpassing the posterior head margin by one-fifth of its length; promesonotum low, convex, short, with posterior declivity smoothly declining towards propodeum; mesosoma foveolate, katepisternum smooth; propodeal spines short, triangular.

###### Description.

**Major workers.** Measurements (*N* = 10): HL: 1.05–1.11 (1.09); HW: 0.98–1.04 (1.0); SL: 0.46–0.52 (0.48); EL: 0.13–0.16 (0.13); WL: 0.84–0.97 (0.88); PSL: 0.14–0.17 (0.15); MTL: 0.47–0.5 (0.49); PNW: 0.52–0.58 (0.54); PTW: 0.12–0.14 (0.13); PPW: 0.35–0.4 (0.37); CI: 90.0–93.9 (91.7); SI: 46.5–51.8 (48.1); PSLI: 12.5–15.4 (13.7); PPI: 31.9–39.0 (34.9); PNI: 53.1–55.5 (54.3); MTI: 45.8 –50.9 (48.8). ***Head*.** In full-face view longer than wide, anterior of eyes slightly convex, posterior of eyes convex (Fig. 39B). In lateral view sub-oval; ventral and dorsal faces convex; dorsal face not depressed posteriorly; inner hypostomal teeth visible. Sides of the head with dense, very long, erect pilosity; whole head with moderately dense, long, suberect to erect pilosity. Antennal scrobes indistinct and not delimited by carinulae; scrobe surface shiny, with thick, longitudinal, and long rugae; interspaces distinctly rugulose. Occipital lobes shiny, with thick, irregular rugae, interspaces with distinct, irregular rugulae not fading posteriorly; frons with moderately dense, thick, longitudinal rugae, interspaces smooth, with indistinct rugulae weakening anteriorly; genae shiny, with dense and thin, irregular rugulae, central part with smooth notch; malar area with thin, longitudinal, dense rugae, interspaces with distinct rugulae. Centre of clypeus shiny and smooth, lateral sides with longitudinal rugulae; median notch present, narrow, and moderately deep; median longitudinal carina present, indistinct; lateral longitudinal carinae present. Scape, when laid back, reaching the midlength of head; pilosity erect (Fig. 39B, D). Inner hypostomal teeth distinct, high, closely spaced, triangular, with rounded apex directed inward, and wide base; outer hypostomal teeth taller and wider than inner hypostomal teeth, lobe-like, directed outward (Fig. 84X). ***Mesosoma*.** In lateral view, promesonotum relatively high and arched, posterior mesonotum relatively steep, with tubercle-like projections; promesonotal groove absent; metanotal groove absent; propodeal spines small to moderately long, triangular, with sharp apex and wide base; humeral area laterally weakly produced (Fig. 39D). Surface shiny, promesonotum foveolate with additional indistinct, sparse rugoreticulations, sometimes sculpture fading on dorsal surface and lower parts of lateral sides; katepisternum rugoreticulate, with smooth notch; anepisternum and lateral sides of propodeum with distinct rugoreticulation; dorsoventral side of propodeum smooth or with indistinct sculpture. Pilosity moderately dense, very long, and erect (Fig. 39D, F). ***Petiole*.** Shiny; peduncle short, finely foveolate, with indistinct horizontal lobes on its basal part; node with fading sculpture, relatively low, triangular, with rounded apex, in rear view node slightly convex; pilosity long and erect (Fig. 39D, F). ***Postpetiole*.** Shiny, finely shagreened, with dorsum at least partially smooth; in dorsal view sides with moderately short, acute, and triangular projections; pilosity long, long and erect (Fig. 39D, F). ***Petiole*.** Shiny and smooth, only basal part of first gastral tergite finely shagreened; pilosity dense, long, and erect (Fig. 39D, F). ***Colour*.** Reddish brown to bright brown (Fig. 39D, F).

**Minor workers.** Measurements (*N* = 10): HL: 0.49–0.55 (0.51); HW: 0.44–0.47 (0.45); SL: 0.46–0.47 (0.46); EL: 0.09–0.11 (0.1); WL: 0.57–0.6 (0.58); PSL: 0.09–0.11 (0.1); MTL: 0.33–0.36 (0.34); PNW: 0.29–0.32 (0.31); PTW: 0.06–0.08 (0.07); PPW: 0.1–0.12 (0.11); CI: 85.7–90.4 (88.6); SI: 100.2–105.6 (103.0); PSLI: 18.1–21.8 (20.0); PPI: 56.5–64.5 (60.8); PNI: 65.9–70.1 (68.2); MTI: 73.5–80.1 (76.1). ***Head*.** Occipital margin straight or indistinctly concave; occipital carina absent (Fig. 39A). Pilosity moderately sparse, moderately long, suberect to erect. Head shiny, foveolate, with additional short, longitudinal, thick rugae on frons; genae with fading sculpture and smooth notch. Clypeus smooth, with basal part finely rugulose; median longitudinal carina present; two lateral longitudinal carinae indistinct. Scape, when laid back, surpassing the posterior head margin by one-fifth of its length; pilosity suberect to erect (Fig. 39A, C). ***Mesosoma*.** In lateral view, promesonotum low, convex, short, with posterior declivity smoothly declining towards propodeum; promesonotal groove absent; metanotal groove absent; propodeal spines short, triangular, with acute apex (Fig. 39C). Sculpture foveolate; katepisternum smooth; anepisternum and mesonotum with fading sculpture and sometimes smooth. Pilosity moderately sparse, long, and erect (Fig. 39C, E). ***Petiole*.** Peduncle short and thin with ventral face slightly convex; with few short, erect setae (Fig. 39C, E). ***Postpetiole*.** Short, low, and convex; with few short, erect setae (Fig. 39C, E). ***Petiole*.** With moderately sparse, erect pilosity (Fig. 39C, E). ***Colour*.** Yellow (Fig. 39C, E).

###### Etymology.

Latin for long pilosity, in reference to the very long pilosity on sides of head in major workers.

###### Biology.

The species was collected between 500–1100 m in elevation, in montane and gallery forests. Nests were located in rotten logs.

#### Revision of the *Pheidole annemariae* group

**Diagnosis. *Major workers*.** Head in full-face view rectangular, slightly longer than wide, in lateral view sub-rectangular; ventral and dorsal faces finely convex; dorsal face finely depressed posteriorly, forming shallow transverse depression; sides of the head with moderately dense, long, erect pilosity; antennal scrobes indistinct and not delimited by carinulae; scrobe surface foveolate to rugo-foveolate, with distinct, thin, moderately sparse to dense, longitudinal rugae; occipital lobes with thick, sparse, irregular rugae, interspaces with fine rugulae fading posteriorly; frons with moderately sparse to dense, thick, longitudinal, and sometimes interrupted rugae, interspaces smooth to rugulose; promesonotum low and arched; posterior mesonotum with distinct teeth-like projections; promesonotal and metanotal grooves absent; propodeal spines long, triangular; mesosoma sculpture well developed, foveolate to rugo-foveolate; gaster finely shagreened; body brown to brownish black. ***Minor workers*.** Head foveolate, genae smooth; scape, when laid back, reaching the posterior head margin or surpassing it by two-fifths of its length; promesonotum low, slightly convex, short; mesonotal spines present, small, and triangular; promesonotal groove absent; metanotal groove absent or indistinct; propodeal spines long, triangular; mesosoma foveolate; katepisternum and mesonotum with smooth notches or smooth; body yellow.

**Comments. **Major workers of this group can be distinguished based on a combination of the following characters: head in full-face view rectangular, and in lateral view sub-rectangular; scrobe surface foveolate to rugo-foveolate, with distinct and thin longitudinal rugae; posterior mesonotum with distinct teeth-like projections, and long, triangular propodeal spines. Minor workers can be separated based on foveolate head and mesosoma, with at least partly smooth genae, katepisternum, and mesonotum; presence of mesonotal spines and long propodeal spines.

This group contains two sympatric species: *P. annemariae* and *P. marieannae* sp. nov. Both species are known from northern part of the evergreen rainforest biome. The distribution of *Pheidole annemariae* spreads between Toamasina and Andapa, while *P. marieannae* is known from lowlands between Antalaha and Vohemar.

##### Key to the *P. annemariae* group

**Table:** 

1	Major workers. Genae with smooth notch, inner and outer hypostomal teeth connected by indistinct concavity, posterior mesonotum with distinct teeth-like projections, propodeal spines very long (Fig. 40A, E). Minor workers. Mesonotal spines distinct, small, and triangular, katepisternum, anepisternum, and mesonotum never entirely smooth (Fig. 40B)	***P. annemariae* Forel**
–	Major workers. Genae never with smooth notch, inner and outer hypostomal teeth not connected by concavity, posterior mesonotum with tubercle-like projections, propodeal spines moderately long (Fig. 40C, F). Minor workers. Mesonotal spines indistinct, katepisternum, anepisternum, and mesonotum entirely smooth (Fig. 40D)	***P. marieannae* sp. nov.**

**Figure 40.**
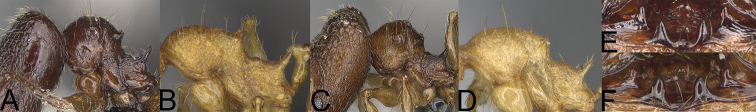
*Pheidole annemariae* Forel, head and profile of major worker (**A**), hypostomal teeth (**E**), profile of minor worker (**B**). *Pheidole marieannae* sp. nov., head of major worker (**C**), hypostomal teeth (**F**), profile of minor worker (**D**).

##### Pheidole annemariae

Taxon classificationAnimaliaHymenopteraFormicidae

Forel, 1918

**Figure 41.**
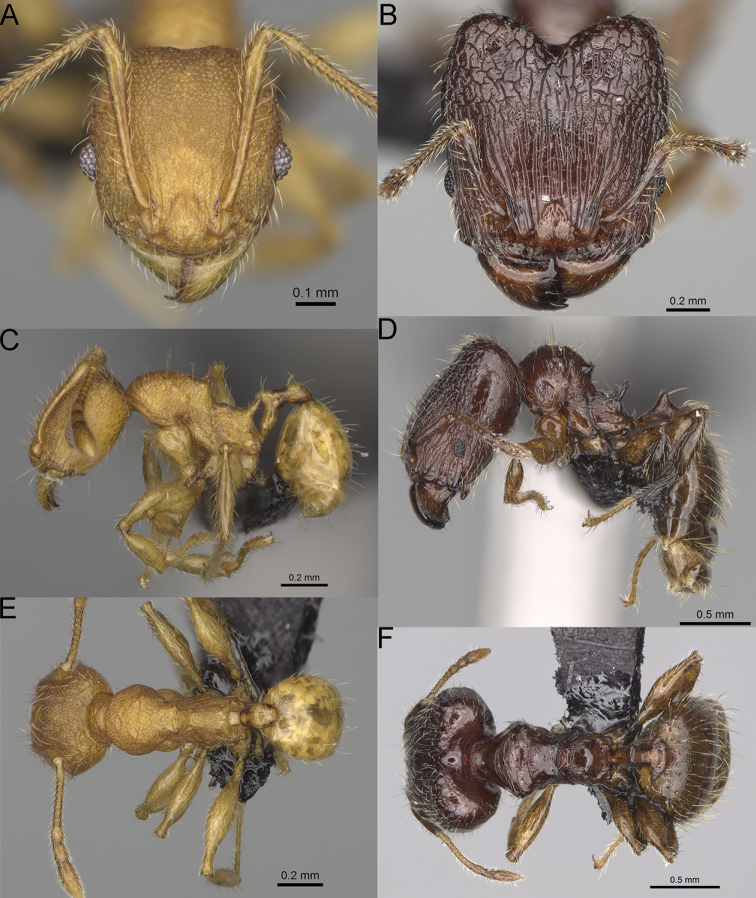
*Pheidole annemariae* Forel, full-face view (**A**), profile (**C**), and dorsal view (**E**) of minor worker (CASENT0077287) and full-face view (**B**), profile (**D**), and dorsal view (**F**) of major worker (CASENT0077290).

Figs 41A–F , 84E , 86E

###### Type material.

*Pheidole annemariae* Forel, 1918: 152 (s.w.q.). Lectotype [designated here]: major worker (middle specimen, CASENT0101688): Madagascar, Toamasina, Ilôt Prune, coll. Friederichs (MHNG) [examined]. Paralectotypes: 2 major workers (CASENT0810541, top and bottom specimens, the same pin as lectotype) (MHNG) [examined], 3 minor workers (CASENT0101829) (MHNG) [examined], 1 minor worker (CASENT0923206) (MHNG) [examined]: the same data as lectotype.

###### Other material.

Madagascar. –***Antsiranana***: •9w., 6s., 2q.; Ambondrobe, 41.1 km 175° Vohemar; -13.71533, 50.10167; alt. 10 m; 29 Nov 2004; B.L. Fisher et al. leg.; CASENT0056072, CASENT0056515, CASENT0056530, CASENT0056545, CASENT0056546, CASENT0056667, CASENT0056671, CASENT0107936, CASENT0109695, CASENT0109742, CASENT0110543, CASENT0110544 (CASC). •1w.; Binara Forest; -13.26392, 49.59919; alt. 1065 m; 18 Oct 2013; B.L. Fisher et al. leg.; CASENT0369879 (CASC). •3w., 1s., 1q.; Cap Est, Forêt d’Andranoanala; -15.24644, 50.46538; alt. 15 m; 16 Mar 2014; B.L. Fisher et al. leg.; CASENT0376154, CASENT0376165, CASENT0376166 (CASC). •7w., 3s.; Forêt de Binara, 9.1 km 233°SW Daraina; -13.26333, 49.60333; alt. 800 m; 5 Dec 2003; B.L. Fisher et al. leg.; CASENT0076461, CASENT0076462, CASENT0076463, CASENT0043234 (CASC). •4w., 1q.; Makirovana Forest; -14.17066, 49.95409; alt. 415 m; 28 Apr 2011; B.L. Fisher et al. leg.; CASENT0231267, CASENT0231268, CASENT0236175 (CASC). •4w.; Makirovana Forest; -14.16666, 49.95; alt. 715 m; 2 May 2011; B.L. Fisher et al. leg.; CASENT0231155, CASENT0231282 (CASC). •1w.; Masoala, Cap Est, Forêt d’Andranoanala; -15.26158, 50.4758; alt. 15 m; 15 Mar 2014; B.L. Fisher et al. leg.; CASENT0377896 (CASC). •9w., 3s.; Parc National de Marojejy, Manantenina River, 28.0 km 38°NE Andapa, 8.2 km 333°NNW Manantenina; -14.43667, 49.775; alt. 450 m; 12 Nov 2003; B.L. Fisher et al. leg.; CASENT0077287–CASENT0077290 (CASC). –***Toamasina***: •1w., 1s.; Nosy Mangabe, 7.43 km S Maroantsetra; -15.4973, 49.76223; alt. 3 m; 25 Jul 2007; B.L. Fisher et al. leg.; CASENT0129807 (CASC). •5w.; P.N. Masoala, 40 km 154°SSE Maroantsetra; -15.72667, 49.95667; alt. 150 m; 14 Oct 2001; Dejean et al. leg.; CASENT0004830, CASENT0004831, CASENT0004970–CASENT0004972 (CASC). •2w.; Tampolo, Masoala Peninsula, 40.4 km 154°SSE Maroantsetra; -15.73, 49.96; alt. 30 m; 28 Nov 2001; B.L. Fisher et al. leg.; CASENT0418227, CASENT0418228 (CASC).

###### Diagnosis.

Head in full-face view rectangular, slightly longer than wide, anterior of eyes slightly convex, posterior of eyes convex; sides of the head with moderately dense, long, erect pilosity; antennal scrobes present, impressed and not delimited; scrobe surface rugo-foveolate, with distinct, thin, moderately sparse, longitudinal rugae; propodeal spines long (PSL: 0.21–0.24 (0.22)); gaster finely shagreened; inner hypostomal teeth distinct, moderately high, closely spaced, triangular, with rounded apex and moderately narrow base; outer hypostomal teeth approximately the same size as inner hypostomal teeth, triangular; inner and outer teeth closely spaced and connected by indistinct concavity. ***Minor workers*.** Head foveolate, genae smooth; propodeal spines long (PSL: 0.15–0.18 (0.16)); promesonotum low, slightly convex, short, with posterior declivity relatively steep; mesonotal spines present; mesosoma foveolate, katepisternum and mesonotum with smooth notches.

###### Redescription.

**Major workers.** Measurements (*N* = 10): HL: 1.16–1.3 (1.21); HW: 1.06–1.19 (1.11); SL: 0.5–0.52 (0.51); EL: 0.13–0.16 (0.14); WL: 0.87–1.02 (0.93); PSL: 0.21–0.24 (0.22); MTL: 0.51–0.56 (0.53); PNW: 0.56–0.64 (0.59); PTW: 0.13–0.16 (0.15); PPW: 0.38–0.47 (0.43); CI: 90.7–93.9 (91.8); SI: 43.5–47.6 (46.2); PSLI: 17.6–20.1 (18.6); PPI: 31.1–38.1 (33.8); PNI: 51.2–55.2 (53.0); MTI: 44.7–50.0 (47.6). ***Head*.** In full-face view rectangular, slightly longer than wide, anterior of eyes slightly convex, posterior of eyes convex, occipital margins of lobes slightly convex (Fig. 41B). In lateral view sub-rectangular; ventral and dorsal faces finely convex; dorsal face finely depressed posteriorly, forming shallow transverse depression between frons and occipital lobes; inner hypostomal teeth visible. In lateral view weakly impressed on vertex. Sides of the head with moderately dense, long, erect pilosity; whole head with dense, moderately long, suberect to erect pilosity. Antennal scrobes indistinct and not delimited by carinulae; scrobe surface rugo-foveolate, with distinct, thin, moderately sparse, longitudinal rugae. Occipital lobes shiny, with thick, sparse, irregular rugae, interspaces with fine rugulae fading posteriorly; frons with moderately sparse, thick, longitudinal, and sometimes interrupted rugae, interspaces variable, smooth to finely rugoreticulate, most often rugoreticulation fading on the central part of frons; genae with dense, fine and irregular rugulae, area behind eyes with smooth notch; malar area with thick, sparse, longitudinal rugae, interspaces rugoreticulate. Centre of clypeus shiny and sometimes with indistinct, short rugulae, lateral sides with sparse and longitudinal rugae; median notch present, moderately wide and shallow; median longitudinal carina present; lateral longitudinal carinae present. Scape, when laid back, reaching the midlength of head; pilosity suberect to erect (Fig. 41B, D). Inner hypostomal teeth distinct, moderately high, closely spaced, triangular, with rounded apex and moderately narrow base; outer hypostomal teeth approximately the same size as inner hypostomal teeth, triangular; inner and outer teeth closely spaced and connected by indistinct concavity (Fig. 84E). ***Mesosoma*.** In lateral view, promesonotum low and arched, dorsal mesonotum finely concave, posterior mesonotum relatively steep, with distinct teeth-like projections; promesonotal groove absent; metanotal groove absent; propodeal spines long, triangular, with sharp apex and moderately wide base; humeral area laterally weakly produced (Fig. 41D). Surface variable; promesonotum finely foveolate to rugo-foveolate, foveolae or rugo-foveolae always fading on lateral sides of pronotum and promesonotal dorsum, sometimes dorsal surface with smooth notch on its centre, additional indistinct, sparse, and vertical rugulae cover promesonotal dorsum and sometimes also its lateral sides; anepisternum and katepisternum rugo-foveolate, katepisternum with smooth notch on its central part; propodeum rugo-foveolate, its dorsoventral surface and lower part of lateral sides with fading sculpture. Pilosity moderately dense, long, erect (Fig. 41D, F). ***Petiole*.** Shiny; peduncle moderately long, foveolate, with indistinct horizontal lobes on its basal part; node relatively high, moderately thick, with rounded apex, in rear view node with shallow dorsoventral concavity or straight; pilosity moderately sparse and erect (Fig. 41D, F). ***Postpetiole*.** Shiny and foveolate, its dorsum with fading sculpture; in dorsal view sides with moderately long, wide, acute, and triangular projections; pilosity long and erect (Fig. 41D, F). ***Petiole*.** Shiny and finely shagreened; pilosity moderately dense, long, and erect (Fig. 41D, F). ***Colour*.** Brown to brownish black; legs yellowish brown (Fig. 41D, F).

**Minor workers.** Measurements (*N* = 10): HL: 0.52–0.57 (0.54); HW: 0.48–0.54 (0.5); SL: 0.47–0.51 (0.48); EL: 0.11–0.12 (0.11); WL: 0.62–0.67 (0.64); PSL: 0.15–0.18 (0.16); MTL: 0.36–0.41 (0.39); PNW: 0.32–0.35 (0.34); PTW: 0.06–0.08 (0.07); PPW: 0.12–0.16 (0.13); CI: 89.3–94.6 (92.4); SI: 92.1–102.3 (97.6); PSLI: 29.3–31.9 (30.7); PPI: 50.0–60.5 (55.8); PNI: 65.1–70.4 (67.5); MTI: 73.5–80.3 (77.8). ***Head*.** Occipital margin straight or indistinctly concave; occipital carina indistinct (Fig. 41A). Pilosity moderately dense, long, suberect. Head foveolate; frons sometimes with fading sculpture; genae smooth. Clypeus finely foveolate with additional short, longitudinal rugulae; median longitudinal carina absent; two lateral longitudinal carinae absent. Scape, when laid back, surpassing the posterior head margin by two-fifths of its length; pilosity suberect to erect (Fig. 41A, C). ***Mesosoma*.** In lateral view, promesonotum low, slightly convex, short, with posterior declivity relatively steep; mesonotal spines present, small and triangular, promesonotal groove absent; metanotal groove absent; propodeal spines long, triangular, with acute apex and narrow base (Fig. 41C). Sculpture foveolate; katepisternum and mesonotum with smooth notches. Pilosity sparse, short, and erect (Fig. 41C, E). ***Petiole*.** Peduncle relatively short and thin; with few short, erect setae (Fig. 41C, E). ***Postpetiole*.** Short, low and convex; with few short, erect setae (Fig. 41C, E). ***Petiole*.** With sparse, erect pilosity (Fig. 41C, E). ***Colour*.** Yellow (Fig. 41C, E).

###### Biology.

The species was collected between 3–1343 m in elevation, in rainforest, littoral rainforest, montane rainforest, and occasionally beach vegetation on sandy soil. Nests were located in rotten logs and branches, and once in canopy.

###### Comments.

*Pheidole annemariae* is most similar to *P. marieannae* sp. nov. **Majors workers.** It differs from *P. marieannae* sp. nov. in presence of smooth notch on genae, inner and outer teeth closely spaced and connected by indistinct concavity, posterior mesonotum with distinct teeth-like projections, and longer propodeal spines. ***Minor workers*.** It differs from *P. marieannae* sp. nov. in presence of small but distinct mesonotal spines and katepisternum, anepisternum, and mesonotum never entirely smooth.

##### Pheidole marieannae sp. nov.

Taxon classificationAnimaliaHymenopteraFormicidae

http://zoobank.org/25AC316A-AFE3-4DB9-8C6C-EA34BCC6ACFD

**Figure 42.**
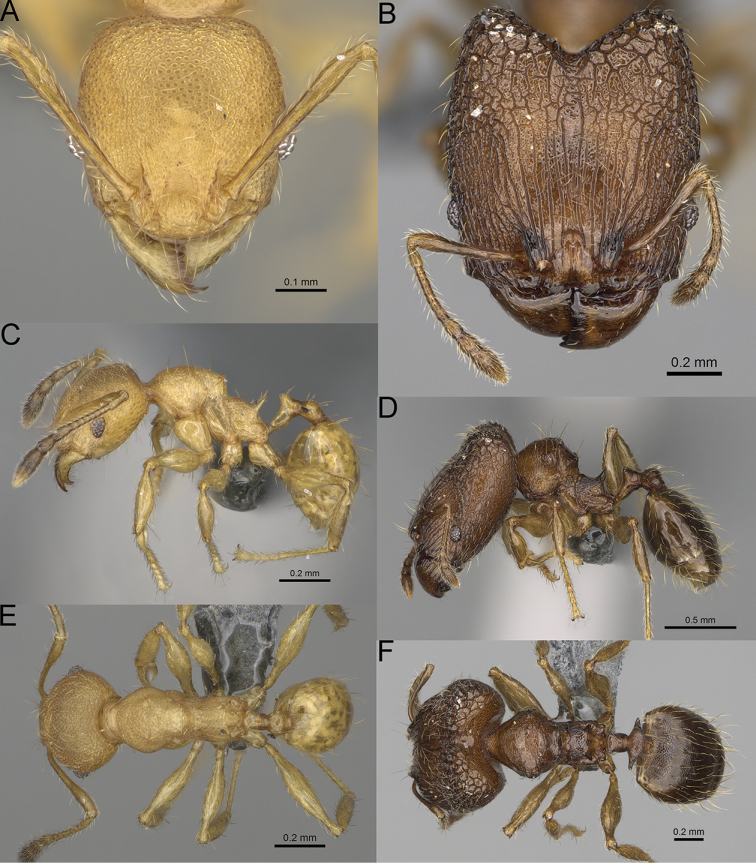
*Pheidole marieannae* sp. nov., full-face view (**A**), profile (**C**), and dorsal view (**E**) of paratype minor worker (CASENT0808178) and full-face view (**B**), profile (**D**), and dorsal view (**F**) of holotype major worker (CASENT0923219).

Figs 42A–F , 85H , 87L

###### Type material.

***Holotype*.** Madagascar. •1 major worker; Antsiranana; Sava Region: Parc National de Marojejy, near Manantenina River; -14.43677, 49.77541; alt. 475 m; 5 Feb 2018; Fisher et al. leg.; BLF40635, CASENT0923219 (CASC). ***Paratypes*.** Madagascar. •2w., 1q.; same data as for holotype; CASENT0808177, CASENT0808178, CASENT0872220 (CASC).

###### Other material.

Madagascar. –***Antsiranana***: •6w., 6s.; Ambondrobe, 41.1km 175° Vohemar; -13.71533, 50.10167; alt. 10 m; 29 Nov 2004; B.L. Fisher et al. leg.; CASENT0056064, CASENT0056072, CASENT0056523, CASENT0056661, CASENT0056664, CASENT0109696, CASENT0109743, CASENT0110541, CASENT0110669 (CASC). •10w., 5s., 1m.; Forêt Ambanitaza, 26.1 km 347° Antalaha; -14.67933, 50.18367; alt. 240 m; 26 Nov 2004; B.L. Fisher et al. leg.; CASENT0054895, CASENT0054958, CASENT0054962, CASENT0055564, CASENT0057274, CASENT0059041, CASENT0109525, CASENT0109533, CASENT0109546, CASENT0109546, CASENT0109547 (CASC). •5w., 2q.; Makirovana Forest; -14.104, 50.03574; alt. 225 m; 4 May 2011; B.L. Fisher et al. leg.; CASENT0212371, CASENT0212374, CASENT0212508, CASENT0230797 (CASC). •4w.; Makirovana Forest; -14.17066, 49.95409; alt. 415 m; 29 Apr 2011; B.L. Fisher et al. leg.; CASENT0231309, CASENT0243330, CASENT0243346 (CASC). •9w.; Makirovana Forest; -14.16044, 49.95216; alt. 550 m; 1 May 2011; B.L. Fisher et al. leg.; CASENT0212460, CASENT0230823, CASENT0231428, CASENT0231431, CASENT0243617, CASENT0243658, CASENT0245034 (CASC). •7w., 6s.; Parc National de Marojejy, Manantenina River, 28.0 km 38°NE Andapa, 8.2 km 333°NNW Manantenina; -14.43667, 49.775; alt. 450 m; 12 Nov 2003; B.L. Fisher et al. leg.; CASENT0045856, CASENT0045977, CASENT0045997, CASENT0046003, CASENT0046078, CASENT0046082, CASENT0048630, CASENT0077344, CASENT0077345 (CASC). •6w., 1s., 1q., 1m.; Sava Region: Parc National de Marojejy, near Manantenina River; -14.43677, 49.77541; alt. 475 m; 5 Feb 2018; B.L. Fisher et al. leg.; CASENT0808180, CASENT0808236, CASENT0808251, CASENT0808252, CASENT0825304, CASENT0825339, CASENT0826675 (CASC).

###### Diagnosis.

Head in full-face view rectangular, slightly longer than wide, anterior of eyes slightly convex, posterior of eyes convex; sides of the head with moderately dense, long, erect pilosity; antennal scrobes present, slightly impressed, and not delimited; scrobe surface foveolate, with distinct, thin, moderately dense, longitudinal rugae; propodeal spines moderately long (PSL: 0.16–0.21 (0.19)); gaster finely shagreened; inner hypostomal teeth distinct, moderately high, closely spaced, triangular, with rounded apex directed outward and moderately wide base; outer hypostomal teeth smaller and narrower than inner hypostomal teeth, triangular with moderately narrow base. ***Minor workers*.** Head foveolate, genae smooth; propodeal spines long (PSL: 0.11–0.19 (0.13)); promesonotum low, slightly convex, short, with posterior declivity steep; mesonotal spines present but indistinct; mesosoma foveolate, katepisternum, anepisternum, and mesonotum smooth.

###### Description.

**Major workers.** Measurements (*N* = 10): HL: 1.03–1.14 (1.07); HW: 0.91–1.02 (0.96); SL: 0.41–0.5 (0.44); EL: 0.11–0.13 (0.12); WL: 0.72–0.91 (0.78); PSL: 0.16–0.21 (0.19); MTL: 0.42–0.54 (0.45); PNW: 0.48–0.59 (0.53); PTW: 0.12–0.16 (0.14); PPW: 0.36–0.46 (0.41); CI: 86.2–92.6 (89.2); SI: 42.8–49.7 (46.1); PSLI: 15.1–19.4 (17.3); PPI: 31.5–36.7 (34.7); PNI: 51.2–60.2 (55.7); MTI: 43.9–53.3 (47.2). ***Head*.** In full-face view rectangular, slightly longer than wide, anterior of eyes slightly convex, posterior of eyes convex, occipital margins of lobes convex (Fig. 42B). In lateral view sub-rectangular; ventral and dorsal faces finely convex; dorsal face finely depressed posteriorly, forming shallow transverse depression between frons and occipital lobes; inner hypostomal teeth visible. In lateral view weakly impressed on vertex. Sides of the head with moderately dense, long, erect pilosity; whole head with moderately dense, short, suberect to erect pilosity. Antennal scrobes shallow and not delimited by carinulae; scrobe surface foveolate, with distinct, thin, moderately dense, longitudinal rugae. Occipital lobes shiny, with thick, sparse, irregular rugae, interspaces with fine rugulae fading posteriorly; frons with moderately dense, thick, longitudinal, and sometimes interrupted rugae, interspaces finely rugulose, most often rugulae fading on the central part of frons; genae with dense, irregular rugulae, area behind eyes with slightly fading sculpture; malar area with thick, sparse, longitudinal rugae, interspaces rugoreticulate. Centre of clypeus shiny and smooth, lateral sides with sparse and longitudinal rugae; median notch present, moderately wide, and shallow; median longitudinal carina present; lateral longitudinal carinae present. Scape, when laid back, reaching the midlength of head; pilosity suberect to erect (Fig. 42B, D). Inner hypostomal teeth distinct, moderately high, closely spaced, triangular, with rounded apex directed outward and moderately wide base; outer hypostomal teeth smaller and narrower than inner hypostomal teeth, triangular with moderately narrow base (Fig. 85H). ***Mesosoma*.** In lateral view, promesonotum relatively low and arched, dorsal mesonotum finely concave, posterior mesonotum relatively steep, with tubercle-like projections; promesonotal groove absent; metanotal groove absent; propodeal spines long, triangular, with sharp apex and moderately wide base; humeral area laterally weakly produced (Fig. 42D). Surface variable; promesonotum finely rugo-foveolate, rugo-foveolae always fading on lateral sides of pronotum and promesonotal dorsum; katepisternum and mesonotum smooth; anepisternum rugulae; propodeum rugoreticulate, its dorsoventral surface with fading sculpture. Pilosity moderately sparse, long, erect (Fig. 42D, F). ***Petiole*.** Shiny; peduncle relatively short, foveolate, with indistinct horizontal lobes on its basal part; node relatively high, moderately thick, with rounded apex, in rear view node indistinctly convex; pilosity moderately sparse and erect (Fig. 42D, F). ***Postpetiole*.** Shiny and shagreened; in dorsal view sides with moderately long, wide, acute, and triangular projections; pilosity moderately sparse and erect (Fig. 42D, F). ***Petiole*.** Shiny and finely shagreened; pilosity moderately dense, long, and erect (Fig. 42D, F). ***Colour*.** Brown to dark brown; legs yellowish brown (Fig. 42D, F).

**Minor workers.** Measurements (*N* = 10): HL: 0.41–0.49 (0.46); HW: 0.39–0.46 (0.42); SL: 0.39–0.47 (0.43); EL: 0.09–0.11 (0.1); WL: 0.47–0.58 (0.54); PSL: 0.11–0.19 (0.13); MTL: 0.28–0.37 (0.32); PNW: 0.25–0.31 (0.29); PTW: 0.06–0.07 (0.06); PPW: 0.1–0.13 (0.11); CI: 87.6–95.1 (92.2); SI: 97.1–114.1 (101.8); PSLI: 24.6–38.4 (28.3); PPI: 50.0–64.2 (56.2); PNI: 62.6–73.7 (67.6); MTI: 71.4–83.7 (76.4). ***Head*.** Occipital margin straight or indistinctly concave; occipital carina indistinct (Fig. 42A). Pilosity moderately dense, short, suberect. Head foveolate; genae smooth. Clypeus with median longitudinal carina absent; two lateral longitudinal carinae absent. Scape, when laid back, reaching the posterior head margin; pilosity suberect to erect (Fig. 42A, C). ***Mesosoma*.** In lateral view, promesonotum low, slightly convex, short, with posterior declivity steep; mesonotal spines present but indistinct; promesonotal groove absent; metanotal groove indistinct; propodeal spines long, triangular, with acute apex and narrow base (Fig. 42C). Sculpture foveolate; katepisternum, anepisternum, and mesonotum smooth. Pilosity sparse, moderately long, and erect (Fig. 42C, E). ***Petiole*.** Peduncle moderately short and thin with ventral face slightly convex; with few short, erect setae (Fig. 42C, E). ***Postpetiole*.** Short, low, and convex; with few short, erect setae (Fig. 42C, E). ***Petiole*.** With sparse and erect pilosity (Fig. 42C, E). ***Colour*.** Yellow (Fig. 42C, E).

###### Etymology.

Conversion of the name of the most similar species, *P. annemariae* Forel.

###### Biology.

The species was collected between 10–550 m in elevation, in rainforest and littoral rainforest. Nests were located in rotten logs, rotten sticks on ground, and soil.

###### Comments.

*Pheidole marieannae* sp. nov. is most similar to *P. annemariae*. ***Major workers*.** It differs from *P. annemariae* in absence of smooth notch on genae, inner and outer teeth never connected by indistinct concavity, posterior mesonotum with tubercle-like projections and shorter propodeal spines. ***Minor workers*.** It differs from *P. annemariae* in presence of indistinct mesonotal spines and katepisternum, anepisternum, and mesonotum entirely smooth.

#### Revision of the *Pheidole makaensis* group

**Diagnosis. *Major workers*.** Head in full-face view elongated, in lateral view sub-oval to sub-rectangular; ventral and dorsal faces convex; dorsal face not or finely depressed posteriorly; sides of the head with moderately dense, long to very long, erect pilosity; antennal scrobes indistinct and not delimited by carinulae; scrobe surface with thick, sparse, longitudinal rugae, interspaces smooth, rugo-foveolate, or foveolate, with thick, longitudinal, short, and interrupted rugae; occipital lobes always with arcuate and/or transverse rugae; genae smooth or with dense, thin, longitudinal rugulae and smooth notch in the centre; inner and outer teeth closely spaced and connected by concavity; promesonotum relatively low and arched; promesonotal groove absent; metanotal groove absent or indistinct; propodeal spines short to moderately long, triangular; mesosoma foveolate with additional sculpture; promesonotum with sculpture reduced; gaster finely shagreened, at least on the basal part of the first tergite; body dark yellow to brown. ***Minor workers*.** Head foveolate; genae smooth or with smooth notch; sometimes frons with additional longitudinal rugae; scape, when laid back, reaching the posterior margin of head or surpassing it by one- to two-fifths of its length; promesonotum low, convex, short; promesonotal groove absent; metanotal groove indistinct; propodeal spines small, triangular; mesosoma foveolate; anepisternum, katepisternum, and mesonotum sometimes with fading sculpture; body yellow to dark yellow.

**Comments.** Major workers can be distinguished based on a combination of the following characters: head in full-face view elongated; sides of the head with moderately dense, long to very long, erect pilosity; occipital lobes always with arcuate and/or transverse rugae; genae at least with a smooth notch; inner and outer teeth closely spaced and connected by concavity. Minor workers can be distinguished based on foveolate head and mesosoma, with genae at least with a smooth notch; small, triangular propodeal spines; yellow to dark yellow body colouration.

This species group contains five species: *P. makaensis* sp. nov., *P. fitarata* sp. nov., *P. rugofitarata* sp. nov., *P. ehazoara* sp. nov., and *P. avaratra* sp. nov. There are two species of this group known only from their type localities: *Pheidole ehazoara* sp. nov. collected in Ehazoara Canyon in Toliara and *P. makaensis* sp. nov. described from Makay Mts. in Toliara. *Pheidole fitarata* sp. nov. is sympatric with *P. avaratra* sp. nov. and distribution of both taxa is limited to northernmost parts of the Sambirano rainforest and dry deciduous forest biomes in the Antsiranana prefecture. *Pheidole rugofitarata* sp. nov. is known from area spread between Belo and Antonibe.

##### Key to the *P. makaensis* group

**Table:** 

1	Major workers. Frons with thick, sparse, and interrupted rugae, interspaces almost entirely smooth, outer hypostomal teeth lobe-like, distinctly bigger than inner hypostomal teeth (Fig. 43A, J). Minor workers. Pilosity on the whole body dense and very long (Fig. 43F)	***P. makaensis* sp. nov.**
–	Major workers. Frons with thick, dense to moderately dense, longitudinal or interrupted rugae, interspaces rugo-foveolate, foveolate or with rugulae, outer hypostomal teeth never lobe-like, approximately as big as inner hypostomal teeth or slightly higher (Fig. 43B–E, K–N). Minor workers. Pilosity on the whole body moderately sparse and never very long (Fig. 43G–I)	**2**
2	Major workers. Rugae on frons fading posteriorly and never connected with rugae on the occipital lobes, genae smooth, inner hypostomal teeth directed inward (Figs 43B, K, 44A). Minor workers. Genae smooth, katepisternum with smooth notch (Fig. 43G)	***P. fitarata* sp. nov.^2^**
–	Major workers. Rugae on frons not fading posteriorly and connected with rugae on the occipital lobes, genae never entirely smooth, inner hypostomal teeth never directed inward (Figs 43D, E, 44C, D). Minor workers. Genae with fading foveolae or only with smooth notch, katepisternum never smooth (Fig. 44H, I)	**3**
3	Major workers. Body dark yellow, promesonotum high and arched, frons with interspaces never rugo-foveolae, outer hypostomal teeth dentate and directed outward (Figs 43E, N, 44D). Minor workers. Scape reaching posterior margin of head, and head with additional longitudinal rugae on frons and malar area, propodeal spines never directed upward (Figs 43I, 44G)	***P. ehazoara* sp. nov.**
–	Major workers. Body yellowish brown to brown, promesonotum high and arched, dorsal mesonotum slightly concave, posterior mesonotum steep, frons with interspaces rugo-foveolae, outer hypostomal teeth dentate with relatively wide base, never directed outward (Figs 43D, M, 44C). Minor workers. Scape surpassing the posterior head margin by one-fifth of its length, and head without additional longitudinal rugae on frons and malae area, propodeal spines directed upward (Figs 43H, 44F)	***P. avaratra* sp. nov.**

**Figure 43.**
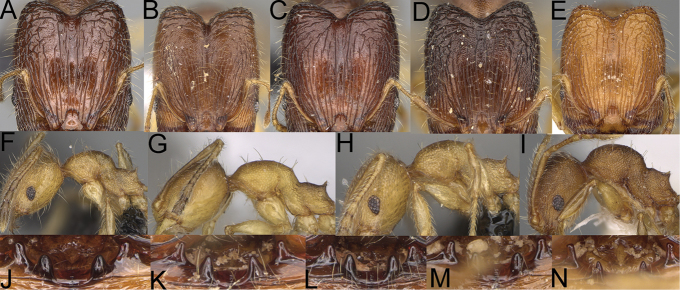
*Pheidole makaensis* sp. nov., head and profile of major worker (**A**), hypostomal teeth (**J**), profile of minor worker (**F**). *Pheidole fitarata* sp. nov., head and profile of major worker (**B**), hypostomal teeth (**K**), profile of minor worker (**G**). *Pheidole rugofitarata* sp. nov., head and profile of major worker (**C**), hypostomal teeth (**L**). *Pheidole avaratra* sp. nov., head and profile of major worker (**D**), hypostomal teeth (**M**), profile of minor worker (**H**). *Pheidole ehazoara* sp. nov., head and profile of major worker (**E**), hypostomal teeth (**N**), profile of minor worker (**I**).

**Figure 44.**
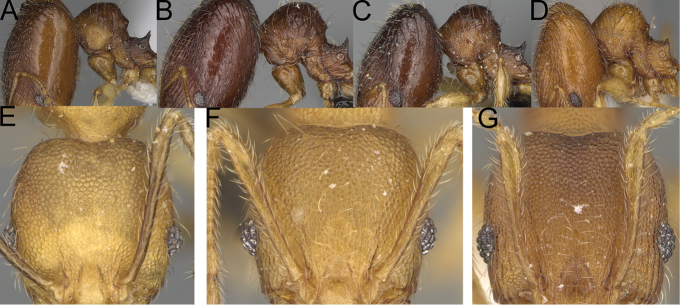
*Pheidole fitarata* sp. nov., profile of major worker (**A**), head of minor worker (**E**). *Pheidole rugofitarata* sp. nov., profile of major worker (**B**). *Pheidole avaratra* sp. nov., profile of major worker (**C**), head of minor worker (**F**). *Pheidole ehazoara* sp. nov., profile of major worker (**D**), head of minor worker (**G**).

##### Pheidole makaensis sp. nov.

Taxon classificationAnimaliaHymenopteraFormicidae

http://zoobank.org/48EF5FB3-DC3F-4B37-B662-E2CF5FE79681

**Figure 45.**
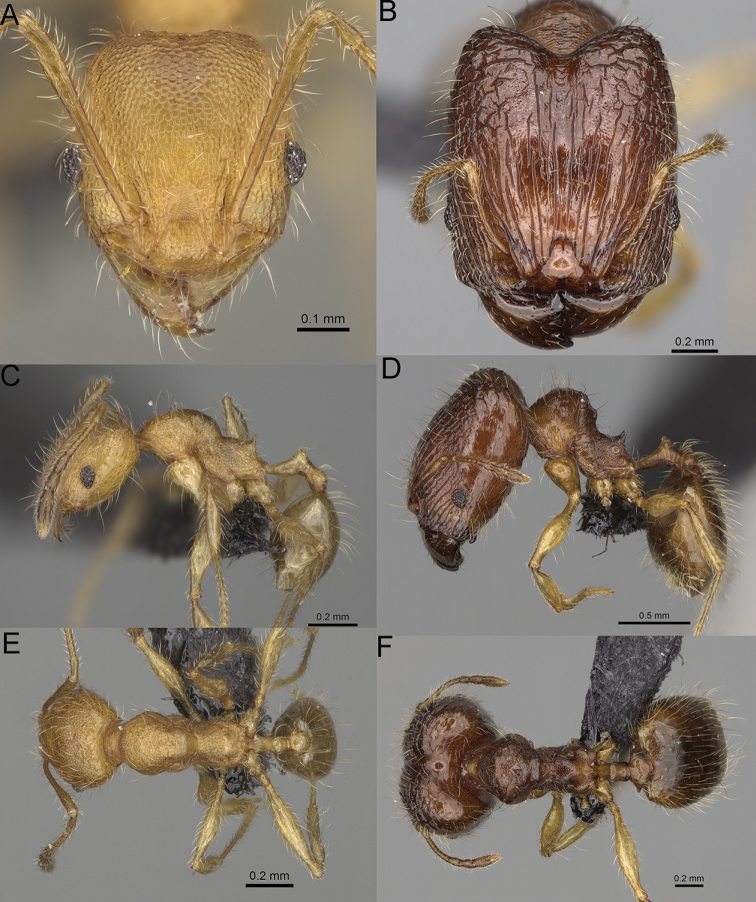
*Pheidole makaensis* sp. nov., full-face view (**A**), profile (**C**), and dorsal view (**E**) of paratype minor worker (CASENT0923188) and full-face view (**B**), profile (**D**), and dorsal view (**F**) of holotype major worker (CASENT0205745).

Figs 45A–F , 85D , 87H

###### Type material.

***Holotype*.** Madagascar. •1 major worker; Toliara; Makay Mts.; -21.25864, 45.16412; alt. 500 m; 8 Dec 2010; Fisher et al. leg.; BLF25762, CASENT0205745 (CASC). ***Paratype*.** Madagascar. •1w.; same data as for holotype; CASENT0923188 (CASC).

###### Diagnosis.

***Major workers*.** Head in full-face view elongated; sides of the head with moderately dense, very long, erect pilosity; occipital lobes with thick, sparse, short, irregular, and slightly arcuate rugae, interspaces smooth; inner hypostomal teeth distinct, moderately high, closely spaced, triangular, with rounded apex and wide base; outer hypostomal teeth bigger and wider than inner hypostomal teeth, lobe-like; inner and outer teeth closely spaced and connected by moderately deep concavity. ***Minor workers*.** Head foveolate, genae smooth; scape, when laid back, surpassing the posterior head margin by two-fifths of its length; promesonotum low, convex, short, with posterior declivity smoothly declining towards propodeum; mesosoma foveolate, anepisternum, katepisternum and mesonotum with fading sculpture; propodeal spines small, triangular.

###### Description.

**Major workers.** Measurements (*N* = 1): HL: 1.24; HW: 1.03; SL: 0.46; EL: 0.14; WL: 0.84; PSL: 0.16; MTL: 0.54; PNW: 0.51; PTW: 0.14; PPW: 0.34; CI: 83.5; SI: 44.2; PSLI: 13.3; PPI: 40.4; PNI: 49.0; MTI: 51.9. ***Head*.** In full-face view longitudinal, longer than wide, anterior of eyes straight, posterior of eyes slightly convex, occipital margin of lobes straight, inclining towards centre (Fig. 45B). In lateral view sub-oval; ventral and dorsal faces convex; dorsal face finely depressed posteriorly, forming indistinct transverse depression between frons and occipital lobes; inner hypostomal teeth visible. Sides of the head with moderately dense, very long, erect pilosity; whole head with moderately dense, long, suberect to erect pilosity. Antennal scrobes indistinct and not delimited by carinulae; scrobe surface shiny, with thick, sparse, longitudinal rugae; interspaces smooth and finely foveolate on the posterior part. Occipital lobes shiny, with thick, sparse, short, irregular, and slightly arcuate rugae, interspaces smooth; frons with moderately sparse, thick, longitudinal, interrupted rugae, interspaces smooth and indistinctly rugulae on the posterior part; genae shiny and smooth; malar area with thick, sparse, longitudinal rugae, interspaces smooth. Centre of clypeus shiny and smooth, lateral sides with longitudinal rugae; median notch present, narrow, and moderately deep; median longitudinal carina absent; lateral longitudinal carinae absent. Scape, when laid back, not reaching the midlength of head; pilosity erect (Fig. 45B, D). Inner hypostomal teeth distinct, moderately high, closely spaced, triangular, with rounded apex and wide base; outer hypostomal teeth bigger and wider than inner hypostomal teeth, lobe-like; inner and outer teeth closely spaced and connected by moderately deep concavity (Fig. 85D). ***Mesosoma*.** In lateral view, promesonotum relatively low and arched, dorsal mesonotum slightly concave, posterior mesonotum steep, with tubercle-like projections; promesonotal groove absent; metanotal groove absent; propodeal spines short, triangular, with sharp apex and relatively wide base; humeral area laterally weakly produced (Fig. 45D). Surface foveolate with additional indistinct and thin rugulae; dorsal and lateral surfaces of pronotum with fading sculpture and sometimes with smooth notches; katepisternum and anepisternum with sculpture reduced and smooth notches. Pilosity moderately dense, very long, and erect (Fig. 45D, F). ***Petiole*.** Shiny; peduncle moderately long, finely foveolate, with distinct, short, horizontal lobes on its basal part; node with fading sculpture, relatively low, triangular, with rounded apex, in rear view node slightly convex; pilosity moderately sparse and erect (Fig. 45D, F). ***Postpetiole*.** Shiny and finely shagreened, dorsum with fading sculpture; in dorsal view sides with very short, acute, and triangular projections; pilosity long, dense, and erect (Fig. 45D, F). ***Petiole*.** Shiny and finely shagreened; pilosity dense, very long, and erect (Fig. 45D, F). ***Colour*.** Reddish brown to brown; lateral sides of mesosoma, and sometimes malar area and lower frons, yellowish brown; legs yellow to yellowish brown (Fig. 45D, F).

**Minor workers.** Measurements (*N* = 1): HL: 0.48; HW: 0.41; SL: 0.44; EL: 0.1; WL: 0.55; PSL: 0.07; MTL: 0.35; PNW: 0.28; PTW: 0.07; PPW: 0.11; CI: 86.8; SI: 107.0; PSLI: 15.5; PPI: 64.6; PNI: 67.8; MTI: 84.3. ***Head*.** Occipital margin straight or indistinctly concave; occipital carina absent (Fig. 45A). Pilosity dense, very long, erect. Head shiny, foveolate; genae smooth. Clypeus smooth or finely foveolate; median longitudinal carina absent; two lateral longitudinal carinae absent. Scape, when laid back, surpassing the posterior head margin by two-fifths of its length; pilosity suberect to erect (Fig. 45A, C). ***Mesosoma*.** In lateral view, promesonotum low, convex, short, with posterior declivity smoothly declining towards propodeum; promesonotal groove absent; metanotal groove indistinct; propodeal spines small, triangular, with acute apex (Fig. 45C). Sculpture foveolate; anepisternum, katepisternum, and mesonotum with fading sculpture. Pilosity moderately sparse, long, and erect (Fig. 45C, E). ***Petiole*.** Peduncle short and thin with ventral face slightly convex; node moderately low, globular, and small; with few short, erect setae (Fig. 45C, E). ***Postpetiole*.** Short, low, and slightly convex; with few short, erect setae (Fig. 45C, E). ***Petiole*.** With moderately sparse, very long, erect pilosity (Fig. 45C, E). ***Colour*.** Yellow (Fig. 45C, E).

###### Etymology.

From the type locality.

###### Biology.

The species was collected at an elevation of 500 m, in gallery forest with bamboo. Nest was located in dead twig above ground.

###### Comments.

*Pheidole makaensis* sp. nov. is most similar to *P. avaratra* sp. nov. ***Major workers*.** It differs from *P. avaratra* sp. nov. in very sparse and interrupted rugae on frons and frons with mostly smooth interspaces; smooth genae, lobe-like outer hypostomal teeth which are distinctly bigger than inner hypostomal teeth, and katepisternum with smooth notch. ***Minor workers*.** It differs from *P. avaratra* sp. nov. in denser and longer pilosity on head, mesosoma, and gaster.

##### Pheidole fitarata sp. nov.

Taxon classificationAnimaliaHymenopteraFormicidae

http://zoobank.org/8CF00FF0-5B19-4EA6-803E-27F62D848627

**Figure 46.**
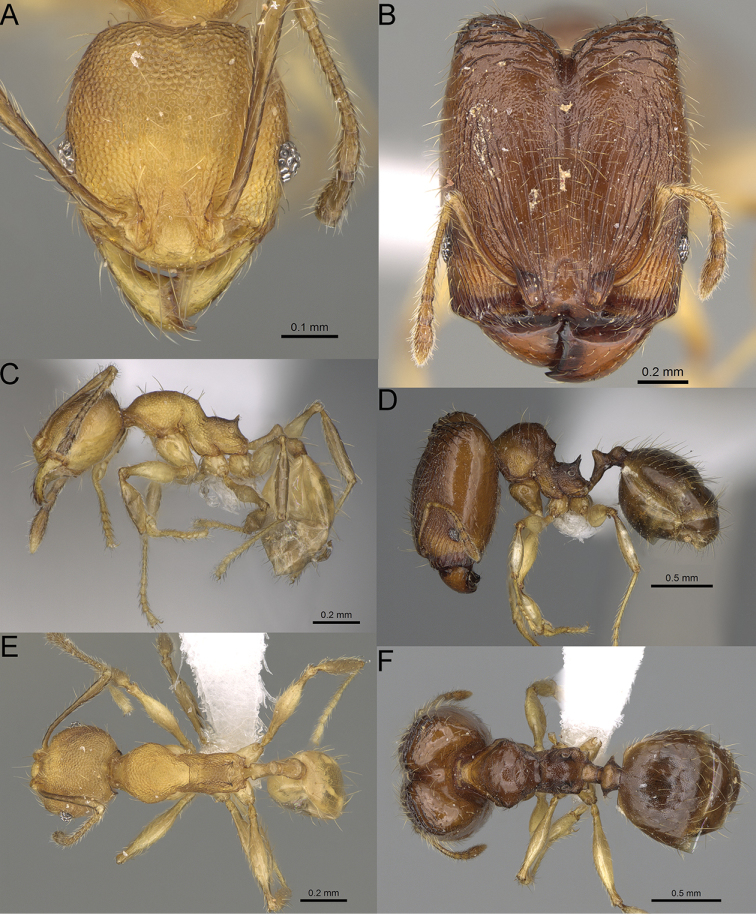
*Pheidole fitarata* sp. nov., full-face view (**A**), profile (**C**), and dorsal view (**E**) of paratype minor worker (CASENT0420025) and full-face view (**B**), profile (**D**), and dorsal view (**F**) of holotype major worker (CASENT0420028).

Figs 46A–F , 84S , 86S

###### Type material.

***Holotype*.** Madagascar. •1 major worker; Antsiranana; Ampasindava, Forêt d’Ambilanivy, 3.9 km 181°S Ambaliha; -13.79861, 48.16167; alt. 600 m; 4 Mar 2001; Fisher et al. leg.; BLF03293, CASENT0420028 (CASC). ***Paratypes*.** Madagascar. •4w., 1s.; same data as for holotype; CASENT0420024–CASENT0420027, CASENT0872215–CASENT0872217 (CASC).

###### Other material.

Madagascar. –***Antsiranana***: •1s.; Ambondrobe, 41.1km 175° Vohemar; -13.71533, 50.10167; alt. 10 m; 29 Nov 2004; B.L. Fisher leg.; CASENT0056078 (CASC). •1w., 4s.; Ampasindava, Forêt d’Ambilanivy, 3.9 km 181°S Ambaliha; -13.79861, 48.16167; alt. 600 m; 4 Mar 2001; B.L. Fisher et al. leg.; CASENT0406700, CASENT0420217, CASENT0420226, CASENT0464109, CASENT0464294 (CASC). •2s.; Forêt d’ Andavakoera, 21.4 km 75°ENE Ambilobe; 4.6 km 356°N Betsiaka; -13.11833, 49.23; alt. 425 m; 15 Dec 2003; B.L. Fisher leg.; CASENT0044135, CASENT0044209 (CASC). •1s.; Montagne des Français, 7.2 km 142°SE Antsiranana (=Diego Suarez); -12.32278, 49.33817; alt. 180 m; 22 Feb 2001; B.L. Fisher et al. leg.; CASENT0461044 (CASC). •1s.; Nosy Be, Réserve Naturelle Intégrale de Lokobe, 6.3 km 112°ESE Hellville; -13.41933, 48.33117; alt. 30 m; 19 Mar 2001; B.L. Fisher et al. leg.; CASENT0462968 (CASC). •1s.; R.S. Manongarivo, 12.8 km 228°SW Antanambao; -13.97667, 48.42333; alt. 780 m; 11 Oct 1998; B.L. Fisher leg.; CASENT0198865 (CASC). •3s.; Réserve Spéciale d’Ambre, 3.5 km 235°SW Sakaramy; -12.46889, 49.24217; alt. 325 m; 26 Jan 2001; B.L. Fisher et al. leg.; CASENT0406576, CASENT0423829, CASENT0423892 (CASC). •1w., 1s.; Réserve Spéciale de l’Ankarana, 13.6 km 192°SSW Anivorano Nord; -12.86361, 49.22583; alt. 210 m; 16 Feb 2001; B.L. Fisher et al. leg.; CASENT0420164, CASENT0420181 (CASC). •7s., 1q.; Réserve Spéciale de l’Ankarana, 22.9 km 224°SW Anivorano Nord; -12.90889, 49.10983; alt. 80 m; 10 Feb 2001; B.L. Fisher et al. leg.; CASENT0420207, CASENT0439168, CASENT0439173, CASENT0439183, CASENT0439197, CASENT0439313, CASENT0439315, CASENT0008034 (CASC).

###### Diagnosis.

***Major workers*.** Head in full-face view elongated; sides of the head with moderately dense, long, erect pilosity; occipital lobes smooth to indistinctly foveolate, with thick, sparse, short, transverse, and sometimes arcuate rugae; inner hypostomal teeth distinct, high, closely spaced, triangular, with rounded apex directed inward; outer hypostomal teeth thinner than inner hypostomal teeth and approximately the same height, triangular. ***Minor workers*.** Head foveolate, genae smooth; scape, when laid back, surpassing the posterior head margin by two-fifths of its length; promesonotum low, convex, short, with posterior declivity relatively convex; mesosoma foveolate, katepisternum with smooth notch; propodeal spines short, triangular.

###### Description.

**Major workers.** Measurements (*N* = 10): HL: 1.13–1.43 (1.29); HW: 0.91–1.12 (1.07); SL: 0.43–0.53 (0.49); EL: 0.12–0.17 (0.14); WL: 0.79–1.02 (0.92); PSL: 0.16–0.21 (0.18); MTL: 0.51–0.59 (0.55); PNW: 0.45–0.64 (0.56); PTW: 0.11–0.19 (0.16); PPW: 0.29–0.47 (0.41); CI: 77.9–88.4 (82.7); SI: 43.5–49.0 (46.5); PSLI: 12.7–15.3 (14.0); PPI: 34.8–42.9 (38.2); PNI: 49.1–56.7 (52.8); MTI: 48.4 –56.1 (52.0). ***Head*.** In full-face view longitudinal, longer than wide, anterior of eyes straight, posterior of eyes slightly convex, occipital margin of lobes straight, inclining toward centre (Fig. 46B). In lateral view sub-rectangular; ventral and dorsal faces finely convex; dorsal face finely depressed posteriorly, forming indistinct transverse depression between frons and occipital lobes; inner hypostomal teeth visible. Sides of the head with moderately dense, long, erect pilosity; whole head with dense, moderately long, suberect to erect pilosity. Antennal scrobes indistinct and not delimited by carinulae; scrobe surface shiny, foveolate, with thick, longitudinal, short, and interrupted rugae. Occipital lobes shiny, smooth to indistinctly foveolate, with thick, sparse, short, transverse, and sometimes arcuate rugae; frons with moderately dense, thick, longitudinal and interrupted rugae, interspaces smooth and indistinctly foveolate on the posterior part, rugae weakening posteriorly and never connected with rugae on the occipital lobes; genae shiny and smooth; malar area with thick, longitudinal, moderately sparse rugae, interspaces smooth. Centre of clypeus shiny and smooth, lateral sides with longitudinal rugae; median notch present, wide, and shallow; median longitudinal carina absent; lateral longitudinal carinae absent. Scape, when laid back, not reaching the mid-length of head; pilosity suberect to erect (Fig. 46B, D). Inner hypostomal teeth distinct, high, closely spaced, triangular, with rounded apex directed inward; outer hypostomal teeth thinner than inner hypostomal teeth and approximately the same height, triangular (Fig. 84S). ***Mesosoma*.** In lateral view, promesonotum relatively low and arched, posterior mesonotum relatively steep, with tubercle-like projections; promesonotal groove absent; metanotal groove indistinct; propodeal spines moderately long, triangular, with sharp apex and wide base; humeral area laterally weakly produced (Fig. 46D). Surface finely foveolate, dorsal surface of promesonotum with fading sculpture and sometimes with smooth notch; katepisternum with smooth notch; lower half of lateral sides of propodeum with additional longitudinal rugae. Pilosity sparse, very long, and erect (Fig. 46D, F). ***Petiole*.** Shiny; peduncle moderately long, finely foveolate, with indistinct horizontal lobes on its basal part; node smooth, relatively high, triangular, with rounded apex, in rear view node slightly convex; pilosity moderately sparse and erect (Fig. 46D, F). ***Postpetiole*.** Shiny and finely shagreened, dorsum with fading sculpture; in dorsal view sides with moderately short, acute, and triangular projections; pilosity long to moderately long, and erect (Fig. 46D, F). ***Petiole*.** Shiny and finely shagreened; pilosity moderately dense, long, and erect (Fig. 46D, F). ***Colour*.** Bright brown to brown; legs, lower frons, malar area and lower parts of lateral sides of mesosoma yellowish brown (Fig. 46D, F).

**Minor workers.** Measurements (*N* = 6): HL: 0.45–0.47 (0.46); HW: 0.39–0.41 (0.4); SL: 0.44–0.46 (0.45); EL: 0.07–0.1 (0.08); WL: 0.52–0.56 (0.55); PSL: 0.08–0.09 (0.08); MTL: 0.33–0.36 (0.35); PNW: 0.26–0.28 (0.27); PTW: 0.06–0.06 (0.06); PPW: 0.09–0.1 (0.1); CI: 85.9–89.5 (87.7); SI: 111.0–113.6 (111.6); PSLI: 17.3–20.4 (18.5); PPI: 61.0–65.2 (62.5); PNI: 66.4–68.8 (67.8); MTI: 82.8–89.4 (86.7). ***Head*.** Occipital margin straight or indistinctly concave; occipital carina absent (Fig. 46A). Pilosity moderately dense, long, suberect. Head shiny, foveolate; genae smooth. Clypeus smooth or finely foveolate; median longitudinal carina absent; two lateral longitudinal carinae absent. Scape, when laid back, surpassing the posterior head margin by two-fifths of its length; pilosity suberect to erect (Fig. 46A, C). ***Mesosoma*.** In lateral view, promesonotum low, convex, short, with posterior declivity relatively convex; promesonotal groove absent; metanotal groove indistinct; propodeal spines short, triangular, with acute apex (Fig. 46C). Sculpture foveolate; katepisternum with smooth notch. Pilosity sparse, long, and erect (Fig. 46C, E). ***Petiole*.** Peduncle short and thin with ventral face slightly convex; with few short, erect setae (Fig. 46C, E). ***Postpetiole*.** Short, low, and convex; sometimes with few short, erect setae (Fig. 46C, E). ***Petiole*.** With moderately sparse, erect pilosity (Fig. 46C, E). ***Colour*.** Yellow (Fig. 46C, E).

###### Etymology.

Malagasy for mirror, in reference to smooth and shiny genae of major workers.

###### Biology.

The species was collected between 10–780 m in elevation, in rainforest, in tropical dry forest, and in littoral rainforest. Nests were located rotten logs.

###### Comments.

*Pheidole fitarata* sp. nov. is most similar to *P. rugofitarata* sp. nov. and *P. avaratra* sp. nov. ***Major workers*.***Pheidole fitarata* sp. nov. differs from *P. rugofitarata* sp. nov. in less distinct and interrupted longitudinal rugae on frons and antennal scrobes, lack of connection between rugae on frons and occipital lobes, smooth genae, and never lobe-like outer hypostomal teeth; from *P. avaratra* sp. nov. in presence of longitudinal, thick, and interrupted rugae on frons and antennal scrobes, frons with rugae weakening posteriorly and never connected with rugae on the occipital lobes, genae smooth, and smaller and directed inward inner hypostomal teeth. ***Minor workers*.***Pheidole fitarata* sp. nov. differs from *P. avaratra* sp. nov. in longer and lower promesonotum and katepisternum with smooth notch.

##### Pheidole rugofitarata sp. nov.

Taxon classificationAnimaliaHymenopteraFormicidae

http://zoobank.org/273DEBF3-9074-43F2-BE54-06465A5D17E3

**Figure 47.**
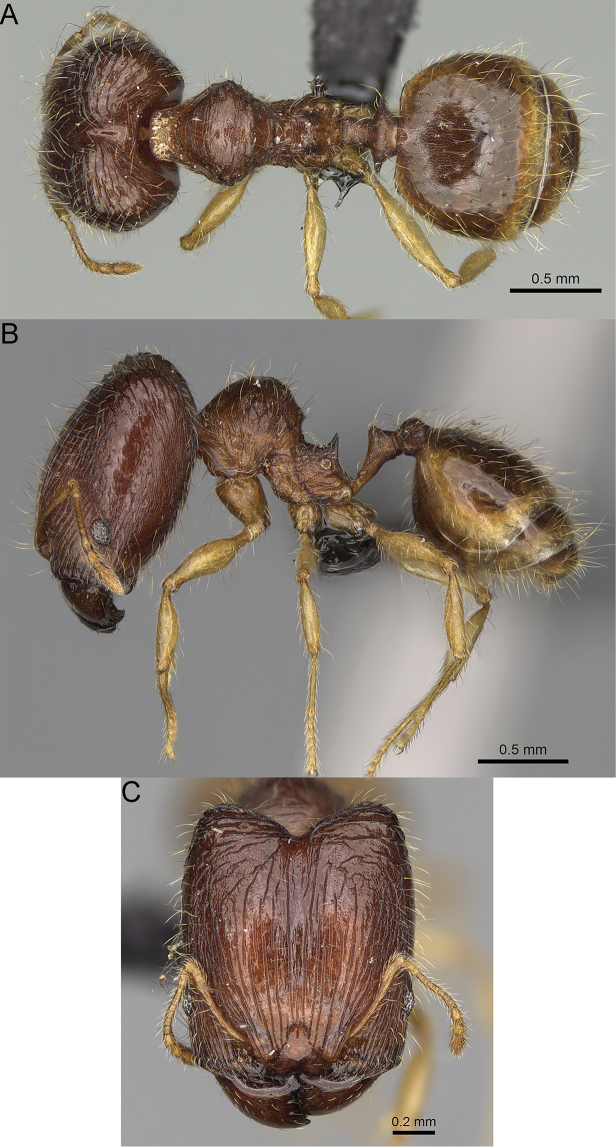
*Pheidole rugofitarata* sp. nov., dorsal view (**A**), profile (**B**), and full-face view (**C**) of holotype major worker (CASENT0078206).

Figs 47A–C , 85V , 88F

###### Type material.

***Holotype*.** Madagascar. •1 major worker; Mahajanga; Parc National Tsingy de Bemaraha, 2.5 km 62°ENE Bekopaka, Ankidrodroa River; -19.13222, 44.81467; alt. 100 m; 11 Nov 2001; Fisher et al. leg.; BLF04341, CASENT0078206 (CASC). ***Paratype*.** Madagascar. •1s.; same data as for holotype; CASENT0078208 (CASC).

###### Other material.

Madagascar. – ***Mahajanga***: •2s.; Forêt Ambohimanga, 26.1 km 314° Mampikony; -15.96267, 47.43817; alt. 250 m; 13 Dec 2004; Fisher et al. leg.; CASENT0054800, CASENT0055004 (CASC). •2s.; Forêt de Tsimembo, 11.0 km 346°NNW Soatana; -18.99528, 44.4435; alt. 50 m; 21 Nov 2001; Fisher et al. leg.; CASENT0483554, CASENT0483640 (CASC). •1s.; Parc National d’Ankarafantsika, Ampijoroa Station Forestière, 5.4 km 331°NW Andranofasika; -16.29889, 46.813; alt. 70 m; 26 Mar 2001; Fisher et al. leg.; CASENT0468979 (CASC). •3s.; Parc National d’Ankarafantsika, Forêt de Tsimaloto, 18.3 km 46°NE de Tsaramandroso; -16.22806, 47.14361; alt. 135 m; 2 Apr 2001; Fisher et al. leg.; CASENT0431979, CASENT0432158, CASENT0432186 (CASC). •1s.; Parc National de Baie de Baly, 12.4 km 337°NNW Soalala; -16.01, 45.265; alt. 10 m; 26 Nov 2002; Fisher et al. leg.; CASENT0025410 (CASC). •5s.; Parc National de Namoroka, 16.9 km 317°NW Vilanandro; -16.40667, 45.31; alt. 100 m; 12 Nov 2002; Fisher et al. leg.; CASENT0038862, CASENT0023652, CASENT0023665, CASENT0023667, CASENT0493106 (CASC). •2s.; Parc National de Namoroka, 9.8 km 300°WNW Vilanandro; -16.46667, 45.35; alt. 140 m; 4 Nov 2002; Fisher et al. leg.; CASENT0032768, CASENT0492055 (CASC). •4s.; Parc National Tsingy de Bemaraha, 3.4 km 93°E Bekopaka, Tombeau Vazimba; -19.14194, 44.828; alt. 50 m; 6 Nov 2001; Fisher et al. leg.; CASENT0078127, CASENT0477444, CASENT0477453, CASENT0477482 (CASC). •4s.; Réserve d’Ankoririka, 10.6 km 13°NE de Tsaramandroso; -16.26722, 47.04861; alt. 210 m; 9 Apr 2001; Fisher et al. leg.; CASENT0471030, CASENT0471209, CASENT0484762 (CASC).

###### Diagnosis.

***Major workers*.** Head in full-face view elongated; sides of the head with moderately dense, long, erect pilosity; occipital lobes indistinctly foveolate, with thick, sparse, short, transverse, and sometimes arcuate rugae; inner hypostomal teeth distinct, high, closely spaced, triangular, with rounded apex; outer hypostomal teeth wider and higher than inner hypostomal teeth, lobe-like.

###### Description.

**Major workers.** Measurements (*N* = 10): HL: 1.29–1.41 (1.36); HW: 1.05–1.17 (1.12); SL: 0.48–0.53 (0.5); EL: 0.14–0.17 (0.15); WL: 0.95–1.02 (0.99); PSL: 0.17–0.21 (0.18); MTL: 0.54–0.57 (0.55); PNW: 0.58–0.68 (0.62); PTW: 0.14–0.17 (0.16); PPW: 0.38–0.47 (0.43); CI: 81.2–85.8 (82.9); SI: 41.8–49.0 (44.3); PSLI: 12.2–15.2 (13.5); PPI: 34.0–42.1 (37.3); PNI: 53.1–59.0 (55.3); MTI: 46.9 –52.5 (49.1). ***Head*.** In full-face view longitudinal, longer than wide, anterior of eyes straight, posterior of eyes slightly convex, occipital margin of lobes straight, inclining toward centre (Fig. 47C). In lateral view sub-oval; ventral and dorsal faces convex; dorsal face not depressed posteriorly; inner hypostomal teeth visible. Sides of the head with moderately dense, long, erect pilosity; whole head with moderately dense, long, suberect to erect pilosity. Antennal scrobes indistinct and not delimited by carinulae; scrobe surface shiny, with moderately dense, longitudinal rugae, interspaces superficially foveolate. Occipital lobes shiny, indistinctly foveolate, with thick, sparse, short, transverse and sometimes arcuate rugae; frons with moderately dense, thick, longitudinal rugae, interspaces smooth and indistinctly foveolate on the posterior part; genae shiny, with dense, thin, longitudinal rugulae and smooth notch in the centre; malar area with thick, longitudinal, moderately sparse rugae, interspaces smooth. Centre of clypeus shiny and smooth, lateral sides with longitudinal rugae; median notch present, wide and shallow; median longitudinal carina absent; lateral longitudinal carinae absent. Scape, when laid back, not reaching the midlength of head; pilosity suberect to erect (Fig. 47B, C). Inner hypostomal teeth distinct, high, closely spaced, triangular, with rounded apex; outer hypostomal teeth wider and higher than inner hypostomal teeth, lobe-like (Fig. 85V). ***Mesosoma*.** In lateral view, promesonotum relatively low and arched, posterior mesonotum relatively steep, with tubercle-like projections; promesonotal groove absent; metanotal groove indistinct; propodeal spines moderately long, triangular, with sharp apex and wide base; humeral area laterally weakly produced (Fig. 47B). Surface finely foveolate with additional irregular, thin, and sparse rugoreticulation; dorsal surface of promesonotum with fading foveolae and thin, sparse, and transverse rugulae; katepisternum with reduced sculpture. Pilosity moderately dense, very long and erect (Fig. 47A, B). ***Petiole*.** Shiny; peduncle moderately long, finely foveolate, with indistinct horizontal lobes on its basal part; node smooth, relatively high, triangular, with rounded apex, in rear view node slightly convex; pilosity long and erect (Fig. 47A, B). ***Postpetiole*.** Shiny and finely shagreened, dorsum with fading sculpture; in dorsal view sides with moderately short, acute, and triangular projections; pilosity long and erect (Fig. 47A, B). ***Petiole*.** Shiny and finely shagreened; pilosity dense, long, and erect (Fig. 47A, B). ***Colour*.** Reddish brown to brown, lateral sides of mesosoma and legs yellowish brown (Fig. 47A, B).

**Minor workers.** Unknown.

###### Etymology.

Similar to *P. fitarata*, but with more distinct head sculpture in major workers.

###### Biology.

The species was collected between 10–250 m in elevation, in rainforest and tropical dry forest. Nests were located in rotten logs.

###### Comments.

***Major workers*.***Pheidole rugofitarata* sp. nov. is most similar to *P. fitarata* sp. nov. and differs from it in presence of distinct and never-interrupted longitudinal rugae on frons and antennal scrobes, rugae on frons connected with rugae on occipital lobes, never entirely smooth genae, and lobe-like outer hypostomal teeth.

##### Pheidole avaratra sp. nov.

Taxon classificationAnimaliaHymenopteraFormicidae

http://zoobank.org/B4CD74DD-A7C3-427E-80F3-4BE73F6829FE

**Figure 48.**
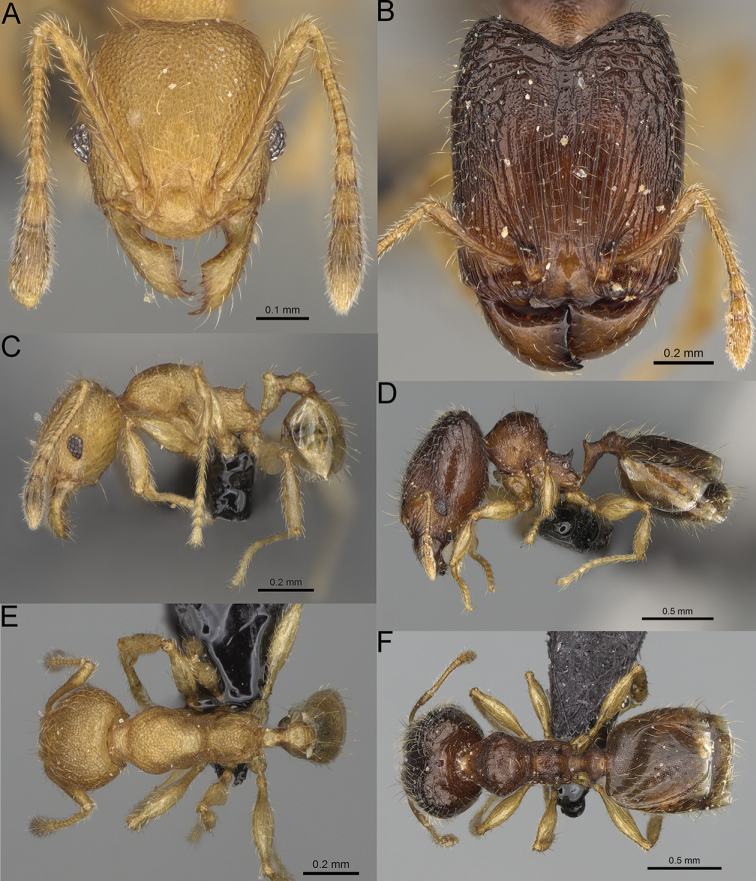
*Pheidole avaratra* sp. nov., full-face view (**A**), profile (**C**), and dorsal view (**E**) of paratype minor worker (CASENT0923184) and full-face view (**B**), profile (**D**), and dorsal view (**F**) of holotype major worker (CASENT0107719).

Figs 48A–F , 84F , 86F

###### Type material.

***Holotype*.** Madagascar. •1 major worker; Antsiranana; Forêt Ambato, 26.6 km 33° Ambanja; -13.4645, 48.55167; alt. 150 m; 10 Dec 2004; Fisher et al. leg.; BLF11593, CASENT0107719 (CASC). ***Paratype*.** Madagascar. •1w.; same data as for holotype; CASENT0923184 (CASC).

###### Other material.

Madagascar. – ***Antsiranana***: •1w., 4s.; Forêt Ambato, 26.6 km 33° Ambanja; -13.4645, 48.55167; alt. 150 m; 8 Dec 2004; Fisher et al. leg.; CASENT0056638, CASENT0056639, CASENT0056704, CASENT0056727, CASENT0107724 (CASC). •1w., 12s.; Nosy Be, Réserve Naturelle Intégrale de Lokobe, 6.3 km 112°ESE Hellville; -13.41933, 48.33117; alt. 30 m; 19 Mar 2001; Fisher et al. leg.; CASENT0403256, CASENT0403258, CASENT0462862, CASENT0463005, CASENT0463136, CASENT0466195, CASENT0466206, CASENT0466207, CASENT0466209, CASENT0466222, CASENT0466243, CASENT0466247, CASENT0466295 (CASC). •2w., 2s.; Nosy Faly; -13.3624, 48.49101; alt. 15 m; 26 Feb 2013; Fisher et al. leg.; CASENT0303115, CASENT0303182 (CASC). •1w., 1s.; Réserve Spéciale d’Ambre, 3.5 km 235°SW Sakaramy; -12.46889, 49.24217; alt. 325 m; 26 Jan 2001; Fisher et al. leg.; CASENT0406637, CASENT0406688 (CASC). •1w., 2s.; Réserve Spéciale de l’Ankarana, 13.6 km 192°SSW Anivorano Nord; -12.86361, 49.22583; alt. 210 m; 16 Feb 2001; Fisher et al. leg.; CASENT0411220, CASENT0440802, CASENT0440818 (CASC).

###### Diagnosis.

***Major workers*.** Head in full-face view elongated; sides of the head with moderately dense, long, erect pilosity; occipital lobes with thick, sparse, short, irregular, and arcuate rugae, interspaces superficially rugulae; inner hypostomal teeth distinct, high, closely spaced, triangular, with rounded apex; outer hypostomal teeth thinner than inner hypostomal teeth and approximately the same height, triangular, but thin. ***Minor workers*.** Head foveolate, genae with smooth notch; scape, when laid back, surpassing the posterior head margin by one-fifth of its length; promesonotum low, convex, short, with posterior declivity smoothly declining towards propodeum; mesosoma foveolate; propodeal spines short, triangular.

###### Description.

**Major workers.** Measurements (*N* = 10): HL: 0.92–1.2 (1.0); HW: 0.71–1.02 (0.8); SL: 0.368–0.502 (0.4); EL: 0.11–0.16 (0.14); WL: 0.71–0.91 (0.99); PSL: 0.12–0.18 (0.13); MTL: 0.41–0.54 (0.44); PNW: 0.4–0.56 (0.45); PTW: 0.1–0.15 (0.12); PPW: 0.25–0.38 (0.3); CI: 78.0–87.9 (82.9); SI: 46.5–51.5 (49.2); PSLI: 11.8–15.2 (13.3); PPI: 35.0–46.2 (38.9); PNI: 51.2–58.9 (55.5); MTI: 52.2 –57.3 (53.8). ***Head*.** In full-face view longitudinal, longer than wide, anterior of eyes straight, posterior of eyes slightly convex, occipital margin of lobes straight, inclining toward centre (Fig. 48B). In lateral view sub-oval; ventral and dorsal faces convex; dorsal face not depressed posteriorly; inner hypostomal teeth visible. Sides of the head with moderately dense, long, erect pilosity; whole head with dense, moderately long, suberect to erect pilosity. Antennal scrobes indistinct and not delimited by carinulae; scrobe surface shiny, with thick, moderately sparse, longitudinal rugae; interspaces superficially rugo-foveolae. Occipital lobes shiny, with thick, sparse, short, irregular, and arcuate rugae, interspaces superficially rugulose; frons with moderately sparse, thick, longitudinal rugae, interspaces smooth and indistinctly rugo-foveolate on the posterior part; genae shiny, with dense and fine rugulae, and smooth notch behind eyes; malar area with thick, sparse, longitudinal rugae, interspaces superficially rugulae. Centre of clypeus shiny and smooth, lateral sides with longitudinal rugae; median notch present, narrow, and moderately deep; median longitudinal carina absent; lateral longitudinal carinae present. Scape, when laid back, not reaching the midlength of head; pilosity suberect to erect (Fig. 48B, D). Inner hypostomal teeth distinct, high, closely spaced, triangular, with rounded apex; outer hypostomal teeth thinner than inner hypostomal teeth and approximately the same height, triangular, but thin (Fig. 84F). ***Mesosoma*.** In lateral view, promesonotum relatively low and arched, dorsal mesonotum slightly concave, posterior mesonotum steep, with tubercle-like projections; promesonotal groove absent; metanotal groove absent; propodeal spines small to moderately long, triangular, with sharp apex and relatively wide base; humeral area laterally weakly produced (Fig. 48D). Surface foveolate, dorsal surface of promesonotum with fading sculpture and sometimes with additional transverse, thin rugulae. Pilosity moderately sparse, very long, and erect (Fig. 48D, F). ***Petiole*.** Shiny; peduncle moderately long, finely foveolate, with indistinct horizontal lobes on its basal part; node with fading sculpture, relatively high, triangular, with rounded apex, in rear view node slightly convex; pilosity moderately sparse and erect (Fig. 48D, F). ***Postpetiole*.** Shiny and finely shagreened, dorsum with fading sculpture; in dorsal view sides with very short, acute, and triangular projections; pilosity moderately long, sparse, and erect (Fig. 48D, F). ***Petiole*.** Shiny and shagreened; pilosity dense, moderately long, and erect (Fig. 48D, F). ***Colour*.** Brown to dark brown; lateral sides of mesosoma, malar area, and lower frons yellowish brown; legs yellow to yellowish brown (Fig. 48D, F).

**Minor workers.** Measurements (*N* = 5): HL: 0.43–0.48 (0.46); HW: 0.39–0.44 (0.42); SL: 0.38–0.47 (0.44); EL: 0.09–0.11 (0.1); WL: 0.5–0.58 (0.55); PSL: 0.08–0.1 (0.93); MTL: 0.29–0.37 (0.35); PNW: 0.27–0.31 (0.29); PTW: 0.05–0.08 (0.07); PPW: 0.1–0.11 (0.11); CI: 87.6–94.8 (90.4); SI: 96.4–110.8 (105.5); PSLI: 17.3–22.0 (20.0); PPI: 46.4–70.3 (61.9); PNI: 67.4–72.8 (69.9); MTI: 74.6–87.6 (83.7). ***Head*.** Occipital margin straight or indistinctly concave; occipital carina absent (Fig. 48A). Pilosity moderately dense, short, suberect. Head shiny, foveolate; genae with smooth notch. Clypeus smooth or finely foveolate; median longitudinal carina absent; two lateral longitudinal carinae absent. Scape, when laid back, surpassing the posterior head margin by one-fifth of its length; pilosity suberect to erect (Fig. 48A, C). ***Mesosoma*.** In lateral view, promesonotum low, convex, short, with posterior declivity smoothly declining towards propodeum; promesonotal groove absent; metanotal groove indistinct; propodeal spines short, triangular, with acute apex (Fig. 48C). Sculpture foveolate. Pilosity sparse, long, and erect (Fig. 48C, E). ***Petiole*.** Peduncle moderately short and thin with ventral face slightly convex; with few short, erect setae (Fig. 48C, E). ***Postpetiole*.** Short, low, and convex; with few short, erect setae (Fig. 48C, E). ***Petiole*.** With moderately sparse, erect pilosity (Fig. 48C, E). ***Colour*.** Yellow (Fig. 48C, E).

###### Etymology.

Malagasy for north, in reference to the distribution of the species.

###### Biology.

The species was collected between 15–210 m in elevation, in rainforest, tropical dry forest, and open secondary vegetation. Nests were located in rotten sticks on ground, under moss, and in soil.

###### Comments.

*Pheidole avaratra* sp. nov. is most similar to *P. fitarata* sp. nov. ***Major workers*.** It differs from *P. fitarata* sp. nov. in presence of longitudinal, very thick, and never interrupted rugae on frons and antennal scrobes, presence of rugae connecting frons and occipital lobes, never entirely smooth genae, and bigger and never directed inward inner hypostomal teeth. ***Minor workers*.***Pheidole avaratra* sp. nov. differs from *P. fitarata* sp. nov. in shorter and higher promesonotum and katepisternum entirely foveolate.

##### Pheidole ehazoara sp. nov.

Taxon classificationAnimaliaHymenopteraFormicidae

http://zoobank.org/D3D0EC8A-6586-4D6A-B972-D9AFDE718683

**Figure 49.**
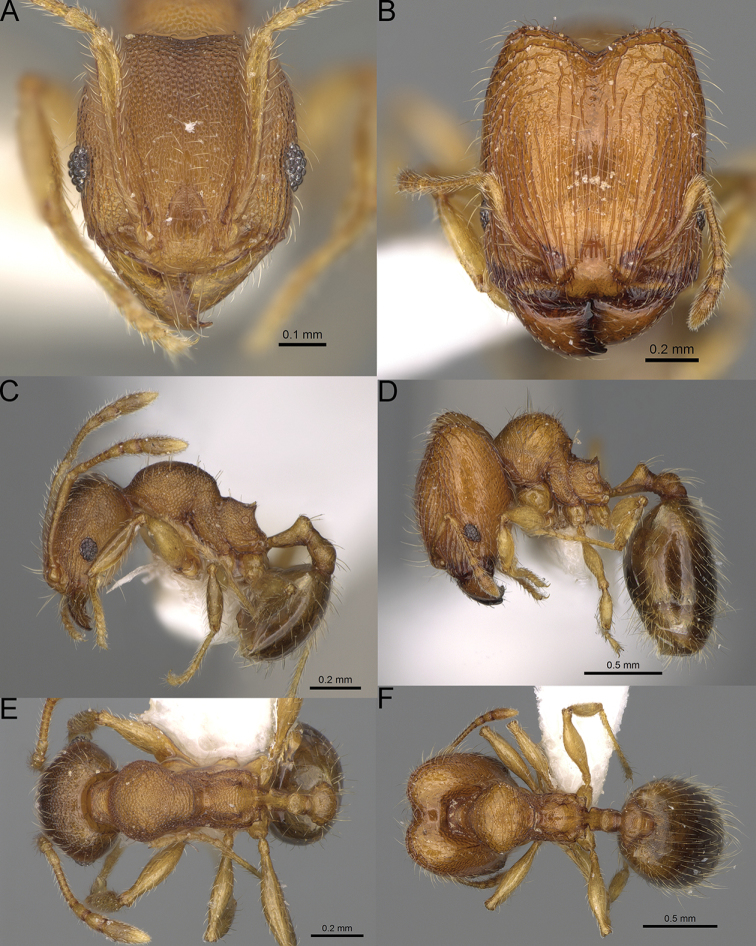
*Pheidole ehazoara* sp. nov., full-face view (**A**), profile (**C**), and dorsal view (**E**) of paratype minor worker (CASENT0923186) and full-face view (**B**), profile (**D**), and dorsal view (**F**) of holotype major worker (CASENT0872059).

Figs 49A–F , 84N , 86N

###### Type material.

***Holotype*.** Madagascar. •1 major worker; Toliara; Ehazoara Canyon, 26 km E Betioky; -23.68333, 44.63333; alt. 175 m; 27 Apr 1997; Fisher et al. leg.; BLF01518, CASENT0872059, middle specimen (CASC). ***Paratype*.** Madagascar. •2w., 2s.; same data as for holotype; CASENT0198555, CASENT0923186, CASENT0872218, CASENT0872219 (CASC).

###### Diagnosis.

***Major workers*.** Head in full-face view elongated; sides of the head with dense, very long, erect pilosity; occipital lobes with thick, sparse, short, irregular, and arcuate rugae, interspaces indistinctly rugulose; inner hypostomal teeth distinct, narrow, and triangular, closely spaced, with narrow and rounded apex directed outward; outer hypostomal teeth bigger and wider than inner hypostomal teeth, dentate. ***Minor workers*.** Head foveolate, foveolate, with additional longitudinal rugae on frons and malar area; genae with fading sculpture; scape, when laid back, reaching head margin; promesonotum low, convex, short, with posterior declivity smoothly declining towards propodeum; mesosoma foveolate; propodeal spines short, triangular.

###### Description.

**Major workers.** Measurements (*N* = 3): HL: 1.07–1.1 (1.08); HW: 0.85–0.88 (0.87); SL: 0.41–0.44 (0.42); EL: 0.14–0.15 (0.14); WL: 0.82–0.86 (0.84); PSL: 0.13–0.14 (0.14); MTL: 0.44–0.46 (0.45); PNW: 0.49–0.51 (0.5); PTW: 0.15–0.16 (0.16); PPW: 0.38–0.42 (0.4); CI: 81.5–83.6 (82.3); SI: 41.8–49.0 (45.9); PSLI: 13.5–15.0 (14.09); PPI: 34.8–37.3 (36.2); PNI: 54.9–55.7 (55.4); MTI: 47.2–52.5 (50.4). ***Head*.** In full-face view longitudinal, longer than wide, anterior of eyes straight, posterior of eyes slightly convex, occipital margin of lobes straight, inclining towards centre (Fig. 49B). In lateral view sub-oval; ventral and dorsal faces convex; dorsal face finely depressed posteriorly, forming indistinct transverse depression between frons and occipital lobes; inner hypostomal teeth visible. Sides of the head with dense, very long, erect pilosity; whole head with dense, moderately long, suberect to erect pilosity. Antennal scrobes indistinct and not delimited by carinulae; scrobe surface shiny, with thick, moderately sparse, longitudinal rugae; interspaces rugulose. Occipital lobes shiny, with thick, sparse, short, irregular, and arcuate rugae, interspaces indistinctly rugulose; frons with moderately sparse, thick, longitudinal rugae, on the anterior part of frons interspaces with fine, short, and longitudinal rugulae, on the posterior part of frons interspaces with fine and irregular rugulae; genae shiny, with fine, dense and longitudinal rugulae, and behind the eyes smooth; malar area with thick, sparse, longitudinal rugae, interspaces smooth to indistinctly rugulose. Centre of clypeus shiny and smooth, lateral sides with longitudinal rugae; median notch indistinct; median longitudinal carina absent; lateral longitudinal carinae absent. Scape, when laid back, not reaching the midlength of head; pilosity erect (Fig. 49B, D). Inner hypostomal teeth distinct, narrow, and triangular, closely spaced, with narrow and rounded apex directed outward; outer hypostomal teeth bigger and wider than inner hypostomal teeth, dentate (Fig. 84N). ***Mesosoma*.** In lateral view, promesonotum relatively low and arched, posterior mesonotum relatively convex, with small, tubercle-like projections; promesonotal groove absent; metanotal groove indistinct; propodeal spines short, triangular, with sharp apex and very wide base; humeral area laterally weakly produced (Fig. 49D). Surface foveolate; promesonotum with additional indistinct and thin, transverse rugulae; its dorsal surface with fading sculpture; propodeum with additional longitudinal rugae, its dorsoventral side with fading sculpture. Pilosity moderately dense, long, suberect to erect (Fig. 49D, F). ***Petiole*.** Shiny; peduncle moderately short, finely foveolate, with distinct, short, horizontal lobes on its basal part; node with fading sculpture, relatively high, triangular, with rounded apex, in rear view node slightly convex; pilosity long and erect (Fig. 49D, F). ***Postpetiole*.** Shiny and finely shagreened, dorsum with fading sculpture; in dorsal view sides with moderately short, very wide, acute, and triangular projections; pilosity long and erect (Fig. 49D, F). ***Petiole*.** Shiny and finely shagreened on the basal part of first gastral tergite; pilosity dense, long, and erect (Fig. 49D, F). ***Colour*.** Dark yellow; lower part of malar area brown; gaster yellowish brown to brown (Fig. 49D, F).

**Minor workers.** Measurements (*N* = 2): HL: 0.52–0.51; HW: 0.47–0.46; SL: 0.42–0.4; EL: 0.12–0.1; WL: 0.6–0.59; PSL: 0.1–0.09; MTL: 0.37–0.34; PNW: 0.33–0.32; PTW: 0.09–0.09; PPW: 0.17–0.15; CI: 83.6–81.5; SI: 41.8–49.0; PSLI: 15.0–13.9; PPI: 36.3–34.8; PNI: 54.9–55.7; MTI: 47.2–52.5. ***Head*.** Occipital margin straight or indistinctly concave; occipital carina indistinct (Fig. 49A). Pilosity moderately dense, short, suberect. Head shiny, foveolate, with additional longitudinal rugae on frons and malar area; genae with fading sculpture. Clypeus with fine, short, and longitudinal rugulae; median longitudinal carina absent; two lateral longitudinal carinae absent. Scape, when laid back, reaching head margin; pilosity suberect to erect (Fig. 49A, C). ***Mesosoma*.** In lateral view, promesonotum low, convex, short, with posterior declivity smoothly declining towards propodeum; promesonotal groove absent; metanotal groove present; propodeal spines small, triangular, with acute apex (Fig. 49C). Sculpture foveolate. Pilosity moderately sparse, moderately short, and erect (Fig. 49C, E). ***Petiole*.** Shiny; peduncle finely foveolate; short and thin; node moderately low, globular, and small; with few short, erect setae (Fig. 49C, E). ***Postpetiole*.** Moderately long, low, and slightly convex; with few short, erect setae (Fig. 49C, E). ***Petiole*.** With moderately sparse, erect pilosity (Fig. 49C, E). ***Colour*.** Dark yellow (Fig. 49C, E).

###### Etymology.

After the locus typicus.

###### Biology.

The species was collected at 175 m in elevation, in tropical dry rainforest. Nest was located under a stone.

###### Comments.

*Pheidole ehazoara* sp. nov. is most similar to *P. fitarata* sp. nov. ***Major workers*.** It differs from *P. fitarata* sp. nov. in dark yellow body colouration, frons with rugae connected with rugae on the occipital lobes, absence of foveolae on head, shorter propodeal spines with wider base, and dentate outer hypostomal teeth directed outward. ***Minor workers*.** It differs from *P. fitarata* sp. nov. in presence of the additional longitudinal rugae on frons and malar area, longer postpetiole, and never smooth genae.

#### Revision of the *Pheidole curvistriata* group

**Diagnosis. *Major workers*.** Head in full face view rectangular, slightly widening posteriorly, in lateral view sub-rectangular with ventral and dorsal faces finely convex, dorsal face finely depressed posteriorly; antennal scrobes shallowly impressed; occipital lobes with thick, sparse, irregular rugae, interspaces smooth to finely rugulose; frons with thin to thick, sparse to dense, longitudinal rugae, interspaces smooth to indistinctly rugulose; lateral sides of head with fine, irregular rugoreticulation or dense and thin longitudinal rugulae, interspaces finely foveolate; promesonotum short, angular, and relatively low; promesonotal groove absent; metanotal groove absent or indistinct; propodeal spines moderately long, narrow, with base slightly wider than top; mesosoma finely rugoreticulate or foveolate, katepisternum and at least lower anepisternum smooth, lateral sides of propodeum with few thick, longitudinal rugae; first gastral tergite finely shagreened; body brown to dark brown. ***Minor workers*.** Head foveolate, with thick, sparse, and longitudinal rugae on frons and sparse, irregular to arcuate, thick rugae on vertex; scape, when laid back, surpassing the posterior head margin by two-fifths of its length; promesonotum low, short, flat, or slightly convex, with steep posterior declivity; promesonotal groove absent; metanotal groove absent or indistinct; propodeal spines short or moderately long, triangular; mesosoma foveolate or foveolate with additional thick and irregular rugae, sometimes anepisternum and katepisternum smooth; body yellow to brown.

**Comments.** Major workers of this group can be distinguished based on a combination of the following characters: head in full face view rectangular, slightly widening posteriorly, in lateral view sub-rectangular; occipital lobes with thick, sparse, irregular rugae, interspaces smooth to finely rugulose; moderately long and narrow propodeal spines; finely rugoreticulate or foveolate mesosoma with smooth katepisternum and at least lower part of anepisternum; finely shagreened first gastral tergite, and brown to dark brown body. Minor workers can be easily distinguished based on foveolate head with additional rugae on frons and irregular to arcuate rugae on vertex; foveolate mesosoma usually with additional irregular rugae and yellow to brown body.

Major workers of this group are extremely similar and sometimes the key features overlap. Therefore, we recommend using minor workers, which possess more distinct and stable morphological characters, for species determination within this group.

This group contains four species: *P. curvistriata* sp. nov., *P. makirovana* sp. nov., *P. mantadia* sp. nov., and *P. moramanaensis* sp. nov. All members of this group are sympatric and, except *P. mantadia* sp. nov. known only from its type locality (Corridor Forestier Analamay-Mantadia, Toamasina), are distributed across central highlands and evergreen forest.

##### Key to the *Pheidole curvistriata* group

**Table:** 

1	Major workers. Frons with sparse, thick, longitudinal rugae with smooth to indistinctly rugulose interspaces; mesosoma with rugulae and sometimes indistinct foveolae, outer hypostomal teeth higher than inner hypostomal teeth but very narrow (Fig. 50A, I, E). Minor workers. Rugae on upper part of frons distinct and never curved outward, mesonotum never smooth (Fig. 51A, E, I)	***P. curvistriata* sp. nov.**
–	Major workers. Frons with longitudinal rugae denser, with interspaces smooth to distinctly rugo-foveolate or foveolate; mesosoma foveolate and sometimes with indistinct rugulae, outer hypostomal teeth higher than inner hypostomal teeth but wide (Fig. 50B–D, F–H, J–L). Minor workers. Rugae on upper part of frons indistinct or distinct and curved outward, mesonotum smooth (Fig. 51B–D, F–H, J–L)	**2**
2	Major workers. Frons and mesosoma with interspaces finely rugo-foveolate (Fig. 50C, K). Minor workers. Rugae on upper part of frons curved outward, mesosoma always with thick rugae, propodeal spines longer (Fig. 51C, G, K)	***P. makirovana* sp. nov.**
–	Major workers. Frons and mesosoma with fine foveolae (Fig. 50B, D, J, L). Minor workers. Rugae on upper part of frons never curved outward, propodeal spines shorter, mesosoma never with thick rugae (Fig. 51B, D, F, H, J, L)	**3**
3	Major workers. Sides of head with sparse and short pilosity, inner hypostomal teeth very low (Fig. 50D, H). Minor workers. Clypeus foveolate to rugoreticulate, rugae on head very indistinct (Fig. 51D)	***P. mantadia* sp. nov.**
–	Major workers. Sides of head with dense and long pilosity, inner hypostomal teeth higher (Fig. 50B, F). Minor workers. Clypeus smooth, rugae on head distinct but thin (Fig. 51B)	***P. moramanaensis* sp. nov.**

**Figure 50.**
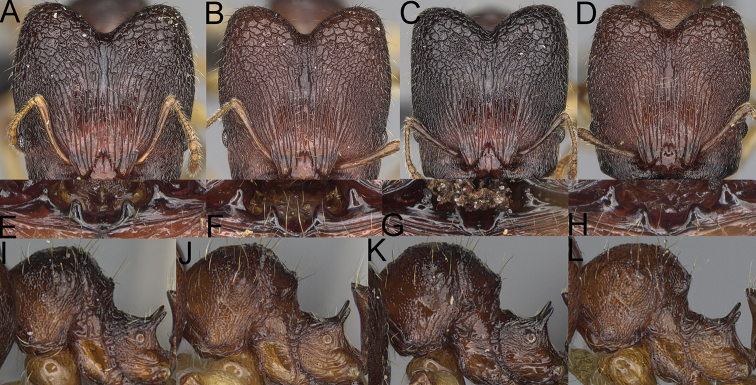
Major workers. *Pheidole curvistriata* sp. nov., head (**A**), profile (**I**), hypostomal teeth (**E**). *Pheidole moramanaensis* sp. nov., head (**B**), profile (**J**), hypostomal teeth (**F**). *Pheidole makirovana* sp. nov., head (**C**), profile (**K**), hypostomal teeth (**G**). *Pheidole mantadia* sp. nov., head (**D**), profile (**L**), hypostomal teeth (**H**).

**Figure 51.**
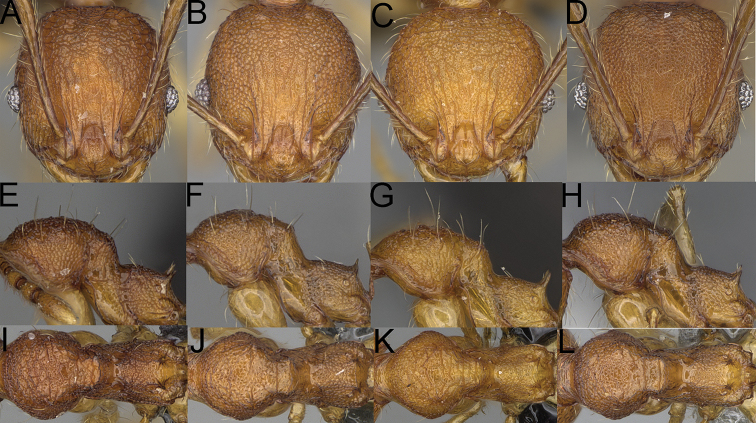
Minor workers. *Pheidole curvistriata* sp. nov., head (**A**), profile (**E**), dorsal view of mesosoma (**I**). *Pheidole moramanaensis* sp. nov., head (**B**), profile (**F**), dorsal view of mesosoma (**J**). *Pheidole makirovana* sp. nov., head (**C**), profile (**G**), dorsal view of mesosoma (**K**). *Pheidole mantadia* sp. nov., head (**D**), profile (**H**), dorsal view of mesosoma (**L**).

##### Pheidole curvistriata sp. nov.

Taxon classificationAnimaliaHymenopteraFormicidae

http://zoobank.org/49EB4EAC-54F3-4B53-A530-604181FFD4C2

**Figure 52.**
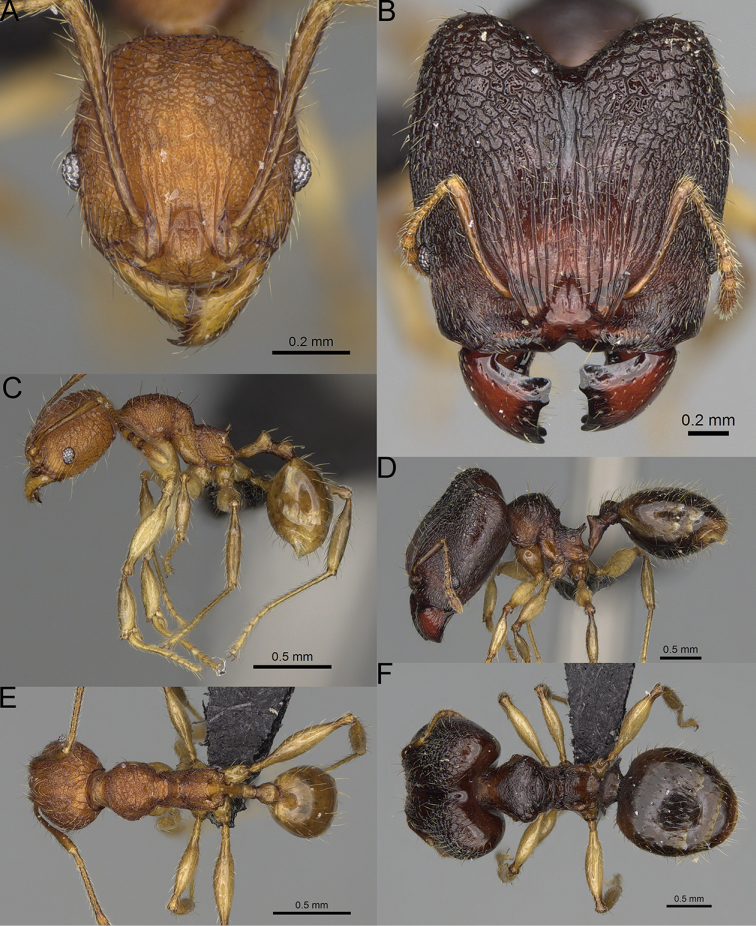
*Pheidole curvistriata* sp. nov., full-face view (**A**), profile (**C**), and dorsal view (**E**) of paratype minor worker (CASENT0492901) and full-face view (**B**), profile (**D**), and dorsal view (**F**) of holotype major worker (CASENT0492904).

Figs 52A–F , 84L , 86L

###### Type material.

***Holotype*.** Madagascar. •1 major worker; Fianarantsoa; Parc National de Ranomafana, Sahamalaotra River, 6.6 km 310°NW Ranomafana; -21.23667, 47.39667; alt. 1150 m; 31 Mar 2003; B.L. Fisher et al. leg.; BLF08623, CASENT0492904, top specimen (CASC). ***Paratypes*.** Madagascar. •9 w., 8s.; same data as for holotype; CASENT0492900–CASENT0492903, CASENT0872240, CASENT0492905, CASENT0872174–CASENT0872184 (CASC).

###### Other material.

Madagascar. –***Antsiranana***: •2w., 2s., 1m.; Sava Region: Parc National de Marojejy, Manantenina River, 28.0 km 24.8°NE Andapa; -14.43461, 49.76074; alt. 780 m; 13 Feb 2018; B.L. Fisher et al. leg.; CASENT0807397–CASENT0807399 (CASC). –***Fianarantsoa***: •8w., 6s.; 2 km W Andrambovato, along river Tatamaly; -21.51167, 47.41; alt. 1075 m; 3 Jun 2005; B.L. Fisher et al. leg.; CASENT0060845, CASENT0060847, CASENT0060859, CASENT0060861, CASENT0060936, CASENT0060937, CASENT0060940, CASENT0060941 (CASC). •2w., 2s.; Ambinanindranomena Non-Protected Area, 39.45 km SE Ambalavao; -21.95386, 47.29427; alt. 1069 m; 1 Feb 2012; Andrianjaka & Ravelomanana leg.; CASENT0293938, CASENT0293939 (CASC). •1w.; Parc National de Ranomafana, Sahamalaotra River, 6.6 km 310°NW Ranomafana; -21.23667, 47.39667; alt. 1150 m; 31 Mar 2003; B.L. Fisher et al. leg.; CASENT0492823 (CASC). •12w., 10s.; Parc National de Ranomafana, Vatoharanana River, 4.1 km 231°SW Ranomafana; -21.29, 47.43333; alt. 1100 m; 27 Mar 2003; B.L. Fisher et al. leg.; CASENT0040349, CASENT0497398, CASENT0497453, CASENT0497454, CASENT0497614, CASENT0497616, CASENT0497627, CASENT0497629 (CASC). –***Toamasina***: •3w., 1m.; Ankerana; -18.40829, 48.82107; alt. 750 m; 23 Jan 2012; B.L. Fisher et al. leg.; CASENT0274813, CASENT0274814 (CASC). •2w., 2s.; Corridor Forestier Analamay-Mantadia, Ambatoharanana; -18.80388, 48.40506; alt. 1013 m; 12 Dec 2012; B.L. Fisher et al. leg.; CASENT0300845, CASENT0301767 (CASC). •4w., 3s., 1q.; Corridor Forestier Analamay-Mantadia, Tsaravoniana; -18.76124, 48.42134; alt. 939 m; 3 Dec 2012; B.L. Fisher et al. leg.; CASENT0297031, CASENT0297032, CASENT0297063, CASENT0297075 (CASC). •2s.; F.C. Andriantantely; -18.695, 48.81333; alt. 530 m; 4 Dec 1998; Ratsirarson leg.; CASENT0198897 (CASC). –***Toliara***: •1s.; Parc National d’Andohahela, Col du Sedro, 3.8 km 113°ESE Mahamavo, 37.6 km 341°NNW Tolagnaro; -24.76389, 46.75167; alt. 900 m; 21 Jan 2002; B.L. Fisher et al. leg.; CASENT0479331 (CASC).

###### Diagnosis.

***Major workers*.** Head in full face view rectangular, slightly widening posteriorly; sides of the head with dense, relatively long, erect pilosity; frons with fine, irregularly rugoreticulate, interspaces finely foveolate; inner hypostomal teeth distinct, moderately high, thick and triangular, with rounded apex; outer hypostomal teeth thinner but higher, with rounded tips, and wide base; inner and outer teeth closely spaced and connected by indistinct concavity; propodeal spines moderately long, narrow, with base slightly wider than top; first gastral tergite finely shagreened. ***Minor workers*.** Whole head foveolate, thick, sparse and longitudinal rugae overlie foveolate sculpture on frons and malar area; sparse, irregular to arcuate, thick rugae overlie foveolate sculpture on vertex; promesonotum low, short, flat or slightly convex, with steep posterior declivity; mesosoma foveolate, with sparse, thick, and irregular rugae overlying foveolae, katepisternum smooth.

###### Description.

**Major workers.** Measurements (*N* = 10): HL: 1.62–1.73 (1.67); HW: 1.49–1.64 (1.57); SL: 0.66–0.73 (0.69); EL: 0.17–0.2 (0.18); WL: 1.15–1.26 (1.18); PSL: 0.23–0.27 (0.25); MTL: 0.68–0.73 (0.7); PNW: 0.67–0.79 (0.73); PTW: 0.19–0.22 (0.21); PPW: 0.61–0.7 (0.65); CI: 91.6–95.7 (94.1); SI: 41.1–45.7 (43.8); PSLI: 13.8–15.8 (14.9); PPI: 29.1–34.7 (31.8); PNI: 44.7–48.7 (46.3); MTI: 42.7–45.7 (44.6). ***Head*.** In full-face view rectangular, slightly widening posteriorly, anterior of eyes relatively straight, posterior of eyes convex (Fig. 52B). In lateral view sub-rectangular; ventral and dorsal faces finely convex; dorsal face finely depressed posteriorly, forming indistinct transverse depression between frons and occipital lobes; inner hypostomal teeth invisible. Sides of the head with dense, relatively long, erect pilosity; whole head with dense, moderately long, suberect to erect pilosity. Antennal scrobes indistinct and not delimited by carinulae. Occipital lobes shiny, with thick, sparse, irregular rugae, interspaces superficially rugulae; frons with thick, sparse, longitudinal rugae, interspaces smooth to indistinctly rugulose; lateral sides of head with fine, irregularly rugoreticulate, interspaces finely foveolate; malar area with dense and thin longitudinal or irregular rugulae; genae with fine, thin, moderately dense to dense rugulae. Clypeus shiny and smooth, with thin, longitudinal rugulae on lateral sides; median notch present, narrow and moderately deep; median longitudinal carina absent; lateral longitudinal carinae absent. Scape, when laid back, reaching midlength of head; pilosity decumbent to erect (Fig. 52B, D). Inner hypostomal teeth distinct, moderately high, thick, and triangular, with rounded apex; outer hypostomal teeth thinner but higher, with rounded tips, and wide base; inner and outer teeth closely spaced and connected by indistinct concavity (Fig. 84L). ***Mesosoma*.** In lateral view, promesonotum short, angular, and relatively low, posterior mesonotum steep, with small tubercle-like projections; promesonotal groove absent; metanotal groove absent or indistinct; propodeal spines moderately long, narrow, with base slightly wider than top, apex rounded; humeral area with small and flat tubercles (Fig. 52D). Surface shiny, finely rugoreticulate, katepisternum and lower anepisternum smooth, lateral sides of propodeum with few thick, longitudinal rugae. Pilosity moderately sparse, long, and erect (Fig. 52D, F). ***Petiole*.** Shiny, finely foveolate; peduncle short, with small, rounded, horizontal lobes on its basal part; node moderately high and narrow, with convex apex, in rear view node relatively straight; pilosity moderately dense and erect (Fig. 52D, F). ***Postpetiole*.** Shiny, with fine and sparse foveolae; short and rounded; in dorsal view sides with relatively long, triangular projections; pilosity long, moderately dense, and erect (Fig. 52D, F). ***Petiole*.** First gastral tergite shiny and finely shagreened; pilosity dense, moderately long, and erect (Fig. 52D, F). ***Colour*.** Unicolourous, brown; legs dark yellow (Fig. 52D, F).

**Minor workers.** Measurements (*N* = 10): HL: 0.62–0.68 (0.66); HW: 0.53–0.63 (0.6); SL: 0.6–0.67 (0.64); EL: 0.1–0.13 (0.12); WL: 0.74–0.84 (0. 8); PSL: 0.12–0.13 (0.12); MTL: 0.49–0.55 (0.52); PNW: 0.38–0.44 (0.42); PTW: 0.07–0.09 (0.08); PPW: 0.13–0.19 (0.15); CI: 86.1–94.5 (90.6); SI: 105.5–112.8 (108.6); PSLI: 17.8–19.2 (18.6); PPI: 47.8–56.5 (52.2); PNI: 68.9–72.7 (70.8); MTI: 85.6–91.9 (87.6). ***Head*.** Occipital margin straight or indistinctly concave; occipital carina indistinct, weakly developed (Fig. 52A). Pilosity moderately dense, long, erect. Whole head foveolate, thick, sparse and longitudinal rugae overlie foveolate sculpture on frons and malar area; sparse, irregular to arcuate, thick rugae overlie foveolate sculpture on vertex. Clypeus shiny, foveolate to rugae; median longitudinal carina present; two lateral longitudinal carinae absent. Scape, when laid back, surpassing the posterior head margin by two-fifths of its length; pilosity erect (Fig. 52A, C). ***Mesosoma*.** In lateral view, promesonotum low, short, flat or slightly convex, with steep posterior declivity; promesonotal groove absent; metanotal groove absent or indistinct; propodeal spines short, triangular, apex acute (Fig. 52C). Sculpture foveolate, with sparse, thick, and irregular rugae overlying foveolae, katepisternum smooth. Pilosity moderately sparse, long, and erect (Fig. 52C, E). ***Petiole*.** Peduncle moderately short and thin; with few long, erect setae (Fig. 52C, E). ***Postpetiole*.** Short, low, and slightly convex; with few short, erect setae (Fig. 52C, E). ***Petiole*.** With sparse, erect pilosity (Fig. 52C, E). ***Colour*.** Unicolourous, yellowish brown to brown (Fig. 52C, E).

###### Etymology.

Latin for arcuate rugae, in reference to head sculpture in minor workers.

###### Biology.

The species was collected between 530–1150 m in elevation, in rainforest and montane rainforest. Nests were located in rotten logs, twigs, and stumps.

###### Comments.

*Pheidole curvistriata* sp. nov. is most similar to *P. mantadia* sp. nov. and *P. moramanaensis* sp. nov. ***Major workers*.***Pheidole curvistriata* sp. nov. can be distinguished from *P. mantadia* sp. nov. and *P. moramanaensis* sp. nov. by absence of fine foveolae sculpture on frons, moderately high and thick inner hypostomal teeth, which are higher than wide, and thin, high, outer hypostomal teeth. ***Minor workers*.***Pheidole curvistriata* sp. nov. can be distinguished from *P. mantadia* sp. nov. and *P. moramanaensis* sp. nov. by frons and malar area with distinct, longitudinal rugae, vertex with indistinct irregular to arcuate rugae, presence of irregular rugae on mesosoma, and never smooth mesonotum.

##### Pheidole moramanaensis sp. nov.

Taxon classificationAnimaliaHymenopteraFormicidae

http://zoobank.org/B9BFD481-97AC-43D0-BABA-9C3BD42C67E6

**Figure 53.**
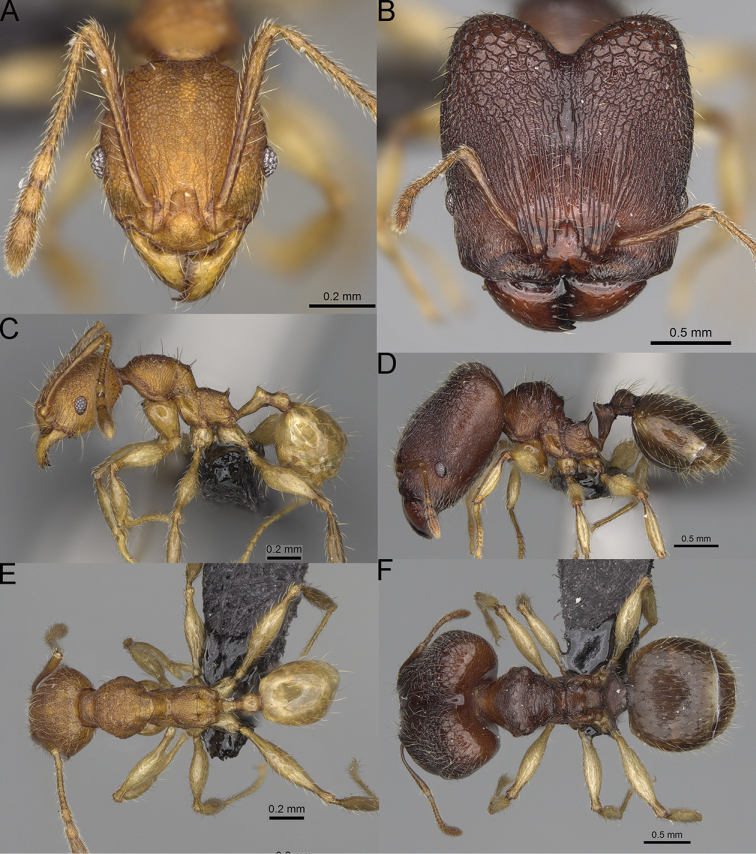
*Pheidole moramanaensis* sp. nov., full-face view (**A**), profile (**C**), and dorsal view (**E**) of paratype minor worker (CASENT0923172) and full-face view (**B**), profile (**D**), and dorsal view (**F**) of holotype major worker (CASENT0050486).

Figs 53A–F , 85L , 87P

###### Type material.

***Holotype*.** Madagascar. •1 major worker; Toamasina; Forêt Ambatovy, 14.3 km 57° Moramanga; -18.85083, 48.32; alt. 1075 m; 23 Mar 2004; B.L. Fisher et al. leg.; BLF10624, CASENT0050486 (CASC). ***Paratypes*.** Madagascar. •2w., 2s.; same data as for holotype; CASENT0923172, CASENT0051295, CASENT0051296, CASENT0872186 (CASC).

###### Other material.

Madagascar. –***Fianarantsoa***: •2w., 1s.; 9.0 km NE Ivohibe; -22.42667, 46.93833; alt. 900 m; 12 Nov 1997; B.L. Fisher et al. leg.; CASENT0198400, CASENT0198899 (CASC). –***Mahajanga***: •2w., 1s., 1q.; Réserve Spéciale Marotandrano, Marotandrano 48.3 km S Mandritsara; -16.28322, 48.81443; alt. 865 m; 7 Dec 2007; B.L. Fisher et al. leg.; CASENT0134236, CASENT0134237 (CASC). –***Toamasina***: •1w., 1q.; Bevolota 17.1 km N Andasibe; -18.77071, 48.43164; alt. 995 m; 12 Dec 2007; B.L. Fisher et al. leg.; CASENT0135181 (CASC). •3w., 2s., 1q.; Corridor Forestier Analamay-Mantadia, Ambatoharanana; -18.80388, 48.40506; alt. 1013 m; 12 Dec 2012; B.L. Fisher et al. leg.; CASENT0300854, CASENT0300855, CASENT0301765 (CASC). •1w., 1s.; Corridor Forestier Analamay-Mantadia, Ambatoharanana; -18.80424, 48.40081; alt. 968 m; 12 Dec 2012; B.L. Fisher et al. leg.; CASENT0301827 (CASC). •1w., 1s.; Corridor Forestier Analamay-Mantadia, Tsaravoniana; -18.76124, 48.42134; alt. 939 m; 2 Dec 2012; B.L. Fisher et al. leg.; CASENT0297007 (CASC). •2w., 1s., 1q.; Forêt Ambatovy, 14.3 km 57° Moramanga; -18.85083, 48.32; alt. 1075 m; 18 Dec 2004; B.L. Fisher et al. leg.; CASENT0110600, CASENT0110601 (CASC).

###### Diagnosis.

***Major workers*.** Head in full face view rectangular, slightly widening posteriorly; sides of the head with moderately dense, long, erect pilosity; frons with thin, moderately dense, longitudinal rugae, interspaces smooth or with indistinct but dense rugulae; low, thick and triangular, with rounded apex; outer hypostomal teeth higher than inner hypostomal teeth, thick, with rounded tips, triangular inner and outer teeth closely spaced and connected by indistinct concavity; propodeal spines moderately long, with base slightly wider than top; first gastral tergite finely shagreened. ***Minor workers*.** Whole head foveolate, fading on frons and genae; thin, sparse, longitudinal to irregular rugae overlie foveolate sculpture; promesonotum low, short, flat, or slightly convex, with steep posterior declivity; mesosoma foveolate, katepisternum and mesonotum smooth.

###### Description.

**Major workers.** Measurements (*N* = 9): HL: 1.61–1.79 (1.69); HW: 1.49–1.68 (1.57); SL: 0.62–0. 7 (0.67); EL: 0.17–0.2 (0.19); WL: 1.15–1.26 (1.2); PSL: 0.23–0.27 (0.26); MTL: 0.62–0.74 (0.68); PNW: 0.71–0.8 (0.75); PTW: 0.2–0.25 (0.22); PPW: 0.58–0.77 (0.7); CI: 91.5–95.2 (93.5); SI: 40.1–45.6 (42.7); PSLI: 14.0–16.2 (15.4); PPI: 28.4–34.0 (31.0); PNI: 45.7–49.5 (47.9); MTI: 41.7–45.1 (43.4). ***Head*.** In full-face view rectangular, slightly widening posteriorly, anterior of eyes relatively straight, posterior of eyes convex (Fig. 53B). In lateral view sub-rectangular; ventral and dorsal faces finely convex; dorsal face finely depressed posteriorly, forming shallow transverse depression between frons and occipital lobes; inner hypostomal teeth invisible. Sides of head with moderately dense, long, erect pilosity; whole head with moderately long, dense, suberect to erect pilosity. Antennal scrobes indistinct and not delimited by carinulae. Occipital lobes shiny, with thick, sparse, irregular rugae, interspaces smooth to finely rugulose; frons with thin, moderately dense, longitudinal rugae, interspaces smooth or with indistinct but dense rugulae; lateral sides of head with thin, dense, longitudinal rugulae, area between rugulae with sparse foveolae; malar area with dense and thin longitudinal rugulae; genae with fine, thin, dense rugulae. Clypeus shiny and smooth, with thin, longitudinal rugulae on the lateral sides; median notch present, wide, and shallow; median longitudinal carina absent; lateral longitudinal carinae absent. Scape, when laid back, reaching the midlength of head; pilosity decumbent to erect (Fig. 53B, D). Inner hypostomal teeth distinct, low, thick and triangular, with rounded apex; outer hypostomal teeth higher than inner hypostomal teeth, thick, with rounded tips, triangular; inner and outer teeth closely spaced and connected by indistinct concavity (Fig. 85L). ***Mesosoma*.** In lateral view, promesonotum short, angular, and relatively low, posterior mesonotum steep, with small, tubercle-like projections; promesonotal groove absent; metanotal groove absent; propodeal spines moderately long, thin, with base slightly wider than top, apex rounded; humeral area with small and flat tubercles (Fig. 53D). Surface shiny, with fine and sparse foveolae; mesonotum and katepisternum smooth, lateral sides of propodeum with few thick, longitudinal rugae; surface with additional indistinct and sparse rugulae on promesonotum. Pilosity moderately sparse, long, and erect (Fig. 53D, F). ***Petiole*.** Shiny, finely shagreened; peduncle short, with small, rounded, horizontal lobes on its basal part; node moderately high and narrow, with convex apex, in rear view node straight; pilosity moderately sparse and erect (Fig. 53D, F). ***Postpetiole*.** Shiny and finely shagreened; short and rounded; in dorsal view sides with relatively long, acute, horn-like projections; pilosity long, moderately dense, and erect (Fig. 53D, F). ***Petiole*.** First gastral tergite shiny and finely shagreened; pilosity moderately dense, long, and erect (Fig. 53D, F). ***Colour*.** Unicolourous, brown to dark brown; legs yellow (Fig. 53D, F).

**Minor workers.** Measurements (*N* = 10): HL: 0.55–0.63 (0.59); HW: 0.51–0.57 (0.53); SL: 0.57–0.62 (0.59); EL: 0.1–0.13 (0.11); WL: 0.67–0.79 (0.74); PSL: 0.1–0.15 (0.11); MTL: 0.43–0.49 (0.46); PNW: 0.36–0.4 (0.38); PTW: 0.06–0.1 (0.08); PPW: 0.11–0.17 (0.08); CI: 87.2–93.8 (90.2); SI: 107.8–114.0 (111.4); PSLI: 16.2–23.5 (19.3); PPI: 52.1–61.8 (56.1); PNI: 68.8–71.8 (70.6); MTI: 83.0–93.3 (87.4). ***Head*.** Occipital margin straight or indistinctly concave; occipital carina indistinct, weakly developed (Fig. 53A). Pilosity moderately dense, long, erect. Whole head foveolate, fading on frons and genae; thin, sparse, longitudinal to irregular rugae overlie foveolate sculpture. Clypeus with median longitudinal carina present; two lateral longitudinal carinae absent. Scape, when laid back, surpassing the posterior head margin by two-fifths of its length; pilosity erect (Fig. 53A, C). ***Mesosoma*.** In lateral view, promesonotum low, short, flat or slightly convex, with steep posterior declivity; promesonotal groove absent; metanotal groove absent or indistinct; propodeal spines very short, triangular, apex acute (Fig. 53C). Sculpture foveolate; katepisternum and mesosoma smooth. Pilosity sparse, long, and erect (Fig. 53C, E). ***Petiole*.** Peduncle moderately short and thin; node low, triangular, and small; with few long, erect setae (Fig. 53C, E). ***Postpetiole*.** Short, low, and slightly convex; with few short, erect setae (Fig. 53C, E). ***Petiole*.** With sparse, erect pilosity (Fig. 53C, E). ***Colour*.** Unicolourous, yellow to yellowish brown (Fig. 53C, E).

###### Etymology.

From the type locality.

###### Biology.

The species was collected between 865–1075 m in elevation, in transition humid forest, rainforest, and montane rainforest. Nests were located in rotten logs, rotted twigs on the ground, and dead bamboo above the ground.

###### Comments.

*Pheidole moramanaensis* sp. nov. is most similar to *P. curvistriata* sp. nov. and *P. mantadia* sp. nov. ***Major workers*.***Pheidole moramanaensis* sp. nov. can be distinguished from *P. curvistriata* sp. nov. by presence of fine foveolate sculpture on frons and mesosoma, low and thick inner hypostomal teeth which are wider than high, and thick outer hypostomal teeth; form *P. mantadia* sp. nov. by denser and longer pilosity on head sides, and higher and narrower outer hypostomal teeth. ***Minor workers*.***Pheidole moramanaensis* sp. nov. can be distinguished from *P. curvistriata* sp. nov. by frons and malar area with thin, longitudinal to irregular rugae, and vertex with thin irregular and never arcuate rugae, absence of irregular rugae on mesosoma, and smooth mesonotum; from *P. mantadia* sp. nov. by frons and malar area with thin but distinct, irregular rugae and smooth clypeus.

##### Pheidole makirovana sp. nov.

Taxon classificationAnimaliaHymenopteraFormicidae

http://zoobank.org/C9CB164B-0A45-49AE-99AB-9AB732539425

**Figure 54.**
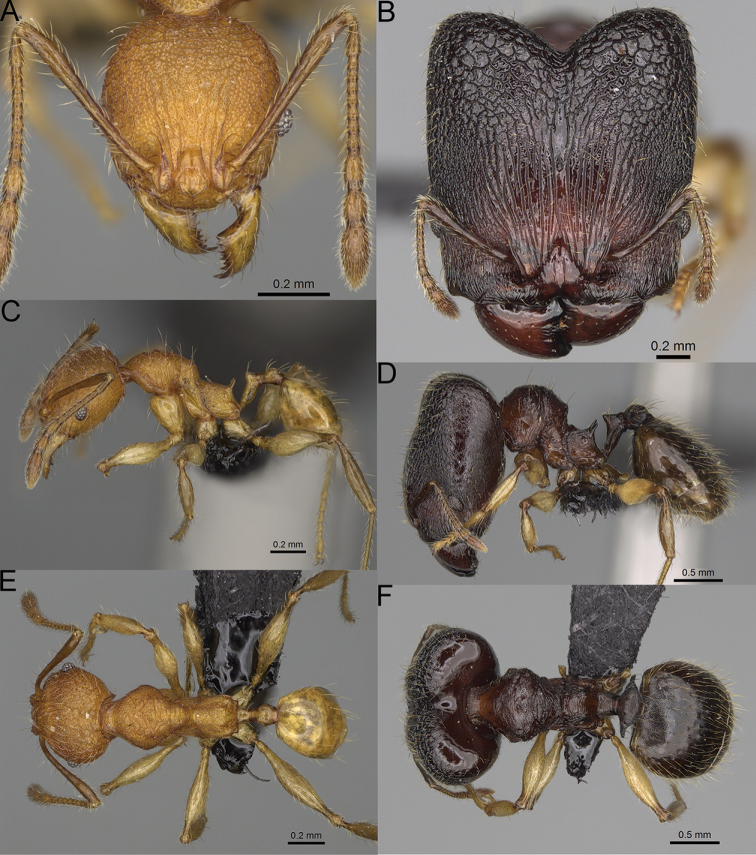
*Pheidole makirovana* sp. nov., full-face view (**A**), profile (**C**), and dorsal view (**E**) of paratype minor worker (CASENT0923174) and full-face view (**B**), profile (**D**), and dorsal view (**F**) of holotype major worker (CASENT0231059).

Figs 54A–F , 85E , 87I

###### Type material.

***Holotype*.** Madagascar. •1 major worker; Antsiranana; Makirovana Forest; -14.16506, 49.9477; alt. 900 m; 30 Apr 2011; B.L. Fisher et al. leg.; BLF26745, CASENT0231059 (CASC). ***Paratype*.** Madagascar. •1w.; same data as for holotype; CASENT0923174 (CASC).

###### Other material.

Madagascar. –***Antsiranana***: •2w., 1s., 2q.; 6.5 km SSW Befingotra, Rés. Anjanaharibe-Sud; -14.75, 49.5; alt. 875 m; 23 Oct 1994; B.L. Fisher leg.; CASENT0198415, CASENT0198572, CASENT0235044, CASENT0235045 (CASC). •8w., 4s., 1q.; 9.2 km WSW Befingotra, Rés. Anjanaharibe-Sud; -14.75, 49.46667; alt. 1200 m; 11 Nov 1994; B.L. Fisher leg.; CASENT0198570, CASENT0198571, CASENT0198574, CASENT0235107 (CASC). •2w., 2q.; Makirovana Forest; -14.16506, 49.9477; alt. 900 m; 30 Apr 2011; B.L. Fisher et al. leg.; CASENT0231036, CASENT0231037, CASENT0923175 (CASC). •6w., 3s.; Parc National de Marojejy, Manantenina River, 27.6 km 35°NE Andapa, 9.6 km 327°NNW Manantenina; -14.435, 49.76; alt. 775 m; 15 Nov 2003; B.L. Fisher et al. leg.; CASENT0495015, CASENT0495016, CASENT0495019 (CASC). •3w.; Parc National de Marojejy, Manantenina River, 28.0 km 38°NE Andapa, 8.2 km 333°NNW Manantenina; -14.43667, 49.775; alt. 450 m; 12 Nov 2003; B.L. Fisher et al. leg.; CASENT0077212 (CASC). –***Mahajanga***: •1w., 1s.; Réserve Spéciale Marotandrano, Marotandrano 48.3 km S Mandritsara; -16.28322, 48.81443; alt. 865 m; 7 Dec 2007; B.L. Fisher et al. leg.; CASENT0134300 (CASC). –***Toamasina***: •2w., 2s.; F.C. Didy; -18.19833, 48.57833; alt. 960 m; 16 Dec 1998; Ratsirarson leg.; CASENT0198558, CASENT0198898 (CASC). •5w., 2s.; Montagne d’Akirindro 7.6 km 341°NNW Ambinanitelo; -15.28833, 49.54833; alt. 600 m; 17 Mar 2003; B.L. Fisher et al. leg.; CASENT0038919, CASENT0496319, CASENT0496320 (CASC). •2s.; Montagne d’Anjanaharibe, 19.5 km 27°NNE Ambinanitelo; -15.17833, 49.635; alt. 1100 m; 12 Mar 2003; B.L. Fisher et al. leg.; CASENT0038295, CASENT0038311 (CASC). •3w., 1s., 1m.; Parc National de Zahamena, Tetezambatana forest, near junction of Nosivola and Manakambahiny Rivers; -17.74298, 48.72936; alt. 860 m; 18 Feb 2009; B.L. Fisher et al. leg.; CASENT0150555, CASENT0150556, CASENT0151067, CASENT0235021 (CASC).

###### Diagnosis.

***Major workers*.** Head in full face view rectangular, slightly widening posteriorly; sides of the head with moderately dense, long, erect pilosity; frons with thick, sparse to moderately sparse, longitudinal rugae, interspaces smooth or with indistinct but dense rugulae; inner hypostomal teeth distinct, low, thick and triangular, with rounded apex; outer hypostomal teeth higher and slightly wider than inner hypostomal teeth, thick, with rounded tips, and wide base; inner and outer teeth closely spaced and connected by indistinct concavity; propodeal spines moderately long, narrow, with base slightly wider than top; first gastral tergite finely shagreened. ***Minor workers*.** Whole head foveolate; thick, sparse, longitudinal to irregular rugae overlie foveolate sculpture on frons and malar area, upper part of frons with rugae curved outward; sparse, irregular to arcuate, thick rugae overlie foveolate sculpture on vertex and genae; promesonotum low, short, flat or slightly convex, with steep posterior declivity; mesosoma foveolate, with sparse, thick and irregular rugae overlying foveolae; anepisternum, katepisternum, and mesosoma smooth.

###### Description.

**Major workers.** Measurements (*N* = 10): HL: 1.7–1.92 (1.8); HW: 1.57–1.7 (1.62); SL: 0.64–0.71 (0.68); EL: 0.18–0.2 (0.19); WL: 1.09–1.27 (1.18); PSL: 0.24–0.27 (0.26); MTL: 0.68–0.73 (0.7); PNW: 0.77–0.8 (0.78); PTW: 0.23–0.28 (0.25); PPW: 0.62–0.73 (0.68); CI: 87.2–95.0 (90.1); SI: 39.0–44.0 (41.7); PSLI: 13.4–15.9 (14.4); PPI: 32.4–39.7 (36.2); PNI: 46.4–50.0 (48.2); MTI: 41.1–44.4 (43.4). ***Head*.** In full-face view rectangular, slightly widening posteriorly, anterior of eyes relatively straight, posterior of eyes slightly convex (Fig. 54B). In lateral view sub-rectangular; ventral and dorsal faces finely convex; dorsal face finely depressed posteriorly, forming shallow transverse depression between frons and occipital lobes; inner hypostomal teeth invisible. Sides of the head with moderately dense, long, erect pilosity; whole head with dense, moderately long, suberect to erect pilosity. Antennal scrobes indistinct and not delimited by carinulae. Occipital lobes shiny, with thick, sparse, irregular rugae, interspaces smooth to finely rugulose; frons with thick, sparse to moderately sparse, longitudinal rugae, interspaces smooth or with indistinct but dense rugulae; lateral sides of head with thin, dense, irregularly rugoreticulate; malar area with dense and thin longitudinal rugulae; genae with fine, thin, dense rugulae. Clypeus shiny and smooth, with thin, longitudinal rugulae on lateral sides; median notch present, narrow and moderately deep; median longitudinal carina absent; lateral longitudinal carinae absent. Scape, when laid back, reaching midlength of head; pilosity decumbent to erect (Fig. 54B, D). Inner hypostomal teeth distinct, low, thick, and triangular, with rounded apex; outer hypostomal teeth higher and slightly wider than inner hypostomal teeth, thick, with rounded tips, and wide base; inner and outer teeth closely spaced and connected by indistinct concavity (Fig. 85E). ***Mesosoma*.** In lateral view, promesonotum short, angular, and relatively low, posterior mesonotum steep, with moderately large, tubercle-like projections; promesonotal groove absent; metanotal groove absent or indistinct; propodeal spines moderately long, narrow, with wide base, apex rounded; humeral area with small and flat tubercles (Fig. 54D). Surface shiny, pronotum, mesonotum, anepisternum, and katepisternum smooth, with very sparse, thick to thin, irregular rugae, lateral sides of propodeum with few thick, longitudinal rugae; sometimes surface with additional indistinct and sparse rugulae on promesonotum. Pilosity moderately sparse, long, and erect (Fig. 54D, F). ***Petiole*.** Shiny, finely shagreened to smooth; peduncle short, with small, rounded horizontal lobes on its basal part; node moderately high and narrow, with convex apex, in rear view node relatively straight; pilosity moderately dense and erect (Fig. 54D, F). ***Postpetiole*.** Shiny and finely shagreened; short and rounded; in dorsal view sides with relatively long, acute, triangular projections; pilosity long, moderately dense and erect (Fig. 54D, F). ***Petiole*.** First gastral tergite shiny and finely shagreened; pilosity dense, long and erect (Fig. 54D, F). ***Colour*.** Unicolourous, brown to dark brown; legs dark yellow (Fig. 54D, F).

**Minor workers.** Measurements (*N* = 10): HL: 0.57–0.62 (0.59); HW: 0.53–0.57 (0.54); SL: 0.56–0.61 (0.59); EL: 0.11–0.12 (0.11); WL: 0.69–0.79 (0.75); PSL: 0.11–0.14 (0.12); MTL: 0.44–0.48 (0.47); PNW: 0.36–0.4 (0.37); PTW: 0.06–0.09 (0.07); PPW: 0.11–0.13 (0.12); CI: 88.5–94.6 (91.8); SI: 103.2–112.8 (107.8); PSLI: 17.9–23.6 (21.0); PPI: 54.6–67.2 (61.4); PNI: 66.8–71.8 (69.2); MTI: 80.0–90.7 (86.0). ***Head*.** Occipital margin straight or indistinctly concave; occipital carina indistinct, weakly developed (Fig. 54A). Pilosity moderately dense, long, erect. Whole head foveolate; thick, sparse, longitudinal to irregular rugae overlie foveolate sculpture on frons and malar area, upper part of frons with rugae curved outward; sparse, irregular to arcuate, thick rugae overlie foveolate sculpture on vertex and genae. Clypeus with median longitudinal carina present; two lateral longitudinal carinae absent. Scape, when laid back, surpassing the posterior head margin by two-fifths of its length; pilosity erect (Fig. 54A, C). ***Mesosoma*.** In lateral view, promesonotum low, short, flat or slightly convex, with steep posterior declivity; promesonotal groove absent; metanotal groove absent or indistinct; propodeal spines moderately long, triangular, apex acute (Fig. 54C). Sculpture foveolate, with sparse, thick, and irregular rugae overlying foveolae; anepisternum, katepisternum, and mesosoma smooth. Pilosity sparse, long, and erect (Fig. 54C, E). ***Petiole*.** Peduncle moderately long and thin; node moderately high, triangular, and small; with few long, erect setae (Fig. 54C, E). ***Postpetiole*.** Short, low, and convex; with few short, erect setae (Fig. 54C, E). ***Petiole*.** With sparse, erect pilosity (Fig. 54C, E). ***Colour*.** Unicolourous, yellowish brown to brown; legs yellow (Fig. 54C, E).

###### Etymology.

From the type locality.

###### Biology.

The species was collected between 450–1200 m in elevation, in transitional humid forest, rainforest, and montane rainforest. Nests were located in rotten logs and rotten twigs on the ground.

###### Comments.

*Pheidole makirovana* sp. nov. is most similar to *P. curvistriata* sp. nov. ***Major workers*.***Pheidole makirovana* sp. nov. differs from *P. curvistriata* sp. nov. by pronotum, mesonotum, anepisternum, and katepisternum with smooth notches and outer hypostomal teeth wider than inner hypostomal teeth. ***Minor workers*.***Pheidole makirovana* sp. nov. differs from *P. curvistriata* sp. nov. by rugae on upper part of frons curved outward and smooth mesosoma.

##### Pheidole mantadia sp. nov.

Taxon classificationAnimaliaHymenopteraFormicidae

http://zoobank.org/E52BA14D-F9F4-429E-9A36-372006991B18

**Figure 55.**
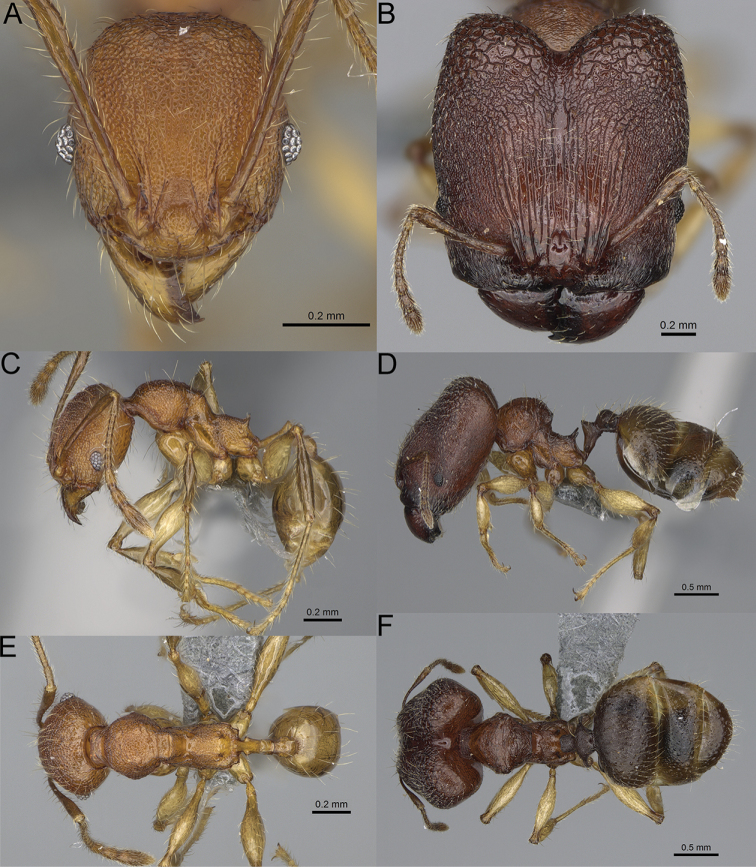
*Pheidole mantadia* sp. nov., full-face view (**A**), profile (**C**), and dorsal view (**E**) of paratype minor worker (CASENT0923169) and full-face view (**B**), profile (**D**), and dorsal view (**F**) of holotype major worker (CASENT0297006).

Figs 55A–F , 85G , 87K

###### Type material.

***Holotype*.** Madagascar. •1 major worker; Toamasina; Corridor Forestier Analamay-Mantadia, Tsaravoniana; -18.76124, 48.42134; alt. 939 m; 3 Dec 2012; B.L. Fisher et al. leg.; BLF30003, CASENT0297006 (CASC). ***Paratype*.** Madagascar. •1 w.; same data as for holotype; CASENT0923169 (CASC).

###### Diagnosis.

***Major workers*.** Head in full face view rectangular, slightly widening posteriorly; sides of the head with sparse, relatively short, erect pilosity; frons with thick, sparse, longitudinal rugae, interspaces smooth to indistinctly rugulose; inner hypostomal teeth distinct, low, thick, bulge-like, with rounded apex; outer hypostomal teeth higher, thick, with rounded tips, triangular; inner and outer teeth closely spaced and connected by indistinct concavity; propodeal spines moderately long, with base slightly wider than top; first gastral tergite shagreened. ***Minor workers*.** Whole head foveolate, frons and malar area with few additional, indistinct, longitudinal rugae, vertex with indistinct irregular rugae, genae with sculpture reduced to absent; promesonotum low, slightly convex, with steep posterior declivity; mesosoma foveolate, katepisternum, and mesonotum smooth.

###### Description.

**Major workers.** Measurements (*N* = 1): HL: 1.68; HW: 1.52; SL: 0.67; EL: 0.17; WL: 1.21; PSL: 0.24; MTL: 0.68; PNW: 0.7; PTW: 0.2; PPW: 0.61; CI: 90.5; SI: 43.9; PSLI: 14.5; PPI: 33.9; PNI: 46.1; MTI: 44.7. ***Head*.** In full-face view rectangular, slightly widening posteriorly, anterior of eyes relatively straight, posterior of eyes convex (Fig. 55B). In lateral view sub-rectangular; ventral and dorsal faces finely convex; dorsal face finely depressed posteriorly, forming indistinct transverse depression between frons and occipital lobes; inner hypostomal teeth invisible. Antennal scrobes indistinct and not delimited by carinulae. Sides of the head with sparse, relatively short, erect pilosity; whole head with dense, moderately long, suberect to erect pilosity. Occipital lobes shiny, with thick, sparse, irregular rugae, interspaces smooth to finely rugulose; frons with thick, sparse, longitudinal rugae, interspaces smooth to indistinctly rugulose; lateral sides of head with fine and sparse rugulae, area between rugulae finely foveolate; malar area with dense and thin longitudinal rugae, interspaces smooth; genae with fine, thin, and dense rugulae. Clypeus shiny and smooth, with thin, longitudinal rugulae on lateral sides; median notch present, narrow, and moderately deep; median longitudinal carina indistinct; lateral longitudinal carinae absent. Scape, when laid back, reaching the midlength of head; pilosity decumbent to erect (Fig. 55B, D). Inner hypostomal teeth distinct, low, thick, bulge-like, with rounded apex; outer hypostomal teeth higher, thick, with rounded tips, triangular (Fig. 85G). ***Mesosoma*.** In lateral view, promesonotum short, angular, and relatively low, posterior mesonotum steep, with small tubercle-like projections; promesonotal groove absent; metanotal groove absent or indistinct; propodeal spines moderately long, with base slightly wider than top, apex rounded; humeral area with small and flat tubercles (Fig. 55D). Surface shiny, finely foveolate, katepisternum, anepisternum, and lateral surfaces of propodeum smooth, with few irregular, thick rugae. Pilosity moderately sparse, long, and erect (Fig. 55D, F). ***Petiole*.** Shiny, finely foveolate; peduncle short, with small, rounded, horizontal lobes on its basal part; node moderately high and narrow, with convex apex, in rear view node relatively straight; pilosity moderately sparse and erect (Fig. 55D, F). ***Postpetiole*.** Shiny, with fine and sparse foveolae; short and rounded; in dorsal view sides with relatively long, acute, horn-like projections; pilosity long, moderately dense and erect (Fig. 55D, F). ***Petiole*.** First gastral tergite shiny and shagreened; pilosity moderately dense, long, and erect (Fig. 55D, F). ***Colour*.** Unicolourous, reddish brown to brown; legs yellow to brown (Fig. 55D, F).

**Minor workers.** Measurements (*N* = 1): HL: 0.58; HW: 0.52; SL: 0.6; EL: 0.11; WL: 0.7; PSL: 0.1; MTL: 0.48; PNW: 0.37; PTW: 0.07; PPW: 0. 1; CI: 89.2; SI: 115.7; PSLI: 17.0; PPI: 64.4; PNI: 71.4; MTI: 91.7. ***Head*.** Occipital margin straight or indistinctly concave; occipital carina indistinct, weakly developed (Fig. 55A). Pilosity moderately sparse, long, and erect. Whole head foveolate, frons and malar area with few additional, indistinct, longitudinal rugae, vertex with indistinct irregular rugae, genae with sculpture reduced to absent. Clypeus shiny, foveolate to rugoreticulate; median longitudinal carina present; two lateral longitudinal carinae absent. Scape, when laid back, surpassing the posterior head margin by two-fifths of its length; pilosity erect (Fig. 55A, C). ***Mesosoma*.** In lateral view, promesonotum low, slightly convex, with steep posterior declivity; promesonotal groove absent; metanotal groove indistinct; propodeal spines short, triangular, apex acute (Fig. 55C). Sculpture foveolate, katepisternum and mesonotum smooth. Pilosity sparse, long, and erect (Fig. 55C, E). ***Petiole*.** Peduncle moderately long and thin; node low, globular, and small; with few long, erect setae (Fig. 55C, E). ***Postpetiole*.** Short, low, and convex; with few short, erect setae (Fig. 55C, E). ***Petiole*.** With sparse, erect pilosity (Fig. 55C, E). ***Colour*.** Unicolourous, yellowish brown to brown (Fig. 55C, E).

###### Etymology.

From the type locality.

###### Biology.

The species was collected at 939 m in elevation, in rainforest. Nest was located in rotten log.

###### Comments.

*Pheidole mantadia* sp. nov. is most similar to *P. curvistriata* sp. nov. and *P. moramanaensis* sp. nov. ***Major workers.****Pheidole mantadia* sp. nov. can be distinguished from *P. curvistriat*a sp. nov. by presence of fine foveolate sculpture on frons and mesosoma, low and thick inner hypostomal teeth, which are wider than high and thick, high outer hypostomal teeth; from *P. moramanaensis* sp. nov. by sparser and shorter pilosity on sides of head. ***Minor workers*.***Pheidole mantadia* sp. nov. can be distinguished from *P. curvistriata* sp. nov. by frons and malar with indistinct, longitudinal rugae, and vertex with indistinct irregular and never arcuate rugae, absence of irregular rugae on mesosoma, and smooth mesonotum; from *P. moramanaensis* sp. nov. by frons and malar area with indistinct, longitudinal rugae, and never smooth clypeus.

#### Revision of the *Pheidole nemoralis* group

**Diagnosis. *Major workers*.** Body size small; head in full-face view square or subrectangular, anterior and posterior sides of eyes slightly convex, in lateral view sub-oval or sub-rectangular, dorsal face not depressed or finely depressed posteriorly; antennal scrobes indistinct and not delimited by carinulae; scrobe surface foveolate with sparse, thick, longitudinal to irregular rugae; occipital lobes with indistinct to distinct, sparse, irregular rugae, interspaces smooth to foveolate; frons with dense, thick, and longitudinal rugae, interspaces smooth to rugo-foveolate; promesonotum moderately short, relatively low and convex; promesonotal and metanotal groove absent; propodeal spines small to moderately long, triangular; mesosoma with fine foveolae or rugo-foveolae; gaster smooth or indistinctly shagreened; body bright brown to dark brown. ***Minor workers*.** Head foveolate, sometimes with reduced or absent sculpture on genae; scape short, when laid back surpassing the posterior head margin by one to two-fifths of its length; promesonotum low, short or long, slightly convex, with relatively steep posterior declivity or declivity smoothly declining towards propodeum; promesonotal groove absent; mesosoma entirely foveolate or foveolate with smooth notches on its lateral sides; propodeal spines minute to moderately long, triangular; body yellow.

**Comments.** Major workers of this group can be distinguished based on the combination of the following characters: relatively small body size; square or subrectangular head in full-face view, indistinct and not delimited by carinulae antennal scrobes with scrobe surface foveolate with sparse, thick, longitudinal to irregular rugae; occipital lobes with indistinct to distinct, sparse, irregular rugae with smooth to foveolate interspaces; frons with dense, thick, and longitudinal rugae, moderately short and relatively low promesonotum, lack of promesonotal and metanotal grooves, entirely sculptured mesosoma and bright brown to dark brown body.

The group is divided into two complexes. The *P. nemoralis* complex contains two sympatric species distributed across evergreen forest and central highlands biomes: *P. nemoralis* and *P. ala* sp. nov. The *P. bemarivoensis* complex also contains two species: *P. bemarahaensis* sp. nov., and *P. bemarivoensis* sp. nov. *Pheidole bemarahaensis* is known only from its type locality (Parc National Tsingy de Bemaraha, Mahajanga) and is sympatric with *P. bemarivoensis* distributed in lowlands spread between Belo and Ambilobe.

##### Key to the *Pheidole nemoralis* group

**Table:** 

1	Major workers. Occipital lobes with indistinct to distinct, sparse, irregular rugae, interspaces sometimes foveolate; head in full-face view sub-rectangular; dorsal face depressed posteriorly (Fig. 56). Minor workers. Head and mesosoma foveolate with smooth notches on genae and lateral sides of mesosoma (Fig. 56)	**2**
–	Major workers. Occipital lobes with indistinct, sparse, irregular rugae, interspaces foveolate and sculpture weakens posteriorly; head in full-face view square; dorsal face not depressed posteriorly (Fig. 57). Minor workers. Head and mesosoma entirely foveolate (Fig. 57)	**3**
2	Major workers. Frons with rugo-foveolate sculpture between rugae and gaster smooth (Fig. 56B, F). Minor workers. Mesosoma with posterior declivity smoothly declining towards propodeum, genae smooth, propodeal spines minute (Fig. 56C)	***P. ala* sp. nov.**
–	Major workers. Frons, at least on the anterior part, with smooth sculpture between rugae, gaster finely shagreened (Fig. 56A, E). Minor workers. Mesosoma with relatively steep posterior declivity, genae never entirely smooth and propodeal spines small (Fig. 56D)	***P. nemoralis* Forel**
3	Major workers. Sides of the head with sparse, relatively short, erect pilosity; at least lower part of frons smooth between rugae, genae finely rugo-foveolate (Fig. 57C, D). Minor workers. Propodeal spines moderately long (Fig. 57F)	***P. bemarahaensis* sp. nov.**
–	Major workers. Sides of the head with sparse, long, erect pilosity; frons finely foveolate between rugae, genae finely foveolate with sculpture sometimes reduced (Fig. 57A, B). Minor workers. Propodeal spines small and indistinct (Fig. 57E)	***P. bemarivoensis* sp. nov.**

**Figure 56.**
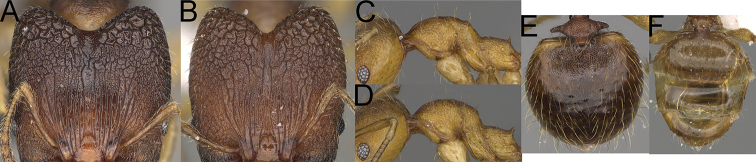
*Pheidole nemoralis* Forel, head of major worker (**A**), profile of minor worker (**D**), gaster of major worker (**E**). *Pheidole ala* sp. nov., head of major worker (**B**), profile of minor worker (**C**), gaster of major worker (**F**).

**Figure 57.**
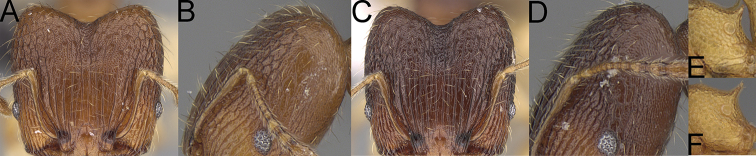
*Pheidole bemarivoensis* sp. nov., head of major worker (**A–B**), propodeal spines of minor worker (**E**). *Pheidole bemarahaensis* sp. nov., head of major worker (**C–D**), propodeal spines of minor worker (**F**).

#### Revision of the *Pheidole nemoralis* complex

**Diagnosis. *Major workers*.** Head in full-face view sub-rectangular, in lateral view sub-rectangular, ventral and dorsal faces relatively convex and dorsal face finely depressed posteriorly; scrobe surface foveolate with sparse, thick, irregular rugae; occipital lobes with indistinct to distinct, sparse, irregular rugae, interspaces sometimes foveolate, and sculpture sometimes weakening posteriorly; frons with dense, thick, and longitudinal rugae, interspaces distinctly to finely foveolate or rugo-foveolate; propodeal spines small; mesosoma with fine but distinct foveolae or rugo-foveolae, and with additional sparse, fine, and irregular rugae; gaster smooth or finely shagreened; body bright brown to dark brown. ***Minor workers*.** Head foveolate; genae with reduced sculpture or smooth; scape, when laid back, surpassing the posterior head margin by one-fifth of its length; promesonotum low, short, slightly convex, with relatively steep posterior declivity or declivity smoothly declining towards propodeum; metanotal groove absent or indistinct; propodeal spines minute to small, triangular; mesosoma foveolate, katepisternum, anepisternum, and mesonotum smooth.

**Comments.** Major workers of this complex can be distinguished based on a combination of the following characters: head in full-face and lateral views sub-rectangular; foveolate scrobe surface with sparse, thick, irregular rugae; small and triangular propodeal spines; mesosoma with fine but distinct foveolae or rugo-foveolae, and additional sparse, irregular rugae; bright brown to dark brown body. Minor workers can be separated based on foveolate head with genae smooth or with reduced sculpture and smooth katepisternum, anepisternum, and mesonotum; minute to small propodeal spines, and yellow body.

##### Pheidole nemoralis

Taxon classificationAnimaliaHymenopteraFormicidae

Forel, 1892

**Figure 58.**
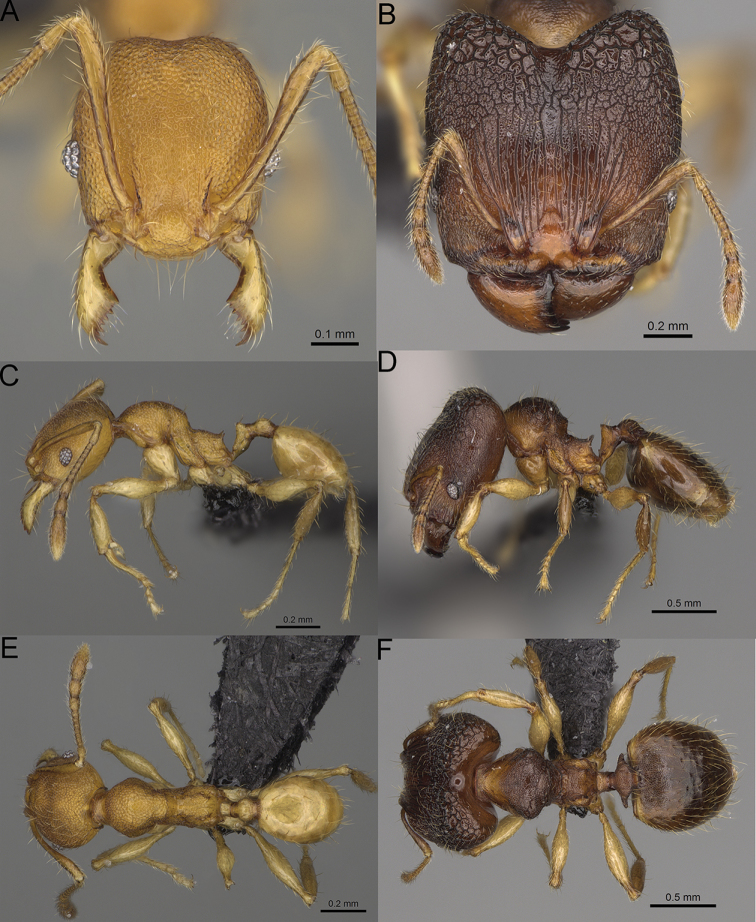
*Pheidole nemoralis* Forel, full-face view (**A**), profile (**C**), and dorsal view (**E**) of minor worker (CASENT0430850) and full-face view (**B**), profile (**D**), and dorsal view (**F**) of major worker (CASENT0460136).

Figs 58A–F , 85N , 87S

###### Type material.

*Pheidole nemoralis* Forel, 1892: 526 (s.w.). Lectotype [designated here]: major worker (top specimen, CASENT0101323): Madagascar, Antananarivo, Andrangoloaka forest, coll. Sikora (MHNG) [examined]. Paralectotypes: 1 minor worker (CASENT0101324, bottom specimen, the same pin as lectotype) (MHNG) [examined], 1 minor worker (CASENT0101584) (MHNG) [examined]: the same data as lectotype.

###### Other material.

Madagascar. –***Antananarivo***: •1w., 1s.; Réserve Spéciale d’Ambohitantely, Forêt d Ambohitantely, Jardin Botanique, 24.1 km 59°NE d Ankazobe; -18.17139, 47.28182; alt. 1620 m; 17 Apr 2001; B.L. Fisher et al. leg.; CASENT0458918, CASENT0458967 (CASC). •2w.; Réserve Speciale d’Ambohitantely; -18.22444, 47.2774; alt. 1490 m; 9 Mar 2012; B.L. Fisher et al. leg.; CASENT0274645 (CASC). •2w.; Réserve Speciale d’Ambohitantely; -18.18762, 47.28576; alt. 1580 m; 8 Mar 2012; B.L. Fisher et al. leg.; CASENT0274610 (CASC). –***Fianarantsoa***: •1w., 1s.; 40 km S Ambalavao, Rés. Andringitra; -22.21667, 46.96667; alt. 1225 m; 19 Oct 1993; B.L. Fisher et al. leg.; CASENT0198554 (CASC). •2w., 1s.; 8.0 km NE Ivohibe; -22.42167, 46.89833; alt. 1200 m; 3 Nov 1997; B.L. Fisher et al. leg.; CASENT0198413, CASENT0198886 (CASC). •2w., 1s.; Parc National Befotaka-Midongy, Papango 27.7 km S Midongy-Sud, Mount Papango; -23.83517, 46.96367; alt. 940 m; 15 Nov 2006; B.L. Fisher et al. leg.; CASENT0119444, CASENT0235039 (CASC). •1w., 1s.; Parc National Befotaka-Midongy, Papango 28.5 km S Midongy-Sud, Mount Papango; -23.84083, 46.9575; alt. 1250 m; 17 Nov 2006; B.L. Fisher et al. leg.; CASENT0118392 (CASC). •1w.; Parc National d’Isalo, Sahanafa River, 29.2 km 351°N Ranohira; -22.31333, 45.29167; alt. 500 m; 10 Feb 2003; B.L. Fisher et al. leg.; CASENT0031712 (CASC). •7w., 5s.; R.S. Ivohibe 8.0 km E Ivohibe; -22.48333, 46.96833; alt. 1200 m; 15 Oct 1997; B.L. Fisher et al. leg.; CASENT0198530, CASENT0198531, CASENT0198887, CASENT0198888 (CASC). •1w., 1s.; R.S. Ivohibe, 7.5 km ENE Ivohibe; -22.47, 46.96; alt. 900 m; 7 Oct 1997; B.L. Fisher et al. leg.; CASENT0196902, CASENT0198896 (CASC). –***Toamasina***: •1w.; Bevolota 17.1 km N Andasibe; -18.77071, 48.43164; alt. 995 m; 12 Dec 2007; B.L. Fisher et al. leg.; CASENT0135188 (CASC). •2s.; Montagne d’Akirindro 7.6 km 341°NNW Ambinanitelo; -15.28833, 49.54833; alt. 600 m; 17 Mar 2003; B.L. Fisher et al. leg.; CASENT0038918, CASENT0039092 (CASC). –***Toliara***: •1w.; Forêt Classée d’Analavelona, 29.2 km 343°NNW Mahaboboka; -22.675, 44.19; alt. 1100 m; 18 Feb 2003; B.L. Fisher et al. leg.; CASENT0032166 (CASC). •7w., 7s., 1q.; Parc National d’Andohahela, Col du Sedro, 3.8 km 113°ESE Mahamavo, 37.6 km 341°NNW Tolagnaro; -24.76389, 46.75167; alt. 900 m; 21 Jan 2002; B.L. Fisher et al. leg.; CASENT0430849, CASENT0430850, CASENT0460132, CASENT0460136, CASENT0460137, CASENT0460138, CASENT0460140, CASENT0479177, CASENT0479179, CASENT0484098 (CASC).

###### Diagnosis.

***Major workers*.** Head in full-face view sub-rectangular, anterior and posterior sides of eyes slightly convex; sides of the head with sparse, relatively long, erect pilosity; scrobe surface shiny, finely foveolate with sparse, thick, irregular rugae; inner hypostomal teeth distinct, closely spaced, triangular, with rounded apex directed inward, and wide base; outer hypostomal teeth slightly thinner and approximately as high as outer hypostomal teeth, and with wider base; gaster finely shagreened; body bright brown to dark brown. ***Minor workers*.** Head foveolate, genae with reduced sculpture; promesonotum low, short, slightly convex, with relatively steep posterior declivity; mesosoma foveolate, katepisternum, anepisternum, and mesosoma smooth; propodeal spines small, triangular; body yellow.

###### Redescription.

**Major workers.** Measurements (*N* = 10): HL: 1.02–1.19 (1.11); HW: 0.99–1.12 (1.05); SL: 0.44–0.5 (0.47); EL: 0.12–0.15 (0.13); WL: 0.75–0.93 (0.84); PSL: 0.14–0.2 (0.16); MTL: 0.43–0.51 (0.47); PNW: 0.49–0.59 (0.55); PTW: 0.13–0.16 (0.15); PPW: 0.36–0.46 (0.41); CI: 92.6–96.4 (94.9); SI: 42.6–48.3 (44.6); PSLI: 13.1–16.7 (14.7); PPI: 31.6–38.4 (35. 1); PNI: 49.7–53.2 (51.8); MTI: 43.2–46.1 (44.5). ***Head*.** In full-face view sub-rectangular, anterior and posterior sides of eyes slightly convex (Fig. 58B). In lateral view sub-rectangular; ventral and dorsal faces relatively convex; dorsal face finely depressed posteriorly, forming shallow transverse depression between frons and occipital lobes; inner hypostomal teeth invisible. Sides of the head with sparse, relatively long, erect pilosity; whole head with dense, short, suberect to erect pilosity. Antennal scrobes indistinct and not delimited by carinulae; scrobe surface shiny, finely foveolate with sparse, thick, irregular rugae. Occipital lobes shiny, with fine foveolae and distinct, sparse, irregular rugae, sculpture not weakening posteriorly; frons and malar area with dense, thick, and longitudinal rugae, interspaces distinctly to finely foveolate, anterior part of frons with smooth sculpture between rugae; genae shiny, with distinct to fine rugulae. Centre of clypeus smooth and shiny, lateral sides with longitudinal rugae; median notch present, moderately wide and shallow; median longitudinal carina absent; lateral longitudinal carinae present. Scape, when laid back, slightly exceeding the midlength of head; pilosity suberect to erect (Fig. 58B, D). Inner hypostomal teeth distinct, closely spaced, triangular, with rounded apex directed inward, and wide base; outer hypostomal teeth slightly thinner and approximately as high as outer hypostomal teeth, and with wider base (Fig. 85N). ***Mesosoma*.** In lateral view, promesonotum short, relatively low, and convex, dorsal mesonotum slightly concave, posterior mesonotum steep, with small tubercle-like projections; promesonotal groove absent; metanotal groove absent; propodeal spines small, triangular, with rounded apex; humeral area laterally well produced (Fig. 58D). Surface shiny, with fine but distinct foveolae or rugo-foveolae, and with additional sparse, fine and irregular rugae. Pilosity moderately sparse, long, and erect (Fig. 58D, F). ***Petiole*.** Shiny and with fine foveolae; peduncle short, with distinct horizontal lobes on its basal part; node relatively high, triangular, with rounded apex, in rear view node slightly convex; pilosity moderately sparse and erect (Fig. 58D, F). ***Postpetiole*.** Shiny, finely shagreened; in dorsal view sides with moderately long, acute, and triangular projections; pilosity long, moderately long and erect (Fig. 58D, F). ***Petiole*.** Shiny and finely shagreened; pilosity moderately dense, short, and erect (Fig. 58D, F). ***Colour*.** Unicolourous, bright brown to dark brown; legs yellowish brown (Fig. 58D, F).

**Minor workers.** Measurements (*N* = 10): HL: 0.49–0.53 (0.51); HW: 0.43–0.47 (0.44); SL: 0.45–0.48 (0.46); EL: 0.09–0.1 (0.1); WL: 0.56–0.63 (0.6); PSL: 0.08–0.09 (0.08); MTL: 0.34–0.37 (0.36); PNW: 0.27–0.31 (0.29); PTW: 0.06–0.08 (0.07); PPW: 0.11–0.13 (0.12); CI: 84.8–89.4 (87.4); SI: 100.6–107.9 (104.2); PSLI: 14.5–18.1 (16.4); PPI: 53.3–65.1 (60.1); PNI: 60.2–68.0 (65.4); MTI: 76.0–83.0 (80.3). ***Head*.** Occipital margin straight or indistinctly concave; occipital carina absent (Fig. 58A). Pilosity moderately sparse, moderately long, suberect to erect. Head foveolate; genae with reduced sculpture. Clypeus with fine and sometimes reduced foveolae; median longitudinal carina absent; two lateral longitudinal carinae absent. Scape, when laid back, surpassing posterior head margin by one-fifth of its length; pilosity erect (Fig. 58A, C). ***Mesosoma*.** In lateral view, promesonotum low, short, slightly convex, with relatively steep posterior declivity; promesonotal groove absent; metanotal groove absent; propodeal spines small, triangular, apex acute (Fig. 58C). Sculpture foveolate; katepisternum, anepisternum, and mesonotum smooth. Pilosity moderately sparse, short, and erect (Fig. 58C, E). ***Petiole*.** Peduncle very short and thin with ventral face slightly convex; with few short, erect setae (Fig. 58C, E). ***Postpetiole*.** Short, low, and convex; with few short, erect setae (Fig. 58C, E). ***Petiole*.** With moderately sparse, erect pilosity (Fig. 58C, E). ***Colour*.** Unicolourous, yellow (Fig. 58C, E).

###### Biology.

The species was collected between 10–1620 m in elevation, in montane rainforest, rainforest, montane shrubland, open secondary vegetation, gallery forest, and littoral rainforest. Nests were located in rotten logs and tree stumps, and in dead twigs above ground.

###### Comments.

*Pheidole nemoralis* is most similar to *P. ala* sp. nov. ***Major workers*.***Pheidole nemoralis* differs from *P. ala* sp. nov. in at least anterior part of frons with partially smooth sculpture between rugae and finely shagreened gaster. ***Minor workers*.***Pheidole nemoralis* differs from *P. ala* sp. nov. in mesosoma with relatively steep posterior declivity, never entirely smooth genae, and small propodeal spines.

##### Pheidole ala sp. nov.

Taxon classificationAnimaliaHymenopteraFormicidae

http://zoobank.org/28B23A79-FBFE-41AB-A4FB-1029E6941339

**Figure 59.**
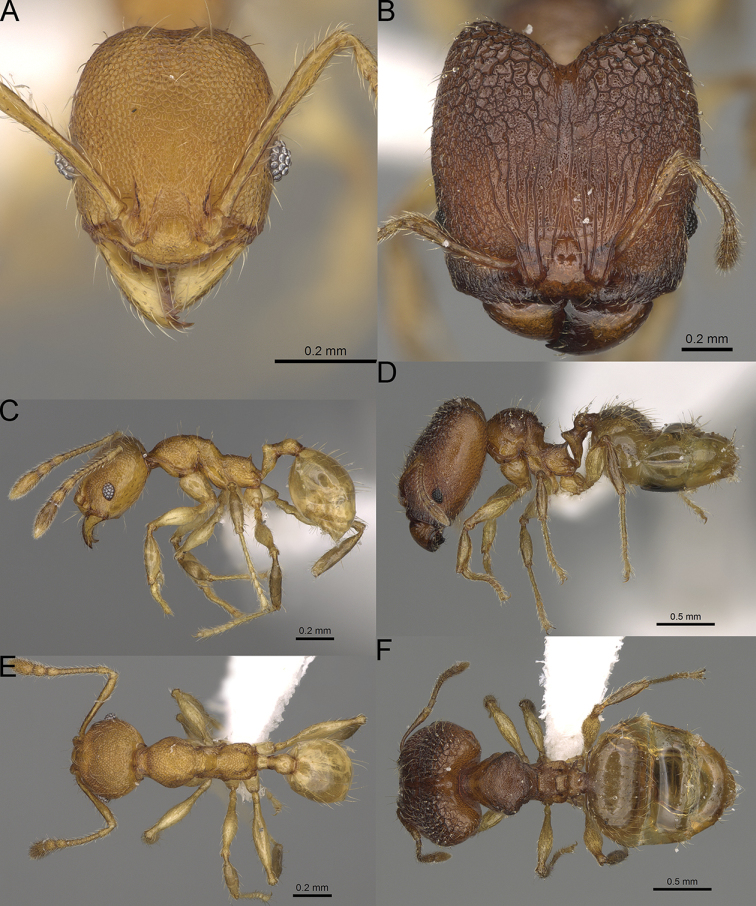
*Pheidole ala* sp. nov., full-face view (**A**), profile (**C**), and dorsal view (**E**) of paratype minor worker (CASENT0427742) and full-face view (**B**), profile (**D**), and dorsal view (**F**) of holotype major worker (CASENT0413606).

Figs 59A–F , 84B , 86B

###### Type material.

***Holotype*.** Madagascar. •1 major worker; Antananarivo; 3 km 41°NE Andranomay, 11.5 km 147°SSE Anjozorobe; -18.47333, 47.96; alt. 1300 m; 5 Dec 2000; B.L. Fisher et al. leg.; BLF02480, CASENT0413606, middle specimen (CASC). ***Paratypes*.** Madagascar. •6w., 2s.; same data as for holotype; CASENT0427741, CASENT0427742, CASENT0872080, CASENT0872192–CASENT0872195 (CASC).

###### Other material.

Madagascar. –***Antananarivo***: •4w., 3s.; 3 km 41°NE Andranomay, 11.5 km 147°SSE Anjozorobe; -18.47333, 47.96; alt. 1300 m; 5 Dec 2000; B.L. Fisher et al. leg.; CASENT0413601, CASENT0413608, CASENT0413609, CASENT0427739, CASENT0427740, CASENT0427745, CASENT0427747 (CASC). •3w., 8s.; Réserve Spéciale d’Ambohitantely, Forêt d Ambohitantely, 20.9 km 72°NE d Ankazobe; -18.22528, 47.28683; alt. 1410 m; 17 Apr 2001; B.L. Fisher et al. leg.; CASENT0437257, CASENT0437264, CASENT0437267, CASENT0479788, CASENT0479789, CASENT0479790, CASENT0480418, CASENT0480437 (CASC). •3w., 1q.; Réserve Speciale d’Ambohitantely; -18.18762, 47.28576; alt. 1580 m; 8 Mar 2012; B.L. Fisher et al. leg.; CASENT0274613, CASENT0274614 (CASC). –***Antsiranana***: •1w., 1s., 1q.; 6.5 km SSW Befingotra, Rés. Anjanaharibe-Sud; -14.75, 49.5; alt. 875 m; 28 Oct 1994; B.L. Fisher et al. leg.; CASENT0198550 (CASC). •1w., 1s., 1q.; 9.2 km WSW Befingotra, Rés. Anjanaharibe-Sud; -14.75, 49.46667; alt. 1200 m; 8 Nov 1994; B.L. Fisher et al. leg.; CASENT0198551 (CASC). –***Fianarantsoa***: •1s.; R.S. Ivohibe 8.0 km E Ivohibe; -22.48333, 46.96833; alt. 1200 m; 15 Oct 1997; B.L. Fisher et al. leg.; CASENT0198029 (CASC). –***Toamasina***: •1s.; Montagne d’Anjanaharibe, 19.5 km 27°NNE Ambinanitelo; -15.17833, 49.635; alt. 1100 m; 12 Mar 2003; B.L. Fisher et al. leg.; CASENT0038234 (CASC). •1w., 1s.; Reserve Betampona, Camp Vohitsivalana, 37.1 km 338° Toamasina; -17.88667, 49.2025; alt. 520 m; 2 Dec 2005; B.L. Fisher et al. leg.; CASENT0067738 (CASC). –Toliara: •2w., 1q.; Parc National d’Andohahela, Col du Sedro, 3.8 km 113°ESE Mahamavo, 37.6 km 341°NNW Tolagnaro; -24.76389, 46.75167; alt. 900 m; 21 Jan 2002; B.L. Fisher et al. leg.; CASENT0430763, CASENT0430764 (CASC).

###### Diagnosis.

***Major workers*.** Head in full-face view sub-rectangular, anterior and posterior sides of eyes slightly convex; sides of head with sparse, short, erect pilosity; scrobe surface shiny, foveolate with sparse, thick, irregular rugae; closely spaced, triangular, with rounded apex and wide base; outer hypostomal teeth slightly thinner and approximately as high as inner hypostomal teeth, with moderately narrow base, triangular; gaster smooth; body bright brown to dark brown. ***Minor workers*.** Head foveolate, genae smooth; promesonotum low, short, slightly convex, with posterior declivity smoothly declining towards propodeum; mesosoma foveolate, katepisternum, anepisternum, and mesosoma smooth; propodeal spines minute, triangular; body yellow.

###### Description.

**Major workers.** Measurements (*N* = 10): HL: 1.09–1.27 (1.18); HW: 1.03–1.21 (1.11); SL: 0.46–0.53 (0.49); EL: 0.12–0.17 (0.14); WL: 0.85–0.95 (0.9); PSL: 0.16–0.18 (0.17); MTL: 0.46–0.56 (0.51); PNW: 0.55–0.64 (0.59); PTW: 0.13–0.17 (0.15); PPW: 0.34–0.49 (0.44); CI: 91.3–96.0 (94.1); SI: 41.7–47.1 (44.1); PSLI: 13.6–16.1 (14.4); PPI: 31.0–40.8 (35.3); PNI: 51.0–54.6 (53.0); MTI: 44.2–48.0 (45.9). ***Head*.** In full-face view longer than wide, anterior and posterior sides of eyes slightly convex (Fig. 59B). In lateral view sub-rectangular; ventral and dorsal faces finely convex; dorsal face finely depressed posteriorly, forming indistinct transverse depression between frons and occipital lobes; inner hypostomal teeth not visible. Sides of the head with sparse, short, erect pilosity; whole head with dense, moderately short, suberect to erect pilosity. Antennal scrobes indistinct and not delimited by carinulae; scrobe surface shiny, foveolate with sparse, thick, irregular rugae. Occipital lobes shiny, with indistinct, sparse, irregular rugae, sculpture weakening posteriorly; frons with dense, thick, and longitudinal rugae, interspaces distinctly to finely rugo-foveolate; genae shiny, with fine rugulae; malar area with dense, thin rugoreticulation. Centre of clypeus smooth and shiny, lateral sides with longitudinal rugae; median notch present, moderately wide, and shallow; median longitudinal carina absent; lateral longitudinal carinae present. Scape, when laid back, slightly exceeding the midlength of head; pilosity suberect to erect (Fig. 59B, D). Inner hypostomal teeth distinct, closely spaced, triangular, with rounded apex, and wide base; outer hypostomal teeth slightly thinner and approximately as high as inner hypostomal teeth, and with moderately narrow base, triangular (Fig. 84B). ***Mesosoma*.** In lateral view, promesonotum short, relatively low, and convex, dorsal mesonotum slightly concave, posterior mesonotum steep, with small, tubercle-like projections; promesonotal groove absent; metanotal groove absent; propodeal spines small, triangular, with rounded apex; humeral area laterally well produced (Fig. 59D). Surface shiny, with fine but distinct foveolae or rugo-foveolae, and with additional sparse, fine, and irregular rugae; katepisternum smooth; lateral sides of pronotum and propodeum with fading sculpture. Pilosity moderately sparse, long, and erect (Fig. 59D, F). ***Petiole*.** Shiny and with fine foveolae; peduncle short, with indistinct horizontal lobes on its basal part; node relatively high, triangular, with rounded apex, in rear view node slightly convex; pilosity moderately sparse and erect (Fig. 59D, F). ***Postpetiole*.** Shiny, finely shagreened; in dorsal view sides with moderately long, acute, and triangular projections; pilosity long, moderately long, and erect (Fig. 59D, F). ***Petiole*.** Shiny and smooth; pilosity dense, moderately long, and erect (Fig. 59D, F). ***Colour*.** Bright brown to brown; legs yellow to bright brown (Fig. 59D, F).

**Minor workers.** Measurements (*N* = 10): HL: 0.47–0.52 (0.49); HW: 0.42–0.46 (0.44); SL: 0.44–0.48 (0.45); EL: 0.09–0.11 (0.1); WL: 0.58–0.63 (0.6); PSL: 0.07–0.09 (0.08); MTL: 0.34–0.39 (0.37); PNW: 0.28–0.3 (0.28); PTW: 0.07–0.08 (0.07); PPW: 0.1–0.13 (0.12); CI: 86.0–90.2 (88.5); SI: 100.2–108.4 (104.4); PSLI: 14.7–18.2 (16.3); PPI: 53.1–66.1 (60.7); PNI: 62.8–67.9 (65.4); MTI: 80.6 –86.5 (83.8). ***Head*.** Occipital margin straight or indistinctly concave; occipital carina absent (Fig. 58A). Pilosity moderately sparse, moderately long, suberect to erect. Head foveolate; genae smooth. Clypeus with fine and sometimes reduced foveolae; median longitudinal carina absent; two lateral longitudinal carinae absent. Scape, when laid back, surpassing the posterior head margin by one-fifth of its length; pilosity erect (Fig. 59A, C). ***Mesosoma*.** In lateral view, promesonotum low, short, slightly convex, with posterior declivity smoothly declining toward propodeum; promesonotal groove absent; metanotal groove indistinct; propodeal spines minute, triangular, apex acute (Fig. 59C). Sculpture foveolate; katepisternum, anepisternum, and mesonotum smooth. Pilosity sparse, long, and erect (Fig. 59C, E). ***Petiole*.** Peduncle short and thin with ventral face slightly convex; node low, bulge-like, and small; with few short, erect setae (Fig. 59C, E). ***Postpetiole*.** Moderately short, low, and slightly convex; with few short, erect setae (Fig. 59C, E). ***Petiole*.** With moderately sparse, erect pilosity (Fig. 59C, E). ***Colour*.** Unicolourous, yellow (Fig. 59C, E).

###### Etymology.

Malagasy for forest, in reference to habitat of the species.

###### Biology.

The species was collected between 520–1410 m in elevation, in rainforest and montane rainforest. Nests were located in rotten logs, and in rotten twigs on the ground.

###### Diagnosis.

*Pheidole ala* sp. nov. is most similar to *P. nemoralis*. ***Major workers*.***Pheidole ala* sp. nov. differs from *P. nemoralis* in frons with rugo-foveolate sculpture between rugae and never shagreened gaster. ***Minor workers*.***Pheidole ala* sp. nov. differs from *P. nemoralis* in mesosoma with posterior declivity smoothly declining towards propodeum, smooth genae, and minute propodeal spines.

#### Revision of the *Pheidole bemarivoensis* complex

**Diagnosis. *Major workers*.** Body size small; head in full-face view square, in lateral view sub-oval, ventral and dorsal faces convex, dorsal face not depressed posteriorly; scrobe foveolate with sparse, thick, longitudinal to irregular rugulae; occipital lobes foveolate, with indistinct, sparse, irregular rugae; sculpture weakening posteriorly; frons with dense, thick, and longitudinal rugae, interspaces smooth to foveolate; propodeal spines small to moderately long; mesosoma with fine foveolae and with additional rugae; gaster indistinctly shagreened; body brown to dark brown. ***Minor workers*.** Head foveolate; scape, when laid back, surpassing the posterior head margin by one to two-fifths of its length; promesonotum low, long, slightly convex, with relatively steep posterior declivity; metanotal groove present; propodeal spines very small or moderately long, triangular; mesosoma foveolate.

**Comments.** Major workers of this group can be distinguished based on a combination of the following characters: small body size; head in full-face view square and in lateral view sub-oval; foveolate scrobes with additional sparse rugulae; foveolate occipital lobes, with additional sparse, irregular rugae; sculpture weakening posteriorly; indistinctly shagreened gaster and brown to dark brown body. Minor workers can be separated based on small body size, entirely foveolate head and mesosoma; scape, when laid back, surpassing the posterior head margin by one- to two-fifths of its length; long and low promesonotum with relatively steep posterior declivity, and yellow body.

##### Pheidole bemarivoensis sp. nov.

Taxon classificationAnimaliaHymenopteraFormicidae

http://zoobank.org/CB6E1BA0-DC84-4613-85DD-53DC22FCDF80

**Figure 60.**
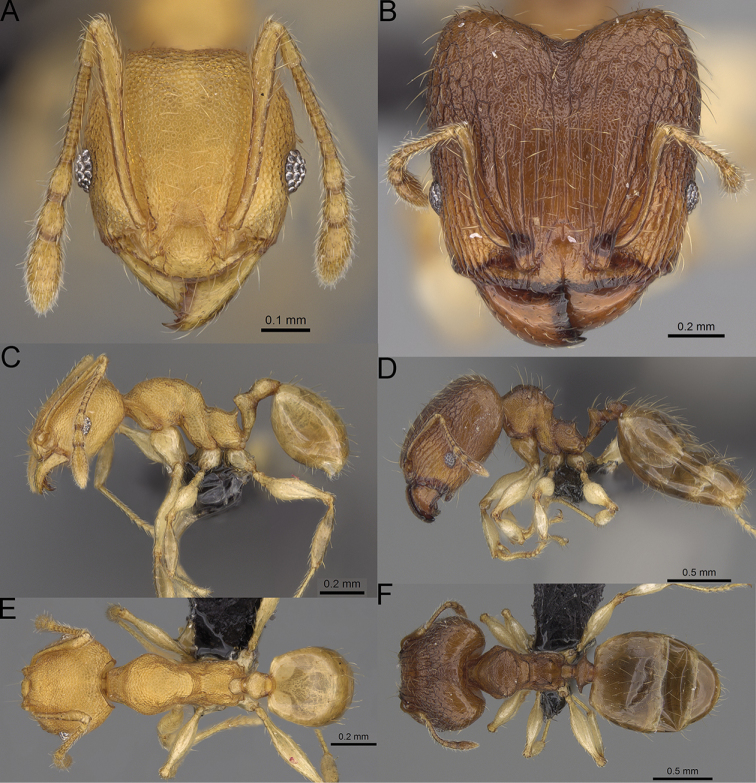
*Pheidole bemarivoensis* sp. nov., full-face view (**A**), profile (**C**), and dorsal view (**E**) of paratype minor worker (CASENT0923160) and full-face view (**B**), profile (**D**), and dorsal view (**F**) of holotype major worker (CASENT0489439).

Figs 60A–F , 84H , 86H

###### Type material.

***Holotype*.** Madagascar. •1 major worker; Mahajanga; Réserve Spéciale de Bemarivo, 23.8 km 223°SW Besalampy; -16.925, 44.36833; alt. 30 m; 19 Nov 2002; B.L. Fisher et al. leg.; BLF06797, CASENT0489439 (CASC). ***Paratypes*.** Madagascar. •8w., 2s., 6m.; same data as for holotype; CASENT0923160, CASENT0235042, CASENT0489441, CASENT0489440, CASENT0489442, CASENT0489438, CASENT0489437, CASENT0872160–CASENT0872168 (CASC).

###### Other material.

Madagascar. –***Antsiranana***: •4w., 3s., 1q.; Ampasindava, Andranomatavy Forest; -13.6648, 47.98702; alt. 275 m; 7 Oct 2013; B.L. Fisher et al. leg.; CASENT0369320, CASENT0369321, CASENT0369333, CASENT0370561 (CASC). •9w., 6s.; Ampasindava, Forêt d’Ambilanivy, 3.9 km 181°S Ambaliha; -13.79861, 48.16167; alt. 600 m; 4 Mar 2001; B.L. Fisher et al. leg.; CASENT0420033–CASENT0420035, CASENT0420037, CASENT0420038, CASENT0421224, CASENT0427706, CASENT0427707, CASENT0427894 (CASC). •2w., 2s.; Forêt Ambato, 26.6 km 33° Ambanja; -13.4645, 48.55167; alt. 150 m; 10 Dec 2004; B.L. Fisher leg.; CASENT0107077, CASENT0107715 (CASC). •4w., 2s.; Galoko chain, Mont Galoko; -13.58745, 48.71419; alt. 380 m; 23 Feb 2013; B.L. Fisher et al. leg.; CASENT0302986, CASENT0302993, CASENT0302995, CASENT0303012, CASENT0303013, CASENT0303015 (CASC). •2w., 1s.; Galoko chain, Mont Galoko; -13.58487, 48.71818; alt. 520 m; 19 Feb 2013; B.L. Fisher et al. leg.; CASENT0302958, CASENT0303564 (CASC). •5w., 4s.; Nosy Be, Réserve Naturelle Intégrale de Lokobe, 6.3 km 112°ESE Hellville; -13.41933, 48.33117; alt. 30 m; 19 Mar 2001; B.L. Fisher et al. leg.; CASENT0421454, CASENT0421455, CASENT0427817, CASENT0427823, CASENT0427829, CASENT0466223, CASENT0466279 (CASC). •1w., 3s.; R.S. Manongarivo, 10.8 km 229°SW Antanambao; -13.96167, 48.43333; alt. 400 m; 8 Nov 1998; B.L. Fisher leg.; CASENT0198891, CASENT0198892 (CASC). •2w., 6s.; Sahamalaza Peninsula, Forêt d’Anabohazo, 21.6 km 247°WSW Maromandia; -14.30889, 47.91433; alt. 120 m; 11 Mar 2001; B.L. Fisher et al. leg.; CASENT0406442, CASENT0406444, CASENT0406447, CASENT0406454, CASENT0458224, CASENT0484564 (CASC). –***Mahajanga***: •4s.; Parc National de Namoroka, 16.9 km 317°NW Vilanandro; -16.40667, 45.31; alt. 100 m; 12 Nov 2002; B.L. Fisher et al. leg.; CASENT0038950, CASENT0038953, CASENT0486454 (CASC). •2w., 1s.; Parc National Tsingy de Bemaraha, 10.6 km ESE 123° Antsalova; -18.70944, 44.71817; alt. 150 m; 16 Nov 2001; B.L. Fisher et al. leg.; CASENT0437624, CASENT0437625, CASENT0437632 (CASC). •6w., 3s., 1m.; Parc National Tsingy de Bemaraha, 2.5 km 62°ENE Bekopaka, Ankidrodroa River; -19.13222, 44.81467; alt. 100 m; 11 Nov 2001; B.L. Fisher et al. leg.; CASENT0425317, CASENT0425320, CASENT0425323, CASENT0443937, CASENT0443958, CASENT0443966, CASENT0443973, CASENT0444152, CASENT0444155 (CASC). •1w.; Réserve forestière Beanka, 53.6 km E Maintirano; -18.04014, 44.53394; alt. 272 m; 25 Oct 2009; B.L. Fisher et al. leg.; CASENT0156697 (CASC). •6w., 8s.; Réserve Spéciale de Bemarivo, 23.8 km 223°SW Besalampy; -16.925, 44.36833; alt. 30 m; 19 Nov 2002; B.L. Fisher et al. leg.; CASENT0491028, CASENT0491029, CASENT0491089, CASENT0491090, CASENT0491091 (CASC).

###### Diagnosis.

***Major workers*.** Small species: HL: HL: 1.01–1.07 (1.04); HW: 0.94–1.02 (0.99), WL: 0.75–0.81 (0.78); head in full-face view square, anterior and posterior sides of eyes slightly convex; in lateral view sub-oval; ventral and dorsal faces convex; dorsal face not depressed posteriorly; sides of the head with sparse, long, erect pilosity; frons with dense, thick, and longitudinal rugae, interspaces superficially foveolate; inner hypostomal teeth distinct, approximately the same size, closely spaced, dentate, with rounded apex and wide base; outer hypostomal teeth with base slightly wider than inner hypostomal teeth; gaster indistinctly shagreened; body brown to dark brown. ***Minor workers*.** Head and mesosoma foveolate; promesonotum low, long, slightly convex, with relatively steep posterior declivity; propodeal spines very small.

###### Description.

**Major workers.** Measurements (*N* = 10): HL: 1.01–1.07 (1.04); HW: 0.94–1.02 (0.99); SL: 0.43–0.51 (0.48); EL: 0.14–0.15 (0.14); WL: 0.75–0.81 (0.78); PSL: 0.14–0.16 (0.15); MTL: 0.44–0.49 (0.47); PNW: 0.41–0.51 (0.45); PTW: 0.1–0.13 (0.12); PPW: 0.33–0.39 (0.36); CI: 92.8–98.1 (95.1); SI: 46.0–51.0 (48.4); PSLI: 13.0–15.5 (14.2); PPI: 29.0–34.3 (32.3); PNI: 43.5–50.1 (45.9); MTI: 44.9–49.3 (47.1). ***Head*.** In full-face view square, anterior and posterior sides of eyes slightly convex (Fig. 60B). In lateral view sub-oval; ventral and dorsal faces convex; dorsal face not depressed posteriorly; inner hypostomal teeth visible. Sides of the head with sparse, long, erect pilosity; whole head with dense, short, suberect to erect pilosity. Antennal scrobes very indistinct and not delimited by carinulae; scrobe surface shiny, finely foveolate with sparse, thick, longitudinal to irregular rugulae in posterior part. Occipital lobes shiny, with fine foveolae and indistinct, sparse, irregular rugae, sculpture weakening posteriorly; frons and malar area with dense, thick, and longitudinal rugae, interspaces superficially foveolate; genae shiny, with fine to indistinct foveolae. Centre of clypeus smooth and shiny, lateral sides with longitudinal rugae; median notch present, narrow, and shallow; median longitudinal carina present; lateral longitudinal carinae present. Scape, when laid back, slightly exceeding the midlength of head; pilosity suberect to erect (Fig. 60B, D). Inner and outer hypostomal teeth distinct, approximately the same size, closely spaced, dentate, with rounded apex and wide base; outer hypostomal teeth with base slightly wider than inner hypostomal teeth (Fig. 84H). ***Mesosoma*.** In lateral view, promesonotum moderately short, relatively low, and convex, posterior mesonotum steep, with small tubercle-like projections; promesonotal groove absent; metanotal groove absent; propodeal spines moderately long, triangular, with rounded apex; humeral area laterally well produced (Fig. 60D). Surface shiny, with fine but distinct foveolae, additional sparse, fine, transverse to irregular rugae on promesonotal dorsum and sometimes propodeum, sculpture slightly weakening on dorsal surface. Pilosity moderately sparse, very long, and erect (Fig. 60D, F). ***Petiole*.** Shiny and with fine foveolae; peduncle short, with short horizontal lobes on its basal part; node relatively high, triangular, with rounded apex, in rear view node straight or dorsoventrally slightly concave; pilosity moderately sparse and erect (Fig. 60D, F). ***Postpetiole*.** Shiny, with fine and sparse foveolae, smooth on dorsum; in dorsal view sides with moderately short, acute, and triangular projections; pilosity moderately long, dense, and erect (Fig. 60D, F). ***Petiole*.** Shiny and indistinctly shagreened; pilosity moderately dense, short, and erect (Fig. 60D, F). ***Colour*.** Unicolourous, brown to dark brown; legs yellow (Fig. 60D, F).

**Minor workers.** Measurements (*N* = 10): HL: 0.43–0.49 (0.47); HW: 0.39–0.44 (0.42); SL: 0.4–0.45 (0.42); EL: 0.09–0.11 (0.1); WL: 0.5–0.58 (0.54); PSL: 0.07–0.1 (0.09); MTL: 0.29–0.35 (0.32); PNW: 0.25–0.29 (0.27); PTW: 0.06–0.08 (0.07); PPW: 0.11–0.14 (0.12); CI: 85.9–92.1 (89.8); SI: 96.5–105.9 (100.3); PSLI: 15.2–20.1 (18.1); PPI: 51.3–62.2 (56.9); PNI: 62.4–67.6 (64.3); MTI: 73.3–79.5 (77.1). ***Head*.** Occipital margin straight or indistinctly concave; occipital carina absent (Fig. 60A). Pilosity moderately sparse, long, suberect to erect. Head foveolate. Clypeus with fine and sometimes reduced foveolae; median longitudinal carina absent; two lateral longitudinal carinae absent. Scape, when laid back, surpassing the posterior head margin by one-fifth of its length; pilosity suberect to erect (Fig. 60A, C). ***Mesosoma*.** In lateral view, promesonotum low, long, slightly convex, with relatively steep posterior declivity; promesonotal groove absent; metanotal groove indistinct; propodeal spines very small, triangular, apex acute (Fig. 60C). Sculpture foveolate. Pilosity sparse, moderately long, and erect (Fig. 60C, E). ***Petiole*.** Shiny and with fine foveolae; peduncle short and thin with ventral face slightly convex; node moderately high, triangular, and small; with few short, erect setae (Fig. 60C, E). ***Postpetiole*.** Moderately long, low, and slightly convex; with few short, erect setae (Fig. 60C, E). ***Petiole*.** With sparse, erect pilosity (Fig. 60C, E). ***Colour*.** Unicolourous, yellow (Fig. 60C, E).

###### Etymology.

From the type locality.

###### Biology.

The species was collected between 30–600 m in elevation, in rainforest, tropical dry forest, and disturbed dry forest. Nests were located in rotten logs, branches on ground, and soil.

###### Comments.

*Pheidole bemarivoensis* sp. nov. is most similar to *P. bemarahaensis* sp. nov. ***Major workers*.***Pheidole bemarivoensis* sp. nov. differs from *P. bemarahaensis* sp. nov. in foveolae covering the whole surface of frons, and weaker sculpture on genae and dorsal surface of promesonotum. ***Minor workers*.***P. bemarivoensis* sp. nov. differs from *P. bemarivoensis* sp. nov. in minute and short propodeal spines.

##### Pheidole bemarahaensis sp. nov.

Taxon classificationAnimaliaHymenopteraFormicidae

http://zoobank.org/C491FD00-0251-4F41-957D-8CDAEA87616E

**Figure 61.**
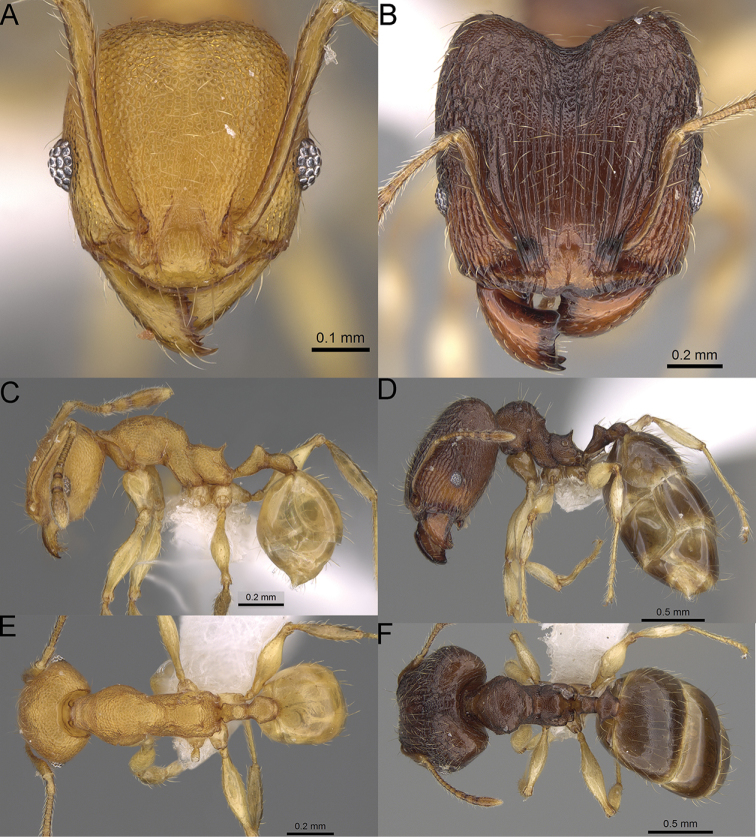
Pheidole bemarahaensis sp. nov., full-face view (**A**), profile (**C**), and dorsal view (**E**) of paratype minor worker (CASENT0425239) and full-face view (**B**), profile (**D**), and dorsal view (**F**) of holotype major worker (CASENT0425236).

Figs 61A–F , 84G , 86G

###### Type material.

***Holotype*.** Madagascar. •1 major worker; Mahajanga; Parc National Tsingy de Bemaraha, 2.5 km 62°ENE Bekopaka, Ankidrodroa River; -19.13222, 44.81467; alt. 100 m; 15 Nov 2001; B.L. Fisher et al. leg.; BLF04388, CASENT0425236, bottom specimen (CASC). ***Paratypes*.** Madagascar. •7w., 1s.; same data as for holotype; CASENT0425237–CASENT0425239, CASENT0872078, CASENT0872169–CASENT0872172 (CASC).

###### Diagnosis.

***Major workers*.** Small species: HL: 1.05–1.04, HW: 1.01–0.98, WL: 0.76–0.69; head in full-face view square, anterior and posterior sides of eyes slightly convex; in lateral view sub-oval; ventral and dorsal faces convex; dorsal face not depressed posteriorly; sides of the head with sparse, relatively long, erect pilosity; frons with dense, thick, and longitudinal rugae, interspaces smooth, only posterior part of frons finely foveolate; inner hypostomal teeth distinct, closely spaced, triangular with relatively narrow base, apex rounded; outer hypostomal teeth slightly higher than inner hypostomal teeth, and with wider base, triangular; gaster indistinctly shagreened; body brown to dark brown. ***Minor workers*.** Head and mesosoma foveolate; promesonotum low, long, slightly convex, with relatively steep posterior declivity; propodeal spines moderately long.

###### Description.

**Major workers.** Measurements (*N* = 2): HL: 1.05–1.04; HW: 1.01–0.98; SL: 0.49–0.51; EL: 0.14–0.14; WL: 0.76–0.69; PSL: 0.15–0.15; MTL: 0.49–0.46; PNW: 0.45–0.45; PTW: 0.11–0.12; PPW: 0.32–0.33; CI: 95.5–94.2; SI: 49.0–51.9; PSLI: 14.3–14.6; PPI: 34.5–34.5; PNI: 44.9–46.3; MTI: 48.6–47.4. ***Head*.** In full-face view square, anterior and posterior sides of eyes slightly convex (Fig. 61B). In lateral view sub-oval; ventral and dorsal faces convex; dorsal face not depressed posteriorly; inner hypostomal teeth visible. Sides of the head with sparse, relatively long, erect pilosity; whole head with dense, short, suberect to erect pilosity. Antennal scrobes very indistinct and not delimited by carinulae; scrobe surface shiny, finely foveolate with sparse, thick, longitudinal to irregular in posterior part rugulae. Occipital lobes shiny, with fine foveolae and distinct, sparse, irregular rugae, sculpture not weakening posteriorly; frons and malar area with dense, thick, and longitudinal rugae, interspaces smooth, and only posterior part of frons finely foveolate; genae shiny, with fine rugo-foveolae. Centre of clypeus smooth and shiny, lateral sides with longitudinal rugae; median notch present, narrow, and shallow; median longitudinal carina present; lateral longitudinal carinae present. Scape, when laid back, slightly exceeding the midlength of head; pilosity suberect to erect (Fig. 61B, D). Inner hypostomal teeth distinct, closely spaced, triangular with relatively narrow base, apex rounded; outer hypostomal teeth slightly higher than outer hypostomal teeth, and with wider base, triangular (Fig. 84G). ***Mesosoma*.** In lateral view, promesonotum moderately short, relatively low and convex, posterior mesonotum steep, with small tubercle-like projections; promesonotal groove absent; metanotal groove absent; propodeal spines small, triangular, with rounded apex; humeral area laterally well produced (Fig. 61D). Surface shiny, with fine but distinct foveolae, additional sparse transverse to irregular rugae on promesonotal dorsum, sculpture slightly weakening on dorsal surface. Pilosity sparse, long, and erect (Fig. 61D, F). ***Petiole*.** Shiny and with fine foveolae; peduncle short, with indistinct horizontal lobes on its basal part; node relatively high, triangular, with rounded apex, in rear view node slightly convex; pilosity moderately sparse and erect (Fig. 61D, F). ***Postpetiole*.** Shiny, with fine and sparse foveolae, smooth on dorsum; in dorsal view sides with short, acute, and triangular projections; pilosity long, moderately long, and erect (Fig. 61D, F). ***Petiole*.** Shiny and indistinctly shagreened; pilosity sparse, short, and erect (Fig. 61D, F). ***Colour*.** Unicolourous, brown to dark brown; malar area and lower frons bright brown; legs yellow (Fig. 61D, F).

**Minor workers.** Measurements (*N* = 7): HL: 0.48–0.51 (0.49); HW: 0.43–0.47 (0.45); SL: 0.42–0.46 (0.45); EL: 0.1–0.11 (0.1); WL: 0.56–0.59 (0.58); PSL: 0.09–0.11 (0.1); MTL: 0.32–0.37 (0.35); PNW: 0.28–0.31 (0.29); PTW: 0.06–0.08 (0.07); PPW: 0.12–0.13 (0.12); CI: 88.8–93.6 (91.1); SI: 98.1–105.1 (100.2); PSLI: 17.9–21.0 (19.8); PPI: 53.4–61.4 (56.3); PNI: 64.1–65.9 (65.2); MTI: 72.5–80.4 (77.1). ***Head*.** Occipital margin straight or indistinctly concave; occipital carina absent (Fig. 61A). Pilosity sparse, short, suberect to erect. Head foveolate. Clypeus with fine and sometimes reduced foveolae and rugae; median longitudinal carina absent; two lateral longitudinal carinae absent. Scape, when laid back, surpassing the posterior head margin by one-fifth of its length; pilosity suberect to erect (Fig. 61A, C). ***Mesosoma*.** In lateral view, promesonotum low, long, slightly convex, with relatively steep posterior declivity; promesonotal groove absent; metanotal groove present; propodeal spines moderately long, triangular, apex acute (Fig. 61C). Sculpture foveolate. Pilosity sparse, short, and erect (Fig. 61C, E). ***Petiole*.** Shiny and with fine foveolae; peduncle short and thin with ventral face slightly convex; node moderately high, triangular, and small; with few short, erect setae (Fig. 61C, E). ***Postpetiole*.** Short, low, and slightly convex; with few short, erect setae (Fig. 61C, E). ***Petiole*.** With moderately sparse, erect pilosity (Fig. 61C, E). ***Colour*.** Unicolourous, yellow (Fig. 61C, E).

###### Etymology.

From the type locality.

###### Biology.

The species was collected at 100 m in elevation, in tropical dry forest. Nest was located in a rotten log.

###### Comments.

*Pheidole bemarahaensis* sp. nov. is most similar to *P. bemarivoensis* sp. nov. ***Major workers*.***Pheidole bemarahaensis* sp. nov. differs from *P. bemarivoensis* sp. nov. in at least lower part of frons smooth between rugae, more distinct rugo-foveolate sculpture on genae, and thicker dorsal surface of promesonotum. ***Minor workers*.***P. bemarahaensis* sp. nov. differs from *P. bemarivoensis* sp. nov. in moderately long propodeal spines.

#### Revision of the *Pheidole petax* group

**Diagnosis. *Major workers*.** Head in full-face view rectangular or square (except *P. boribora*), anterior and posterior of eyes relatively straight, in lateral view sub-rectangular, ventral and dorsal faces finely convex or flat and dorsal face finely depressed posteriorly; antennal scrobes shallowly impressed and not delimited (except *P. ankerana* complex); occipital lobes with thick, sparse, irregular rugae; frons with thick, sparse to moderately sparse, longitudinal rugae; antennal scrobes with finely foveolate interspaces (except *P. glabra* sp. nov. and *P. ankerana* complex); promesonotum relatively low and angular; propodeal spines small to moderately long, with wide base; body yellowish brown to black. ***Minor workers*.** Head foveolate, sometimes with few additional rugae; genae and frons sometimes with smooth notches; scape short, when laid back, surpassing the posterior head margin by one- to two-fifths of its length; promesonotum slightly convex, with relatively steep posterior declivity; mesosoma foveolate, sometimes with smooth notches on the lateral sides; propodeal spines minute to relatively long, triangular or narrow; body yellow to yellowish brown (except *P. brevipilosa* complex).

**Comments.** Major workers of this group can be separated by the combination of the following characters: rectangular or square head in full-face view (except *P. boribora*) in lateral view sub-rectangular, shallowly impressed antennal scrobes usually not delimited by carinulae (except *P. ankerana* complex), thick, sparse, irregular rugae on occipital lobes and frons always with thick longitudinal rugae; foveolate antennal scrobes (except *P. glabra* sp. nov. and *P. ankerana* complex); relatively low and angular promesonotum and yellowish brown to black body. Minor workers can be separated based on foveolate head, sometimes with few additional rugae and smooth notches on genae and frons, short scape which, when laid back, surpass the posterior head margin by one- to two-fifths of its length, foveolate mesosoma, sometimes with smooth notches on the lateral sides and yellow to yellowish brown body (except *P. brevipilosa* complex).

The group consists of five complexes. *Pheidole petax* Forel creates a single species complex common across evergreen forests and central highlands, and is sympatric with all members of the group. The *P. brevipilosa* complex contains two species: *P. brevipilosa* sp. nov. and *P. glabra* sp. nov. *Pheidole brevipilosa* sp. nov. is known from two localities: Réserve Spéciale Marotandrano, Mahajanga and Forêt de Petriky, Toliara. While *P. glabra* sp. nov. is known from the area surrounding Tolagnaro, Toliara. The *P. mavesatra* complex contains two species: *P. mavesatra* sp. nov., and *P. goavana* sp. nov. distributed in the northern part of the island. *Pheidole mavesatra* sp. nov. is known only from its type locality (Tampolo, Toamasina), and *P. goavana* is distributed exclusively in the Antsiranana prefecture, in the area spread between Ambalabe and Antisiranana. The *P. ankerana* complex contains two species: *P. ankerana* sp. nov. known from two localities: Ankerana and Parc National de Zahamena, Toamasina, and *P. vatovavensis* sp. nov. so far recorded only from Forêt Classée Vatovavy, Fianarantsoa. Finally, the *P. boribora* complex contains two species known only from their type localities: *P. boribora* sp. nov. and *P. miramila* sp. nov. *Pheidole boribora* sp. nov. was described from Parc National d’Andohahela, Toliara, and *P. miramila* sp. nov. from Ambalavao, Fianarantsoa.

##### Key to the *Pheidole petax* group

**Table:** 

1	Major workers. Head in full-face view sub-oval; antennal scrobes distinctly foveolate with very fine thin, sparse, longitudinally or irregularly rugoreticulate (Fig. 62A, B). Minor workers. Head foveolate with no additional sculpture; mesosoma foveolate with katepisternum, anepisternum and mesonotum smooth; body yellow (Fig. 73A, C, E)	**2**
–	Major workers. Head in full-face view rectangular or square; antennal scrobes with dense, thin to thick, most often longitudinal, rugulae and finely foveolate to rugo-foveolate interspaces (Figs 63A, C, 64A, B, 65A, B). Minor workers. Character combination different (Figs 63B, D–F, 64G–J, 65C, D)	**3**
2	Major workers. Head with frons and antennal scrobes foveolate; sides of head with moderately dense, long, erect pilosity; inner hypostomal teeth dentate; propodeal spines small (Fig. 62A, C, E)	***P. boribora* sp. nov.**
–	Major workers. Head with frons and antennal scrobes never foveolate; sides of head with sparse, short, erect pilosity; inner hypostomal teeth triangular; propodeal spines moderately long (Fig. 62B, D, F)	***P. miramila* sp. nov.**
3	Major workers. Sides of the head with moderately dense to dense, long, suberect to erect pilosity; inner and outer teeth closely spaced and connected by concavity; body yellowish to reddish brown; genae with smooth notch (Fig. 63A, C, G–H). Minor workers. Head foveolate and sometimes with additional thin rugae, mesosoma foveolate with additional thick rugulae or foveolate with moderately long propodeal spines, body yellow (Fig. 63B, D–F)	**4**
–	Major workers. Sides of the head with sparse to moderately dense, short to moderately long, decumbent to suberect pilosity; inner and outer teeth not closely spaced and never connected by concavity; body bright brown to black; genae never with smooth notch (Figs 64A, B, 65A, B). Minor workers. Head foveolate with reduced sculpture on genae and mesosoma foveolate without additional rugulae (if additional rugae occurs then body never yellow), propodeal spines small (Figs 64G–J, 65C, D)	**5**
4	Major workers. Pilosity of head sides dense and long, frons with area between rugae smooth, antennal scrobes never foveolate, inner hypostomal teeth very low, lobe-like (Fig. 63A, G). Minor workers. Head and mesosoma foveolate with additional thin, sparse rugae, propodeal spines minute (Fig. 63B, E)	***P. ankerana* sp. nov.**
–	Major workers. Pilosity of head sides sparser and shorter, frons with area between rugae never smooth, antennal scrobes foveolate, inner hypostomal teeth low, triangular (Fig. 63C, H). Minor workers. Head and mesosoma foveolate and never with additional rugae, propodeal spines relatively long (Fig. 63D, F)	***P. vatovavensis* sp. nov.**
5	Major workers. Head in full-face view square; body brownish black to black (Fig. 64A–D). Minor workers. Head foveolate with few additional, indistinct, longitudinal rugae on frons and malar area; body dark brown (Fig. 64G–J)	**6**
–	Major workers. Head in full-face view rectangular; body brown to brownish black (Fig. 65A, B). Minor workers. Head foveolate without additional sculpture; body yellow to dark yellow (Fig. 65C, D)	**7**
6	Major workers. Head with foveolate sculpture, outer hypostomal teeth small and thin, with rounded tips directed outward, pronotum and propodeum without smooth notches (Fig. 64A, C, E). Minor workers. Head pilosity short and sparse, promesonotum with additional irregular rugae, frons and dorsal surface of pronotum with never reduced or smooth sculpture (Fig. 64G, I)	***P. brevipilosa* sp. nov.**
–	Major workers. Head never with foveolate sculpture, outer hypostomal teeth well developed, with rounded tips never directed outward, pronotum and propodeum with smooth notches (Fig. 64B, D, F). Minor workers. Head pilosity long and dense, promesonotum without additional irregular rugae, frons and dorsal surface of pronotum with reduced or smooth sculpture (Fig. 64H, J)	***P. glabra* sp. nov.**
7	Major workers. Sides of the head with sparse, short, decumbent pilosity; propodeal spines small (Fig. 66B, D). Minor workers. Promesonotum short, slightly convex, with relatively steep posterior declivity (Fig. 66C)	***P. petax* Forel**
–	Major workers. Sides of the head with sparse, relatively long, suberect pilosity; propodeal spines moderately long (Fig. 65A, B). Minor workers. Promesonotum low, long, relatively flat or slightly convex, with steep posterior declivity (Fig. 65C, D)	**8**
8	Major workers. Body brown to dark brown; katepisternum smooth; promesonotum and lateral surfaces of propodeum with additional thick, sparse and irregular rugae (Fig. 65A). Minor workers. Promesonotum low, long and relatively flat; propodeal spines narrow (Fig. 65C)	***P. mavesatra* sp. nov.**
–	Major workers. Body brownish black; katepisternum never smooth; promesonotum and lateral surfaces of propodeum with additional thin, moderately dense rugoreticulation (Fig. 65B). Minor workers. Promesonotum low, long, and slightly convex; propodeal spines triangular (Fig. 65D)	***P. goavana* sp. nov.**

**Figure 62.**
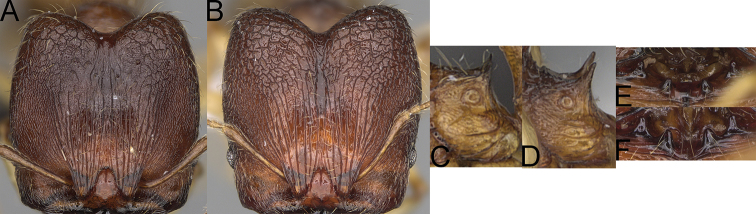
*Pheidole boribora* sp. nov., head of major worker (**A**), propodeal spines of major worker (**C**), hypostomal teeth (**E**). *Pheidole miramila* sp. nov., head of major worker (**B**), propodeal spines of major worker (**D**), hypostomal teeth (**F**).

**Figure 63.**
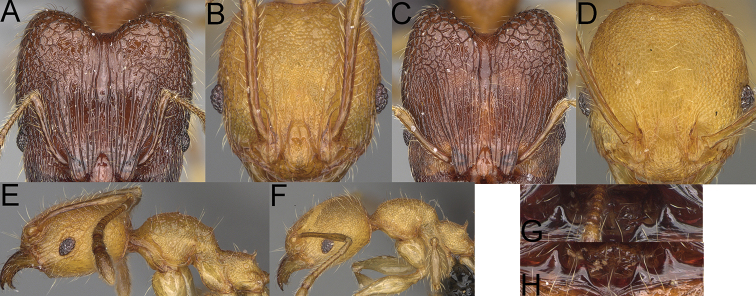
*Pheidole ankerana* sp. nov., head of major worker (**A**), head of minor worker (**B**), profile of minor worker (**E**), hypostomal teeth (**G**). *Pheidole vatovavensis* sp. nov., head of major worker (**C**), head of minor worker (**D**), profile of minor worker (**F**), hypostomal teeth (**H**).

**Figure 64.**
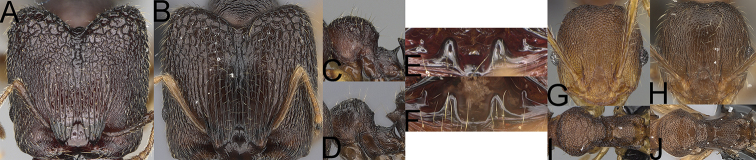
*Pheidole brevipilosa* sp. nov., head of major worker (**A**), profile of major worker (**C**), hypostomal teeth (**E**), head of minor worker (**G**), dorsum of mesosoma of minor worker (**I**). *Pheidole glabra* sp. nov., head of major worker (**B)**, profile of major worker (**D**), hypostomal teeth (**F**), head of minor worker (**H**), dorsum of mesosoma of minor worker (**J**).

**Figure 65.**
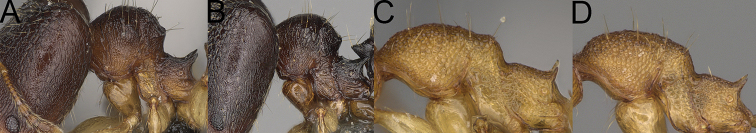
Profile. *Pheidole mavesatra* sp. nov., major worker (**A**), minor worker (**C**). *Pheidole goavana* sp. nov., major worker (**B**), minor worker (**D**).

**Figure 66.**
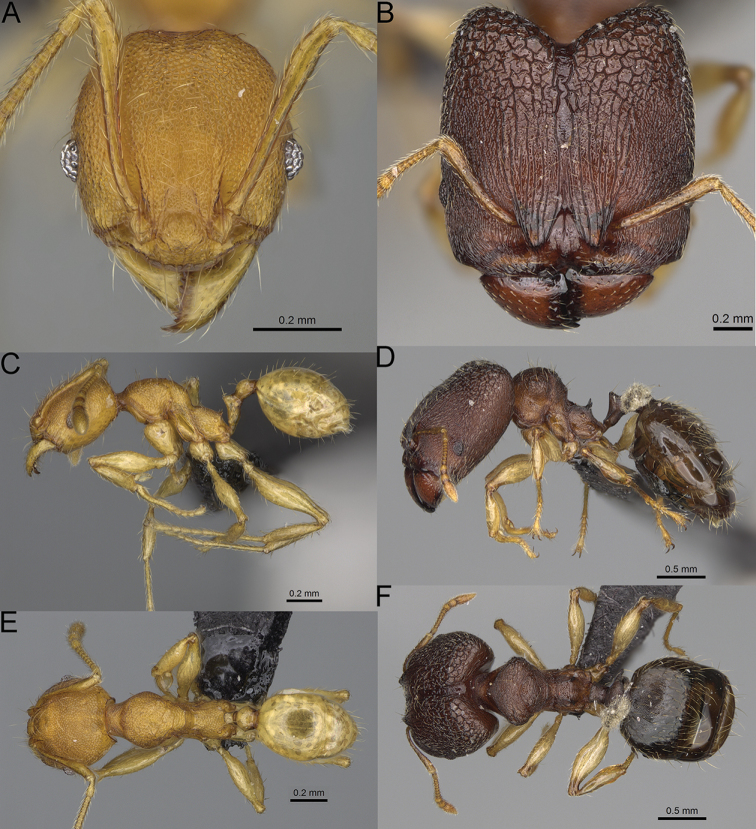
*Pheidole petax* Forel, full-face view (**A**), profile (**C**), and dorsal view (**E**) of minor worker (CASENT0455969) and full-face view (**B**), profile (**D**), and dorsal view (**F**) of major worker (CASENT0119429).

#### Revision of the *Pheidole petax* complex

**Diagnosis. *Major workers*.** Head in full-face view rectangular, anterior and posterior of eyes relatively straight, in lateral view sub-rectangular, ventral and dorsal faces finely convex; sides of the head with sparse, short, decumbent pilosity; occipital lobes with interspaces smooth; frons with interspaces smooth to finely foveolate; antennal scrobes with dense, fine rugulae, and sometimes with additional sparse and fine, longitudinal rugae; promesonotal and metanotal grooves absent; propodeal spines small, with wide base; pronotum with sparse, irregular, thin rugae, interspaces smooth to finely foveolate; katepisternum smooth; first gastral tergite shagreened; body brown to dark brown. ***Minor workers*.** Head foveolate, genae with smooth notches; scape, when laid back, surpassing the posterior head margin by two-fifths of its length; promesonotum slightly convex, with relatively steep posterior declivity; mesosoma foveolate, katepisternum and, sometimes, lateral sides of propodeum smooth; promesonotal groove absent; metanotal groove indistinct; propodeal spines very short and triangular.

**Comments.** Major workers of this complex can be distinguished based on a compilation of the following characters: head in full-face view rectangular and in lateral view sub-rectangular; sides of the head with sparse, short, and decumbent pilosity; occipital lobes with thick and irregular rugae with smooth interspaces; frons with thick and longitudinal rugae and smooth to finely foveolate interspaces; small propodeal spines with wide base; smooth katepisternum; shagreened first gastral tergite, and brown to dark brown body. Minor workers can be separated based on foveolate head and mesosoma, with smooth katepisternum and genae with smooth notches; promesonotum with relatively steep posterior declivity; very short propodeal spines and yellowish-brown body.

This complex complies only one species, *P. petax* Forel.

##### Pheidole petax

Taxon classificationAnimaliaHymenopteraFormicidae

Forel, 1895 stat. nov.

Figs 66A–F , 85Q , 88A

###### Type material.

*Pheidole nemoralis petax* Forel, 1895b: 488 (s.w.). Lectotype [designated here]: major worker (CASENT0101321): Madagascar, Central Madagascar, coll. Sikora (MHNG) [examined]. Paralectotype: 1 minor worker (the same pin as lectotype, CASENT0101322) (MHNG) [examined]: the same data as lectotype.

###### Other material.

Madagascar. –***Antananarivo***: •1w.; Manakambahiny; -18.93217, 47.53617; 20 Jan 2004; Ranaivo leg.; CASENT0008742 (CASC). –***Fianarantsoa***: •2w., 2s.; 2 km W Andrambovato, along river Tatamaly; -21.51167, 47.41; alt. 1075 m; 3 Jun 2005; B.L. Fisher et al. leg.; CASENT0060954, CASENT0060955 (CASC). •1w., 2s.; 43 km S Ambalavao, Rés. Andringitra; -22.23333, 47; alt. 825 m; 5 Oct 1993; B.L. Fisher leg.; CASENT0198401, CASENT0198900 (CASC). •6w., 9s., 3q.; 45 km S. Ambalavao; -22.21667, 47.01667; alt. 785 m; 25 Sep 1993; B.L. Fisher leg.; CASENT0003649, CASENT0003658, CASENT0198402, CASENT0198404, CASENT0198405, CASENT0198407–CASENT0198410 (CASC). •2w., 3s.; 9.0 km NE Ivohibe; -22.42667, 46.93833; alt. 900 m; 12 Nov 1997; B.L. Fisher et al. leg.; CASENT0198412, CASENT0198893, CASENT0198895 (CASC). •4w., 5s.; Forêt de Vevembe, 66.6 km 293° Farafangana; -22.791, 47.18183; alt. 600 m; 24 Apr 2006; B.L. Fisher et al. leg.; CASENT0070685, CASENT0070700, CASENT0070758, CASENT0070826, CASENT0108007 (CASC). •7w., 8s.; Parc National Befotaka-Midongy, Papango 27.7 km S Midongy-Sud, Mount Papango; -23.83517, 46.96367; alt. 940 m; 15 Nov 2006; B.L. Fisher et al. leg.; CASENT0119130, CASENT0119132, CASENT0119429, CASENT0119430, CASENT0119434, CASENT0119448, CASENT0119658 (CASC). •1w.; Parc National Befotaka-Midongy, Papango 28.5 km S Midongy-Sud, Mount Papango; -23.84083, 46.9575; alt. 1250 m; 17 Nov 2006; B.L. Fisher et al. leg.; CASENT0128568 (CASC). –***Mahajanga***: •2w., 2s.; Réserve Spéciale Marotandrano, Marotandrano 48.3 km S Mandritsara; -16.28322, 48.81443; alt. 865 m; 7 Dec 2007; B.L. Fisher et al. leg.; CASENT0134250, CASENT0136553 (CASC). –***Toamasina***: •1w.; Ambatovy, 12.4 km NE Moramanga; -18.84773, 48.29568; alt. 1000 m; 5 Mar 2007; B.L. Fisher et al. leg.; CASENT0121407 (CASC). •3w., 1s.; Ambatovy, 12.4 km NE Moramanga; -18.84963, 48.2947; alt. 1010 m; 3 Mar 2007; B.L. Fisher et al. leg.; CASENT0114818, CASENT0124520, CASENT0124563, CASENT0124590 (CASC). •1w.; Ambatovy, 12.4 km NE Moramanga; -18.85813, 48.28488; alt. 1040 m; 5 Mar 2007; B.L. Fisher et al. leg.; CASENT0124202 (CASC). •2w., 3s.; Ambatovy, 12.4 km NE Moramanga; -18.83937, 48.30842; alt. 1080 m; 4 Mar 2007; B.L. Fisher et al. leg.; CASENT0121207, CASENT0121278, CASENT0123810, CASENT0123851, CASENT0123885 (CASC). •7w., 3s.; Analamay; -18.80623, 48.33707; alt. 1068 m; 21 Mar 2004; B.L. Fisher et al. leg.; CASENT0046552, CASENT0048445, CASENT0049051, CASENT0050529 (CASC). •2w.; Ankerana; -18.4061, 48.82029; alt. 725 m; 16 Jan 2012; B.L. Fisher et al. leg.; CASENT0274776 (CASC). •12w.; Ankerana; -18.40062, 48.81311; alt. 865 m; 17 Jan 2012; B.L. Fisher et al. leg.; CASENT0274711, CASENT0274846, CASENT0274847, CASENT0274900, CASENT0274906, CASENT0274911 (CASC). •6w., 4s.; Bevolota 17.1 km N Andasibe; -18.77071, 48.43164; alt. 995 m; 12 Dec 2007; B.L. Fisher et al. leg.; CASENT0135107, CASENT0135159, CASENT0135175, CASENT0135176, CASENT0135178, CASENT0135200 (CASC). •1w., 1s.; Corridor Forestier Analamay-Mantadia, Ambatoharanana; -18.79956, 48.4028; alt. 1058 m; 12 Dec 2012; B.L. Fisher et al. leg.; CASENT0300462 (CASC). •3w., 2s., 1q.; Corridor Forestier Analamay-Mantadia, Ambatoharanana; -18.80398, 48.40358; alt. 1064 m; 12 Dec 2012; B.L. Fisher et al. leg.; CASENT0300405, CASENT0300406, CASENT0302207, CASENT0302209 (CASC). •1w., 2s.; Corridor Forestier Analamay-Mantadia, Ambatoharanana; -18.80424, 48.40081; alt. 968 m; 12 Dec 2012; B.L. Fisher et al. leg.; CASENT0291231, CASENT0301825 (CASC). •4w., 2s., 2q.; Corridor Forestier Analamay-Mantadia, Ambohibolakely; -18.77908, 48.36628; alt. 1014 m; 28 Nov 2012; B.L. Fisher et al. leg.; CASENT0300215–CASENT0300218 (CASC). •4w., 3s., 1q.; Corridor Forestier Analamay-Mantadia, Ambohibolakely; -18.76087, 48.37128; alt. 1044 m; 29 Nov 2012; B.L. Fisher et al. leg.; CASENT0296961, CASENT0300327, CASENT0300328 (CASC). •2w., 2s.; Corridor Forestier Analamay-Mantadia, Ambohibolakely; -18.76131, 48.36437; alt. 983 m; 23 Nov 2012; B.L. Fisher et al. leg.; CASENT0299219, CASENT0302058, CASENT0302061 (CASC). •3w., 2s., 1q.; Corridor Forestier Analamay-Mantadia, Tsaravoniana; -18.76465, 48.41938; alt. 1039 m; 5 Dec 2012; B.L. Fisher et al. leg.; CASENT0299298, CASENT0299299, CASENT0300801 (CASC). •5w., 3s., 2q.; Corridor Forestier Analamay-Mantadia, Tsaravoniana; -18.76124, 48.42134; alt. 939 m; 3 Dec 2012; B.L. Fisher et al. leg.; CASENT0297036, CASENT0297037, CASENT0297045, CASENT0297046, CASENT0297048 (CASC). •1s.; Corridor Forestier Analamay-Mantadia, Tsaravoniana; -18.76369, 48.4203; alt. 984 m; 2 Dec 2012; B.L. Fisher et al. leg.; CASENT0299402 (CASC). •15w., 8s., 1q.; Forêt Ambatovy, 14.3 km 57° Moramanga; -18.85083, 48.32; alt. 1075 m; 21 Mar 2004; B.L. Fisher et al. leg.; CASENT0047075, CASENT0047557, CASENT0048999, CASENT0050389, CASENT0050391, CASENT0053643, CASENT0053644, CASENT0053735, CASENT0058852, CASENT0058895, CASENT0058896, CASENT0058907, CASENT0058908 (CASC). •5w., 7s., 1m.; Montagne d’Akirindro 7.6 km 341°NNW Ambinanitelo; -15.28833, 49.54833; alt. 600 m; 17 Mar 2003; B.L. Fisher et al. leg.; CASENT0039193, CASENT0496357, CASENT0496447, CASENT0496449, CASENT0496462, CASENT0496464 (CASC). •1w.; Parc National d´ Andasibe-Mantadia, Forêt de Mantadia, 25.7 km 248°Moramanga; -18.81402, 48.43028; alt. 1040 m; 14 Jul 2006; Raharimalala & Blaimer leg.; CASENT0117457 (CASC). •9w., 3s., 4q.; Parc National de Zahamena, Besaky River; -17.75244, 48.85321; alt. 760 m; 22 Feb 2009; B.L. Fisher et al. leg.; CASENT0149781, CASENT0149784, CASENT0149785, CASENT0152048, CASENT0152049, CASENT0152310, CASENT0152311, CASENT0152344, CASENT0152345, CASENT0152346, CASENT0217995 (CASC). •5w., 4w., 1q.; Parc National de Zahamena, Onibe River; -17.75908, 48.85468; alt. 780 m; 22 Feb 2009; B.L. Fisher et al. leg.; CASENT0152064, CASENT0152117, CASENT0152118, CASENT0152159, CASENT0153317, CASENT0153410 (CASC). •3w., 3s.; Parc National de Zahamena, Sahavorondrano River; -17.75257, 48.85725; alt. 765 m; 23 Feb 2009; B.L. Fisher et al. leg.; CASENT0150629, CASENT0150630, CASENT0150636 (CASC). •1w., 1s.; Reserve Betampona, Camp Rendrirendry 34.1 km 332° Toamasina; -17.924, 49.19967; alt. 390 m; 30 Nov 2005; B.L. Fisher et al. leg.; CASENT0067809 (CASC). •4w., 2s., 1q., 1m.; Réserve Spéciale Ambatovaky, Sandrangato River; -16.81745, 49.2925; alt. 400 m; 26 Feb 2010; B.L. Fisher et al. leg.; CASENT0160460, CASENT0162149, CASENT0162150, CASENT0162210 (CASC). •3w., 3s.; Réserve Spéciale Ambatovaky, Sandrangato River; -16.8162, 49.29202; alt. 425 m; 25 Feb 2010; B.L. Fisher et al. leg.; CASENT0161882, CASENT0161897, CASENT0161898 (CASC). •2w.; Réserve Spéciale Ambatovaky, Sandrangato River; -16.80561, 49.29507; alt. 480 m; 27 Feb 2010; B.L. Fisher et al. leg.; CASENT0163058 (CASC). •1w., 1s.; Réserve Spéciale Ambatovaky, Sandrangato River; -16.7674, 49.26813; alt. 500 m; 23 Feb 2010; B.L. Fisher et al. leg.; CASENT0161932 (CASC). •7w., 1q., 1m.; Réserve Spéciale Ambatovaky, Sandrangato River; -16.7633, 49.26692; alt. 520 m; 22 Feb 2010; B.L. Fisher et al. leg.; CASENT0160490, CASENT0162105, CASENT0162981, CASENT0163830, CASENT0163876, CASENT0163938, CASENT0163983 (CASC). •1s; Station forestière Analamazaotra, Analamazaotra 1.3 km S Andasibe; -18.38466, 48.41271; alt. 980 m; 11 Dec 2007; B.L. Fisher et al. leg.; CASENT0139899 (CASC). •8w., 5s.; Torotorofotsy; -18.87082, 48.34737; alt. 1070 m; 24 Mar 2004; B.L. Fisher et al. leg.; CASENT0048136, CASENT0048382, CASENT0048408, CASENT0048467, CASENT0049030, CASENT0051147, CASENT0051149 (CASC).

###### Diagnosis.

***Major workers*.** Head in full-face view rectangular, anterior and posterior of eyes relatively straight; sides of head with sparse, short, decumbent pilosity; scrobe surface with dense, fine rugulae and sometimes with additional sparse and fine, longitudinal rugae, interspaces and rugulae finely foveolate; inner hypostomal teeth distinct, big, thick, triangular, with rounded apex; outer hypostomal teeth approximately as high as inner hypostomal teeth but thinner, triangular, with rounded tips and wide base; first gastral tergite shagreened, at least on its basal part; body brown to dark brown. ***Minor workers*.** Head foveolate, only genae with smooth notches, covering at least their central part; promesonotum slightly convex, with relatively steep posterior declivity; mesosoma foveolate, only katepisternum and sometimes lateral sides of propodeum smooth; propodeal spines very short and triangular; body yellowish brown.

###### Redescription.

**Major workers.** Measurements (*N* = 10): HL: 1.37–1.6 (1.52); HW: 1.22–1.48 (1.4); SL: 0.56–0.64 (0.59); EL: 0.13–0.19 (0.16); WL: 1.08–1.24 (1.17); PSL: 0.21–0.24 (0.23); MTL: 0.57–0.66 (0.61); PNW: 0.72–0.88 (0.79); PTW: 0.18–0.25 (0.21); PPW: 0.61–0.75 (0.69); CI: 88.8–96.0 (91.7); SI: 40.3–45.6 (42.5); PSLI: 14.1–16.1 (15.0); PPI: 28.5–34.0 (30.7); PNI: 53.3–59.6 (56.7); MTI: 40.5–46.8 (43.9). ***Head*.** In full-face view rectangular, anterior and posterior of eyes relatively straight (Fig. 66B). In lateral view sub-rectangular; ventral and dorsal faces finely convex; dorsal face finely depressed posteriorly, forming shallow transverse depression between frons and occipital lobes; inner hypostomal teeth visible. Sides of head with sparse, short, decumbent pilosity; whole head with moderately dense, short, decumbent to erect pilosity. Antennal scrobes indistinct and not delimited by carinulae. Occipital lobes with thick, sparse, irregular rugae, interspaces smooth and shiny; frons with thick, sparse to moderately sparse, longitudinal rugae, interspaces variable, smooth to finely foveolate; malar area and lateral sides of head with dense, fine rugulae and sometimes with additional sparse and fine, longitudinal rugae, interspaces and rugulae finely foveolate; genae with very fine and dense rugulae, sometimes rugulae weakening posteriorly or absent. Clypeus shiny and smooth, with a few indistinct, longitudinal rugae on the lateral sides; median notch present, wide, and shallow; median longitudinal carina absent; lateral longitudinal carinae absent. Scape, when laid back, reaching slightly beyond midlength of head; pilosity decumbent to erect (Fig. 66B, D). Inner hypostomal teeth distinct, big, thick, triangular, with rounded apex; outer hypostomal teeth approximately as high as inner hypostomal teeth but thinner, triangular, with rounded tips and wide base (Fig. 85Q). ***Mesosoma*.** In lateral view, promesonotum short, angular and relatively low, posterior mesonotum convex or slightly steep, without projections; promesonotal groove absent; metanotal groove absent; propodeal spines small, with base wide, apex rounded; humeral area with wide and flat tubercles (Fig. 66D). Pronotum shiny, with sparse, irregular, thin rugae, interspaces smooth to finely foveolate; mesonotum and anepisternum finely foveolate; katepisternum smooth; propodeum finely foveolate and sometimes with additional rugae. Pilosity moderately dense, long, and erect (Fig. 66D, F). ***Petiole*.** Shiny; peduncle finely foveolate, without horizontal lobes on its basal part; node smooth, moderately high and narrow, with convex apex, in rear view node dorsoventrally concave; pilosity moderately dense and erect (Fig. 66D, F). ***Postpetiole*.** Finely foveolate and shiny; in dorsal view sides with moderately long, acute, narrow, and horn-like projections; pilosity long, moderately dense, and erect (Fig. 66D, F). ***Petiole*.** First gastral tergite shagreened, at least on its basal part; pilosity dense, moderately short, and erect (Fig. 66D, F). ***Colour*.** Brown to dark brown, legs yellow to bright brown (Fig. 66D, F).

**Minor workers.** Measurements (*N* = 10): HL: 0.53–0.58 (0.55); HW: 0.48–0.51 (0.49); SL: 0.47–0.53 (0.5); EL: 0.09–0.12 (0.11); WL: 0.6–0.69 (0.67); PSL: 0.07–0.09 (0.06); MTL: 0.37–0.44 (0.4); PNW: 0.29–0.36 (0.32); PTW: 0.05–0.08 (0.07); PPW: 0.11–0.16 (0.14); CI: 86.7–93.6 (89.5); SI: 98.2–103.8 (101.5); PSLI: 12.6–16.7 (14.4); PPI: 41.5–58.8 (49.4); PNI: 61.3–70.0 (65.8); MTI: 76.4–88.2 (82.2). ***Head*.** Occipital margin straight or indistinctly concave; occipital carina indistinct, weakly developed (Fig. 66A). Pilosity moderately dense, moderately long, decumbent to erect. Whole head foveolate, only genae with smooth notches covering at least their central part. Clypeus shiny, foveolate; median longitudinal carina present; two lateral longitudinal carinae absent. Scape, when laid back, surpassing the posterior head margin by two-fifths of its length; pilosity suberect to erect (Fig. 66A, C). ***Mesosoma*.** In lateral view, promesonotum slightly convex, with relatively steep posterior declivity; promesonotal groove absent; metanotal groove indistinct; propodeal spines very short and triangular, apex acute (Fig. 66C). Sculpture foveolate, only katepisternum and sometimes lateral sides of propodeum smooth. Pilosity moderately sparse, long, and erect (Fig. 66C, E). ***Petiole*.** Peduncle short and thin with ventral face slightly convex; node low, globular, and small; with few long, erect setae (Fig. 66C, E). ***Postpetiole*.** Short, low, and slightly convex; with few short, erect setae (Fig. 66C, E). ***Petiole*.** With sparse, erect pilosity (Fig. 66C, E). ***Colour*.** Unicolourous, yellowish brown (Fig. 66C, E).

###### Biology.

The species was collected between 64–1343 m in elevation, in rainforest, montane rainforest, transitional humid forest, an urban garden, and grassland. Nests were located in rotten logs and tree stumps, and in dead twigs above ground.

###### Comments.

This species in most similar to *P. brevipilosa* sp. nov. and *P. glabra* sp. nov. ***Major workers*.***Pheidole petax* can be distinguished from *P. brevipilosa* sp. nov. and *P. glabra* sp. nov. by never erect pilosity on sides of head, and more rectangular head shape in full-face view. ***Minor workers*.***Pheidole petax* can be distinguished from *P. brevipilosa* sp. nov. by yellowish brown body colouration, lack of additional indistinct, longitudinal rugae on frons and malar area, lack of additional sparse, irregular rugae on promesonotal dorsum, and foveolate clypeus; from *P. glabra* sp. nov. by yellowish brown body colouration, sparser pilosity on head, lack of additional indistinct, longitudinal rugae on frons and malar area, never reduced or absent sculpture on the central part of frons, and dorsal surface of pronotum with never reduced sculpture.

#### Revision of the *Pheidole brevipilosa* complex

**Diagnosis. *Major workers*.** Head in full-face view square, anterior and posterior of eyes relatively straight, in lateral view sub-rectangular; ventral and dorsal faces relatively flat; occipital lobes with interspaces smooth or superficially rugulose; frons with interspaces smooth to finely rugo-foveolate; promesonotal groove absent; metanotal groove absent or indistinct; propodeal spines moderately long, with base slightly wider than top; mesosoma with fine and sparse to dense rugoreticulation, katepisternum smooth, sometimes pronotum and propodeum partially smooth; first gastral tergite finely shagreened; body brownish black to black. ***Minor workers*.** Head foveolate, frons and malar area with few additional, indistinct, longitudinal rugae, genae and sometimes frons with weaker sculpture or smooth; scape, when laid back, surpassing the posterior head margin by one-fifth of its length; promesonotum low, slightly convex, with relatively steep posterior declivity; promesonotal groove absent; metanotal groove indistinct; propodeal spines very short, triangular; mesosoma foveolate; katepisternum smooth and promesonotal dorsum with additional sparse, irregular rugae or katepisternum, anepisternum, and parts of propodeum and pronotum smooth.

**Comments.** Major workers of this complex can be distinguished based on a combination of the following characters: head in full-face view square and in lateral view sub-rectangular; occipital lobes with thick and irregular rugae with smooth or superficially rugulae interspaces; frons with thick and longitudinal rugae with smooth to finely rugo-foveolate interspaces; moderately long propodeal spines, with base slightly wider than top; katepisternum, pronotum, and propodeum smooth or with reduced sculpture; finely shagreened first gastral tergite; and brownish black to black body. Minor workers can be separated based on the following characters: head foveolate with few additional, indistinct, longitudinal rugae on frons and malar area; genae and sometimes frons smooth or with reduced sculpture; very short and triangular propodeal spines; never entirely foveolate mesosoma; and dark brown body colouration.

##### Pheidole brevipilosa sp. nov.

Taxon classificationAnimaliaHymenopteraFormicidae

http://zoobank.org/505478A2-8A9C-4A1F-BD00-A5598BC3A5AE

**Figure 67.**
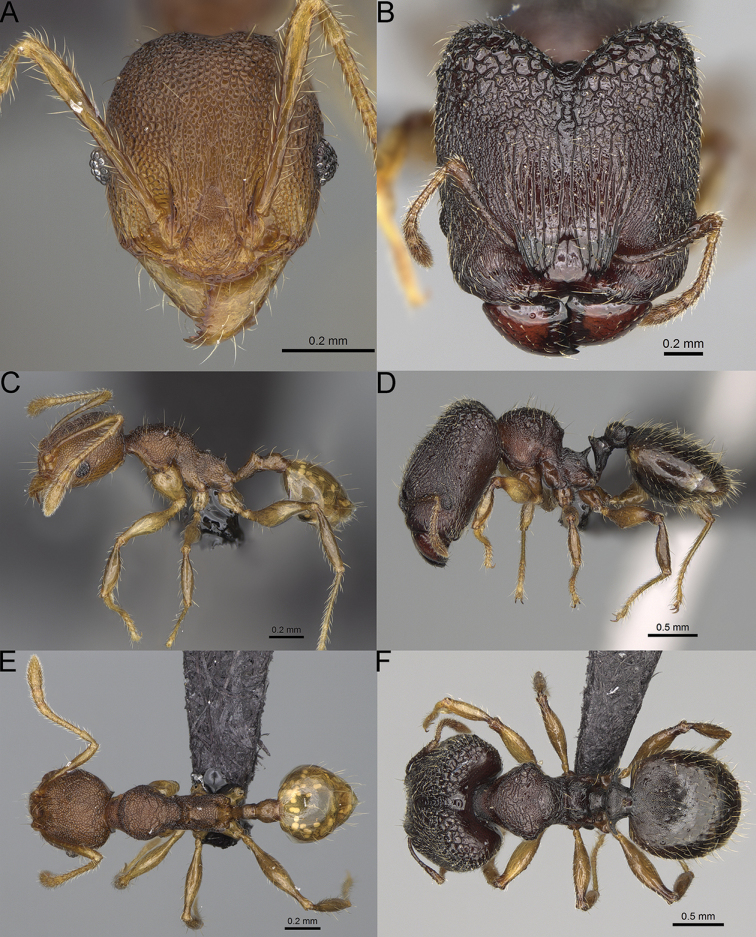
*Pheidole brevipilosa* sp. nov., full-face view (**A**), profile (**C**), and dorsal view (**E**) of paratype minor worker (CASENT0134271) and full-face view (**B**), profile (**D**), and dorsal view (**F**) of holotype major worker (CASENT0235034).

Figs 67A–F , 84K , 86K

###### Type material.

***Holotype*.** Madagascar. •1 major worker; Mahajanga; Réserve Spéciale Marotandrano, Marotandrano 48.3 km S Mandritsara; -16.28322, 48.81443; alt. 865 m; 7 Dec 2007; B.L. Fisher et al. leg.; BLF19163, CASENT0235034 (CASC). ***Paratype*.** Madagascar. •1 w.; same data as for holotype; CASENT0134271 (CASC).

###### Other material.

Madagascar. – ***Toliara***: •1s.; Forêt de Petriky, 12.5 km W 272° Tolagnaro; -25.06167, 46.87; alt. 10 m; 22 Nov 1998; B.L. Fisher leg.; CASENT0198889 (CASC).

###### Diagnosis.

***Major workers*.** Head in full-face view square, anterior and posterior of eyes relatively straight; dorsal face indistinctly depressed posteriorly; sides of the head with dense, short, erect pilosity; malar area, lateral sides of head, and genae with dense, fine rugulae and foveolae; inner hypostomal teeth distinct, big, thick, triangular, with rounded apex; outer hypostomal teeth small and thin, with rounded tips directed outward, lobe-like; propodeal spines moderately long, with base slightly wider than top; first gastral tergite shiny and finely shagreened; body brownish black to black. ***Minor workers*.** Whole head foveolate, frons and malar area with few additional, indistinct, longitudinal rugae, genae with weaker sculpture; promesonotum low, slightly convex, with relatively steep posterior declivity; mesosoma foveolate, only katepisternum smooth and promesonotal dorsum with additional sparse, irregular rugae; propodeal spines very small, triangular; body dark brown.

###### Description.

**Major workers.** Measurements (*N* = 2): HL: 1.29–1.49; HW: 1.26–1.41; SL: 0.58–0.61; EL: 0.15–0.17; WL: 0.93–1.22; PSL: 0.2–0.22; MTL: 0.54–0.64; PNW: 0.61–0.81; PTW: 0.18–0.21; PPW: 0.61–0.69; CI: 97.2–94.8; SI: 46.1–43.2; PSLI: 15.3–14.8; PPI: 29.3–30.0; PNI: 48.3–57.5; MTI: 42.9–45.8. ***Head*.** In full-face view square, anterior and posterior of eyes relatively straight (Fig. 67B). In lateral view sub-rectangular; ventral and dorsal faces relatively flat; inner hypostomal teeth visible. Sides of the head with dense, short, erect pilosity; whole head with dense, short, suberect to erect pilosity. Antennal scrobes indistinct and not delimited by carinulae. Occipital lobes with thick, sparse, irregular rugae, interspaces superficially rugulose; frons with thick, sparse to moderately sparse, longitudinal rugae, interspaces variable, smooth to finely rugulose and foveolae; malar area, lateral sides of head, and genae with dense, fine rugulae and foveolae. Clypeus shiny and smooth, with thin, longitudinal rugulae on the lateral sides; median notch present, wide and moderately deep; median longitudinal carina absent; lateral longitudinal carinae absent. Scape, when laid back, reaching the midlength of head; pilosity decumbent to erect (Fig. 67B, D). Inner hypostomal teeth distinct, big, thick, triangular, with rounded apex; outer hypostomal teeth small and thin, with rounded tips directed outward, lobe-like (Fig. 84K). ***Mesosoma*.** In lateral view, promesonotum short, angular and relatively low, posterior mesonotum relatively steep to steep, with indistinct, tubercle-like projections; promesonotal groove absent; metanotal groove indistinct; propodeal spines moderately long, with base slightly wider than top, apex rounded; humeral area with wide and flat tubercles (Fig. 67D). Surface shiny, with fine and dense rugoreticulation, only katepisternum smooth. Pilosity dense, moderately long, and erect (Fig. 67D, F). ***Petiole*.** Shiny, with dense and fine rugulae; peduncle short, with small, rounded, horizontal lobes on its basal part; node moderately high and narrow, with convex apex, in rear view node dorsoventrally concave; pilosity moderately dense and erect (Fig. 67D, F). ***Postpetiole*.** Shiny, with fine and dense rugulae; short and rounded; in dorsal view sides with long, narrow, acute, horn-like projections; pilosity long, moderately dense, and erect (Fig. 67D, F). ***Petiole*.** First gastral tergite shiny and finely shagreened; pilosity dense, moderately long, and erect (Fig. 67D, F). ***Colour*.** Unicolourous, brownish black to black; legs dark yellow to brown (Fig. 67D, F).

**Minor workers.** Measurements (*N* = 1): HL: 0.55; HW: 0.48; SL: 0.51; EL: 0.12; WL: 0.67; PSL: 0.08; MTL: 0.4; PNW: 0.33; PTW: 0.07; PPW: 0.14; CI: 87.2; SI: 105.6; PSLI: 15.2; PPI: 52.6; PNI: 68.3; MTI: 82.0. ***Head*.** Occipital margin straight or indistinctly concave; occipital carina indistinct, weakly developed (Fig. 67A). Pilosity moderately sparse, moderately long, and erect. Whole head foveolate, frons and malar area with few additional, indistinct, longitudinal rugae, genae with weaker sculpture. Clypeus shiny, rugoreticulate; median longitudinal carina absent; two lateral longitudinal carinae absent. Scape, when laid back, surpassing the posterior head margin by one-fifth of its length; pilosity erect (Fig. 67A, C). ***Mesosoma*.** In lateral view, promesonotum low, slightly convex, with relatively steep posterior declivity; promesonotal groove absent; metanotal groove indistinct; propodeal spines very small, triangular, apex acute (Fig. 67C). Sculpture foveolate, only katepisternum smooth and promesonotal dorsum with additional sparse, irregular rugae. Pilosity sparse, long, and erect (Fig. 67C, E). ***Petiole*.** Shiny; peduncle finely rugulose, short and thin with ventral face slightly convex; node low, triangular, and small; with few long, erect setae (Fig. 67C, E). ***Postpetiole*.** Short, low, and convex; with few short, erect setae (Fig. 67C, E). ***Petiole*.** With sparse, erect pilosity (Fig. 67C, E). ***Colour*.** Unicolourous, dark brown (Fig. 67C, E).

###### Etymology.

Latin for short setae, in reference to short setosity on the head sides in major workers.

###### Biology.

The species was collected between 10–467 m in elevation, in transition humid forest, rainforest, and littoral rainforest. Nests were located in rotten logs.

###### Comments.

This species in most similar to *P. petax* and *P. glabra* sp. nov. ***Major workers*.***Pheidole brevipilosa* sp. nov. can be distinguished from *P. petax* by presence of erect pilosity on sides of head and lateral sides of head, and square head shape in full-face view; from *P. glabra* sp. nov. by presence of foveolae sculpture on head, weakly developed outer hypostomal teeth, pronotum with sculpture never reduced and lacking smooth notches on its lateral or dorsal surfaces, and lateral sides of propodeum without smooth notches**. *Minor workers*.***Pheidole brevipilosa* sp. nov. can be distinguished from *P. petax* by dark brown body colouration, presence of additional indistinct, longitudinal rugae on frons and malar area, presence of additional sparse, irregular rugae on promesonotal dorsum, and clypeus never foveolate; from *P. glabra* sp. nov. by sparser and shorter pilosity on head, and frons, dorsal surface of pronotum, promesonotal declivity, and lateral surfaces of propodeum with never reduced sculpture.

##### Pheidole glabra sp. nov.

Taxon classificationAnimaliaHymenopteraFormicidae

http://zoobank.org/C54726DF-9759-447D-AC25-87D471335D43

**Figure 68.**
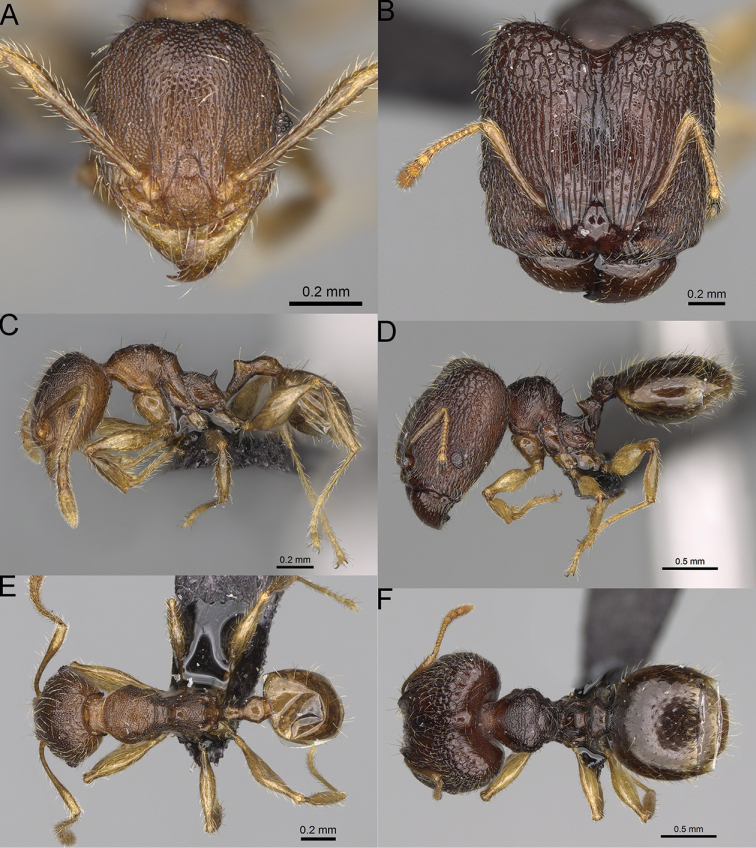
*Pheidole glabra* sp. nov., full-face view (**A**), profile (**C**), and dorsal view (**E**) of paratype minor worker (CASENT0235034) and full-face view (**B**), profile (**D**), and dorsal view (**F**) of holotype major worker (CASENT0134271).

Figs 68A–F , 84T , 86T

###### Type material.

***Holotype*.** Madagascar. •1 major worker; Toliara; Grand Lavasoa, 25.9 km W Tolagnaro; -25.08767, 46.749; alt. 450 m; 30 Nov 2006; B.L. Fisher et al. leg.; BLF15399, CASENT0122911 (CASC). ***Paratype*.** Madagascar. •1 w.; same data as for holotype; CASENT0235037 (CASC).

###### Other material.

Madagascar. – ***Toliara***: •1w., 1s.; Manatantely, 8.9 km NW Tolagnaro; -24.9815, 46.92567; alt. 100 m; 27 Nov 2006; B.L. Fisher et al. leg.; CASENT0125808, CASENT0125809 (CASC).

###### Diagnosis.

***Major workers*.** Head in full-face view square, anterior and posterior of eyes relatively straight; sides of the head with dense, short, erect pilosity; malar area with sparse to moderately dense longitudinal rugae, interspaces smooth to finely rugulose; inner hypostomal teeth distinct, big, thick, triangular, with rounded apex; outer hypostomal teeth slightly but distinctly smaller and thin, with rounded tips, and wide base; propodeal spines moderately long, with base slightly wider than top; first gastral tergite shiny and finely shagreened; body brownish black to black. ***Minor workers*.** Whole head with sparse foveolae, frons and malar area with few additional, indistinct, longitudinal rugae, genae and centre of frons with sculpture reduced to absent; promesonotum low, slightly convex, with relatively steep posterior declivity; mesosoma foveolate, reduced on dorsal surface of pronotum, with katepisternum, anepisternum, promesonotal declivity, and lateral surfaces of propodeum smooth; propodeal spines very short, triangular; body dark brown.

###### Description.

**Major workers.** Measurements (*N* = 1): HL: 1.38; HW: 1.33; SL: 0.58; EL: 0.15; WL: 0.9; PSL: 0.2; MTL: 0.56; PNW: 0.63; PTW: 0.17; PPW: 0.61; CI: 96.3; SI: 43.8; PSLI: 14.1; PPI: 27.9; PNI: 47.1; MTI: 42.2. ***Head*.** In full-face view square, anterior and posterior of eyes relatively straight (Fig. 68B). In lateral view sub-rectangular; ventral and dorsal faces relatively flat; dorsal face indistinctly depressed posteriorly, forming indistinct transverse depression between frons and occipital lobes; inner hypostomal teeth visible. Sides of the head with dense, short, erect pilosity; whole head with dense, short, suberect to erect pilosity. Antennal scrobes indistinct and not delimited by carinulae; scrobe surface shiny, with dense to sparse, fine, longitudinal to irregular rugae, interspaces smooth to rugulae. Occipital lobes with thick, sparse, irregular rugae, interspaces smooth; frons with thick, sparse to moderately sparse, longitudinal rugae, interspaces smooth; malar area with sparse to moderately dense longitudinal rugae, interspaces smooth to finely rugulose; genae with fine, thin to indistinct rugulae. Clypeus shiny and smooth, with thin, longitudinal rugulae on the lateral sides; median notch present, wide and shallow; median longitudinal carina absent; lateral longitudinal carinae absent. Scape, when laid back, slightly surpassing the mid-length of head; pilosity decumbent to erect (Fig. 68B, D). Inner hypostomal teeth distinct, big, thick, triangular, with rounded apex; outer hypostomal teeth slightly but distinctly smaller and thin, with rounded tips, and wide base (Fig. 84T). ***Mesosoma*.** In lateral view, promesonotum short, angular, and relatively low, posterior mesonotum slightly steep, with indistinct tubercle-like projections; promesonotal groove absent; metanotal groove absent or indistinct; propodeal spines moderately long, with base slightly wider than top, apex rounded; humeral area with small and flat tubercles (Fig. 68D). Surface shiny, with fine and sparse rugoreticulation, pronotum with sculpture reduced, sometimes with smooth notches on lateral sides and centre of its dorsal surface; katepisternum and lateral sides of propodeum at least partially smooth. Pilosity moderately dense, long, and erect (Fig. 68D, F). ***Petiole*.** Shiny, with dense and fine rugulae; peduncle short, with small, rounded, horizontal lobes on its basal part; node moderately high and narrow, with convex apex, in rear view node slightly dorsoventrally concave; pilosity moderately and erect, long to moderately long (Fig. 68D, F). ***Postpetiole*.** Shiny, with fine and dense rugulae; short and rounded; in dorsal view sides with relatively long, acute, narrow and triangular projections; pilosity long, moderately dense, and erect (Fig. 68D, F). ***Petiole*.** First gastral tergite shiny and finely shagreened; pilosity moderately dense, moderately long, and erect (Fig. 68D, F). ***Colour*.** Unicolourous, brownish black to black; legs yellow to brown (Fig. 68D, F).

**Minor workers.** Measurements (*N* = 1): HL: 0.53; HW: 0.47; SL: 0.52; EL: 0.1; WL: 0.59; PSL: 0.1; MTL: 0.4; PNW: 0.32; PTW: 0.07; PPW: 0.11; CI: 88.4; SI: 111.4; PSLI: 18.0; PPI: 64.3; PNI: 68.9; MTI: 84.1. ***Head*.** Occipital margin straight or indistinctly concave; occipital carina indistinct, weakly developed (Fig. 68A). Pilosity moderately dense, long, erect. Whole head with sparse foveolae, frons and malar area with few additional, indistinct, longitudinal rugae, genae and centre of frons with sculpture reduced to absent. Clypeus shiny, rugoreticulate; median longitudinal carina absent; two lateral longitudinal carinae absent. Scape, when laid back, surpassing the posterior head margin by one-fifth of its length; pilosity erect (Fig. 68A, C). ***Mesosoma*.** In lateral view, promesonotum low, slightly convex, with relatively steep posterior declivity; promesonotal groove absent; metanotal groove indistinct; propodeal spines very short, triangular, apex acute (Fig. 68C). Sculpture foveolate, reduced on dorsal surface of pronotum, with katepisternum, anepisternum, promesonotal declivity, and lateral surfaces of propodeum smooth. Pilosity sparse, long, and erect (Fig. 68C, E). ***Petiole*.** Peduncle short and thin with ventral face slightly convex; node low, globular, and small; with few long, erect setae (Fig. 68C, E). ***Postpetiole*.** Moderately long, low, and convex; with few short, erect setae (Fig. 68C, E). ***Petiole*.** With moderately sparse, erect pilosity (Fig. 68C, E). ***Colour*.** Unicolourous, dark brown (Fig. 68C, E).

###### Etymology.

Latin for smooth, in reference to reduced sculpture of minor workers.

###### Biology.

The species was collected at 450 m in elevation, in rainforest. Nest was located in rotten log.

###### Comments.

This species is most similar to *P. petax* and *P. brevipilosa* sp. nov. ***Major workers*.***Pheidole glabra* sp. nov. can be distinguished from *P. petax* by presence of erect pilosity on sides of head and lateral sides of head, and square head shape in full-face view; from *P. brevipilosa* sp. nov. by absence of foveolate sculpture on head, well-developed outer hypostomal teeth, pronotum with reduced sculpture and presence of smooth notches on its lateral and dorsal surfaces, and propodeum with presence of smooth notches on lateral sides**. *Minor workers*.***Pheidole glabra* sp. nov. can be distinguished from *P. petax* by dark brown body colouration, presence of additional indistinct, longitudinal rugae on frons and malar area, clypeus never foveolate, and reduced sculpture on frons, dorsal surface of pronotum, promesonotal declivity, and lateral surfaces of propodeum; from *P. brevipilosa* sp. nov. by denser and longer pilosity on head, and frons, dorsal surface of pronotum, promesonotal declivity, and lateral surfaces of propodeum with sculpture reduced or absent.

#### Revision of the *Pheidole mavesatra* complex

**Diagnosis. *Major workers*.** Head in full-face view rectangular, anterior of eyes relatively straight, posterior of eyes slightly convex; in lateral view sub-rectangular; ventral and dorsal faces finely convex or relatively flat; scrobe surface shiny with sparse, thin, longitudinal rugae; occipital lobes with interspaces foveolae; frons with interspaces superficially foveolate; genae shiny, with distinct and thin rugulae; promesonotal groove absent; metanotal groove absent or indistinct; propodeal spines moderately long, triangular, with very wide base; mesosoma foveolate with additional thick, sparse, and irregular rugae on promesonotum and propodeum, and katepisternum smooth or mesosoma with thin, moderately dense to dense rugoreticulation with foveolate interspaces; gaster finely shagreened; body brown to brownish black. ***Minor workers*.** Head foveolate and genae with reduced sculpture; scape, when laid back, surpassing the posterior head margin by one-fifth of its length; promesonotum low, long, relatively flat or slightly convex, with steep posterior declivity; promesonotal groove absent; metanotal groove indistinct; propodeal spines moderately long to long, triangular or narrow; mesosoma foveolate; katepisternum at least partly, and sometimes mesosoma, smooth.

**Comments.** Major workers of this complex can be distinguished based on a combination of the following characters: head in full-face view rectangular and in lateral view sub-rectangular; foveolate antennal scrobes with additional thin, longitudinal rugae; occipital lobes with thick and irregular rugae and foveolate interspaces, foveolae fading posteriorly; moderately long and triangular propodeal spines; finely shagreened gaster and brown to brownish black body. Minor workers can be separated based on: foveolate head and mesosoma with reduced sculpture on genae and katepisternum; low and long promesonotum, with steep posterior declivity, and yellow body.

##### Pheidole mavesatra sp. nov.

Taxon classificationAnimaliaHymenopteraFormicidae

http://zoobank.org/EFA92733-E4F6-4351-9E36-91E266A59FD7

**Figure 69.**
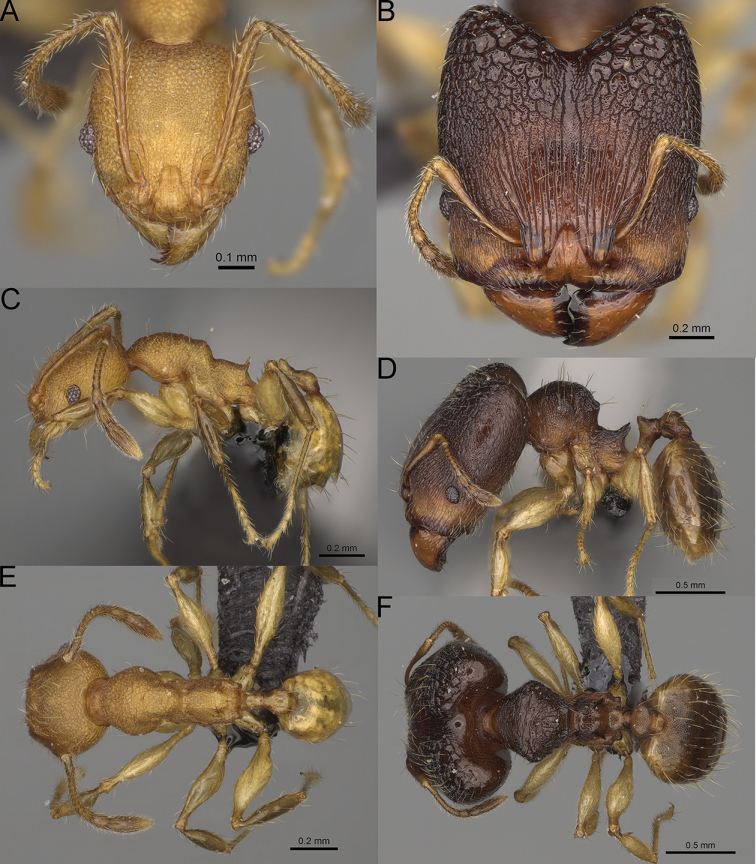
*Pheidole mavesatra* sp. nov., full-face view (**A**), profile (**C**), and dorsal view (**E**) of paratype minor worker (CASENT0923177) and full-face view (**B**), profile (**D**), and dorsal view (**F**) of holotype major worker (CASENT0134807).

Figs 69A–F , 85J , 87N

###### Type material.

***Holotype*.** Madagascar. •1 major worker; Toamasina; Tampolo, 39.4 km SSE Maroantsetra; -15.70978, 49.96965; alt. 218 m; 30 Aug 2007; B.L. Fisher et al. leg.; BLF17995, CASENT0134807 (CASC). ***Paratype*.** Madagascar. •1w.; same data as for holotype; CASENT0923177 (CASC).

###### Diagnosis.

***Major workers*.** Head in full-face view rectangular, anterior of eyes relatively straight, posterior of eyes slightly convex; sides of the head with sparse, relatively long, suberect pilosity; scrobe surface shiny, foveolate with sparse, thin, longitudinal rugae; closely spaced, low, triangular, with rounded apex directed inward; outer hypostomal teeth thinner and higher than outer hypostomal teeth, dentate, and with relatively narrow base; gaster finely shagreened; body brown to dark brown. ***Minor workers*.** Head foveolate, genae with reduced sculpture; promesonotum low, long, relatively flat, with steep posterior declivity; mesosoma foveolate; katepisternum and sometimes mesosoma smooth; propodeal spines moderately long, triangular, narrow; body yellow.

###### Description.

**Major workers.** Measurements (*N* = 1): HL: 1.3; HW: 1.2; SL: 0.53; EL: 0.14; WL: 0.92; PSL: 0.21; MTL: 0.52; PNW: 0.62; PTW: 0.15; PPW: 0.47; CI: 92.6; SI: 43.8; PSLI: 15.8; PPI: 32.5; PNI: 51.5; MTI: 43.1. ***Head*.** In full-face view rectangular, anterior of eyes relatively straight, posterior of eyes slightly convex (Fig. 69B). In lateral view sub-rectangular; ventral and dorsal faces finely convex; dorsal face finely depressed posteriorly, forming shallow transverse depression between frons and occipital lobes; inner hypostomal teeth visible. Sides of the head with sparse, relatively long, suberect pilosity; whole head with dense, moderately short, suberect to erect pilosity. Antennal scrobes indistinct and not delimited by carinulae; scrobe surface shiny, foveolate with sparse, thin, longitudinal rugae. Occipital lobes shiny, with indistinct foveolae and distinct, sparse, thick, irregular rugae, foveolae fading posteriorly; frons with dense, thick, and longitudinal rugae, interspaces superficially foveolate; genae shiny, with distinct and thin rugulae; malar area with thin, dense, longitudinal rugae. Centre of clypeus smooth and shiny, lateral sides with longitudinal rugae; median notch present, moderately wide, and shallow; median longitudinal carina indistinct; lateral longitudinal carinae present. Scape, when laid back, reaching the midlength of head; pilosity suberect to erect (Fig. 69B, D). Inner hypostomal teeth distinct, closely spaced, low, triangular, with rounded apex directed inward; outer hypostomal teeth thinner and higher than outer hypostomal teeth, dentate, and with relatively narrow base (Fig. 85J). ***Mesosoma*.** In lateral view, promesonotum relatively low and angular, posterior mesonotum steep, with indistinct tubercle-like projections; promesonotal groove absent; metanotal groove absent; propodeal spines moderately long, triangular, with rounded apex and very wide base; humeral area laterally well produced (Fig. 69D). Surface shiny and foveolate, foveolae fading on the dorsal surface of promesonotum; promesonotum and lateral surfaces of propodeum with additional thick, sparse, and irregular rugae; katepisternum smooth. Pilosity sparse, long and erect (Fig. 69D, F). ***Petiole*.** Shiny and with fine foveolae; peduncle moderately long, with indistinct horizontal lobes on its basal part; node relatively high, triangular, with rounded apex, in rear view node slightly convex; pilosity long and erect (Fig. 69D, F). ***Postpetiole*.** Shiny, finely shagreened; in dorsal view sides with moderately long, acute, and horn-like projections; pilosity long and erect (Fig. 69D, F). ***Petiole*.** Shiny and finely shagreened; pilosity moderately dense, long, and erect (Fig. 69D, F). ***Colour*.** Unicolourous, brown to dark brown; lower part of frons and malar area brighter that the rest of head (Fig. 69D, F).

**Minor workers.** Measurements (*N* = 1): HL: 0.51; HW: 0.48; SL: 0.51; EL: 0.11; WL: 0.6; PSL: 0.09; MTL: 0.37; PNW: 0.31; PTW: 0.05; PPW: 0.11; CI: 93.9; SI: 105.6; PSLI: 18.1; PPI: 46.5; PNI: 65.1; MTI: 78.0. ***Head*.** Occipital margin straight or indistinctly concave; occipital carina absent (Fig. 69A). Pilosity moderately sparse, moderately long, suberect to erect. Head foveolate; genae with reduced sculpture. Clypeus with fine and sometimes reduced foveolae; median longitudinal carina absent; two lateral longitudinal carinae absent. Scape, when laid back, surpassing the posterior head margin by one-fifth of its length; pilosity erect (Fig. 69A, C). ***Mesosoma*.** In lateral view, promesonotum low, long, relatively flat, with steep posterior declivity; promesonotal groove absent; metanotal groove indistinct; propodeal spines moderately long, triangular, narrow, and with acute apex (Fig. 69C). Sculpture foveolate; katepisternum and sometimes mesosoma smooth. Pilosity moderately sparse, long, and erect (Fig. 69C, E). ***Petiole*.** Peduncle short and thin with ventral face slightly convex; with few short, erect setae (Fig. 69C, E). ***Postpetiole*.** Moderately short, low and convex; with few short, erect setae (Fig. 69C, E). ***Petiole*.** With sparse, erect pilosity (Fig. 69C, E). ***Colour*.** Unicolourous, yellow (Fig. 69C, E).

###### Etymology.

Malagasy for heavy, in reference to massive head of major workers.

###### Biology.

The species was collected at 218 m in elevation, in disturbed rainforest. Nests were located under moss.

###### Comments.

*Pheidole mavesatra* sp. nov. is most similar to *P. goavana* sp. nov. ***Major workers*.***Pheidole mavesatra* sp. nov. differs from *P. goavana* sp. nov. in brown to dark brown body colouration, smooth katepisternum, and promesonotum and lateral surfaces of propodeum with additional thick, sparse, and irregular rugae. ***Minor workers*.***Pheidole mavesatra* sp. nov. differs from *P. goavana* sp. nov. in promesonotum low, long, relatively flat, and narrow propodeal spines.

##### Pheidole goavana sp. nov.

Taxon classificationAnimaliaHymenopteraFormicidae

http://zoobank.org/06E9F5A4-1CAD-4BC1-AA57-D41A7DF7204F

**Figure 70.**
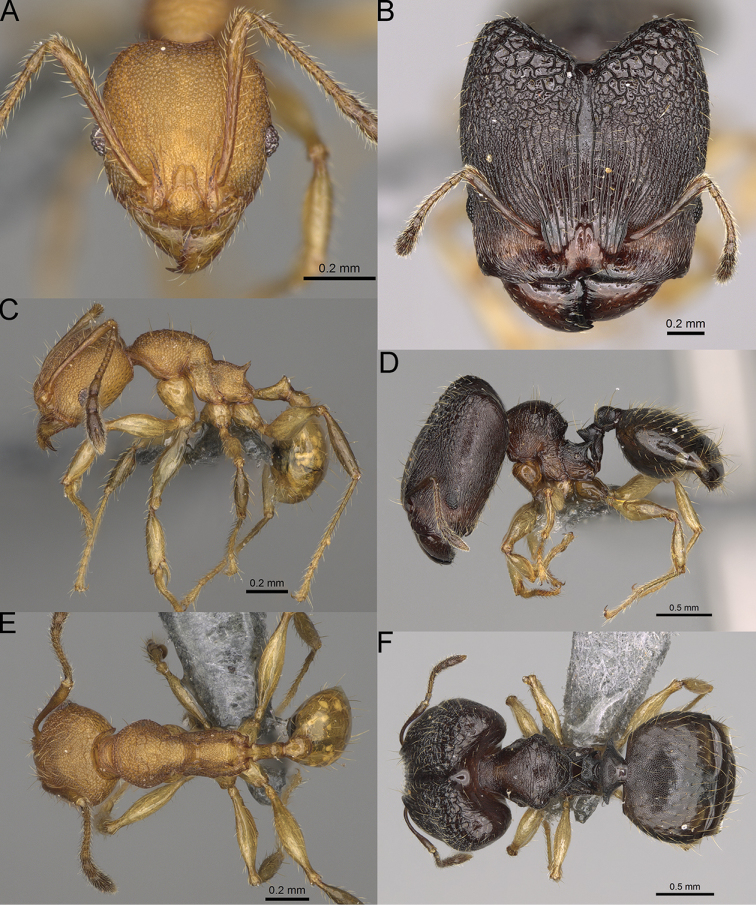
*Pheidole goavana* sp. nov., full-face view (**A**), profile (**C**), and dorsal view (**E**) of paratype minor worker (CASENT0923176) and full-face view (**B**), profile (**D**), and dorsal view (**F**) of holotype major worker (CASENT0374538).

Figs 70A–F , 84U , 86U

###### Type material.

***Holotype*.** Madagascar. •1 major worker; Antsiranana; Masoala National Park; -15.32331, 50.30751; alt. 60 m; 10 Mar 2014; B.L. Fisher et al. leg.; BLF33068, CASENT0374538 (CASC). ***Paratype*.** Madagascar. •1w.; same data as for holotype; CASENT0923176 (CASC).

###### Other material.

Madagascar. – ***Antsiranana***: •1s.; Forêt Ambanitaza, 26.1 km 347° Antalaha; -14.67933, 50.18367; alt. 240 m; 26 Nov 2004; B.L. Fisher et al. leg.; CASENT0054891 (CASC). •1w., 1s.; Masoala National Park; -15.3014, 50.22776; alt. 280 m; 7 Mar 2014; B.L. Fisher et al. leg.; CASENT0353609 (CASC). •2w., 1s., 1q.; Masoala National Park; -15.32331, 50.30751; alt. 60 m; 10 Mar 2014; B.L. Fisher et al. leg.; CASENT0374529, CASENT0374530 (CASC). •1w., 1s.; Parc National Montagne d’Ambre, Mahasarika; -12.53176, 49.17662; alt. 1135 m; 17 Nov 2007; B.L. Fisher et al. leg.; CASENT0134369 (CASC).

###### Diagnosis.

***Major workers*.** Head in full-face view rectangular, anterior of eyes relatively straight, posterior of eyes slightly convex; sides of the head with sparse, relatively long, suberect pilosity; scrobe surface shiny, foveolate with moderately dense, thin, longitudinal rugae; inner hypostomal teeth distinct, closely spaced, moderately high, triangular, with rounded apex; outer hypostomal teeth thinner and approximately as high as outer hypostomal teeth, dentate, and with relatively narrow base; gaster finely shagreened; body brownish black. ***Minor workers*.** Head foveolate, genae with reduced sculpture; promesonotum low, slightly convex, with steep posterior declivity; mesosoma foveolate; lower half of katepisternum smooth; propodeal spines moderately long, triangular, narrow; body yellow.

###### Description.

**Major workers.** Measurements (*N* = 4): HL: 1.31–1.49 (1.44); HW: 1.17–1.37 (1.32); SL: 0.54–0.6 (0.57); EL: 0.14–0.17 (0.15); WL: 0.9–0.99 (0.96); PSL: 0.19–0.24 (0.22); MTL: 0.53–0.57 (0.55); PNW: 0.62–0.69 (0.67); PTW: 0.17–0.2 (0.19); PPW: 0.54–0.61 (0.57); CI: 89.1–92.3 (91.4); SI: 42.1–46.0 (43.7); PSLI: 14.4–16.2 (15.3); PPI: 31.5–33.2 (32.3); PNI: 50.2–52.9 (51.0); MTI: 40.5–44.9 (42.1). ***Head*.** In full-face view rectangular, anterior of eyes relatively straight, posterior of eyes slightly convex (Fig. 70B). In lateral view sub-rectangular; ventral and dorsal faces relatively flat; dorsal face finely depressed posteriorly, forming indistinct transverse depression between frons and occipital lobes; inner hypostomal teeth visible. Sides of the head with sparse, relatively long, suberect pilosity; whole head with dense, short, suberect to erect pilosity. Antennal scrobes indistinct and not delimited by carinulae; scrobe surface shiny, foveolate with moderately dense, thin, longitudinal rugae. Occipital lobes shiny, with indistinct foveolae and distinct, sparse, thick, irregular rugae, foveolae fading posteriorly; frons with dense, thick, and longitudinal rugae, interspaces superficially foveolate; genae shiny, with dense and thin rugulae; malar area with thin, dense longitudinal rugae. Centre of clypeus smooth and shiny, lateral sides with longitudinal rugae; median notch present, moderately wide and shallow; median longitudinal carina present; lateral longitudinal carinae present. Scape, when laid back, reaching midlength of head; pilosity suberect (Fig. 70B, D). Inner hypostomal teeth distinct, closely spaced, moderately high, triangular, with rounded apex; outer hypostomal teeth thinner and approximately as high as outer hypostomal teeth, dentate, and with relatively narrow base (Fig. 84U). ***Mesosoma*.** In lateral view, promesonotum relatively low and angular, posterior mesonotum steep, with distinct tubercle-like projections; promesonotal groove absent; metanotal groove indistinct; propodeal spines moderately long, triangular, with rounded apex and wide base; humeral area laterally well produced (Fig. 70D). Surface shiny, with thin, moderately dense to dense rugoreticulation, interspaces finely foveolate. Pilosity moderately sparse, long, and erect (Fig. 70D, F). ***Petiole*.** Shiny and with fine foveolae; peduncle short, with distinct horizontal lobes on its basal part; node relatively high, triangular, with rounded apex, in rear view node dorsoventrally convex; pilosity moderately sparse and erect (Fig. 70D, F). ***Postpetiole*.** Shiny, finely shagreened; in dorsal view sides with long, very wide, acute, and triangular projections; pilosity moderately long and erect (Fig. 70D, F). ***Petiole*.** Shiny and finely shagreened; pilosity moderately dense, short, and erect (Fig. 70D, F). ***Colour*.** Unicolourous, brownish black; lower part of frons and malar area brighter that the rest of head (Fig. 70D, F).

**Minor workers.** Measurements (*N* = 4): HL: 0.48–0.55 (0.51); HW: 0.44–0.49 (0.47); SL: 0.46–0.5 (0.47); EL: 0.1–0.11 (0.1); WL: 0.6–0.63 (0.61); PSL: 0.1–0.12 (0.11); MTL: 0.36–0.38 (0.37); PNW: 0.31–0.34 (0.32); PTW: 0.06–0.07 (0.065); PPW: 0.12–0.14 (0.12); CI: 89.8–95.2 (92.3); SI: 98.3–103.9 (101.0); PSLI: 20.3–22.7 (21.3); PPI: 49.6–55.2 (53.1); PNI: 67.2–70.2 (68.7); MTI: 76.0–81.6 (78.8). ***Head*.** Occipital margin straight or indistinctly concave; occipital carina absent (Fig. 70A). Pilosity moderately sparse, moderately long, suberect to erect. Head foveolate; genae with reduced sculpture. Clypeus with fine and sometimes reduced foveolae; median longitudinal carina absent; two lateral longitudinal carinae absent. Scape, when laid back, surpassing the posterior head margin by one-fifth of its length; pilosity erect (Fig. 70A, C). ***Mesosoma*.** In lateral view, promesonotum low, long, slightly convex, with steep posterior declivity; promesonotal groove absent; metanotal groove indistinct; propodeal spines moderately long, triangular, narrow, and with acute apex (Fig. 70C). Sculpture foveolate; lower half of katepisternum smooth. Pilosity moderately sparse, long, and erect (Fig. 70C, E). ***Petiole*.** Peduncle short and thin with ventral face slightly convex; with few short, erect setae (Fig. 70C, E). ***Postpetiole*.** Short, low, and convex; with few short, erect setae (Fig. 70C, E). ***Petiole*.** With moderately sparse, erect pilosity (Fig. 70C, E). ***Colour*.** Unicolourous, yellow (Fig. 70C, E).

###### Etymology.

Malagasy for massive, in reference to massive head of major workers.

###### Biology.

The species was collected between 60–1135 m in elevation, in rainforest, and in montane rainforest. Nests were located in rotten logs.

###### Comments.

*Pheidole goavana* sp. nov. is most similar to *P. mavesatra* sp. nov. ***Major workers*.***Pheidole mavesatra* sp. nov. differs from *P. goavana* sp. nov. in brownish black body colouration, katepisternum never smooth, and promesonotum and lateral surfaces of propodeum with additional thin, moderately dense to dense rugoreticulation. ***Minor workers*.***Pheidole mavesatra* sp. nov. differs from *P. goavana* sp. nov. in promesonotum low, long, slightly convex, and triangular propodeal spines.

#### Revision of the *Pheidole ankerana* complex

**Diagnosis. *Major workers*.** Head in full-face view rectangular, in lateral view sub-rectangular, ventral and dorsal faces finely convex; sides of the head with moderately dense to dense, long, suberect to erect pilosity; scrobe surface shiny, with dense, thick, and irregular rugoreticulate or foveolate with thick, longitudinal rugae; occipital lobes and frons with interspaces smooth or finely rugulose; inner and outer teeth closely spaced and connected by concavity; promesonotal and metanotal grooves absent; propodeal spines moderately long, triangular; mesosoma with fine to thin, dense rugoreticulation and sometimes additional foveolae, sculpture weakening on dorsum, katepisternum smooth; gaster smooth; body yellowish to reddish brown. ***Minor worker*.** Whole head foveolate, sometimes with additional thin, sparse rugae; scape, when laid back, surpassing the posterior head margin by one to two-fifths of its length; promesonotum low, short, slightly convex; propodeal spines minute or relatively long, triangular; mesosoma foveolate, sometimes with additional thin, sparse rugae, katepisternum smooth.

**Comments.** Major workers of this complex can be distinguished based on a combination of the following characters: head in full-face view rectangular, in lateral view sub-rectangular; occipital lobes with thick, sparse, irregular rugae with at least partially smooth interspaces; inner and outer teeth closely spaced and connected by concavity; moderately long propodeal spines; sculpture of mesosoma weakening on dorsum; smooth gaster and yellowish to reddish brown body colouration. Minor workers have foveolate head and mesosoma, sometimes with additional thin rugae, smooth katepisternum, and yellow body.

##### Pheidole ankerana sp. nov.

Taxon classificationAnimaliaHymenopteraFormicidae

http://zoobank.org/5845FFBC-72D4-43A6-AF1D-11C57445217F

**Figure 71.**
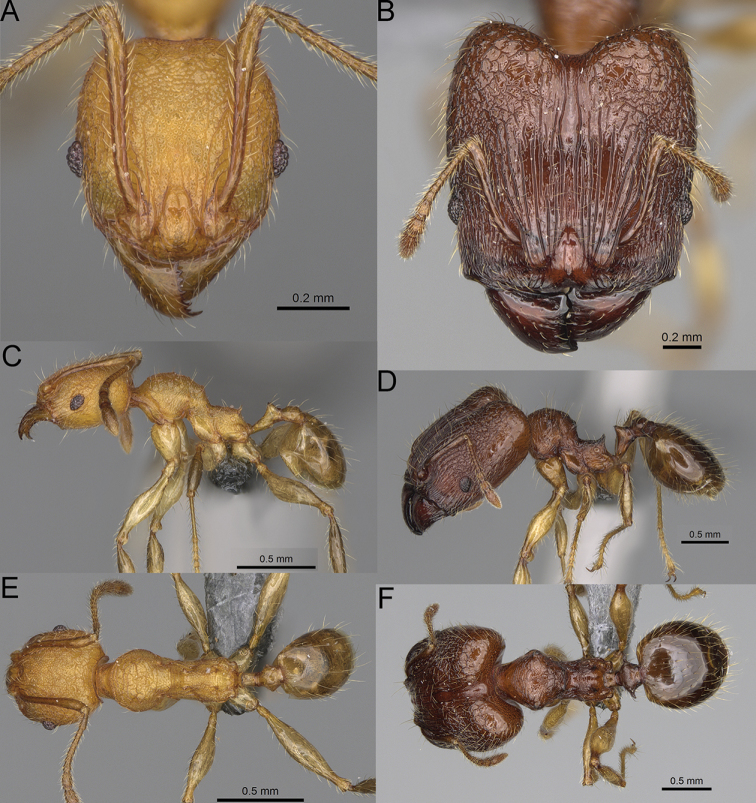
*Pheidole ankerana* sp. nov., full-face view (**A**), profile (**C**), and dorsal view (**E**) of paratype minor worker (CASENT0274003) and full-face view (**B**), profile (**D**), and dorsal view (**F**) of holotype major worker (CASENT0923171).

Figs 71A–F , 84D , 86D

###### Type material.

***Holotype*.** Madagascar. •1 major worker; Toamasina; Ankerana; -18.4104, 48.8189; alt. 855 m; 27 Jan 2012; B.L. Fisher et al. leg.; BLF28148, CASENT0923171 (CASC). ***Paratypes*.** Madagascar. •2w., 1q.; same data as for holotype; CASENT0274002, CASENT0274003, CASENT0872185 (CASC).

###### Other material.

Madagascar. – ***Toamasina***: •3w.; Ankerana; -18.4061, 48.82029; alt. 725 m; 20 Jan 2012; B.L. Fisher et al. leg.; CASENT0274747, CASENT0274762 (CASC). •3w., 1m.; Ankerana; -18.40829, 48.82107; alt. 750 m; 21 Jan 2012; B.L. Fisher et al. leg.; CASENT0275260, CASENT0275261 (CASC). •2w.; Ankerana; -18.4104, 48.8189; alt. 855 m; 22 Jan 2012; B.L. Fisher et al. leg.; CASENT0274408, CASENT0275713 (CASC). •2w.; Ankerana; -18.40062, 48.81311; alt. 865 m; 17 Jan 2012; B.L. Fisher et al. leg.; CASENT0274909 (CASC). •1w., 1s.; Parc National de Zahamena, Besaky River; -17.75244, 48.85321; alt. 760 m; 22 Feb 2009; B.L. Fisher et al. leg.; CASENT0149769 (CASC).

###### Diagnosis.

***Major workers*.** Head in full-face view rectangular, with lateral sides relatively straight, only posteriormost part slightly convex; sides of the head with dense, long, suberect pilosity; occipital lobes and genae shiny, with thick, sparse, irregular rugae; frons with sparse, thick, and longitudinal rugae, interspaces smooth; inner hypostomal teeth distinct, small, and low, closely spaced, lobe-like, with rounded apex and wide base; outer hypostomal teeth bigger and wider than inner hypostomal teeth, triangular; inner and outer teeth closely spaced and connected by concavity; propodeal spines moderately long, triangular, with acute apex; gaster smooth; body reddish brown. ***Minor workers*.** Head foveolate with additional thin, sparse rugae on the whole surface; promesonotum low, short, slightly convex, with relatively steep posterior declivity; propodeal spines minute, triangular; mesosoma foveolate with additional thin, sparse rugae, katepisternum smooth.

###### Description.

**Major workers.** Measurements (*N* = 5): HL: 1.42–1.52 (1.48); HW: 1.34–1.43 (1.38); SL: 0.61–0.65 (0.63); EL: 0.16–0.2 (0.18); WL: 1.07–1.16 (1.12); PSL: 0.19–0.21 (0.2); MTL: 0.65–0.68 (0.66); PNW: 0.62–0.67 (0.64); PTW: 0.15–0.17 (0.16); PPW: 0.47–0.51 (0.49); CI: 92.1–94.1 (93.3); SI: 44.7–46.2 (45.5); PSLI: 12.1–14.6 (13.4); PPI: 28.9–32.7 (31.6); PNI: 45.7–47.5 (46.6); MTI: 47.4–48.8 (48.3). ***Head*.** In full-face view rectangular, with lateral sides relatively straight, only posteriormost part slightly convex (Fig. 71B). In lateral view sub-rectangular; ventral and dorsal faces finely convex; dorsal face finely depressed posteriorly, forming shallow transverse depression between frons and occipital lobes; inner hypostomal teeth invisible. Sides of the head with dense, long, suberect pilosity; whole head with dense, moderately long, suberect to erect pilosity. Antennal scrobes indistinct and not delimited by carinulae; scrobe surface shiny, with dense, thick, and irregular rugoreticulation. Occipital lobes and genae shiny, with thick, sparse, irregular rugae; frons with sparse, thick, and longitudinal rugae, interspaces smooth; malar area with dense, thick, and longitudinal rugae, interspaces smooth to finely rugulose. Centre of clypeus smooth and shiny, lateral sides with longitudinal rugae; median notch present, narrow and shallow to moderate; median longitudinal carina present; lateral longitudinal carinae absent. Scape, when laid back, slightly exceeding the midlength of head; pilosity decumbent to erect (Fig. 71B, D). Inner hypostomal teeth distinct, small and low, closely spaced, lobe-like, with rounded apex and wide base; outer hypostomal teeth bigger and wider than inner hypostomal teeth, triangular; inner and outer teeth closely spaced and connected by concavity (Fig. 84D). ***Mesosoma*.** In lateral view, promesonotum short, angular, and low, posterior mesonotum relatively steep, with indistinct tubercle-like projection; promesonotal groove absent; metanotal groove absent; propodeal spines moderately long, triangular, with acute apex; humeral area laterally weakly produced (Fig. 71D). Surface shiny, with fine to thin, dense rugoreticulation, sculpture weakening on dorsum, katepisternum smooth. Pilosity moderately sparse, long, and erect (Fig. 71D, F). ***Petiole*.** Shiny; peduncle shagreened, short, without horizontal lobes on its basal part; node smooth, relatively high and thin, triangular, with rounded apex, in rear view node straight; pilosity moderately sparse and erect (Fig. 71D, F). ***Postpetiole*.** Finely shagreened, shiny; in dorsal view sides with moderately short, acute, and horn-like projections; pilosity long, moderately sparse and erect (Fig. 71D, F). ***Petiole*.** Smooth; pilosity moderately dense, long, and erect (Fig. 71D, F). ***Colour*.** Unicolourous, reddish brown; legs dark yellow (Fig. 71D, F).

**Minor workers.** Measurements (*N* = 5): HL: 0.61–0.69 (0.66); HW: 0.55–0.63 (0.59); SL: 0.58–0.64 (0.61); EL: 0.11–0.13 (0.12); WL: 0.75–0.85 (0.8); PSL: 0.09–0.11 (0.1); MTL: 0.48–0.55 (0.51); PNW: 0.37–0.43 (0.4); PTW: 0.06–0.08 (0.07); PPW: 0.13–0.2 (0.16); CI: 88.5–93.8 (90.3); SI: 99.2–105.1 (102.4); PSLI: 14.4–17.3 (15.5); PPI: 38.2–52.7 (45.5); PNI: 66.1–68.6 (67.6); MTI: 79.5–90.1 (86.9). ***Head*.** Occipital margin straight or indistinctly concave; occipital carina indistinct, weakly developed (Fig. 71A). Pilosity moderately dense, long, erect. Whole head foveolate with additional thin, sparse rugae on the whole surface. Clypeus shiny, with sparse, irregular rugae; median longitudinal carina absent; two lateral longitudinal carinae absent. Scape, when laid back, surpassing the posterior head margin by one-fifth of its length; pilosity erect (Fig. 71A, C). ***Mesosoma*.** In lateral view, promesonotum low, short, slightly convex, with relatively steep posterior declivity; promesonotal groove absent; metanotal groove absent; propodeal spines minute, triangular, apex acute (Fig. 71C). Sculpture foveolate with additional thin, sparse rugae on the whole surface, katepisternum smooth. Pilosity sparse, long, and erect (Fig. 71C, E). ***Petiole*.** Peduncle short and thin; node moderately high, triangular and small; with few long, erect setae (Fig. 71C, E). ***Postpetiole*.** Short, low, and slightly convex; with few short, erect setae (Fig. 71C, E). ***Petiole*.** With moderately sparse, erect pilosity (Fig. 71C, E). ***Colour*.** Unicolourous, yellow (Fig. 71C, E).

###### Etymology.

From the type locality.

###### Biology.

The species was collected between 725–865 m in elevation, in rainforest. Nests were located in rotten logs.

###### Comments.

***Major workers*.***Pheidole ankerana* sp. nov. is most similar to *P. vatovavensis*. It differs from *P. vatovavensis* in denser and longer pilosity on the sides of head, smooth sculpture of interspaces on frons, sculpture of scrobes never foveolate, lower promesonotum and lobe-like shape of inner hypostomal teeth. ***Minor workers*.***Pheidole ankerana* sp. nov. differs from *P. vatovavensis* sp. nov. by presence of additional thin, sparse rugae on the whole surface of head and mesosoma and minute size of propodeal spines.

##### Pheidole vatovavensis sp. nov.

Taxon classificationAnimaliaHymenopteraFormicidae

http://zoobank.org/93F87A9F-0EC1-4FF5-80BA-A0FFD0E38D60

**Figure 72.**
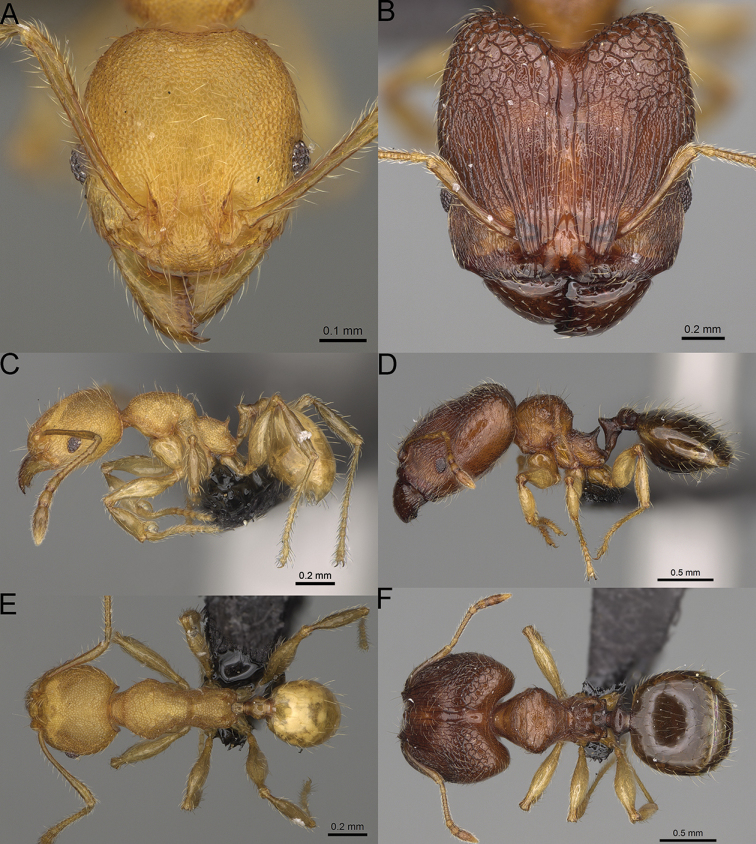
*Pheidole vatovavensis* sp. nov., full-face view (**A**), profile (**C**), and dorsal view (**E**) of paratype minor worker (CASENT0923179) and full-face view (**B**), profile (**D**), and dorsal view (**F**) of holotype major worker (CASENT0060282).

Figs 72A–F , 85Y , 88I

###### Type material.

***Holotype*.** Madagascar. •1 major worker; Fianarantsoa; 7.6 km 122° Kianjavato, Forêt Classée Vatovavy; -21.4, 47.94; alt. 175 m; 6 Jun 2005; B.L. Fisher et al. leg.; BLF12315, CASENT0060282 (CASC). ***Paratypes*.** Madagascar. •2w.; same data as for holotype; CASENT0060283, CASENT0923179 (CASC).

###### Other material.

Madagascar. – ***Fianarantsoa***: •7w., 10s., 1q.; 7.6 km 122° Kianjavato, Forêt Classée Vatovavy; -21.4, 47.94; alt. 175 m; 6 Jun 2005; B.L. Fisher et al. leg.; CASENT0060466, CASENT0060467CASENT0060470–CASENT0060472, CASENT0061080, CASENT0061081, CASENT0061146, CASENT0061179, CASENT0061183, CASENT0061257, CASENT0061259, CASENT0061351, CASENT0061352, CASENT0061435 (CASC).

###### Diagnosis.

***Major workers*.** Head in full-face view sub-rectangular, with lateral sides relatively straight, only posterior-most part slightly convex; sides of the head with moderately dense, long, erect pilosity; occipital lobes shiny, with thick, irregular rugae, interspaces with indistinct, irregular rugulae fading posteriorly; frons with moderately dense, thick, and longitudinal rugae, interspaces with dense but fine and irregular rugulae; inner hypostomal teeth distinct, small, and low, closely spaced, triangular, with rounded apex and wide base; outer hypostomal teeth bigger and wider than inner hypostomal teeth, triangular, with tops directed inward, triangular; inner and outer teeth closely spaced and connected by concavity; gaster smooth; body reddish brown. ***Minor workers*.** Head foveolate; genae with fading sculpture; promesonotum low, convex, moderately long, with posterior declivity smoothly declining towards propodeum; propodeal spines relatively long, triangular; mesosoma foveolate, katepisternum smooth.

###### Description.

**Major workers.** Measurements (*N* = 8): HL: 1.22–1.37 (1.3); HW: 1.19–1.31 (1.24); SL: 0.53–0.59 (0.56); EL: 0.13–0.16 (0.15); WL: 0.92–1.01 (0.97); PSL: 0.19–0.23 (0.21); MTL: 0.52–0.56 (0.54); PNW: 0.58–0.65 (0.61); PTW: 0.12–0.15 (0.13); PPW: 0.45–0.51 (0.47); CI: 94.2–99.4 (95.4); SI: 42.3–46.7 (44.9); PSLI: 14.7–16.8 (15.9); PPI: 26.1–33.3 (28.0); PNI: 46.6–51.7 (49.2); MTI: 41.4–45.2 (43.3). ***Head*.** In full-face view longer than wide, anterior of eyes straight, posterior of eyes convex (Fig. 72B). In lateral view sub-rectangular; ventral and dorsal faces finely convex; dorsal face finely depressed posteriorly, forming shallow transverse depression between frons and occipital lobes; inner hypostomal teeth invisible. Sides of the head with moderately dense, long, erect pilosity; whole head with dense, long, suberect to erect pilosity. Antennal scrobes weakly impressed and indistinctly delimited ventrally and posteriorly by carinulae; scrobe surface shiny, foveolate with thick, longitudinal, and long rugae. Occipital lobes shiny, with thick, irregular rugae, interspaces with indistinct, irregular rugulae fading posteriorly; frons with moderately dense, thick, and longitudinal rugae, interspaces with dense but fine and irregular rugulae; genae shiny, with dense and thin, irregular rugulae, central part with reduced sculpture; malar area with thin, longitudinal, dense rugoreticulation. Centre of clypeus shiny and smooth, lateral sides with longitudinal rugulae; median notch present, moderately wide and shallow; median longitudinal carina present; lateral longitudinal carinae present. Scape, when laid back, slightly surpassing the midlength of head; pilosity suberect (Fig. 72B, D). Inner hypostomal teeth distinct, small, and low, closely spaced, triangular, with rounded apex and wide base; outer hypostomal teeth bigger and wider than inner hypostomal teeth, triangular, with tops directed inward, triangular; inner and outer teeth closely spaced and connected by concavity (Fig. 85Y). ***Mesosoma*.** In lateral view, promesonotum relatively low and arched, posterior mesonotum steep, without tubercle-like projections; promesonotal groove absent; metanotal groove absent; propodeal spines moderately long, triangular, with rounded apex and wide base; humeral area laterally well produced (Fig. 72D). Surface shiny, with fine and sparse foveolae and additional rugoreticulation; dorsal surface of promesonotum with fading foveolae and a few thick, transverse rugae; katepisternum smooth. Pilosity moderately sparse, long, and erect (Fig. 72D, F). ***Petiole*.** Shiny and with fine foveolae; peduncle short, with indistinct horizontal lobes on its basal part; node relatively high, triangular, with rounded apex, in rear view node slightly convex; pilosity moderately sparse and erect (Fig. 72D, F). ***Postpetiole*.** Shiny, finely shagreened; in dorsal view sides with moderately long, triangular projections; pilosity long, moderately sparse, and erect (Fig. 72D, F). ***Petiole*.** Shiny and smooth; pilosity moderately sparse, long, and erect (Fig. 72D, F). ***Colour*.** Head reddish brown; mesosoma and legs yellowish brown; gaster brown (Fig. 72D, F).

**Minor workers.** Measurements (*N* = 2): HL: 0.56–0.52; HW: 0.48–0.47; SL: 0.5–0.48; EL: 0.1–0.1; WL: 0.64–0.6; PSL: 0.11–0.1; MTL: 0.38–0.37; PNW: 0.33–0.33; PTW: 0.06–0.06; PPW: 0.14–0.16; CI: 86.4–90.5; SI: 103.7–100.2; PSLI: 19.8–19.3; PPI: 42.2–47.2; PNI: 67.1–69.6; MTI: 77.7–77.2. ***Head*.** Occipital margin straight or indistinctly concave; occipital carina absent (Fig. 72A). Pilosity moderately sparse, long, suberect to erect. Head foveolate; genae with fading sculpture. Clypeus foveolate; median longitudinal carina absent; two lateral longitudinal carinae absent. Scape, when laid back, surpassing the posterior head margin by two-fifths of its length; pilosity suberect (Fig. 72A, C). ***Mesosoma*.** In lateral view, promesonotum low, convex, moderately long, with posterior declivity smoothly declining toward propodeum; promesonotal groove absent; metanotal groove distinct; propodeal spines relatively long, triangular, with acute apex (Fig. 72C). Sculpture foveolate; katepisternum smooth. Pilosity moderately sparse, short, and erect (Fig. 72C, E). ***Petiole*.** Peduncle moderately short and thin with ventral face slightly convex; with few long, erect setae (Fig. 72C, E). ***Postpetiole*.** Short, low, and convex; with few short, erect setae (Fig. 72C, E). ***Petiole*.** With sparse, erect pilosity (Fig. 72C, E). ***Colour*.** Yellow (Fig. 72C, E).

###### Etymology.

From the type locality.

###### Biology.

The species was collected at 175 m in elevation, in rainforest. Nests were located in rotten logs.

###### Comments.

*Pheidole vatovavensis* sp. nov. is most similar to *P. ankerana* sp. nov. ***Major workers*.***Pheidole vatovavensis* sp. nov. differs from *P. ankerana* sp. nov. in sparser and shorter pilosity on the sides of head, frons with interspaces never smooth, foveolate sculpture on scrobes, higher promesonotum, and triangular shape of inner hypostomal teeth. ***Minor workers*.***Pheidole vatovavensis* sp. nov. differs from *P. ankerana* sp. nov. in lack of additional thin, sparse rugae on the head and mesosoma and presence of relatively long propodeal spines.

#### Revision of the *Pheidole boribora* complex

**Diagnosis. *Major workers*.** Head in full-face view oval, in lateral view sub-rectangular, ventral and dorsal faces finely convex; sides of the head with sparse to moderately dense, short or long, erect pilosity; occipital lobes with interspaces smooth to finely foveolate; frons with interspaces smooth to finely foveolate; lateral sides of head with additional thin, sparse, longitudinal rugae or with thin, dense irregular rugoreticulation; promesonotal groove absent; metanotal groove absent or indistinct; propodeal spines small or moderately long, triangular; mesosoma with very sparse, transverse to irregular thin rugae, sometimes pronotum and propodeum with additional indistinct and sparse foveolae; gaster finely shagreened; body yellowish to reddish brown. ***Minor workers*.** Head foveolate; scape, when laid back, surpassing the posterior head margin by two-fifths of its length; promesonotum low, short, slightly convex; mesosoma foveolate, but mesonotum, anepisternum, katepisternum and lateral surfaces of propodeum smooth.

**Comments.** Major workers of this complex can be distinguished based on a combination of the following characters: head in full-face view oval, in lateral view sub-rectangular; occipital lobes with thick, sparse, irregular rugae, interspaces smooth to finely foveolate; mesosoma predominately with very sparse, transverse to irregular, thin rugae; finely shagreened gaster and yellowish to reddish brown body. Minor workers can be separated based on the following characters: foveolate head and mesosoma, but sculpture of mesosoma strongly reduced to absent on its lateral sides and yellow body.

Minor workers of *P. miramila* sp. nov. are unknown.

##### Pheidole boribora sp. nov.

Taxon classificationAnimaliaHymenopteraFormicidae

http://zoobank.org/EDD6BF4F-581F-4246-B931-502AB27EC1BB

**Figure 73.**
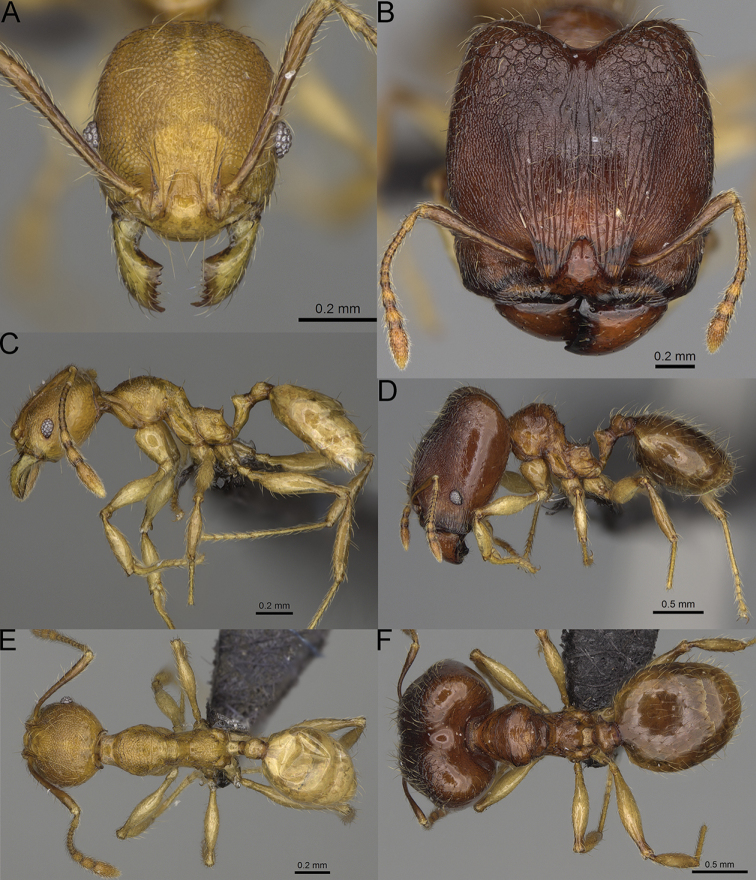
*Pheidole boribora* sp. nov., full-face view (**A**), profile (**C**), and dorsal view (**E**) of paratype minor worker (CASENT0456014) and full-face view (**B**), profile (**D**), and dorsal view (**F**) of holotype major worker (CASENT0456013).

Figs 73A–F , 84J , 86J

###### Type material.

***Holotype*.** Madagascar. •1 major worker; Toliara; Parc National d’Andohahela, Col du Sedro, 3.8 km 113°ESE Mahamavo, 37.6 km 341°NNW Tolagnaro; -24.76389, 46.75167; alt. 900 m; 21 Jan 2002; B.L. Fisher et al. leg.; BLF05130, CASENT0456013 (CASC). ***Paratypes*.** Madagascar. •4w.; same data as for holotype; CASENT0456014–CASENT0456016, CASENT0872187–CASENT0872190 (CASC).

###### Diagnosis.

***Major workers*.** Head in full-face view oval, relatively as long as wide, anterior and posterior of eyes moderately convex; sides of the head with moderately dense, long, erect pilosity; genae smooth and shiny; inner hypostomal teeth distinct, distinct, low, thick, and triangular, with rounded apex; outer hypostomal teeth approximately the same size as inner hypostomal teeth, thick, dentate, with rounded tips; propodeal spines small, triangular; first gastral tergite finely shagreened; body reddish brown. ***Minor workers*.** Head foveolate; promesonotum low, short, slightly convex, with relatively steep posterior declivity; propodeal spines small; mesosoma foveolate, foveolate, mesonotum, anepisternum, katepisternum and lateral surfaces of propodeum smooth; body yellow.

###### Description.

**Major workers.** Measurements (*N* = 1): HL: 1.5; HW: 1.4; SL: 0.66; EL: 0.17; WL: 1.07; PSL: 0.17; MTL: 0.66; PNW: 0.58; PTW: 0.19; PPW: 0.41; CI: 93.4; SI: 47.2; PSLI: 11.2; PPI: 45.5; PNI: 41.6; MTI: 46.9. ***Head*.** In full-face view oval, anterior and posterior of eyes moderately convex (Fig. 73B). In lateral view sub-rectangular; ventral and dorsal faces finely convex; dorsal face finely depressed posteriorly, forming shallow transverse depression between frons and occipital lobes; inner hypostomal teeth invisible. Sides of the head with moderately dense, long, erect pilosity; whole head with moderately dense, short, suberect to erect pilosity. Antennal scrobes indistinct and not delimited by carinulae. Occipital lobes shiny, with thick, sparse, irregular rugae, interspaces smooth to finely foveolate; frons with thick, moderately dense, longitudinal rugae, interspaces smooth to finely foveolate; lateral sides of head foveolate, with additional thin, sparse, longitudinal rugae; malar area with dense and thin longitudinal rugulae; genae smooth and shiny. Clypeus shiny and smooth, with thin, longitudinal rugulae on the lateral sides; median notch present, narrow, and moderately deep; median longitudinal carina absent; lateral longitudinal carinae absent. Scape, when laid back, reaching the midlength of head; pilosity decumbent to erect (Fig. 73B, D). Inner hypostomal teeth distinct, low, thick, and triangular, with rounded apex; outer hypostomal teeth approximately the same size as inner hypostomal teeth, thick, dentate, with rounded tips (Fig. 84J). ***Mesosoma*.** In lateral view, promesonotum short, angular, and relatively low, posterior mesonotum steep, with moderately large, tubercle-like projections; promesonotal groove absent; metanotal groove absent or indistinct; propodeal spines small, triangular, with base wide, apex rounded; humeral area with small and flat tubercles (Fig. 73D). Surface shiny, with very sparse, transverse to irregular thin rugae, pronotum and propodeum with additional indistinct and sparse foveolae. Pilosity moderately dense, long, and erect (Fig. 73D, F). ***Petiole*.** Shiny, finely shagreened to smooth; peduncle short, with small, rounded, horizontal lobes on its basal part; node moderately high and triangular, with convex apex, in rear view node relatively straight; pilosity moderately sparse and erect (Fig. 73D, F). ***Postpetiole*.** Shiny and finely shagreened; short and rounded; in dorsal view sides with short, acute, triangular projections; pilosity long, moderately dense, and erect (Fig. 73D, F). ***Petiole*.** First gastral tergite shiny and finely shagreened; pilosity dense, long and erect (Fig. 73D, F). ***Colour*.** Head reddish brown; mesosoma and gaster yellowish brown to brown; legs dark yellow (Fig. 73D, F).

**Minor workers.** Measurements (*N* = 3): HL: 0.55–0.58 (0.56); HW: 0.49–0.5 (0.49); SL: 0.57–0.58 (0.57); EL: 0.11–0.11 (0.11); WL: 0.68–0.76 (0.74); PSL: 0.07–0.1 (0.08); MTL: 0.42–0.46 (0.43); PNW: 0.31–0.32 (0.31); PTW: 0.06–0.08 (0.07); PPW: 0.12–0.14 (0.13); CI: 85.8–89.4 (87.5); SI: 116.2–116.8 (116.4); PSLI: 12.0–17.0 (14.6); PPI: 46.0–59.8 (53.3); PNI: 62.9–64.2 (63.5); MTI: 85.0–91.2 (88.0). ***Head*.** Occipital margin straight or indistinctly concave; occipital carina indistinct, weakly developed (Fig. 73A). Pilosity moderately dense, long, erect. Whole head foveolate. Clypeus with median longitudinal carina absent; two lateral longitudinal carinae absent. Scape, when laid back, surpassing the posterior head margin by two-fifths of its length; pilosity erect (Fig. 73A, C). ***Mesosoma*.** In lateral view, promesonotum low, short, slightly convex, with relatively steep posterior declivity; promesonotal groove absent; metanotal groove absent; propodeal spines small, triangular, apex acute (Fig. 73C). Sculpture foveolate; katepisternum, anepisternum and mesonotum smooth. Pilosity sparse, long, and erect (Fig. 73C, E). ***Petiole*.** Peduncle short and thin with ventral face slightly convex; node low, triangular, and small; with few moderately long, erect setae (Fig. 73C, E). ***Postpetiole*.** Short, low, and convex; with few moderately long, erect setae (Fig. 73C, E). ***Petiole*.** With moderately sparse, erect pilosity (Fig. 73C, E). ***Colour*.** Unicolourous, yellow (Fig. 73C, E).

###### Etymology.

Malagasy for oval, in reference to the head shape of major workers.

###### Biology.

The species was collected at 900 m in elevation, in montane rainforest. Nest was located in a rotten log.

###### Comments.

***Major workers*.***Pheidole boribora* sp. nov. is most similar to *P. miramila* sp. nov. and differs from it by presence of foveolae on head, dense and long pilosity of sides of head, dentate inner hypostomal teeth, and small propodeal spines. ***Minor workers*.***Pheidole boribora* sp. nov. is most similar to *P. petax* and differs from it by smooth mesonotum, anepisternum, and lateral surfaces of propodeum.

##### Pheidole miramila sp. nov.

Taxon classificationAnimaliaHymenopteraFormicidae

http://zoobank.org/14F03E8E-3A4A-44F1-A4A2-95C4E609DA58

**Figure 74.**
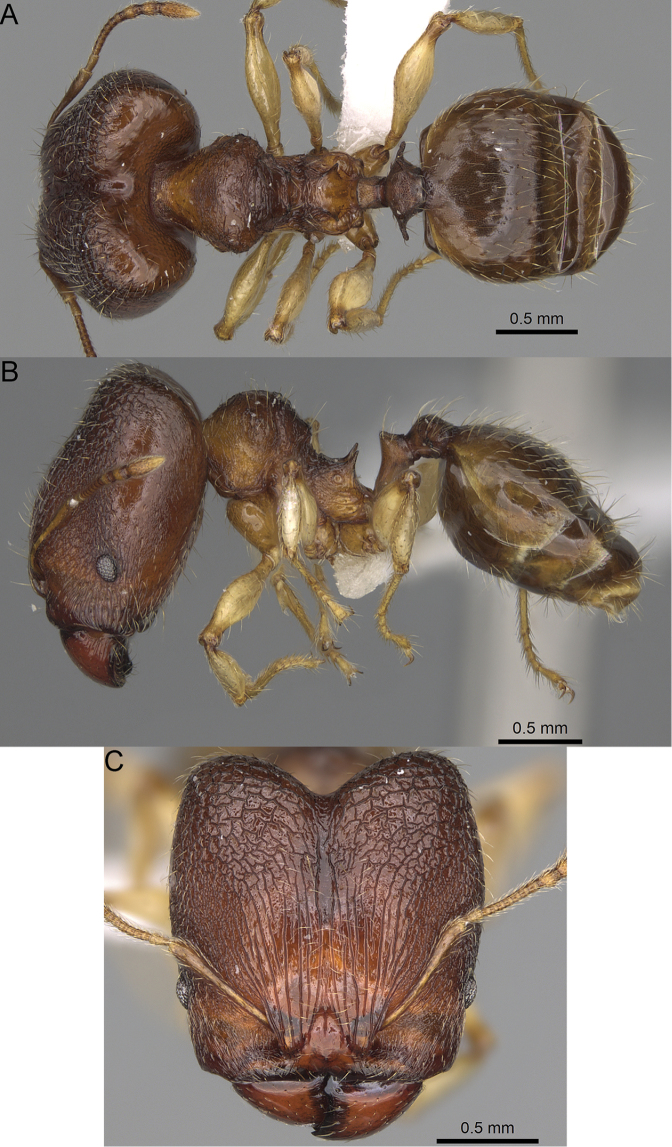
*Pheidole miramila* sp. nov., dorsal view (**A**), profile (**B**), and full-face view (**C**) of holotype major worker (CASENT0198567).

Figs 74A–C , 85K , 87O

###### Type material.

***Holotype*.** Madagascar. •1 major worker; Fianarantsoa; 45 km S. Ambalavao; -22.21667, 47.01667; alt. 785 m; 25 Sep 1993; B.L. Fisher leg.; BLF00696, CASENT0198567, top specimen (CASC). ***Paratype*.** Madagascar. •1s.; same data as for holotype; CASENT0872079 (CASC).

###### Diagnosis.

***Major workers*.** Head in full-face view oval, relatively as long as wide, anterior and posterior of eyes moderately convex; sides of the head with sparse, short, erect pilosity; genae smooth and shiny; inner hypostomal teeth distinct, low, thick, and triangular, with rounded apex; outer hypostomal teeth higher, thick, dentate, with rounded tips; propodeal spines moderately long, triangular; first gastral tergite finely shagreened; body yellowish brown to brown.

###### Description.

**Major workers.** Measurements (*N* = 2): HL: 1.63–1.7; HW: 1.57–1.59; SL: 0.68–0.67; EL: 0.19–0.16; WL: 1.12–1.15; PSL: 0.26–0.24; MTL: 0.65–0.69; PNW: 0.76–0.74; PTW: 0.18–0.2; PPW: 0.64–0.61; CI: 96.8–93.2; SI: 43.0–42.2; PSLI: 16.2–14.3; PPI: 27.9–33.0; PNI: 48.1–46.4; MTI: 41.5–43.7. ***Head*.** In full-face view oval, anterior and posterior of eyes moderately convex (Fig. 74C). In lateral view sub-rectangular; ventral and dorsal faces finely convex; dorsal face finely depressed posteriorly, forming shallow transverse depression between frons and occipital lobes; inner hypostomal teeth invisible. Sides of the head with sparse, short, erect pilosity; whole head with moderately dense, short, fine, suberect to erect pilosity. Antennal scrobes indistinct and not delimited by carinulae. Occipital lobes shiny, with thick, sparse, irregular rugae, interspaces smooth; frons with thick, moderately dense, longitudinal rugae, interspaces smooth; lateral sides of head with thin, dense, irregular rugoreticulation; malar area with dense and thin longitudinal rugulae; genae smooth and shiny. Clypeus shiny and smooth, with thin, longitudinal rugulae on the lateral sides; median notch present, narrow and moderately deep; median longitudinal carina absent; lateral longitudinal carinae absent. Scape, when laid back, reaching the midlength of head; pilosity decumbent to erect (Fig. 74B, C). Inner hypostomal teeth distinct, low, thick and triangular, with rounded apex; outer hypostomal teeth higher, thick, dentate, with rounded tips (Fig. 85K). ***Mesosoma*.** In lateral view, promesonotum short, angular, and relatively low, posterior mesonotum steep, with moderately large, tubercle-like projections; promesonotal groove absent; metanotal groove absent or indistinct; propodeal spines moderately long, triangular, with base wide, apex rounded; humeral area with small and flat tubercles (Fig. 74B). Surface shiny, with very sparse, transverse to irregular thin rugae. Pilosity moderately sparse, long, and erect (Fig. 74A, B). ***Petiole*.** Shiny, finely shagreened to smooth; peduncle short, with small, rounded horizontal lobes on its basal part; node moderately high and triangular, with convex apex, in rear view node relatively straight; pilosity moderately sparse and erect (Fig. 74A, B). ***Postpetiole*.** Shiny and finely shagreened; short and rounded; in dorsal view sides with relatively long, acute, triangular projections; pilosity long, moderately sparse and erect (Fig. 74A, B). ***Petiole*.** First gastral tergite shiny and finely shagreened; pilosity moderately sparse, long and erect (Fig. 74A, B). ***Colour*.** Unicolourous, yellowish brown to brown (Fig. 74A, B).

**Minor workers.** Unknown.

###### Etymology.

Malagasy for a soldier, in reference to the fact that this species is known only from its major workers.

###### Biology.

The species was collected at 785 m in elevation, in rainforest. Nesting preferences unknown.

###### Comments.

*Pheidole miramila* sp. nov. is most similar to *P. boribora* sp. nov. ***Major workers*.***Pheidole miramila* sp. nov. differs from *P. boribora* sp. nov. by absence of foveolae on head, sparse and short pilosity of sides of head, triangular inner hypostomal teeth, and propodeal spines moderately long. ***Minor workers*.** Unknown.

#### Revision of the *Pheidole masoala* group

**Diagnosis. *Major workers*.** Small species; head in full-face view sub-rectangular or oval, anterior and posterior sides of eyes relatively straight or convex; in lateral view sub-rectangular; ventral and dorsal faces relatively flat; dorsal face finely depressed posteriorly (except *Pheidole zavamanira*); antennal scrobes absent, indistinct or well developed; scrobe surface and lateral sides of head foveolate with or without additional sparse, thick, and irregular to longitudinal rugae or with thick, longitudinal, and long rugae with smooth to indistinctly rugulose interspaces; frons always with thick to thin, longitudinal rugae; occipital lobes with thick irregular rugae, interspaces smooth to rugulae; promesonotum relatively low and evenly angular; promesonotal groove absent; mesosoma never entirely smooth, most often foveolate with additional indistinct, irregular, short rugulae; gaster smooth to finely shagreened; body yellow to black. ***Minor workers*.** Head and mesosoma at least partly foveolate, sometimes with additional rugae; genae always with reduced sculpture or smooth; scape, when laid back, reaching the posterior head margin or surpassing it by one- to two-fifths of its length; promesonotum low, long, flat, or slightly convex, with relatively convex to steep posterior declivity; promesonotal groove absent; metanotal groove present; body yellow to dark brown.

**Comments.** Species of this group have small body size, in both major and minor workers. Major workers can be distinguished by a combination of the following characters: head in full-face and lateral views sub-rectangular or oval with flat ventral and dorsal faces; in most cases antennal scrobes, when present, are indistinctly to distinctly delimited; foveolate sculpture on the lateral sides of head, sometimes with additional rugae (except *P. lamperos*); frons always with longitudinal rugae and occipital lobes with irregular rugae; relatively low and evenly angular promesonotum; lack of promesonotal groove, and never entirely smooth mesosoma. Minor workers can be distinguished based on at least partly foveolate head and mesosoma, with smooth genae; short scape reaching the posterior head margin or surpassing it by at most two-fifths of its length; low, long, flat, or slightly convex promesonotum, with relatively convex to steep posterior declivity; and lack of promesonotal groove.

The group is divided into three complexes. The *P. masoala* complex contains five species: *P. masoala* sp. nov., *P. madinika* sp. nov., *P. fisaka* sp. nov., *P. binara* sp. nov., and *P. andapa* sp. nov. *Pheidole masoala* sp. nov. is widespread within evergreen forest but its distribution centre is located on the northern part of the biome. *Pheidole madinika* sp. nov. and *P. andapa* sp. nov. are sympatric and their distribution is limited to Parc National de Marojejy, Antsiranana. *Pheidole fisaka* sp. nov. is known from several sampling sites located in Ankerana, Toamasina. *Pheidole binara* sp. nov. was collected in two places in the Antsiranana prefecture: Kalabenono mountain and Binara Forest. *Pheidole lamperos* sp. nov. and *P. zavamanira* sp. nov. create a single-species complexes and both are known only from their type localities. *Pheidole lamperos* sp. nov. was sampled on the Galoko mountain and can be sympatric with *P. binara* known from another mountain of the Galoko chain. While *P. zavamanira* sp. nov. was sampled in several places in Réserve Spéciale d’Ambohijanahary, Toliara.

##### Key to the *Pheidole masoala* group

**Table:** 

1	Major workers. Antennal scrobes shiny, with thick, longitudinal, and long rugae; interspaces smooth to indistinctly rugulose; promesonotum, katepisternum, anepisternum, and dorsoventral surface of propodeum smooth, with very indistinct, short irregular rugulae (Fig. 75K, L). Minor workers. Head and mesosoma with sparse but distinct foveolae and smooth interspaces, vertex with additional arcuate rugae and promesonotum with additional transverse rugae (Fig. 76K, L)	***P. lamperos* sp. nov.**
–	Major workers. Antennal scrobes absent or predominately foveolate with additional fine rugae; promesonotum predominately foveolate (Fig. 75A–J). Minor workers. Head and mesosoma with dense foveolae, vertex never with additional arcuate rugae and promesonotum never with additional transverse rugae (Fig. 76F–J)	**2**
2	Major workers. Head in full-face view oval, antennal scrobes absent; lateral sides of head shiny, foveolate with a few thin, irregular to longitudinal short rugae (Fig. 75M, N). Minor workers. Head foveolate, frons with a few indistinct, short, longitudinal rugulae (Fig. 76M, N)	***P. zavamanira* sp. nov.**
–	Major workers. Head in full-face view sub-rectangular, antennal scrobes present; lateral sides of head shiny, foveolate with a fine to thick, irregular to longitudinal short rugae (Fig. 75A–J). Minor workers. Head foveolate, frons never with short, longitudinal rugulae (Fig. 76F–J)	**3**
3	Major workers. Body dark brown to black; gaster smooth or with very indistinctly shagreened first gastral tergite; propodeum with reduced sculpture and its lateral sides partially smooth (Figs 75G, J, 76B, E). Minor workers. Promesonotum in lateral view box-like, with posterior declivity steep (Fig. 76G, J)	**4**
–	Major workers. Body yellow to yellowish brown; gaster indistinctly shagreened; propodeum never with smooth notches on lateral sides (Figs 75F, H–I, 76A, C, D). Minor workers. Promesonotum in lateral view flat or slightly convex, with relatively convex posterior declivity (Fig. 76F, H, I)	**5**
4	Major workers. Body dark brown; sides of head with moderately dense, moderately long, erect pilosity; antennal scrobes indistinctly delimited by carinulae; gaster smooth (Figs 75B, G, 76B). Minor workers. Katepisternum foveolate with smooth notch; body yellow (Fig. 76G)	***P. madinika* sp. nov.**
–	Major workers. Body black; antennal scrobes not delimited by carinulae; sides of head with sparse, long, erect pilosity; gaster smooth, only basal part of first gastral tergite indistinctly shagreened (Figs 75E, J, 76E). Minor workers. Katepisternum smooth; body dark yellow (Fig. 76J)	***P. andapa* sp. nov.**
5	Major workers. Antennal scrobes well developed, delimited ventrally and posteriorly by carinulae (Fig. 75A). Minor workers. Propodeal spines minute, katepisternum smooth, lateral sides of pronotum and propodeum with uniform foveolae (Fig. 76F)	***P. masoala* sp. nov.**
–	Major workers. Antennal scrobes not delimited or delimited indistinctly by carinulae (Fig. 75C, D). Minor workers. Propodeal spines small and katepisternum at least partially foveolate or propodeal spines minute and lateral sides of pronotum and propodeum with fading foveolae (Fig. 76H, –I)	**6**
6	Major workers. Frons with smooth surface between rugae, katepisternum smooth, propodeal dorsum with fading foveolae (Fig. 75C, H). Minor workers. Promesonotum short and convex, lateral sides of pronotum and propodeum with fading foveolae, katepisternum smooth, propodeal spines minute (Fig. 76H)	***P. fisaka* sp. nov.**
–	Major workers. Frons with surface between rugae distinctly rugo-foveolate, katepisternum never smooth, propodeal dorsum with uniform foveolae (Fig. 75D, I). Minor workers. Promesonotum long and slightly convex, mesosoma uniformly foveolate, propodeal spines small (Fig. 76I)	***P. binara* sp. nov.**

**Figure 75.**
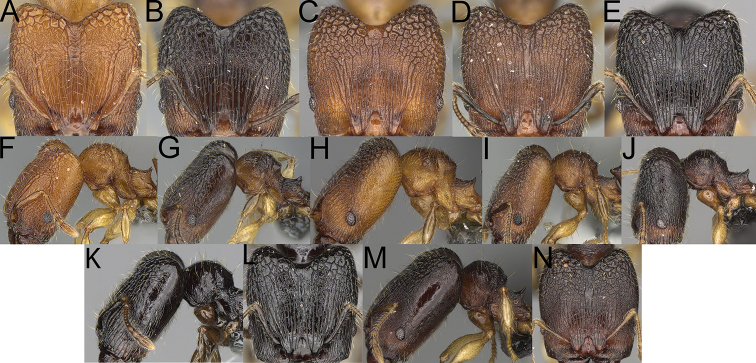
Major workers. *Pheidole masoala* sp. nov., head (**A**), profile (**F**). *Pheidole madinika* sp. nov., head (**B**), profile (**G**). *Pheidole fisaka* sp. nov., head (**C**), profile (**H**). *Pheidole binara* sp. nov., head (**D**), profile (**I**). *Pheidole andapa* sp. nov., head (**E**), profile (**J**). *Pheidole lamperos* sp. nov., profile (**K**), head (**L**). *Pheidole zavamanira* sp. nov., profile (**M**), head (**N**).

**Figure 76.**
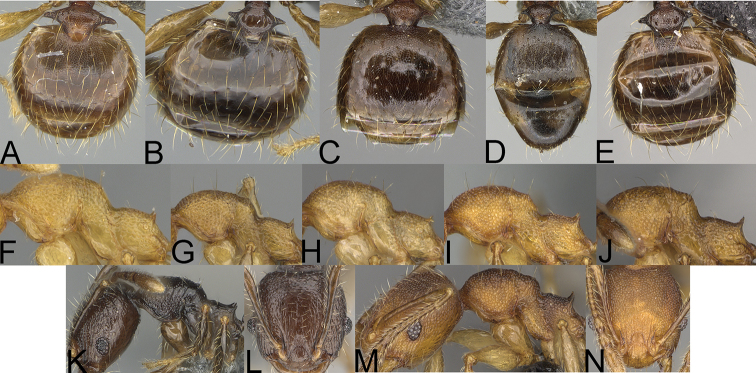
*Pheidole masoala* sp. nov., gaster of major worker (**A**), profile of minor worker (**F**). *Pheidole madinika* sp. nov., gaster of major worker (**B**), profile of minor worker (**G**). *Pheidole fisaka* sp. nov., gaster of major worker (**C**), profile of minor worker (**H**). *Pheidole binara* sp. nov., gaster of major worker (**D**), profile of minor worker (**I**). *Pheidole andapa* sp. nov., gaster of major worker (**E**), profile of minor worker (**J**). Minor workers. *Pheidole lamperos* sp. nov., profile (**K**), head (**L**). *Pheidole zavamanira* sp. nov., profile (**M**), head (**N**).

#### Revision of the *Pheidole masoala* complex

**Diagnosis. *Major workers*.** Head in full-face view sub-rectangular, anterior and posterior sides of eyes relatively straight or convex; in lateral view sub-rectangular; ventral and dorsal faces relatively flat; dorsal face finely depressed posteriorly; antennal scrobes indistinct or well developed; not delimited or delimited indistinctly to distinctly; scrobe surface foveolate, sometimes with additional sparse, thick, and irregular to longitudinal rugae; occipital lobes with interspaces smooth to rugulae; frons with moderately dense to dense, thick, and longitudinal rugae, interspaces smooth to rugo-foveolate; promesonotum moderately long; metanotal groove absent or present; propodeal spines small, triangular (only *P. andapa* sp. nov. has propodeal spines moderately long); mesosoma with fine foveolae, additional sparse and thin rugoreticulation on promesonotum or propodeum; gaster smooth to finely shagreened; body dark yellow to black. ***Minor workers*.** Head foveolate, genae with reduced sculpture or smooth; scape, when laid back, reaching the posterior head margin or surpassing it by one- to two-fifths of its length; promesonotum low, long, flat, or slightly convex, with relatively convex to steep posterior declivity (*P. andapa* sp. nov. has promesonotum short); propodeal spines minute to small, triangular; mesosoma foveolate, katepisternum smooth or with smooth notches (*P. binara* sp. nov. has katepisternum foveolate and promesonotum short); body yellow to dark yellow.

**Comments.** Major workers can be distinguished by a combination of the following characters: head in full-face and lateral views sub-rectangular with flat ventral and dorsal faces; in most cases antennal scrobes are indistinctly to distinctly delimited (not delimited in *P. fisaka* sp. nov. and *P. andapa* sp. nov.); scrobe surface foveolate, sometimes with additional sparse and thick rugae; small and triangular propodeal spines (only *P. andapa* sp. nov. has propodeal spines moderately long); finely foveolate mesosoma with additional sparse and thin rugoreticulation; smooth to finely shagreened gaster, and dark yellow to dark brown body. Minor workers can be distinguished based on foveolate head and mesosoma, with genae and katepisternum smooth or with reduced sculpture (*P. binara* sp. nov. has katepisternum foveolate); minute to small and triangular propodeal spines, and body yellow to dark yellow.

##### Pheidole masoala sp. nov.

Taxon classificationAnimaliaHymenopteraFormicidae

http://zoobank.org/170762F5-C1DE-4BF4-A551-B07B1C763053

**Figure 77.**
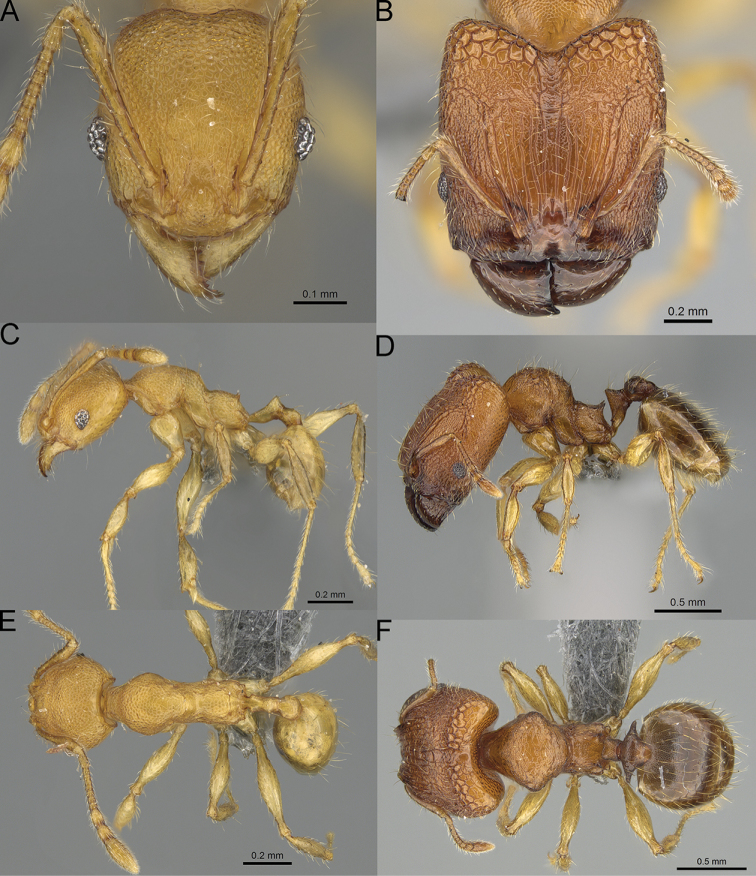
*Pheidole masoala* sp. nov., full-face view (**A**), profile (**C**), and dorsal view (**E**) of paratype minor worker (CASENT0923165) and full-face view (**B**), profile (**D**), and dorsal view (**F**) of holotype major worker (CASENT0375363).

Figs 77A–F , 85I , 87M

###### Type material.

***Holotype*.** Madagascar. •1 major worker; Antsiranana; Masoala National Park; -15.32331, 50.30751; alt. 60 m; 12 Mar 2014; B.L. Fisher et al. leg.; BLF32969, CASENT0375363 (CASC). ***Paratype*.** Madagascar. •1w.; same data as for holotype; CASENT0923165 (CASC).

###### Other material.

Madagascar. –***Antsiranana***: •2w., 1s.; 2.0 km S Andrakata; -14.65, 49.71667; alt. 520 m; 2 Dec 1994; B.L. Fisher leg.; CASENT0198553 (CASC). •3w., 5s., 1q., 1m.; Forêt Ambanitaza, 26.1 km 347° Antalaha; -14.67933, 50.18367; alt. 240 m; 26 Nov 2004; B.L. Fisher leg.; CASENT0054845, CASENT0054898, CASENT0055597, CASENT0109607, CASENT0109608, CASENT0109634, CASENT0109635 (CASC). •1w., 1s.; Masoala National Park; -15.3014, 50.22776; alt. 280 m; 7 Mar 2014; B.L. Fisher et al. leg.; CASENT0377564 (CASC). •1w., 1s.; Masoala National Park; -15.32331, 50.30751; alt. 60 m; 10 Mar 2014; B.L. Fisher et al. leg.; CASENT0375396 (CASC). –***Toamasina***: •2w., 4s.; Parc National Mananara-Nord, 7.1 km 261° Antanambe; -16.455, 49.7875; alt. 225 m; 15 Nov 2005; B.L. Fisher et al. leg.; CASENT0067475, CASENT0067490, CASENT0069439, CASENT0069448 (CASC). •1w., 1m.; Réserve Spéciale Ambatovaky, Sandrangato River; -16.7755, 49.26427; alt. 430 m; 24 Feb 2010; B.L. Fisher et al. leg.; CASENT0161802 (CASC). •1w., 1s.; Réserve Spéciale Ambatovaky, Sandrangato River; -16.77274, 49.26551; alt. 450 m; 20 Feb 2010; B.L. Fisher et al. leg.; CASENT0162680 (CASC). –***Toliara***: •1w., 1s.; Forêt Ivohibe 55.0 km N Tolagnaro; -24.569, 47.204; alt. 200 m; 3 Dec 2006; B.L. Fisher et al. leg.; CASENT0122620 (CASC).

###### Diagnosis.

***Major workers*.** Small species: HL: 0.93–1.17 (1.05), HW: 0.89–1.07 (0.97), WL: 0.76–0.92 (0.84); head in full-face view sub-rectangular, anterior and posterior sides of eyes relatively straight; sides of the head with sparse, moderately long, erect pilosity; antennal scrobes present, well delimited, and forming distinct dorsal concavity beneath frontal carina, scrobe surface foveolate, sometimes with additional sparse, thick, and irregular rugae, delimited ventrally and posteriorly by carinulae; inner hypostomal teeth distinct, big, closely spaced, triangular, with rounded apex and wide base; outer hypostomal teeth smaller and thinner than inner hypostomal teeth, with moderately wide base, triangular; gaster finely shagreened; body dark yellow. ***Minor workers*.** Head foveolate, genae with reduced sculpture or partially smooth; promesonotum low, long, flat or slightly convex, with relatively convex posterior declivity; propodeal spines minute, indistinct; mesosoma foveolate, katepisternum smooth.

###### Description.

**Major workers.** Measurements (*N* = 10): HL: 0.93–1.17 (1.05); HW: 0.89–1.07 (0.97); SL: 0.41–0.49 (0.45); EL: 0.13–0.16 (0.14); WL: 0.76–0.92 (0.84); PSL: 0.14–0.18 (0.16); MTL: 0.44–0.5 (0.45); PNW: 0.53–0.62 (0.58); PTW: 0.13–0.18 (0.15); PPW: 0.37–0.48 (0.43); CI: 90.8–95.9 (93.1); SI: 45.4–49.3 (46.7); PSLI: 13.4–16.2 (15.0); PPI: 31.9–37.5 (34.9); PNI: 56.7–63.7 (59.3); MTI: 44.9–49.1 (46.7). ***Head*.** In full-face view sub-rectangular, anterior and posterior sides of eyes relatively straight (Fig. 77B). In lateral view sub-rectangular; ventral and dorsal faces relatively flat; dorsal face finely depressed posteriorly, forming indistinct transverse depression between frons and occipital lobes; inner hypostomal teeth visible. Sides of the head with sparse, moderately long, erect pilosity; whole head with moderately dense, short, suberect to erect pilosity. Head depressed posteriorly, forming shallow transverse depression between frons and occipital lobes. Antennal scrobes distinct and delimited by carinulae; scrobe surface foveolate, sometimes with additional sparse, thick, and irregular rugae, delimited ventrally and posteriorly by carinulae. Occipital lobes shiny, with thick, sparse, irregular rugae, interspaces smooth; frons with dense, thick, and longitudinal rugae, interspaces smooth, and with fine but distinct foveolae on the upper half of frons; malar area with dense, thick, longitudinal to irregular rugulae, interspaces smooth; genae shiny, smooth to finely foveolate. Centre of clypeus smooth and shiny, lateral sides with longitudinal rugae; median notch present, wide and indistinct; median longitudinal carina absent; lateral longitudinal carinae present. Scape, when laid back, slightly exceeding midlength of head; pilosity suberect to erect (Fig. 77B, D). Inner hypostomal teeth distinct, big, closely spaced, triangular, with rounded apex and wide base; outer hypostomal teeth smaller and thinner than inner hypostomal teeth, with moderately wide base, triangular (Fig. 85I). ***Mesosoma*.** In lateral view, promesonotum moderately long, relatively low, and evenly angular, tubercle-like projections absent; promesonotal groove absent; metanotal groove absent; propodeal spines small, triangular, with acute apex; humeral area laterally weakly produced (Fig. 77D). Surface shiny, with fine foveolae, additional sparse and thin rugae on promesonotal dorsum and sometimes propodeum, sculpture slightly weakening on lateral surfaces of pronotum; katepisternum smooth. Pilosity sparse, moderately long, and erect (Fig. 77D, F). ***Petiole*.** Shiny and foveolate; peduncle short, with distinct horizontal lobes on its basal part; node relatively low, triangular, with rounded apex, in rear view node straight; pilosity moderately sparse and erect (Fig. 77D, F). ***Postpetiole*.** Shiny, with fine and sparse foveolae, smooth on dorsum; in dorsal view sides with long, acute, and triangular projections; pilosity long, moderately dense, and erect (Fig. 77D, F). ***Petiole*.** Shiny and finely shagreened; pilosity dense, moderately long, and erect (Fig. 77D, F). ***Colour*.** Unicolourous, dark yellow; lower part of malar area, frons, and gaster brown (Fig. 77D, F).

**Minor workers.** Measurements (*N* = 10): HL: 0.44–0.48 (0.46); HW: 0.39–0.43 (0.42); SL: 0.39–0.42 (0.4); EL: 0.09–0.1 (0.1); WL: 0.52–0.56 (0.54); PSL: 0.06–0.08 (0.07); MTL: 0.3–0.34 (0.32); PNW: 0.27–0.29 (0.27); PTW: 0.05–0.07 (0.06); PPW: 0.1–0.11 (0.1); CI: 88.5–95.2 (91.4); SI: 93.9–99.5 (97.3); PSLI: 13.3–16.5 (14.7); PPI: 50.5–64.6 (57.9); PNI: 63.8–68.5 (65.9); MTI: 73.4–80.0 (76.2). ***Head*.** Occipital margin straight or indistinctly concave; occipital carina absent (Fig. 77A). Pilosity moderately sparse, long, erect. Head foveolate, genae with reduced sculpture or partially smooth. Clypeus foveolate; median longitudinal carina absent; two lateral longitudinal carinae absent. Scape, when laid back, surpassing the posterior head margin by one-fifth of its length; pilosity suberect (Fig. 77A, C). ***Mesosoma*.** In lateral view, promesonotum low, long, flat or slightly convex, with relatively convex posterior declivity; promesonotal groove absent; metanotal groove indistinct; propodeal spines minute, indistinct, triangular, apex acute (Fig. 77C). Sculpture foveolate, katepisternum smooth. Pilosity moderately sparse, moderately short, and erect (Fig. 77C, E). ***Petiole*.** Peduncle very short and thin with ventral face slightly convex; with few short, erect setae (Fig. 77C, E). ***Postpetiole*.** Short, low, and slightly convex; with few short, erect setae (Fig. 77C, E). ***Petiole*.** With sparse, erect pilosity (Fig. 77C, E). ***Colour*.** Unicolourous, yellow (Fig. 77C, E).

###### Etymology.

From the type locality.

###### Biology.

The species was collected between 30–520 m in elevation, in rainforest and disturbed rainforest. Nests were located in rotten logs and sticks on the ground.

###### Comments.

*Pheidole masoala* sp. nov. is most similar to *P. madinika* sp. nov. ***Major workers*.***Pheidole masoala* sp. nov. differs from *P. madinika* sp. nov. in dark yellow body colouration, presence of shallow transverse depression between frons and occipital lobes, and well developed antennal scrobes which are delimited ventrally and posteriorly by carinulae. ***Minor workers*.***Pheidole masoala* sp. nov. differs from *P. madinika* sp. nov. by relatively convex posterior declivity of promesonotum, and shallow and indistinct metanotal groove.

##### Pheidole madinika sp. nov.

Taxon classificationAnimaliaHymenopteraFormicidae

http://zoobank.org/71B60910-DAC6-4F59-9391-CAC0112B6562

**Figure 78.**
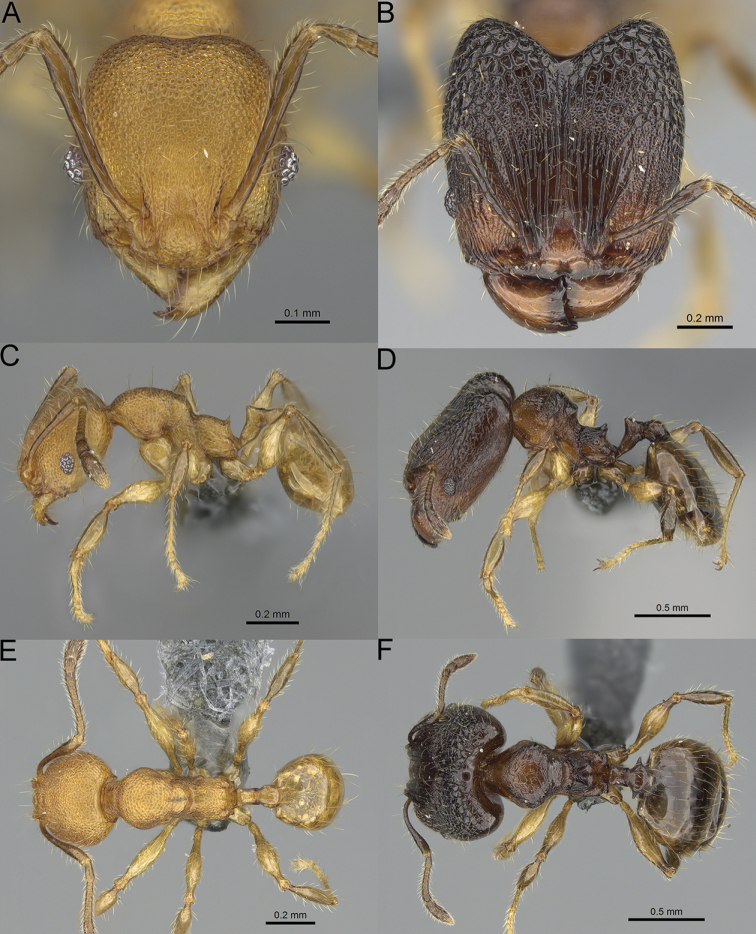
*Pheidole madinika* sp. nov., full-face view (**A**), profile (**C**), and dorsal view (**E**) of paratype minor worker (CASENT0923166) and full-face view (**B**), profile (**D**), and dorsal view (**F**) of holotype major worker (CASENT0808089).

Figs 78A–F , 84A , 87D

###### Type material.

***Holotype*.** Madagascar. •1 major worker; Antsiranana; Sava Region: Parc National de Marojejy, Manantenina River, 27.9 km 24.3°NE Andapa; -14.43462, 49.75853; alt. 850 m; 9 Feb 2018; B.L. Fisher et al. leg.; BLF40907, CASENT0808089 (CASC). ***Paratypes*.** Madagascar. •2w., 1m.; same data as for holotype; CASENT0808090, CASENT0923166, CASENT0872173 (CASC).

###### Other material.

Madagascar. –***Antsiranana***: •1w., 2s.; Parc National de Marojejy, Manantenina River, 27.6 km 35°NE Andapa, 9.6 km 327°NNW Manantenina; -14.435, 49.76; alt. 775 m; 15 Nov 2003; B.L. Fisher et. al. leg.; CASENT0494713, CASENT0494715 (CASC).

###### Diagnosis.

***Major workers*.** Small species: HL: 0.98–1.06 (1.02), HW: 0.91–0.94 (0.92, WL: 0.73–0.83 (0.78); head in full-face view sub-rectangular, anterior and posterior sides of eyes relatively convex; sides of head with sparse, moderately long, erect pilosity; antennal scrobes present, weakly impressed, and indistinctly delimited, scrobe surface shiny, foveolate with sparse, thick, longitudinal rugulae; inner hypostomal teeth distinct, moderately high, closely spaced, triangular, with rounded apex and wide base; outer hypostomal teeth smaller and thinner than inner hypostomal teeth, with moderately wide base, lobe-like; gaster smooth; body dark brown. ***Minor workers*.** Head foveolate, genae with reduced sculpture or partially smooth; promesonotum low, long, flat or slightly convex, with steep posterior declivity; propodeal spines small; mesosoma foveolate, katepisternum foveolate with smooth notch.

###### Description.

**Major workers.** Measurements (*N* = 3): HL: 0.98–1.06 (1.02); HW: 0.91–0.94 (0.92); SL: 0.41–0.45 (0.43); EL: 0.11–0.14 (0.12); WL: 0.73–0.83 (0.78); PSL: 0.13–0.16 (0.14); MTL: 0.42–0.46 (0.44); PNW: 0.45–0.51 (0.48); PTW: 0.12–0.14 (0.13); PPW: 0.28–0.35 (0.31); CI: 88.8–92.3 (90.3); SI: 45.6–48.4 (47.0); PSLI: 12.7–14.7 (13.7); PPI: 39.3–43.3 (41.7); PNI: 49.7–53.9 (51.5); MTI: 46.4–48.8 (47.8). ***Head*.** In full-face view sub-rectangular, anterior and posterior sides of eyes relatively convex (Fig. 78B). In lateral view sub-rectangular; ventral and dorsal faces relatively flat; dorsal face finely depressed posteriorly, forming indistinct transverse depression between frons and occipital lobes; inner hypostomal teeth invisible. Sides of the head with moderately dense, moderately long, erect pilosity; whole head with dense, short, suberect to erect pilosity. Antennal scrobes present, indistinctly delimited by carinulae; scrobe surface shiny, foveolate with sparse, thick, longitudinal rugulae. Occipital lobes shiny, with thick, sparse, irregular rugae, interspaces smooth; frons with dense, thick, and longitudinal rugae, interspaces smooth, and with fine but distinct foveolae on the upper half of frons; malar area with dense, thick, longitudinal to irregular rugulae, interspaces foveolate; genae shiny, with fine rugulae. Centre of clypeus smooth and shiny, lateral sides with longitudinal rugae; median notch present, wide, and indistinct; median longitudinal carina absent; lateral longitudinal carinae present. Scape, when laid back, slightly exceeding the midlength of head; pilosity suberect to erect (Fig. 78B, D). Inner hypostomal teeth distinct, moderately high, closely spaced, triangular, with rounded apex and wide base; outer hypostomal teeth smaller and thinner than inner hypostomal teeth, with moderately wide base, lobe-like (Fig. 85A). ***Mesosoma*.** In lateral view, promesonotum moderately long, relatively low and convex, posterior mesonotum steep, with small, tubercle-like projections; promesonotal groove absent; metanotal groove present; propodeal spines small, triangular, with rounded apex; humeral area laterally weakly produced (Fig. 78D). Surface shiny, with fine and sparse foveolae, additional sparse and thin rugae on promesonotal dorsum and sometimes propodeum, sculpture slightly weakening on dorsal and lateral surfaces of pronotum; katepisternum and lower parts of lateral surfaces of propodeum smooth. Pilosity sparse, long and subdecumbent to erect (Fig. 78D, F). ***Petiole*.** Shiny and foveolate; peduncle short, without horizontal lobes on its basal part; node relatively high, triangular, with rounded apex, in rear view node straight; pilosity moderately sparse and erect (Fig. 78D, F). ***Postpetiole*.** Shiny, with fine and sparse foveolae, smooth on dorsum; in dorsal view sides with short, acute, and triangular projections; pilosity long, moderately sparse, and erect (Fig. 78D, F). ***Petiole*.** Shiny and smooth; pilosity sparse, moderately long, and erect (Fig. 78D, F). ***Colour*.** Unicolourous, dark brown; lower part of lateral sides of mesosoma, malar area and frons yellowish brown (Fig. 78D, F).

**Minor workers.** Measurements (*N* = 3): HL: 0.42–0.48 (0.45); HW: 0.38–0.42 (0.4); SL: 0.4–0.42 (0.41); EL: 0.08–0.09 (0.08); WL: 0.49–0.53 (0.51); PSL: 0.07–0.07 (0.07); MTL: 0.3–0.32 (0.31); PNW: 0.26–0.26 (0.26); PTW: 0.06–0.07 (0.06); PPW: 0.08–0.1 (0.09); CI: 87.1–90.7 (89.2); SI: 100.5–105.2 (102.3); PSLI: 15.4–16.0 (15.7); PPI: 63.7–70.4 (67.5); PNI: 62.8–68.8 (65.7); MTI: 75.75–79.5 (77.1). ***Head*.** Occipital margin straight or indistinctly concave; occipital carina absent (Fig. 78A). Pilosity moderately dense, long, erect. Head foveolate, genae with reduced sculpture or partially smooth. Clypeus foveolate; median longitudinal carina absent; two lateral longitudinal carinae absent. Scape, when laid back, surpassing the posterior head margin by one-fifth of its length; pilosity suberect (Fig. 78A, C). ***Mesosoma*.** In lateral view, promesonotum low, long, flat or slightly convex, with steep posterior declivity; promesonotal groove absent; metanotal groove distinct; propodeal spines small, indistinct, triangular, apex acute (Fig. 78C). Sculpture foveolate, katepisternum foveolate with smooth notch. Pilosity sparse, long, and erect (Fig. 78C, E). ***Petiole*.** Peduncle short and thin with ventral face slightly convex; node low, triangular and small; with few short, erect setae (Fig. 78C, E). ***Postpetiole*.** Short, low, and slightly convex; with few short, erect setae (Fig. 78C, E). ***Petiole*.** With sparse, erect pilosity (Fig. 78C, E). ***Colour*.** Unicolourous, yellow (Fig. 78C, E).

###### Etymology.

Malagasy for small, in reference to small body size.

###### Biology.

The species was collected between 775–850 in elevation, in rainforest. Nests were located in rotten logs.

###### Comments.

*Pheidole madinika* sp. nov. is most similar to *P. masoala* sp. nov. ***Major workers*.***Pheidole madinika* sp. nov. differs from *P. masoala* sp. nov. in dark brown body colouration, absence of transverse depression between frons and occipital lobes, and weakly impressed and not distinctly delimited antennal scrobes. ***Minor workers*.***Pheidole madinika* sp. nov. differs from *P. masoala* sp. nov. by steep posterior declivity of promesonotum, and relatively deep and distinct metanotal groove.

##### Pheidole fisaka sp. nov.

Taxon classificationAnimaliaHymenopteraFormicidae

http://zoobank.org/2BA075C8-7EA3-444A-B35F-CE9BA0DC0798

**Figure 79.**
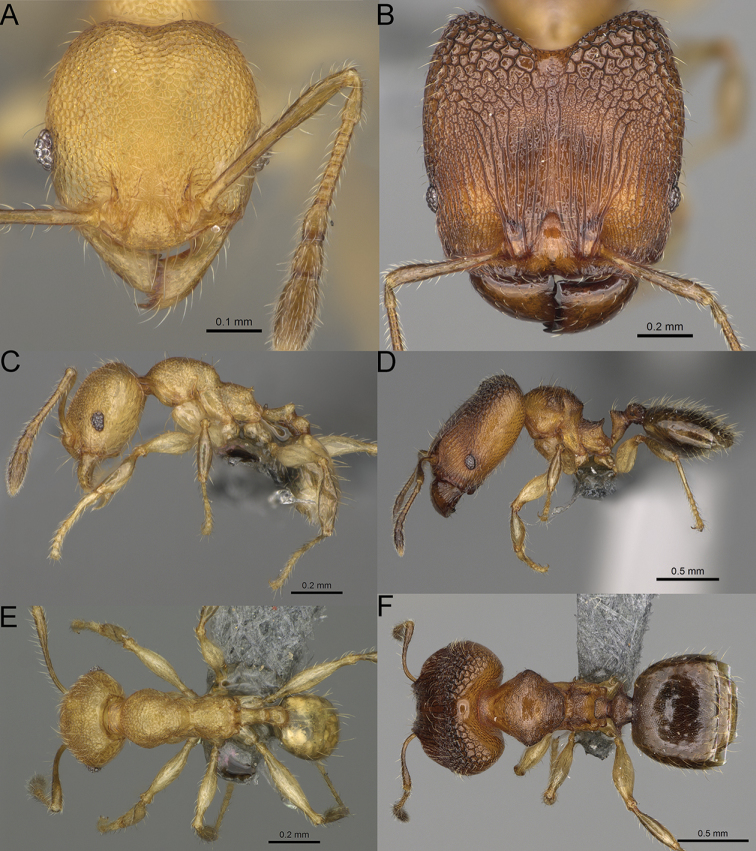
*Pheidole fisaka* sp. nov., full-face view (**A**), profile (**C**), and dorsal view (**E**) of paratype minor worker (CASENT0923180) and full-face view (**B**), profile (**D**), and dorsal view (**F**) of holotype major worker (CASENT0274930).

Figs 79A–F , 84R , 86R

###### Type material.

***Holotype*.** Madagascar. •1 major worker; Toamasina; Ankerana; -18.40062, 48.81311; alt. 865 m; 17 Jan 2012; B.L. Fisher et al. leg.; CASENT0274930 (CASC). ***Paratype*.** Madagascar. •1w.; same data as for holotype; BLF27851, CASENT0923180 (CASC).

###### Other material.

Madagascar. –***Toamasina***: •1w., 1s.; Ankerana; -18.4061, 48.82029; alt. 725 m; 16 Jan 2012; B.L. Fisher et al. leg.; CASENT0273490, CASENT0273492 (CASC). •2w., 1m.; Ankerana; -18.40829, 48.82107; alt. 750 m; 21 Jan 2012; B.L. Fisher et al. leg.; CASENT0275257, CASENT0275258 (CASC). •3w., 1q.; Ankerana; -18.4104, 48.8189; alt. 855 m; 27 Jan 2012; B.L. Fisher et al. leg.; CASENT0274031, CASENT0274032, CASENT0274033 (CASC).

###### Diagnosis.

***Major workers*.** Small species: HL: 1.01–1.0; HW: 0.99–0.98, WL: 0.8–0.76; head in full-face view sub-rectangular, anterior of eyes straight, posterior of eyes convex; sides of the head with moderately dense, short, erect pilosity; antennal scrobes indistinct and not delimited by carinulae; scrobe surface shiny, foveolate with thick, longitudinal to irregular, and long rugae; inner hypostomal teeth distinct, moderately high, closely spaced, triangular, with rounded apex and wide base; outer hypostomal teeth thinner and narrower than inner hypostomal teeth, dentate; gaster finely shagreened; body yellowish brown. ***Minor workers*.** Head foveolate, genae smooth; promesonotum low, convex, short, with posterior declivity smoothly declining towards propodeum; propodeal spines minute; mesosoma foveolate, lateral sides of pronotum and propodeum with fading sculpture, katepisternum smooth.

###### Description.

**Major workers.** Measurements (*N* = 2): HL: 1.01–1.0; HW: 0.99–0.98; SL: 0.44–0.45; EL: 0.13–0.13; WL: 0.8–0.76; PSL: 0.17–0.14; MTL: 0.440.44; PNW: 0.58–0.54; PTW: 0.16–0.14; PPW: 0.39–0.35; CI: 98.5–97.7; SI: 44.5–46.2; PSLI: 17.1–13.6; PPI: 40.6–40.5; PNI: 58.0–55.0; MTI: 44.6–45.3. ***Head*.** In full-face view longer than wide, anterior of eyes straight, posterior of eyes convex; slightly widening posteriorly (Fig. 79B). In lateral view sub-rectangular; ventral and dorsal faces relatively flat; dorsal face finely depressed posteriorly, forming shallow, transverse depression between frons and occipital lobes; inner hypostomal teeth visible. Sides of the head with moderately dense, short, erect pilosity; whole head with dense, short, suberect to erect pilosity. Antennal scrobes indistinct and not delimited by carinulae; scrobe surface shiny, foveolate with thick, longitudinal to irregular, and long rugae. Occipital lobes shiny, with thick, irregular rugae, interspaces with distinct, irregular rugulae; frons with moderately dense, thick and longitudinal rugae, interspaces smooth to rugo-foveolate in the posterior part; genae shiny, with dense and thin, irregular rugoreticulation; malar area with thin, longitudinal, dense rugoreticulation. Centre of clypeus shiny and smooth, lateral sides with longitudinal rugulae; median notch present, wide, and shallow; median longitudinal carina absent; lateral longitudinal carinae present. Scape, when laid back, reaching the midlength of head; pilosity suberect (Fig. 79B, D). Inner hypostomal teeth distinct, moderately high, closely spaced, triangular, with rounded apex and wide base; outer hypostomal teeth thinner and narrower than inner hypostomal teeth, dentate (Fig. 84R). ***Mesosoma*.** In lateral view, promesonotum low and arched, posterior mesonotum steep, without tubercle-like projections; promesonotal groove absent; metanotal groove absent; propodeal spines short, triangular, with rounded apex and wide base; humeral area laterally well produced (Fig. 79D). Surface shiny, with fine and moderately dense foveolae and additional rugoreticulation on the lateral sides of propodeum; dorsal surface of promesonotum with fading foveolae; katepisternum smooth. Pilosity moderately dense and long, erect (Fig. 79D, F). ***Petiole*.** Shiny and with fine foveolae; peduncle short, with indistinct horizontal lobes on its basal part; node relatively high, triangular, with rounded apex, in rear view node slightly dorsoventrally concave; pilosity moderately sparse and erect (Fig. 79D, F). ***Postpetiole*.** Shiny, finely shagreened; in dorsal view sides with moderately long, acute, and triangular projections; pilosity long, moderately long, and erect (Fig. 79D, F). ***Petiole*.** Shiny and finely shagreened; pilosity dense, moderately long, and erect (Fig. 79D, F). ***Colour*.** Yellow; frons, occipital lobes; dorsal surface of mesosoma and gaster yellowish brown to brown (Fig. 79D, F).

**Minor workers.** Measurements (*N* = 2): HL: 0.43–0.43; HW: 0.4–0.39; SL: 0.38–0.37; EL: 0.09–0.08; WL: 0.48–0.48; PSL: 0.07–0.06; MTL: 0.28–0.29; PNW: 0.25–0.25; PTW: 0.06–0.06; PPW: 0.09–0.09; CI: 92.9–91.8; SI: 95.0–93.1; PSLI: 15.2–14.5; PPI: 68.5–63.4; PNI: 62.3–63.9; MTI: 70.5–73.0. ***Head*.** Occipital margin straight or indistinctly concave; occipital carina absent (Fig. 79A). Pilosity moderately dense, short, suberect to erect. Head foveolate; genae smooth. Clypeus foveolate; median longitudinal carina absent; absence of two lateral longitudinal carinae. Scape, when laid back, reaching the posterior head margin; pilosity erect (Fig. 79A, C). ***Mesosoma*.** In lateral view, promesonotum low, convex, short, with posterior declivity smoothly declining towards propodeum; promesonotal groove absent; metanotal groove indistinct; propodeal spines minute, triangular, with acute apex (Fig. 79C). Sculpture foveolate; lateral sides of pronotum and propodeum with fading sculpture; katepisternum smooth. Pilosity moderately sparse, moderately long, and erect (Fig. 79C, E). ***Petiole*.** Peduncle very short and thin; with few short, erect setae (Fig. 79C, E). ***Postpetiole*.** Short, low and convex; with moderately short, erect setae (Fig. 79C, E). ***Petiole*.** With sparse, erect pilosity (Fig. 79C, E). ***Colour*.** Yellow (Fig. 79C, E).

###### Etymology.

Malagasy for flat, in reference for shape of the head in major workers.

###### Biology.

The species was collected between 725–865 m in elevation, in rainforest. Nests were located in rotten logs.

###### Comments.

*Pheidole fisaka* sp. nov. is most similar to *P. binara* sp. nov. ***Major workers*.***Pheidole fisaka* sp. nov. differs from *P. binara* sp. nov. in smooth surface between rugae on frons and katepisternum and fading foveolae on propodeal dorsum. ***Minor workers*.***Pheidole fisaka* sp. nov. differs from *P. binara* sp. nov. in shorter and more convex promesonotum, and lateral sides of pronotum and propodeum with fading foveolae and smooth katepisternum.

##### Pheidole binara sp. nov.

Taxon classificationAnimaliaHymenopteraFormicidae

http://zoobank.org/C7378E6D-D708-4D9A-B696-18052069BFE1

**Figure 80.**
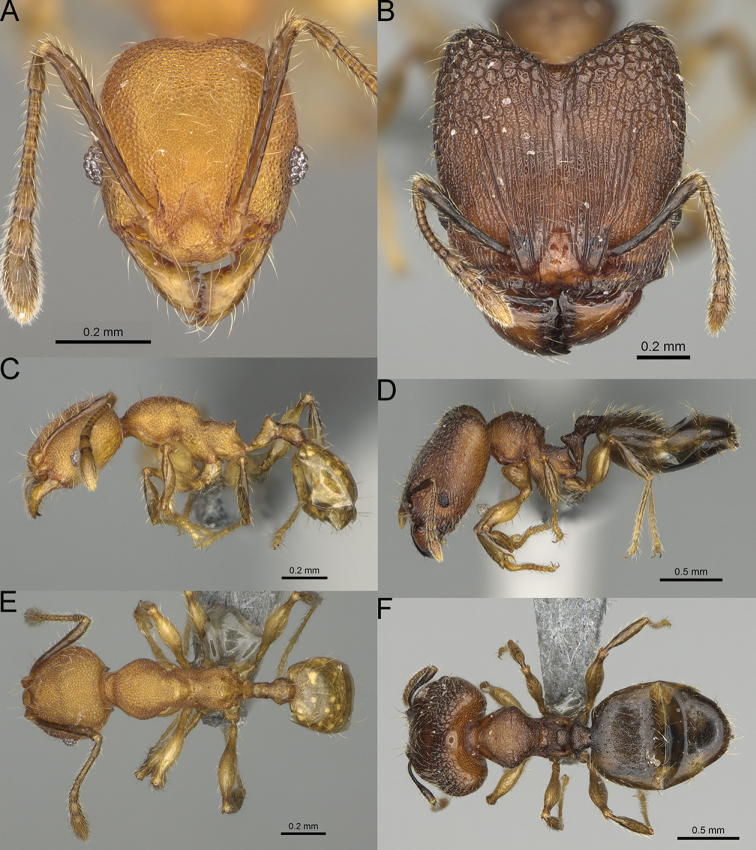
*Pheidole binara* sp. nov., full-face view (**A**), profile (**C**), and dorsal view (**E**) of paratype minor worker (CASENT0923182) and full-face view (**B**), profile (**D**), and dorsal view (**F**) of holotype major worker (CASENT0353299).

Figs 80A–F , 84I , 86I

###### Type material.

***Holotype*.** Madagascar. •1 major worker; Antsiranana; Binara forest; -13.26388, 49.60141; alt. 500 m; 19 Sep 2013; B.L. Fisher et al. leg.; BLF32213, CASENT0353299 (CASC). ***Paratypes*.** Madagascar. •1w.; same data as for holotype; CASENT0923182 (CASC).

###### Diagnosis.

***Major workers*.** Small species: HL: 1.03, HW: 0.97, WL: 0.8; head in full-face view sub-rectangular, anterior of eyes straight, posterior of eyes convex; sides of the head with moderately sparse, long, erect pilosity; antennal scrobes present and indistinctly delimited ventrally and posteriorly by carinulae, scrobe surface shiny, foveolate with thick, longitudinal to irregular, and long rugae; inner hypostomal teeth distinct, moderately high, closely spaced, triangular, with rounded apex and wide base; outer hypostomal teeth thinner, smaller, and narrower than inner hypostomal teeth, dentate; gaster finely shagreened; body yellowish brown. ***Minor workers*.** Head foveolate, genae with fading sculpture but never smooth; promesonotum low, moderately convex, short, with relatively convex posterior declivity; propodeal spines short; mesosoma foveolate.

###### Description.

**Major workers.** Measurements (*N* = 1): HL: 1.03; HW: 0.97; SL: 0.49; EL: 0.14; WL: 0.8; PSL: 0.15; MTL: 0.48; PNW: 0.52; PTW: 0.14; PPW: 0.37; CI: 93.8; SI: 50.8; PSLI: 14.4; PPI: 38.6; PNI: 53.9; MTI: 49.3. ***Head*.** In full-face view longer than wide, anterior of eyes straight, posterior of eyes slightly convex (Fig. 80B). In lateral view sub-rectangular; ventral and dorsal faces relatively flat; dorsal face finely depressed posteriorly, forming shallow transverse depression between frons and occipital lobes; inner hypostomal teeth invisible. Sides of head with moderately sparse, long, erect pilosity; whole head with dense, short, suberect to erect pilosity. Antennal scrobes indistinct and indistinctly delimited ventrally and posteriorly by carinulae; scrobe surface shiny, foveolate with thick, longitudinal to irregular, and long rugae. Occipital lobes shiny, with thick, irregular rugae, interspaces with distinct, irregular rugulae; frons with moderately dense, thick, and longitudinal rugae, interspaces distinctly rugo-foveolate; genae shiny, with dense and thin, irregular rugoreticulation; malar area with thin, longitudinal, dense rugoreticulation. Centre of clypeus shiny and smooth, lateral sides with longitudinal rugulae; median notch present, wide, and moderately deep; median longitudinal carina present; lateral longitudinal carinae present. Scape, when laid back, slightly surpassing the midlength of head; pilosity suberect (Fig. 80B, D). Inner hypostomal teeth distinct, moderately high, closely spaced, triangular, with rounded apex and wide base; outer hypostomal teeth thinner, smaller, and narrower than inner hypostomal teeth, dentate (Fig. 84I). ***Mesosoma*.** In lateral view, promesonotum relatively low and arched, posterior mesonotum steep, without tubercle-like projections; promesonotal groove absent; metanotal groove absent; propodeal spines short, triangular, with rounded apex and wide base; humeral area laterally well produced (Fig. 80D). Surface shiny, foveolate, and with additional short, transverse to irregular, thick rugae on the dorsal surface of propodeum. Pilosity sparse, very long, and erect (Fig. 80D, F). ***Petiole*.** Shiny and with fine foveolae; peduncle short, with indistinct horizontal lobes on its basal part; node relatively high, triangular, with rounded apex, in rear view node slightly convex; pilosity long and erect (Fig. 80D, F). ***Postpetiole*.** Shiny, finely shagreened; in dorsal view sides with moderately short, acute, and triangular projections; pilosity long and erect (Fig. 80D, F). ***Petiole*.** Shiny and finely shagreened; pilosity dense, moderately long, and erect (Fig. 80D, F). ***Colour*.** Yellowish brown; frons, occipital lobes; dorsal surface of mesosoma and gaster brown (Fig. 80D, F).

**Minor workers.** Measurements (*N* = 1): HL: 0.48; HW: 0.44; SL: 0.43; EL: 0.09; WL: 0.58; PSL: 0.09; MTL: 0.34; PNW: 0.29; PTW: 0.07; PPW: 0.13; CI: 90.3; SI: 99.1; PSLI: 18.8; PPI: 53.5; PNI: 66.8; MTI: 77.1. ***Head*.** Occipital margin straight or indistinctly concave; occipital carina indistinct (Fig. 80A). Pilosity moderately sparse, moderately long, suberect to erect. Head foveolate; genae with fading sculpture but never smooth. Clypeus foveolate; median longitudinal carina absent; two lateral longitudinal carinae absent. Scape, when laid back, surpassing the posterior head margin by one-fifth of its length; pilosity erect (Fig. 80A, C). ***Mesosoma*.** In lateral view, promesonotum low, moderately convex, short, with relatively convex posterior declivity; promesonotal groove absent; metanotal groove indistinct; propodeal spines short, triangular, with acute apex (Fig. 80C). Sculpture foveolate. Pilosity moderately sparse, long, and erect (Fig. 80C, E). ***Petiole*.** Peduncle short and thin; with few short, erect setae (Fig. 80C, E). ***Postpetiole*.** Moderately short, low, and convex; with few short, erect setae (Fig. 80C, E). ***Petiole*.** With sparse, erect pilosity (Fig. 80C, E). ***Colour*.** Yellow (Fig. 80C, E).

###### Etymology.

From the type locality.

###### Biology.

The species was collected between 498–900 m in elevation, in rainforest. Nests were located in rotten logs.

###### Comments.

*Pheidole binara* sp. nov. is most similar to *P. masoala* sp. nov. and *P. fisaka* sp. nov. ***Major workers*.***Pheidole binara* sp. nov. differs from *P. masoala* sp. nov. in absence of shallow transverse depression between frons and occipital lobes, weakly developed antennal scrobes which are not delimited ventrally and posteriorly by carinulae, presence of distinct sculpture between thick rugae on frons and occipital lobes, and never smooth katepisternum; from *P. fisaka* sp. nov. in never smooth surface between rugae on frons, never smooth katepisternum, and distinct foveolation on propodeal dorsum. ***Minor workers*.***Pheidole binara* sp. nov. differs from *P. masoala* sp. nov. in never smooth sculpture on frons and katepisternum; from *P. fisaka* sp. nov. in longer and lower promesonotum, and uniformly foveolate mesosoma sculpture.

##### Pheidole andapa sp. nov.

Taxon classificationAnimaliaHymenopteraFormicidae

http://zoobank.org/2B1D7901-048D-4E99-8808-83C790980FCA

**Figure 81.**
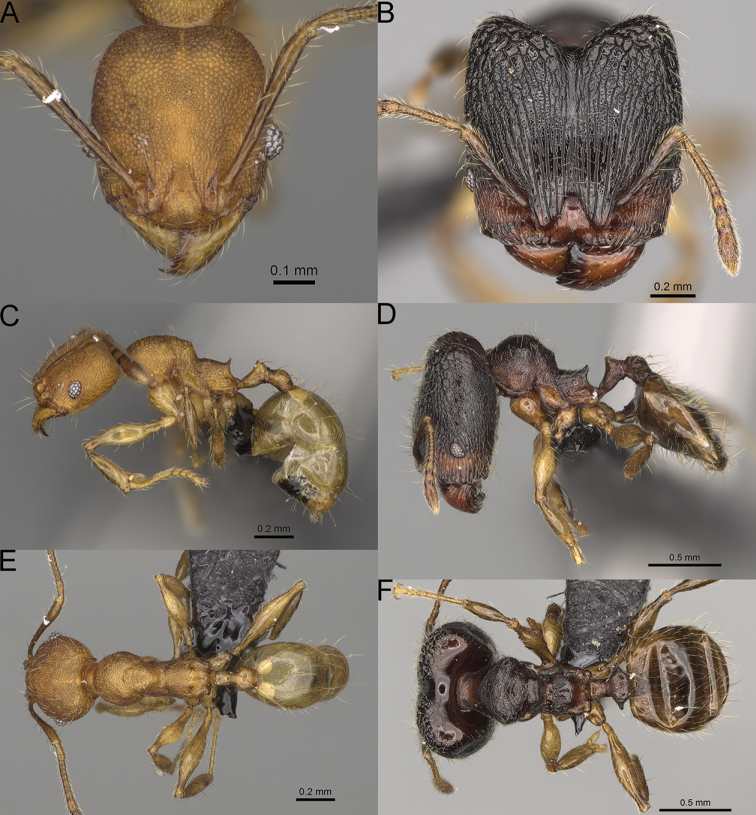
*Pheidole andapa* sp. nov., full-face view (**A**), profile (**C**), and dorsal view (**E**) of paratype minor worker (CASENT0923178) and full-face view (**B**), profile (**D**), and dorsal view (**F**) of holotype major worker (CASENT0068047).

Figs 81A–F , 84C , 86C

###### Type material.

***Holotype*.** Madagascar. •1 major worker; Antsiranana; Parc National de Marojejy, Antranohofa, 26.6 km 31°NNE Andapa, 10.7 km 318°NW Manantenina; -14.44333, 49.74333; alt. 1325 m; 14 Dec 2005; B.L. Fisher et al. leg.; BLF13640, CASENT0068047 (CASC). ***Paratypes*.** Madagascar. •2w., 1s.; same data as for holotype; CASENT0068048, CASENT0923178, CASENT0872204 (CASC).

###### Diagnosis.

***Major workers*.** Head in full-face view sub-rectangular, anterior of eyes straight, posterior of eyes convex; sides of the head with sparse, long, erect pilosity; scrobe surface shiny, foveolate with moderately dense, thick, longitudinal rugae; inner hypostomal teeth distinct, closely spaced, moderately high, triangular, with rounded apex; outer hypostomal teeth thinner and approximately as high as outer hypostomal teeth, triangular, and with relatively wide base; propodeal spines moderately long, with base slightly wider than top; gaster smooth; body black. ***Minor workers*.** Head foveolate, genae smooth; promesonotum low, slightly convex, short, with posterior declivity steep; mesosoma foveolate; katepisternum and mesosoma smooth; propodeal spines very short, triangular; body dark yellow.

###### Description.

**Major workers.** Measurements (*N* = 2): HL: 1.07–1.23; HW: 0.98–1.12; SL: 0.47–0.49; EL: 0.12–0.13; WL: 0.86–0.89; PSL: 0.16–0.17; MTL: 0.48–0.5; PNW: 0.49–0.59; PTW: 0.15–0.15; PPW: 0.35–0.43; CI: 91.8–91.0; SI: 48.3–44.2; PSLI: 15.0–13.6; PPI: 42.4–35.6; PNI: 50.3–52.7; MTI: 48.8–44.8. ***Head*.** In full-face view sub-rectangular, anterior of eyes straight, posterior of eyes convex (Fig. 81B). In lateral view sub-rectangular; ventral and dorsal faces relatively flat; dorsal face finely depressed posteriorly, forming indistinct transverse depression between frons and occipital lobes; inner hypostomal teeth visible. Sides of the head with sparse, long, erect pilosity; whole head with moderately sparse, short, suberect to erect pilosity. Antennal scrobes very indistinct and not delimited by carinulae; scrobe surface shiny, foveolate with moderately dense, thick, longitudinal rugae. Occipital lobes shiny, with indistinct foveolae and sparse, thick, irregular rugae, foveolae fading posteriorly; frons, on the anterior part, with dense, thick and longitudinal rugae and interspaces smooth, posterior part with rugae longitudinal and interspaces foveolate; genae shiny, with dense and thin rugulae; malar area with thick, dense rugo-foveolae. Centre of clypeus smooth and shiny, lateral sides with longitudinal rugae; median notch present, moderately wide, and shallow; median longitudinal carina absent; lateral longitudinal carinae present. Scape, when laid back, reaching the midlength of head; pilosity suberect to erect (Fig. 81B, D). Inner hypostomal teeth distinct, closely spaced, moderately high, triangular, with rounded apex; outer hypostomal teeth thinner and approximately as high as outer hypostomal teeth, triangular, and with relatively wide base (Fig. 84C). ***Mesosoma*.** In lateral view, promesonotum relatively low and convex, dorsal mesonotum slightly convex, posterior mesonotum steep, with small tubercle-like projections; promesonotal groove absent; metanotal groove absent; propodeal spines moderately long, triangular, with rounded apex and wide base; humeral area laterally well produced (Fig. 81D). Surface shiny, with fine and dense foveolae; promesonotal dorsum with additional indistinct, transverse, short rugulae; katepisternum and lower half of lateral surfaces of propodeum smooth. Pilosity moderately sparse, long, and erect (Fig. 81D, F). ***Petiole*.** Shiny and with fine foveolae; peduncle moderately short, with indistinct horizontal lobes on its basal part; node relatively high, triangular, with rounded apex, in rear view node dorsoventrally concave; pilosity long and erect (Fig. 81D, F). ***Postpetiole*.** Shiny, finely shagreened; in dorsal view sides with short, acute, and triangular projections; pilosity long and erect (Fig. 81D, F). ***Petiole*.** Shiny and smooth; only basal part of first gastral tergite indistinctly shagreened; pilosity moderately sparse, long, and erect (Fig. 81D, F). ***Colour*.** Black; lateral sides of mesosoma and malar area reddish brown; legs dark yellow (Fig. 81D, F).

**Minor workers.** Measurements (*N* = 2): HL: 0.49–0.5; HW: 0.44–0.43; SL: 0.46–0.43; EL: 0.11–0.1; WL: 0.64–0.61; PSL: 0.09–0.08; MTL: 0.37–0.36; PNW: 0.31–0.3; PTW: 0.06–0.06; PPW: 0.14–0.12; CI: 90.2–86.9; SI: 104.1–98.8; PSLI: 18.8–16.0; PPI: 44.8–50.0; PNI: 69.5–70.0; MTI: 83.9–84.4. ***Head*.** Occipital margin straight or indistinctly concave; occipital carina absent (Fig. 81A). Pilosity moderately sparse, moderately long, suberect to erect. Head foveolate; genae with smooth notches. Clypeus with fine and fading foveolae; median longitudinal carina absent; two lateral longitudinal carinae absent. Scape, when laid back, surpassing the posterior head margin by two-fifths of its length; pilosity suberect (Fig. 81A, C). ***Mesosoma*.** In lateral view, promesonotum low, slightly convex, short, with posterior declivity steep; promesonotal groove absent; metanotal groove indistinct; propodeal spines short, triangular, with acute apex (Fig. 81C). Sculpture foveolate; katepisternum and mesosoma smooth. Pilosity sparse, long, and erect (Fig. 81C, E). ***Petiole*.** Peduncle very short and thin with ventral face slightly convex; with few short, erect setae (Fig. 81C, E). ***Postpetiole*.** Short, low, and convex; with few short, erect setae (Fig. 81C, E). ***Petiole*.** With moderately sparse, erect pilosity (Fig. 81C, E). ***Colour*.** Unicolourous, dark yellow (Fig. 81C, E).

###### Etymology.

From the type locality.

###### Biology.

The species was collected at 1325 m in elevation, in montane rainforest. Nest was located in the petiole of Melastomataceae.

#### Revision of the *Pheidole lamperos* complex

**Diagnosis. Major worker.** Head, in full-face, view sub-rectangular, in lateral view sub-rectangular, ventral and dorsal faces relatively flat, dorsal face finely depressed posteriorly; antennal scrobes indistinct and not delimited by carinulae; occipital lobes with thick, irregular rugae, interspaces distinctly rugulose; antennal scrobes and frons with thick, longitudinal, and long rugae, interspaces smooth to indistinctly rugulose; genae with smooth posterior part; metanotal groove absent; propodeal spines moderately long; promesonotum, katepisternum, anepisternum, and dorsoventral surface of propodeum predominantly smooth, with very indistinct, short irregular rugulae; gaster finely shagreened; body black. **Minor worker.** Head with sparse but distinct foveolae and smooth interspaces, frons with additional longitudinal to irregular, thick rugae, vertex with thick, sparse, and arcuate rugae, genae smooth; scape, when laid back, surpassing the posterior head margin by one-fifth of its length; promesonotum, in lateral view, low, long, and slightly convex; propodeal spines short, triangular; mesosoma with thick and sparse foveolae with smooth interspaces, promesonotum with additional sparse, thick, transverse rugae; body dark brown.

**Comments.** Major workers of this complex can be easily distinguished by a combination of the following characters: head, in full-face and lateral view, sub-rectangular; antennal scrobes with smooth to indistinctly rugulae interspaces between rugae; mesosoma predominantly smooth, with very indistinct, short, irregular rugulae, and black body colouration. Minor workers can be distinguished from other groups by sparse but distinct foveolae with smooth interspaces covering head and mesosoma, additional arcuate rugae on vertex and transverse rugae on promesonotum, and dark brown body colouration.

##### Pheidole lamperos sp. nov.

Taxon classificationAnimaliaHymenopteraFormicidae

http://zoobank.org/1636E403-170C-4F0B-9D13-684F71CD8C92

**Figure 82.**
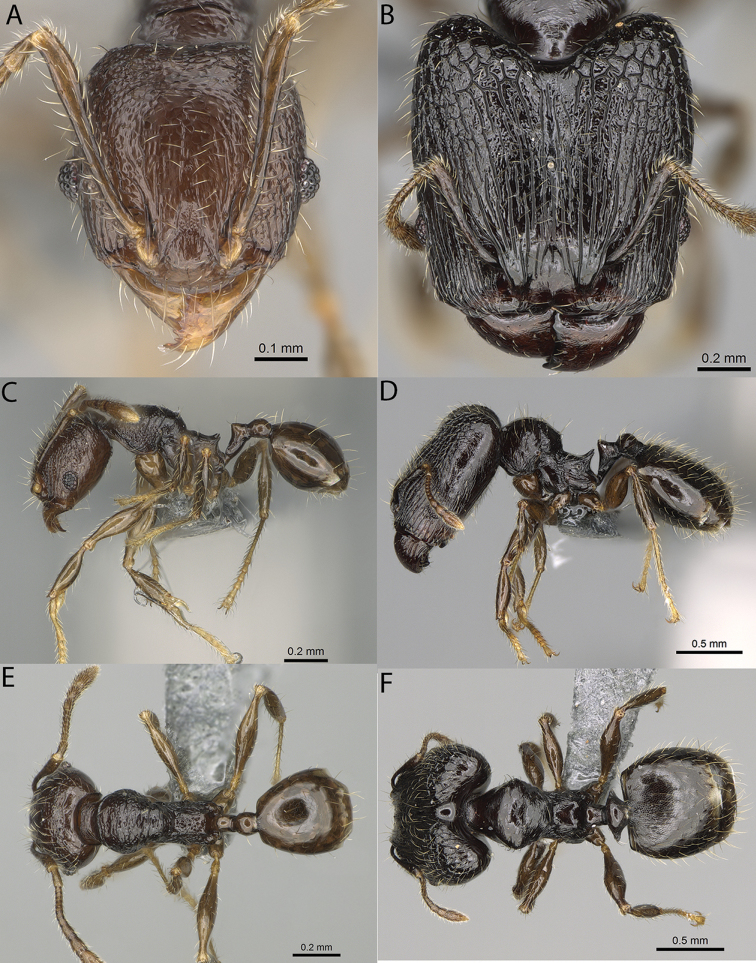
*Pheidole lamperos* sp. nov., full-face view (**A**), profile (**C**), and dorsal view (**E**) of paratype minor worker (CASENT0923181) and full-face view (**B**), profile (**D**), and dorsal view (**F**) of holotype major worker (CASENT0300132).

Figs 82A–F , 84W , 87A

###### Type material.

***Holotype*.** Madagascar. •1 major worker; Antsiranana; Galoko chain, Mont Galoko; -13.5888, 48.72864; alt. 980 m; 20 Feb 2013; B.L. Fisher et al. leg.; BLF30940, CASENT0300132 (CASC). ***Paratypes*.** Madagascar. •2w., 1q.; same data as for holotype; CASENT0300131, CASENT0923181, CASENT0872205 (CASC).

###### Other material.

Madagascar. – ***Antsiranana***: •2w., 1s., 1m.; Galoko chain, Mont Galoko; -13.59358, 48.73157; alt. 1100 m; 22 Feb 2012; B.L. Fisher et al. leg.; CASENT0301011, CASENT0301013 (CASC).

###### Diagnosis.

***Major workers*.** Head sub-rectangular; body black; sides of the head with moderately sparse, long, erect pilosity; frons with moderately dense, thick, longitudinal, and interrupted rugae, interspaces smooth to indistinctly rugulose; genae shiny, with sparse and thin, irregular rugoreticulation, posterior part smooth; inner hypostomal teeth distinct, moderately high, closely spaced, triangular, with rounded apex and wide base; outer hypostomal teeth thinner, smaller, and narrower than inner hypostomal teeth, dentate. ***Minor workers*.** Body dark brown; head with sparse but distinct foveolae, and additional longitudinal to irregular, thick rugae on frons and malar area, and thick, sparse, and arcuate rugae on vertex; mesosoma with thick and sparse foveolae; promesonotum with additional sparse, thick, transverse rugae; lateral sides of propodeum with thick, longitudinal rugae; katepisternum and mesonotum smooth.

###### Description.

**Major workers.** Measurements (*N* = 2): HL: 1.16–1.13; HW: 1.08–1.02; SL: 0.46–0.5; EL: 0.16–0.13; WL: 0.88–0.82; PSL: 0.18–0.17; MTL: 0.47–0.46; PNW: 0.55–0.49; PTW: 0.15–0.13; PPW: 0.41–0.32; CI: 93.1–90.1; SI: 42.7–48.8; PSLI: 15.7–14.8; PPI: 36.7–41.6; PNI: 50.9–48.1; MTI: 43.5–44.7. ***Head*.** In full-face view longer than wide, anterior of eyes straight, posterior of eyes relatively straight (Fig. 82B). In lateral view sub-rectangular; ventral and dorsal faces relatively flat; dorsal face finely depressed posteriorly, forming shallow transverse depression between frons and occipital lobes; inner hypostomal teeth visible. Sides of the head with moderately sparse, long, erect pilosity; whole head with dense, short, suberect to erect pilosity. Antennal scrobes indistinct and not delimited by carinulae; scrobe surface shiny, with thick, longitudinal, and long rugae; interspaces smooth to indistinctly rugulose. Occipital lobes shiny, with thick, irregular rugae, interspaces with distinct, irregular rugulae fading posteriorly; frons with moderately dense, thick, longitudinal, and interrupted rugae, interspaces smooth to indistinctly rugulose; genae shiny, with sparse and thin, irregular rugoreticulation, posterior part smooth; malar area with thin, longitudinal, dense rugae. Centre of clypeus shiny and smooth, lateral sides with longitudinal rugulae; median notch present, narrow and moderately shallow; median longitudinal carina present, indistinct; lateral longitudinal carinae present. Scape, when laid back, reaching the midlength of head; pilosity erect (Fig. 82B, D). Inner hypostomal teeth distinct, moderately high, closely spaced, triangular, with rounded apex and wide base; outer hypostomal teeth thinner, smaller, and narrower than inner hypostomal teeth, dentate (Fig. 84W). ***Mesosoma*.** In lateral view, promesonotum relatively low and arched, posterior mesonotum relatively steep, without tubercle-like projections; promesonotal groove absent; metanotal groove absent; propodeal spines moderately long, triangular, with rounded apex and wide base; humeral area laterally weakly produced (Fig. 82D). Surface shiny, promesonotum, katepisternum, anepisternum, and dorsoventral surface of propodeum smooth, with very indistinct, short irregular rugulae, only lower part of lateral sides of propodeum with thin, longitudinal rugulae; lateral sides of propodeum shiny, with thick, longitudinal rugae. Pilosity moderately sparse, long, and erect (Fig. 82D, F). ***Petiole*.** Shiny; peduncle short, finely foveolate, with indistinct horizontal lobes on its basal part; node smooth, relatively high, triangular, with rounded apex, in rear view node slightly convex; pilosity moderately sparse and erect (Fig. 82D, F). ***Postpetiole*.** Shiny, finely shagreened, with smooth dorsum; in dorsal view sides with moderately long, acute, wide, and triangular projections; pilosity moderately long, and erect (Fig. 82D, F). ***Petiole*.** Shiny and finely shagreened; pilosity dense, moderately long, and erect (Fig. 82D, F). ***Colour*.** Black; legs brownish black (Fig. 82D, F).

**Minor workers.** Measurements (*N* = 4): HL: 0.47–0.48 (0.48); HW: 0.44–0.46 (0.44); SL: 0.41–0.44 (0.43); EL: 0.08–0.09 (0.09); WL: 0.55–0.58 (0.56); PSL: 0.09–0.1 (0.09); MTL: 0.31–0.33 (0.32); PNW: 0.28–0.29 (0.29); PTW: 0.06–0.06 (0.06); PPW: 0.1–0.11 (0.1); CI: 92.4–95.4 (93.7); SI: 92.5–97.6 (95.6); PSLI: 19.0–19.6 (19.4); PPI: 55.3–59.4 (58.0); PNI: 63.2–64.5 (63.9); MTI: 70.1–73.4 (72.0). ***Head*.** Occipital margin straight or indistinctly concave; occipital carina present, weakly developed (Fig. 82A). Pilosity sparse, long, suberect to erect. Head shiny, with sparse but distinct foveolae, and additional longitudinal to irregular, thick rugae on frons and malar area, and thick, sparse, and arcuate rugae on vertex; genae and malar area smooth. Clypeus smooth, with basal part finely rugulose; median longitudinal carina present; two lateral longitudinal carinae indistinct. Scape, when laid back, surpassing the posterior head margin by one-fifth of its length; pilosity erect (Fig. 82A, C). ***Mesosoma*.** In lateral view, promesonotum low, slightly convex, long, with posterior declivity smoothly declining towards propodeum; promesonotal groove absent; metanotal groove absent; propodeal spines short, triangular, with acute apex (Fig. 82C). Sculpture with thick and sparse foveolae; promesonotum with additional sparse, thick, transverse rugae; lateral sides of propodeum with thick, longitudinal rugae; katepisternum and mesonotum smooth. Pilosity sparse, moderately long, and decumbent to erect (Fig. 82C, E). ***Petiole*.** Peduncle short and thin with ventral face slightly convex; with few short, erect setae (Fig. 82C, E). ***Postpetiole*.** Short, low and convex; with few short, erect setae (Fig. 82C, E). ***Petiole*.** With sparse, erect pilosity (Fig. 82C, E). ***Colour*.** Dark brown (Fig. 82C, E).

###### Etymology.

Greek for shiny [?aµpe???], in reference to dark and very shiny sculpture of minor and major workers.

###### Biology.

The species was collected at elevation between 980–1100 meters, in montane forest. Nests were located in rotten logs.

#### Revision of the *Pheidole zavamanira* complex

**Diagnosis. *Major workers*.** Head in full-face view oval, in lateral view sub-rectangular; ventral and dorsal faces relatively flat and dorsal face not depressed posteriorly; antennal scrobes absent; lateral sides of head foveolate with a few thin, irregular to longitudinal short rugae; occipital lobes shiny, with fine and sparse foveolae and sparse, thick, irregular rugae, foveolae fading posteriorly; genae shiny, with dense and thin rugulae, central part smooth; metanotal groove absent; propodeal spines moderately long; mesosoma with fine and dense foveolae and additional indistinct, irregular, short rugulae on promesonotum, katepisternum smooth; first gastral tergite finely shagreened; body bright brown. ***Minor workers*.** Head foveolate, frons with a few indistinct, short, longitudinal rugulae; scape, when laid back, surpassing the posterior head margin by two-fifths of its length; promesonotum low, convex, moderately long, with posterior declivity steep; propodeal spines short, triangular; mesosoma foveolate; body smoky yellow.

**Comments.** Major workers of this complex can be distinguished based on a combination of the following characters: head in full-face view oval, in lateral view sub-rectangular; lateral sides of head foveolate with a few indistinct rugae; genae with smooth notches; moderately long propodeal spines; smooth katepisternum, and finely shagreened first gastral tergite. Minor workers can be separated based on foveolate head and mesosoma, frons with a few indistinct rugulae; short propodeal spines; and smoky yellow body colouration.

##### Pheidole zavamanira sp. nov.

Taxon classificationAnimaliaHymenopteraFormicidae

http://zoobank.org/9530D7EB-52ED-4FCC-A448-2B91E51846B6

**Figure 83.**
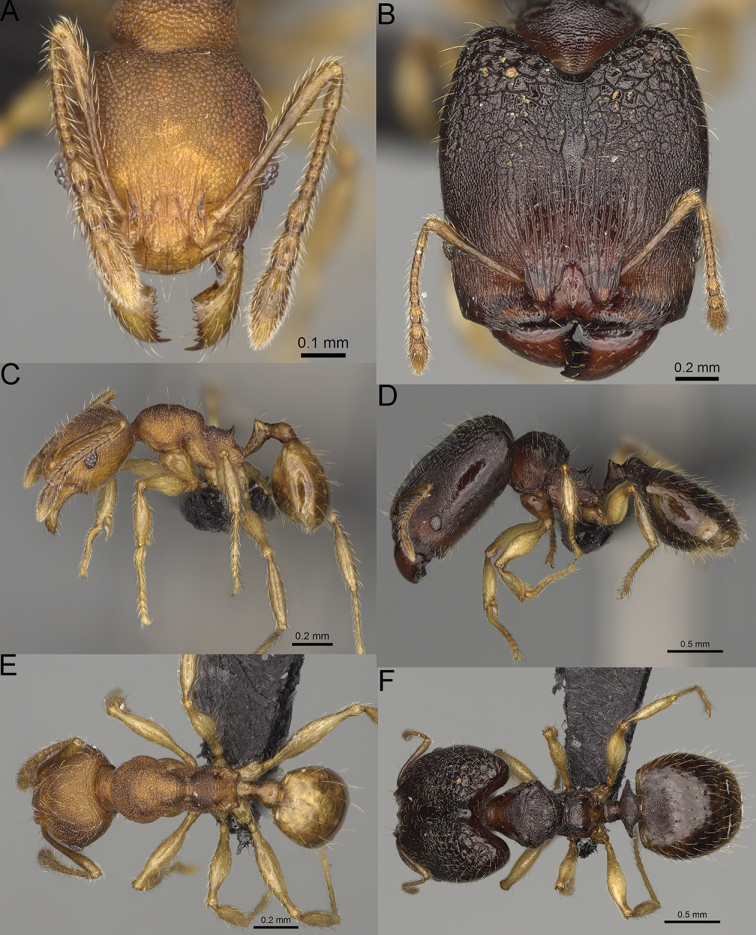
*Pheidole zavamanira* sp. nov., full-face view (**A**), profile (**C**), and dorsal view (**E**) of paratype minor worker (CASENT0485890) and full-face view (**B**), profile (**D**), and dorsal view (**F**) of holotype major worker (CASENT0485886).

Figs 83A–F , 85A, B , 88L

###### Type material.

***Holotype*.** Madagascar. •1 major worker; Toliara; Réserve Spéciale d’Ambohijanahary, Forêt d’Ankazotsihitafototra, 35.2 km 312°NW Ambaravaranala; -18.26667, 45.40667; alt. 1050 m; 13 Jan 2003; B.L. Fisher et al. leg.; BLF07069, CASENT0485886, bottom specimen (CASC). ***Paratypes*.** Madagascar. •11w., 1s.; same data as for holotype; CASENT0872081, CASENT0485887–CASENT0485890, CASENT0872198–CASENT0872203 (CASC).

###### Other material.

Madagascar. – ***Toliara***: •1s.; Réserve Spéciale d’Ambohijanahary, Forêt d’Ankazotsihitafototra, 35.2 km 312°NW Ambaravaranala; -18.26667, 45.40667; alt. 1050 m; 13 Jan 2003; Fisher et al. leg.; CASENT0028216 (CASC). •2s.; Réserve Spéciale d’Ambohijanahary, Forêt d’Ankazotsihitafototra, 34.6 km 314°NW Ambaravaranala; -18.26, 45.41833; alt. 1100 m; 16 Jan 2003; Fisher et al. leg.; CASENT0029511, CASENT0029704 (CASC).

###### Diagnosis.

***Major workers*.** Head in full-face view oval, relatively as long as wide, anterior and posterior of eyes moderately convex; sides of the head with sparse, short, erect pilosity; genae shiny, with dense and thin rugulae, central part smooth; inner hypostomal teeth distinct, closely spaced, moderately low, triangular, with rounded apex directed inward; outer hypostomal teeth thinner and higher than inner hypostomal teeth, dentate, and with relatively wide base; propodeal spines moderately long; first gastral tergite finely shagreened; body bright brown. ***Minor workers*.** Head foveolate; frons with a few indistinct, short, longitudinal rugulae; promesonotum low, convex, moderately long, with posterior declivity steep; propodeal spines short; mesosoma foveolate; body dark yellow.

###### Description.

**Major workers.** Measurements (*N* = 4): HL: 1.4–1.52 (1.46); HW: 1.26–1.33 (1.3); SL: 0.54–0.58 (0.57); EL: 0.15–0.17 (0.16); WL: 0.92–0.97 (0.94); PSL: 0.19–0.23 (0.2); MTL: 0.56–0.58 (0.57); PNW: 0.65–0.72 (0.67); PTW: 0.15–0.19 (0.18); PPW: 0.55–0.66 (0.58); CI: 87.8–90.4 (88.9); SI: 42.1–44.3 (43.6); PSLI: 12.9–15.1 (13.8); PPI: 27.8–33.4 (30.3); PNI: 49.1–54.3 (51.5); MTI: 43.2 –45.2 (44.2). ***Head*.** In full-face view oval, anterior and posterior of eyes moderately convex (Fig. 83B). In lateral view sub-rectangular; ventral and dorsal faces relatively flat; dorsal face not depressed posteriorly; inner hypostomal teeth visible. Sides of the head with sparse, short, erect pilosity; whole head with sparse, long, suberect to erect pilosity. Antennal scrobes absent; lateral sides of head shiny, foveolate with a few thin, irregular to longitudinal short rugae. Occipital lobes shiny, with fine and sparse foveolae and sparse, thick, irregular rugae, foveolae fading posteriorly; frons on the anterior part with sparse, thick, and longitudinal rugae, interspaces smooth, and posterior part with rugae thinner and more irregular, interspaces foveolate; genae shiny, with dense and thin rugulae, central part smooth; malar area with thin, longitudinal, dense rugoreticulation. Centre of clypeus shiny with indistinct longitudinal rugulae, lateral sides with longitudinal rugae; median notch present, moderately wide, and shallow; median longitudinal carina present; lateral longitudinal carinae present. Scape, when laid back, reaching the midlength of head; pilosity suberect to erect (Fig. 83B, D). Inner hypostomal teeth distinct, closely spaced, moderately low, triangular, with rounded apex directed inward; outer hypostomal teeth thinner and higher then inner hypostomal teeth, dentate, and with relatively wide base (Fig. 85A, B). ***Mesosoma*.** In lateral view, promesonotum moderately low and arched, posterior mesonotum steep, without tubercle-like projections; promesonotal groove absent; metanotal groove absent; propodeal spines moderately long, triangular, with rounded apex and wide base; humeral area laterally well produced (Fig. 83D). Surface shiny, with fine and dense foveolae; promesonotal dorsum with additional indistinct, irregular, short rugulae; katepisternum smooth. Pilosity sparse, long, and erect (Fig. 83D, F). ***Petiole*.** Shiny and with fine foveolae; peduncle short, with indistinct horizontal lobes on its basal part; node relatively high, triangular, with rounded apex, in rear view node slightly convex; pilosity moderately sparse and erect (Fig. 83D, F). ***Postpetiole*.** Shiny, finely shagreened; in dorsal view sides with long, acute, and triangular projections; pilosity long, moderately sparse, and erect (Fig. 83D, F). ***Petiole*.** Shiny, first gastral tergite finely shagreened; pilosity moderately sparse, long, and erect (Fig. 83D, F). ***Colour*.** Unicolourous, bright brown; lower part of frons and malar area brighter that the rest of head (Fig. 83D, F).

**Minor workers.** Measurements (*N* = 10): HL: 0.49–0.52 (0.51); HW: 0.46–0.48 (0.47); SL: 0.47–0.49 (0.48); EL: 0.09–0.11 (0.1); WL: 0.54–0.59 (0.57); PSL: 0.09–0.11 (0.1); MTL: 0.36–0.38 (0.37); PNW: 0.3–0.32 (0.31); PTW: 0.06–0.08 (0.07); PPW: 0.12–0.14 (0.13); CI: 89.9–94.5 (92.0); SI: 98.9–105.8 (103.0); PSLI: 16.7–22.8 (19.6); PPI: 50.0–60.7 (54.6); PNI: 64.2–69.0 (66.8); MTI: 76.2–80.6 (78.6). ***Head*.** Occipital margin straight or indistinctly concave; occipital carina absent (Fig. 83A). Pilosity moderately sparse, moderately long, suberect to erect. Head foveolate; frons with a few indistinct, short, longitudinal rugulae. Clypeus with fine and fading foveolae; median longitudinal carina present; two lateral longitudinal carinae present. Scape, when laid back, surpassing the posterior head margin by two-fifths of its length; pilosity suberect (Fig. 83A, C). ***Mesosoma*.** In lateral view, promesonotum low, convex, moderately long, with posterior declivity steep; promesonotal groove absent; metanotal groove distinct; propodeal spines short, triangular, with acute apex (Fig. 83C). Sculpture foveolate. Pilosity moderately sparse, long, and erect (Fig. 83C, E). ***Petiole*.** Peduncle short and thin with ventral face slightly convex; with few short, erect setae (Fig. 83C, E). ***Postpetiole*.** Short, low, and convex; with few short, erect setae (Fig. 83C, E). ***Petiole*.** With sparse, erect pilosity (Fig. 83C, E). ***Colour*.** Smoky yellow; lower part of frons, malar area and lateral sides of mesosoma yellow (Fig. 83C, E).

**Figure 84.**
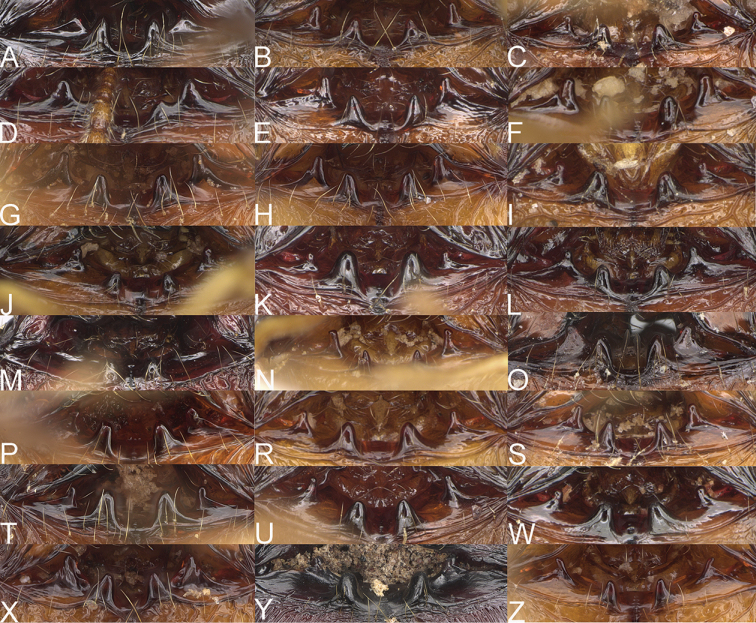
Hypostomal teeth. *Pheidole aelloea* sp. nov. (**A**). *P. ala* sp. nov. (**B**). *P. andapa* sp. nov. (**C**). *P. ankerana* sp. nov. (**D**). *P. annemariae* Forel (**E**). *P. avaratra* sp. nov. (**F**). *P. bemarahaensis* sp. nov. (**G**). *P. bemarivoensis* sp. nov. (**H**). *P. binara* sp. nov. (**I**). *P. boribora* sp. nov. (**J**). *P. brevipilosa* sp. nov. (**K**). *P. curvistriata* sp. nov. (**L**). *P. diakritos* sp. nov. (**M**). *P. ehazoara* sp. nov. (**N**). *P. ensifera* Forel (**O**). *P. ferruginea* sp. nov. (**P**). *P. fisaka* sp. nov. (**R**). *P. fitarata* sp. nov. (**S**). *P. glabra* sp. nov. (**T**). *P. goavana* sp. nov. (**U**). *P. lamperos* sp. nov. (**W**). *P. longipilosa* sp. nov. (**X**). *P. longispinosa* Forel (**Y**). *P. lutea* sp. nov. (**Z**).

**Figure 85.**
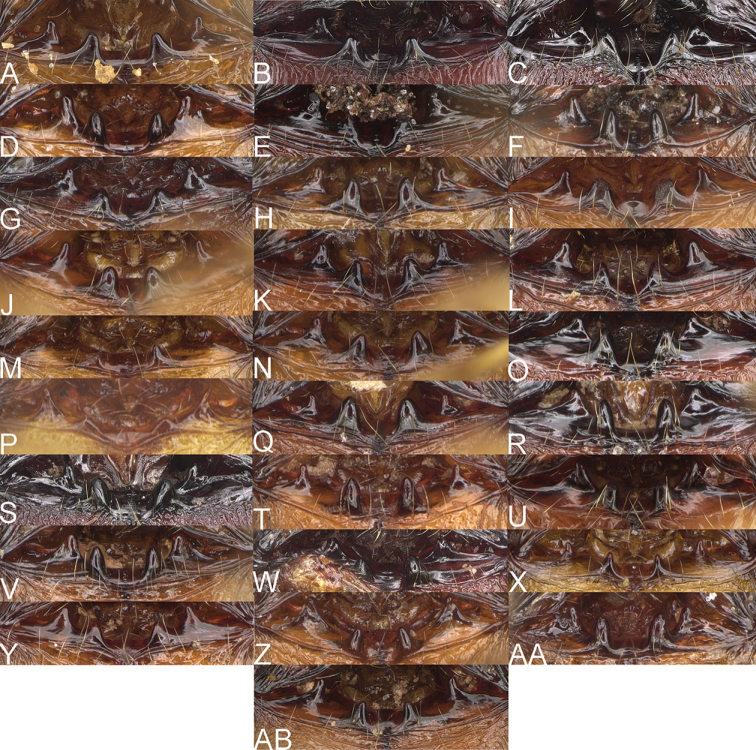
Hypostomal teeth. *Pheidole madinika* sp. nov. (**A**). *P. mahaboensis* sp. nov. (**B**). *P. maizina* sp. nov. (**C**). *P. makaensis* sp. nov. (**D**). *P. makirovana* sp. nov. (**E**). *P. manantenensis* sp. nov. (**F**). *P. mantadia* sp. nov. (**G**). *P. marieannae* sp. nov. (**H**). *P. masoala* sp. nov. (**I**). *P. mavesatra* sp. nov. (**J**). *P. miramila* sp. nov. (**K**). *P. moramanaensis* sp. nov. (**L**). *P. navoatrensis* sp. nov. (**M**). *P. nemoralis* Forel (**N**). *P. ocypodea* sp. nov. (**O**). *P. parviocula* sp. nov. (**P**). *P. petax* Forel (**Q**). *P. podargea* sp. nov. (**R**). *P. praegrandis* sp. nov. (**S**). *P. ranohirensis* sp. nov. (**T**). *P. rugocephala* sp. nov. (**U**). *P. rugofitarata* sp. nov. (**V**). *P. scabrata* Forel (**W**). *P. typhlos* sp. nov. (**X**). *P. vatovavensis* sp. nov. (**Y**). *P. voasara* sp. nov. (**Z**). *P. vohemarensis* sp. nov. (**AA**). *P. zavamanira* sp. nov. (**AB**).

**Figure 86.**
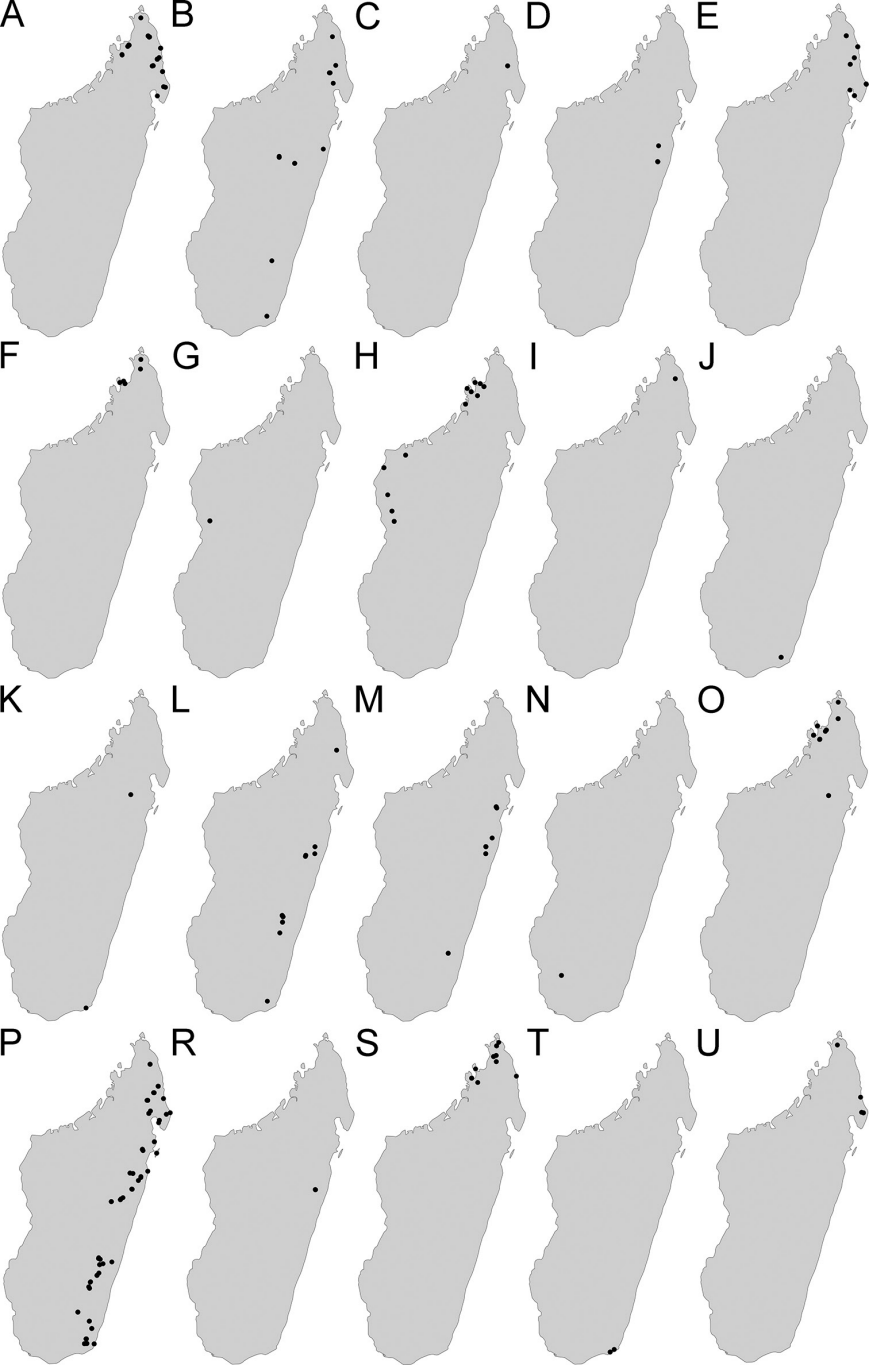
Distribution. *Pheidole aelloea* sp. nov. (**A**). *P. ala* sp. nov. (**B**). *P. andapa* sp. nov. (**C**). *P. ankerana* sp. nov. (**D**). *P. annemariae* Forel (**E**). *P. avaratra* sp. nov. (**F**). *P. bemarahaensis* sp. nov. (**G**). *P. bemarivoensis* sp. nov. (**H**). *P. binara* sp. nov. (**I**). *P. boribora* sp. nov. (**J**). *P. brevipilosa* sp. nov. (**K**). *P. curvistriata* sp. nov. (**L**). *P. diakritos* sp. nov. (**M**). *P. ehazoara* sp. nov. (**N**). *P. ensifera* Forel (**O**). *P. ferruginea* sp. nov. (**P**). *P. fisaka* sp. nov. (**R**). *P. fitarata* sp. nov. (**S**). *P. glabra* sp. nov. (**T**). *P. goavana* sp. nov. (**U**).

**Figure 87.**
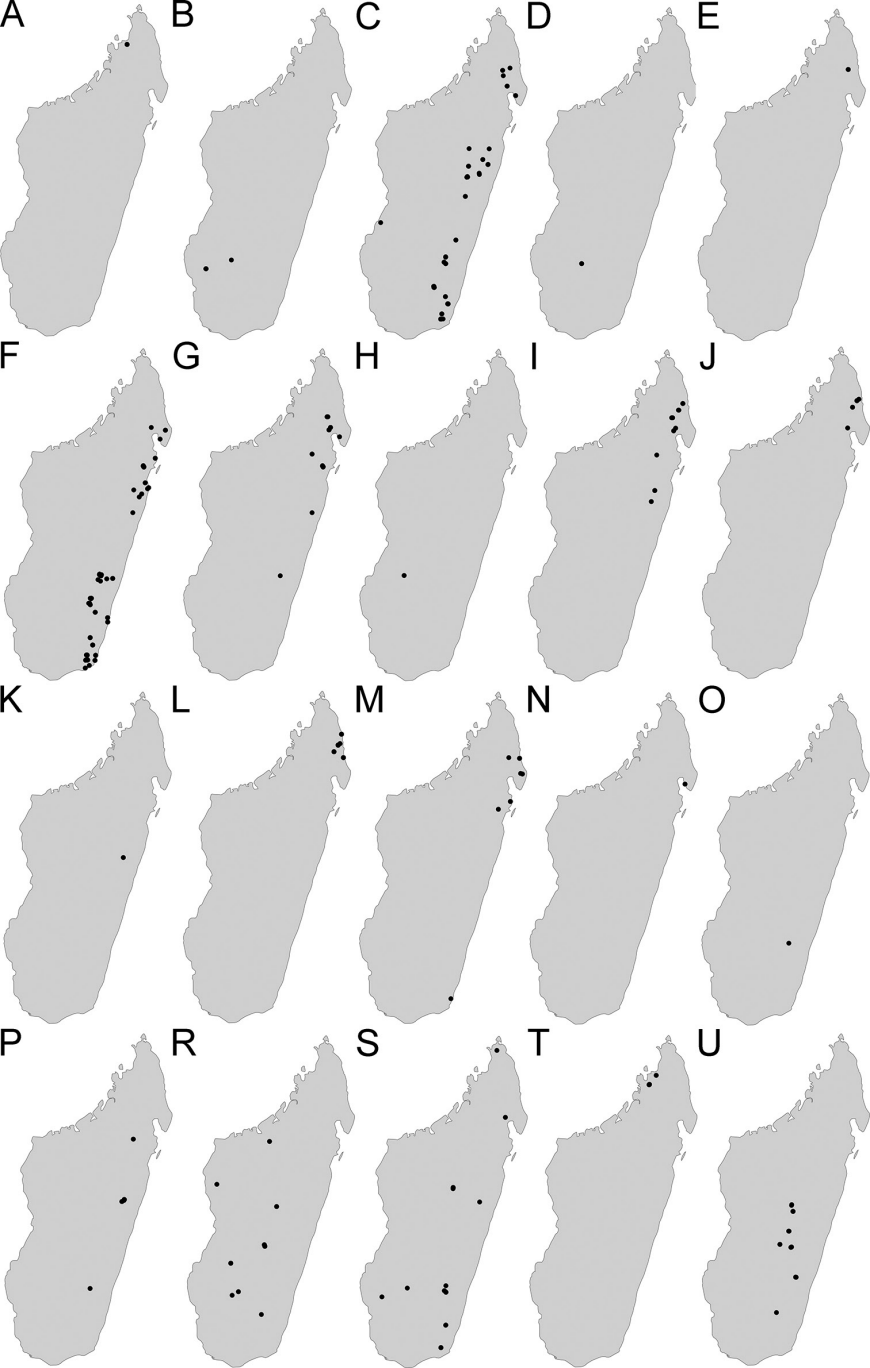
Distribution. *Pheidole lamperos* sp. nov. (**A**). *P. longipilosa* sp. nov. (**B**). *P. longispinosa* Forel (**C**). *P. lutea* sp. nov. (**D**). *P. madinika* sp. nov. (**E**). *P. mahaboensis* sp. nov. (**F**). *P. maizina* sp. nov. (**G**). *P. makaensis* sp. nov. (**H**). *P. makirovana* sp. nov. (**I**). *P. manantenensis* sp. nov. (**J**). *P. mantadia* sp. nov. (**K**). *P. marieannae* sp. nov. (**L**). *P. masoala* sp. nov. (**M**). *P. mavesatra* sp. nov. (**N**). *P. miramila* sp. nov. (**O**). *P. moramanaensis* sp. nov. (**P**). *P. navoatrensis* sp. nov. (**R**). *P. nemoralis* Forel (**S**). *P. ocypodea* sp. nov. (**T**). *P. parviocula* sp. nov. (**U**).

**Figure 88.**
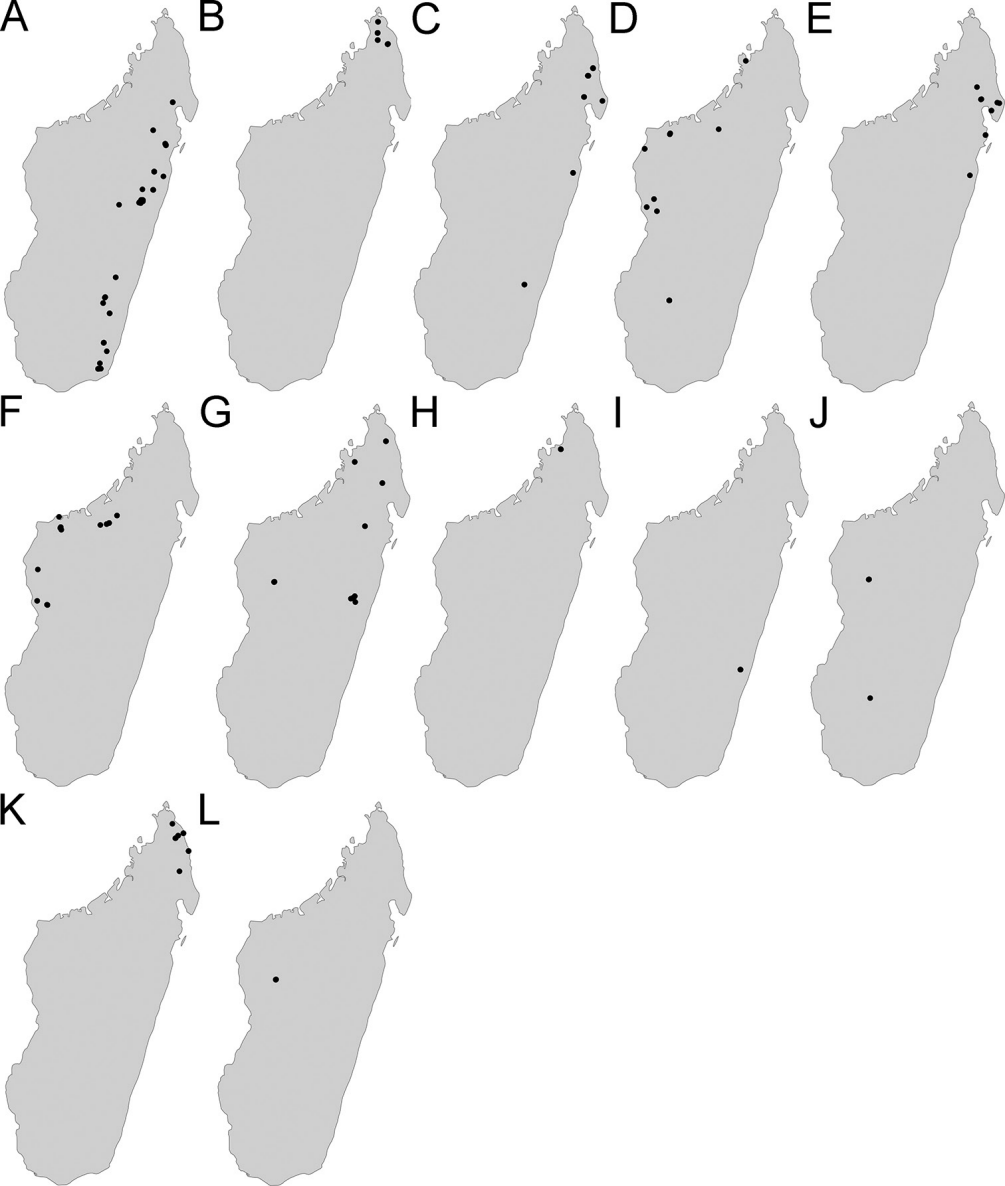
Distribution. *Pheidole petax* Forel (**A**). *P. podargea* sp. nov. (**B**). *P. praegrandis* sp. nov. (**C**). *P. ranohirensis* sp. nov. (**D**). *P. rugocephala* sp. nov. (**E**). *P. rugofitarata* sp. nov. (**F**). *P. scabrata* Forel (**G**). *P. typhlos* sp. nov. (**H**). *P. vatovavensis* sp. nov. (**I**). *P. voasara* sp. nov. (**J**). *P. vohemarensis* sp. nov. (**K**). *P. zavamanira* sp. nov. (**L**).

###### Etymology.

Malagasy for plants, in reference to nesting habits of this species.

###### Biology.

The species was collected between 1050–1100 m in elevation, in montane rainforest. Nests were located in rotten logs.

## Supplementary Material

XML Treatment for Pheidole diakritos

XML Treatment for Pheidole lutea

XML Treatment for Pheidole ranohirensis

XML Treatment for Pheidole voasara

XML Treatment for Pheidole navoatrensis

XML Treatment for Pheidole parviocula

XML Treatment for Pheidole typhlos

XML Treatment for Pheidole longispinosa

XML Treatment for Pheidole mahaboensis

XML Treatment for Pheidole praegrandis

XML Treatment for Pheidole scabrata

XML Treatment for Pheidole maizina

XML Treatment for Pheidole ensifera

XML Treatment for Pheidole aelloea

XML Treatment for Pheidole ocypodea

XML Treatment for Pheidole podargea

XML Treatment for Pheidole ferruginea

XML Treatment for Pheidole rugocephala

XML Treatment for Pheidole manantenensis

XML Treatment for Pheidole vohemarensis

XML Treatment for Pheidole longipilosa

XML Treatment for Pheidole annemariae

XML Treatment for Pheidole marieannae

XML Treatment for Pheidole makaensis

XML Treatment for Pheidole fitarata

XML Treatment for Pheidole rugofitarata

XML Treatment for Pheidole avaratra

XML Treatment for Pheidole ehazoara

XML Treatment for Pheidole curvistriata

XML Treatment for Pheidole moramanaensis

XML Treatment for Pheidole makirovana

XML Treatment for Pheidole mantadia

XML Treatment for Pheidole nemoralis

XML Treatment for Pheidole ala

XML Treatment for Pheidole bemarivoensis

XML Treatment for Pheidole bemarahaensis

XML Treatment for Pheidole petax

XML Treatment for Pheidole brevipilosa

XML Treatment for Pheidole glabra

XML Treatment for Pheidole mavesatra

XML Treatment for Pheidole goavana

XML Treatment for Pheidole ankerana

XML Treatment for Pheidole vatovavensis

XML Treatment for Pheidole boribora

XML Treatment for Pheidole miramila

XML Treatment for Pheidole masoala

XML Treatment for Pheidole madinika

XML Treatment for Pheidole fisaka

XML Treatment for Pheidole binara

XML Treatment for Pheidole andapa

XML Treatment for Pheidole lamperos

XML Treatment for Pheidole zavamanira

## Footnotes

Note: If outer hypostomal teeth are lobe-like, bigger and wider than inner hypostomal teeth (Fig. 13K) then see a diagnosis of P. voasara sp. nov. of which minor workers are unknown.

Note: If outer hypostomal teeth are lobe-like and relatively as high as outer hypostomal teeth (Fig. 43L), and sculpture on katepisternum is fading (Fig. 44B), then see description of Pheidole rugofitarata sp. nov., for which minor workers are unknown.
